# Tandem Duplication‐Driven Neofunctionalization of UDP‐Glycosyltransferases Shapes the Diversification of Triterpenoid Saponins in the Cucurbitaceae

**DOI:** 10.1002/advs.76159

**Published:** 2026-06-18

**Authors:** Guangyi Wang, Mengmeng Li, Xuehui Dai, Yanchen Zhang, Yuhan Wu, Zhaotao Yan, Chenfei Tian, Hongkai Fan, Haili Liu, Xiaowei Zhang, Yiming Yang, Jiayu Lu, Yuwei Sun, Yong Wang

**Affiliations:** ^1^ CAS‐Key Laboratory of Synthetic Biology CAS Center for Excellence in Molecular Plant Sciences Institute of Plant Physiology and Ecology Chinese Academy of Sciences Shanghai China; ^2^ University of Chinese Academy of Sciences Beijing China; ^3^ State Key Laboratory of Bioreactor Engineering School of Biotechnology East China University of Science and Technology Shanghai China; ^4^ Technology Center Shanghai Tobacco Group Co., Ltd. Shanghai China; ^5^ The SATCM Key Laboratory for New Resources & Quality Evaluation of Chinese Medicine Institute of Chinese Materia Medica Shanghai University of Traditional Chinese Medicine Shanghai China

**Keywords:** comparative genomics, cucurbitaceae, tandem duplication, triterpenoid saponins, UDP‐glycosyltransferase

## Abstract

Tandem duplication of tailoring enzymes allows evolutionary innovation that diversifies plant specialized metabolism. Here, we present an interesting example of how tandem duplicated UDP‐glycosyltransferases undergo neofunctionalization and shape the chemical diversity of triterpenoid saponins in the Cucurbitaceae family. A chromosome‐level genome of *Siraitia grosvenorii* was assembled and aligned with multiple cucurbit genomes, revealing a specific UGT73AM tandem duplication responsible for regio‐selective glycosylation (e.g. the rare 1,4‐linked disaccharide) of diverse saponins such as mogrosides, ginsenosides, and momordicines. Comparative genomics depicted the evolutionary trajectory of a universal saponin‐biosynthesizing UGT73 tandem arrays syntenously preserved across core eudicots, where lineage‐specific UGT copies contribute to distinct metabolic phenotypes. A crystal structure of SgUGT73AM30 (mogrol 25‐*O*‐glycosyltransferase) in complex with UDP and mogrol was obtained to elucidate the molecular basis of the regio‐specific decoration on vicinal diol of the substrates. Altogether, these findings provide insights into tandem duplication‐driven diversification of glycosyltransferases and lay the foundation for engineered glycosylation of valuable triterpenoid saponins.

## Graphical abstract

We present a chromosome‐level genome assembly of *Siraitia grosvenorii* and, through comparative genomics, uncover a conserved UGT73 tandem array driving triterpenoid saponin diversification in Cucurbitaceae. Crystalized SgUGT73AM30 further reveals the regioselectivity mechanism underlying its catalytic activity.

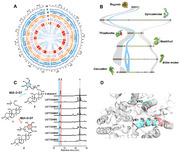

## 1 Introduction

Plants are ultimate chemists capable of synthesizing chemicals with extraordinary structural diversity. As an important class of plant‐derived chemicals, triterpenoid saponins are specialized terpene glycosides widely distributed in land plants, which play a crucial role in host defense, development, and shaping rhizosphere microbiota [1, 2]. These complex metabolites typically consist of a multicyclic C_30_ aglycone with one or more saccharide chains covalently bound to the different sites of the aglycone. The attachment of sugar chains (called glycosylation) in plant specialized metabolism is mainly governed by uridine diphosphate (UDP)‐dependent glycosyltransferases (UGTs), a class of GT1 family members utilizing UDP‐sugars as sugar donors [3]. Diverse glycosylation patterns endow triterpenoid saponins with remarkable physiological properties. For example, the trisaccharide side chain of avenacin A‐1 is essential for soil‐borne pathogens and disease resistance in oat (*Avena* species) [4]. Astragaloside IV from the dried root of *Astragalus* species, along with its analogues having different glycosylation patterns, could act as autophagy modulators [5]. Recently, QS21, a triterpenoid saponin derived from soapbark (*Quillaja saponaria*), has garnered considerable attention due to its significant importance as a vaccine adjuvant [6].

**FIGURE 1:**
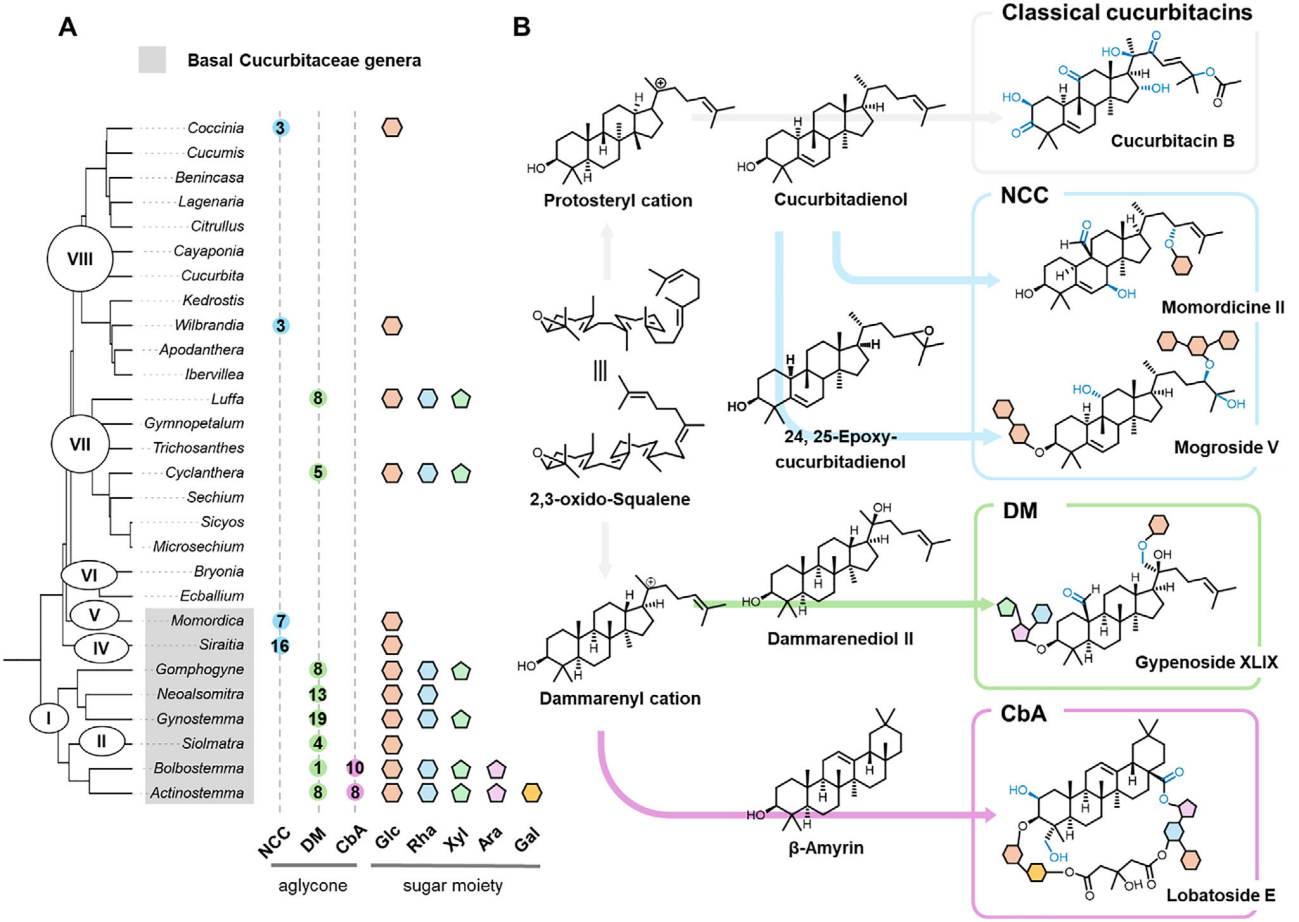
Chemical diversity of specialized terpenoid saponins in basal Cucurbitaceae genera. (A) Distribution of the non‐cucurbitacin terpenoids in Cucurbitaceae family. The basal Cucurbitaceae genera comprise Clade I–V. Specialized saponins of each genus recorded in literature are indicated by the numbers in discs. NCC, non‐canonical cucurbitacin saponins (with less than five oxidized sites); DM, dammarane‐derived saponins; CbA, cyclic beta‐amyrin‐derived saponins. Glc, Rha, Xyl, Ara, and Gal represent the d‐glucose, l‐rhamnose, d‐xylose, l‐arabinose, and d‐galactose moieties of specialized saponins, respectively. (B) Major biosynthetic pathway of specialized saponins in the Cucurbitaceae family. In brief, the ‘Chair‐Boat‐Chair’ cyclization of 2, 3‐oxidosqualene forms cucurbitadienol (the common precursor of classical cucurbitacins and NCC saponins) via the protosteryl cation, while the ‘Chair‐Chair‐Chair’ cyclization gives the dammarenyl cation, which further produces dammarenediol II and β‐amyrin (the precursors of DM and CbA saponins).

The Cucurbitaceae family (cucurbits) is a major contributor of natural triterpenoid saponins, encompassing 8 clades, 96 genera, and approximately 1000 species [7]. Species radiation occurring in Cucurbitaceae evolution, particularly during the Eocene climatic optimum, gave rise to several late‐diverged clades (Clade VI‐VIII) represented by many important cultivated gourds like cucumber (*Cucumis sativus*), watermelon (*Citrullus lanatus*), and pumpkin (*Cucurbita pepo*) (Figure 1A) [8]. These lately evolved cucurbits generally produce cucurbitadienol‐derived cucurbitacins, which confer bitterness and resistance to herbivores. While classical cucurbitacins are tetracyclic triterpenoids, highly oxygenated but barely glycosylated, previous phytochemical investigations have also uncovered that non‐canonical cucurbitacins (NCC) with less oxidation and diverse glycosylation are abundantly present in the basal genera *Siraitia* (monk fruit, Clade IV, Siraitieae) and *Momordica* (bitter melon, Clade V, Momordiceae). These findings indicate that the earlier‐diverging Cucurbitaceae species also serve as important sources of triterpenoid saponins (Figure 1A,B). Among the NCC found in basal cucurbit lineages, mogroside V is a characteristic saponin stored in the fruits of *Siraitia grosvenorii (S. grosvenorii)* and regarded as a promising next‐generation natural sweetener [9], meanwhile, momordicine II derived from the fruits of *M. charantia* is a promising anti‐diabetic agent [10] (Figure 1B). Except for *Siraitia* and *Momordica* species, other early‐diverged cucurbits (Clade I–III) have also evolved the abilities of biosynthesizing dammarane (DM)‐derived saponins and cyclic β‐amyrin (CbA)‐derived saponins. For instance, tubeimoside I from *Bolbostemma paniculatum* (Clade I, Actinostemmateae) exhibited potent antiviral activity [11]. The aerial parts of *Gynostemma pentaphyllum* (Clade I, Gomphogyneae) enrich the hepatoprotective saponin gypenoside XLIX [12] (Figure 1B). Notably, the chemical diversity in basal Cucurbitaceae is not confined to aglycones structures but also reflected in the richness of different glycosyls. There is a tendency that the more basal the species, the greater the diversity of sugar units installed in the glycan chains. For example, the sugar chain of a potent antitumor agent lobatoside E (isolated from *Actinostemma lobatum*, Clade I) comprises different types of sugars, including d‐glucose, l‐arabinose, l‐rhamnose, d‐galactose, and d‐xylose [13].

In sharp contrast to the remarkable chemical diversity and manifold bioactivity, the biosynthesis of triterpenoid saponins derived from basal Cucurbitaceae has been less studied. Over the past decades, substantial efforts have focused on deciphering biosynthetic pathways of classical cucurbitacins in different gourds, whereas those involved in NCC, DM, and CbA saponin biosynthesis remain largely unexplored. Hitherto, researchers have elucidated an intricate oxidation network involving cytochrome P450 monooxygenases (CYP87, CYP88 and CYP81) which decorate multiple sites of the cucurbitane skeleton by installing hydroxyl and keto groups on C7, C11, C19 and C25 of the classical cucurbitacins (Figure S1) [14, 15]. Similarly, CYP87D18 from *S. grosvenorii* catalyzes the oxidation of 24, 25‐epoxy‐cucurbitadienol at C11 position, providing precursors for mogrol, a common aglycone of all mogroside sweeteners [16], while in *M. charantia* CYP81AQ19, CYP88L7, and CYP88L8 respectively oxidize C23 of cucurbitadienol, form the C5‐C19 ether bridge, and introduce a C7‐β‐hydroxyl group in the biosynthesis of *Momordica* NCC saponins [17].

Unlike relatively well‐characterized oxidation enzymes, there are sporadic reports of Cucurbitaceae UGTs participating in the biosynthesis of classical cucurbitacins, ginsenosides (DM saponins), and mogrosides (NCC saponins) (Figure S2). These include cucurbitacin C 3‐*O*‐glucosyltransferase (UGT73AM3 from *C. sativus*) [18], cucurbitacin E 2‐*O*‐glucosyltransferase (UGT74F2 from *C. lanatus*) [19], and several *G. pentaphyllum* UGT71, UGT74, and UGT94 family glycosyltransferases that produce ginsenoside F2, Rh2, Rg3, and Rd [20]. In *S. grosvenorii*, the identified UGTs so far have been proved to glycosylate the aglycone mogrol at C3 (UGT74AC1 and UGT74AJ4) [21, 22] and C24 (UGT74DD1) [23]. Additional sugar‐chain‐extending UGT94‐289‐3 is responsible for the formation of downstream glycosides such as mogroside III and mogroside IVA, mainly through β‐(1→6)‐glucosylation, nevertheless the promiscuity of UGT94‐289‐3 renders the sugar chain elongation networks unclear [22]. Considering the wide variety of triterpenoid saponins in the basal Cucurbitaceae species, there is an apparent mismatch of the currently characterized UGTs to numerous unknown glycosylation steps involving diverse glycosyls and sugar linkages in the NCC, DM, and CbA saponin biosynthesis.

In most cucurbits, cytochrome P450 genes and cucurbitadienol synthase (CPQ) implicated in the classical/non‐canonical cucurbitacins pathway are organized into biosynthetic gene clusters within a conserved genomic region (Figure S3) [15], whereas many UGTs have been identified as members of tandem duplicated genes that could also be frequently observed in the genomes of Cucurbitaceae species [22]. Tandem duplication generates additional genetic elements serving as raw material for evolutionary innovation. Commonly, the duplicated gene copies first create a functional redundancy, acting as a “backup” to preserve essential functions. This phenomenon could be found in tailoring enzyme‐mediated plant specialized metabolism. For instance, three tandem‐duplicated *UGT73CB* genes in *Aralia elata* have been shown to uniformly add a glucose moiety at C3 of calenduloside E with similar gene expression pattern [24]. Duplication of *Isodon*‐specific *CYP706V* subfamily gene provided redundant *ent*‐kaurene‐hydroxylases and contributed to the biosynthesis of oridonin in *Isodon rubescens* [25]. Likewise, duplicated CYP71Ds in *Salvia miltiorrhiza* were proposed to be involved in the hetero‐cyclization of tanshinone biosynthesis [26]. Besides functional conservation, tandem duplicated tailoring enzymes in fact undergo neofunctionalization and subfunctionalization that profoundly drive the complexity of specialized metabolism, playing a crucial role in the diversification of metabolic phenotype [27]. Although there are reports on the “backup” manner of tandem duplicated UGTs, neofunctionalized UGT generated from the tandem duplication event and their actual functions are scarcely documented. With the increasing availability of high‐quality genomes across Cucurbitaceae, we are gaining more opportunities to unveil the tandem duplicated UGTs in Cucurbitaceae (especially in the basal clade). Nevertheless, the evolutionary trajectory underlying the formation of UGT tandem duplicates and their contribution to the diversity of triterpenoid saponin glycosylation remains poorly understood.

In the present study, a high‐quality chromosome‐level genome of *S. grosvenorii* was assembled and analyzed with multiple Cucurbitaceae assemblies. Through comparative genomics, we revealed a cucurbit‐specific tandem duplication of UGT73AM glycosyltransferases that underwent neofunctionalization to acquire distinct regio‐specific glycosylation capabilities, contributing to the saponin biosynthesis of mogroside in *Siraitia*, momordicine in *Momordica*, and ginsenoside in *Gynostemma*. We also found the syntenic UGT73 tandem duplication widely distributed in core eudicots, within which the emergence of distinctly evolved glycosyltransferase copies corresponds to the metabolic phenotype in particular plant species. SgUGT73AM30, a mogroside‐25‐*O*‐glucoside synthase from *S. grosvenorii*, was crystallized in complex with UDP and mogrol to elucidate the molecular mechanism underlying its regiospecific glycosylation. Structure‐guided mutagenesis indicated the α4 helix of SgUGT73AM30/SgUGT73AM19 pair was crucial for the regio‐selective decoration of vicinal diol on the side chain of mogrol. Taken together, these findings provide a mechanistic example of how tandemly duplicated UGTs can diversify in vitro regioselectivity and may contribute to metabolic diversification in related plant lineages.

## 2 Results

### 2.1 High Quality Genome Assembly of *S. grosvenorii*

Despite numerous documented cucurbit genomes, little attention has been paid to the basal lineages of Cucurbitaceae, especially those genera in Clade II (Zanonieae and Triceratieae) and Clade IV (Siraitieae) (Figure S4) [8]. In the 224 Cucurbitaceae genomes sequenced so far, only 30 (13.4%) belong to the basal clades, and most of them are poorly assembled with scaffold N50 less than 1 Mb (Figure S4B,C).

**FIGURE 2:**
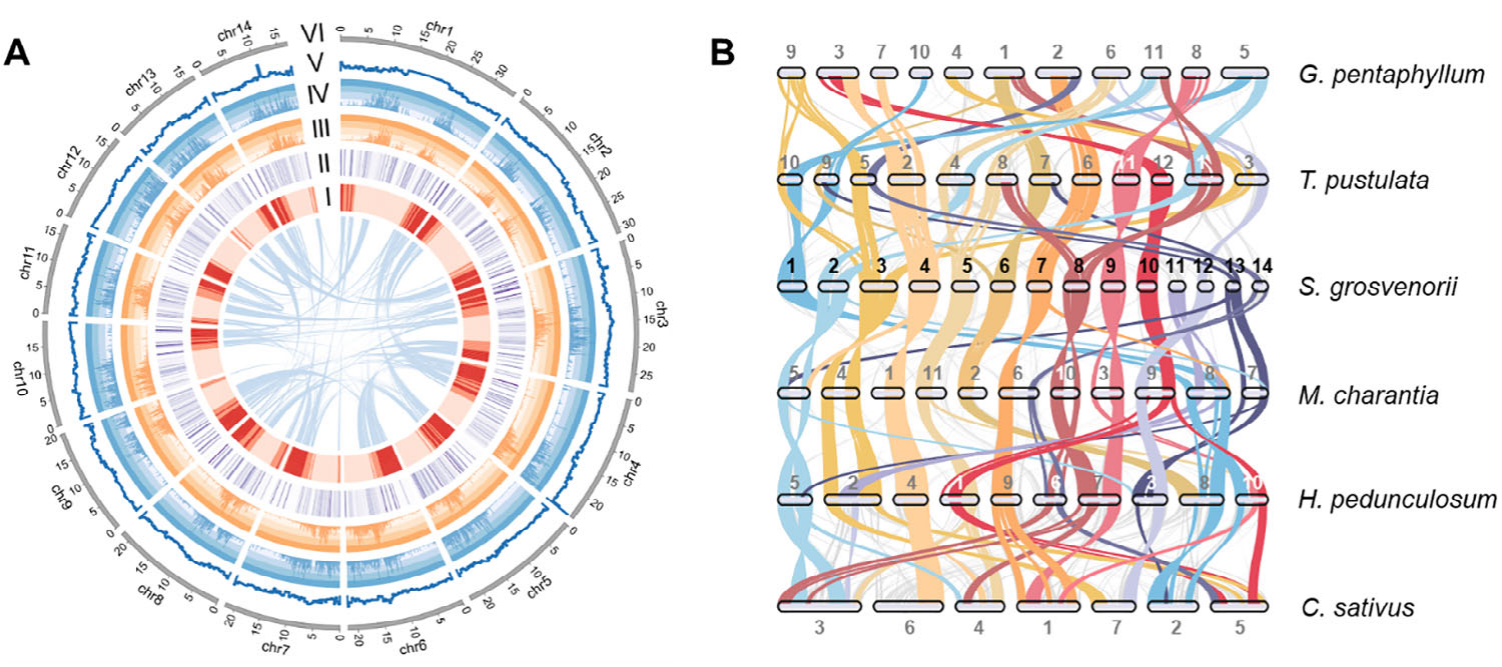
Genomic features of *S. grosvenorii* and collinearity analysis of Cucurbitaceae plants. (A) Genomic characteristics of *S. grosvenorii* depicted in a circos plot showcasing gene density distribution (I), LAI content (II), Gypsy (III) and Copia (IV) LTR retrotransposon density, GC content within a 300‐kb window (V), and the karyotype of each chromosome (VI). The central region displays inter‐genomic syntenic blocks (>300 kb). (B) Collinearity analysis of six Cucurbitaceae species, covering clades I, III–V, and VII–VIII. The collinearity supported a close relationship of the *T. pustulata*‐*S. grosvenorii*‐*M. charantia* group.

We here present a chromosome‐level genome assembly of *S. grosvenorii* belonging to Clade IV. The genome size was estimated to be approximately 374 Mb with heterozygosity ratio 0.95 based on 19 *k*‐mer analysis (Figure S5A). The 27.60 Gb (∼87.4× coverage) HiFi reads were assembled using hifiasm, along with removal of redundant sequences by purge_dups, resulting in a draft assembly of 315.62 Mb (42 scaffolds, N50 length of 21.88 Mb) with a GC content of 33.64%. Finally, 313.95 Mb (99.47%) of the scaffolds were anchored onto 14 pseudochromosomes (*2n* = 28) by 151.4× coverage reads from Hi‐throughput chromatin conformation capture (Hi‐C) library (Figure S5B) (Table 1). Integration of RNA‐seq‐assisted annotation, *de novo* prediction, and homologous protein search identified 21, 096 protein‐coding genes. Approximately 54.96% of the genome was annotated as transposable element (TE), and the vast majority (32.85%) were long terminal repeat (LTR)‐retrotransposons mainly composed of Gypsy (8.19%) and Copia (6.51%) elements (Figure 2A). Benchmarking universal single‐copy ortholog (BUSCO) assessment revealed 98.7% and 98.9% completeness for the genome assembly and annotations, respectively (Table 1 and Table S1). The LTR assembly index (LAI) score of the final assembly reached 12.91, indicating a reference‐level genome with the highest quality achieved in this species to date (Table S1) [22, 28].

**TABLE 1:** Summary of six Cucurbitaceae genome assemblies.

	*S. grosvenorii*	*G. pentaphyllum*	*T. pustulata*	*M. charantia*	*C. moschata*	*C. sativus*
(Pseudo)chromosomes	14	11	12	11	20	7
Assembly size [Mb]	315.6	582.9	220.5	303.0	269.9	226.2
Total scaffolds	42	578	80, 810	193	3, 500	85
Scaffold N50 [Mb]	21.8	50.8	7.9	25.3	4.0	31.1
Longest scaffold [Mb]	34.4	/	/	/	/	40.9
Total contigs	/	1, 232	86, 626	221	14, 432	174
Contig N50 [Mb]	/	1.7	0.017	9.8	0.048	8.9
GC content [%]	33.6	33.0	39.0	35.5	36.5	32.8
Protein	21, 096	25, 282	19, 847	41, 016	32, 205	24, 317
BUSCO [%]	98.7	94.9	96.5	96.4	97.3	/
Reference	this study	[29]	ASM4400753v1	[30]	[31]	[32]

The obtained *S. grosvenorii* genomes facilitated macrosynteny analysis within Cucurbitaceae. We selected six representative members from the major clades of cucurbits, including *G. pentaphyllum* (Clade I), *Thladiantha pustulata* (Clade III), *S. grosvenorii* (Clade VI), *Momordica charantia* (Clade V), *Herpetospermum pedunculosum* (Clade VII), and *C. sativus* (Clade VIII). Due to the absence of annotation information of *T. pustulata*, we further conducted *de novo* and homologous gene prediction of the reported *T. pustulata* genome (complete BUSCO of 91.6%) (Table S2). Regardless of diverse chromosomes numbers ranging from 7 to 14, the aligned genomes show an overall high degree of collinearity across cucurbit taxa (Figure 2B). In particular, the genome synteny of *T. pustulata*‐*S. grosvenorii*‐*M. charantia* group supported their close evolutionary relationship [8].

### 2.2 Versatile UGT73 Glycosyltransferases from *S. grosvenorii* Facilitate Mogrosides Biosynthesis

**FIGURE 3:**
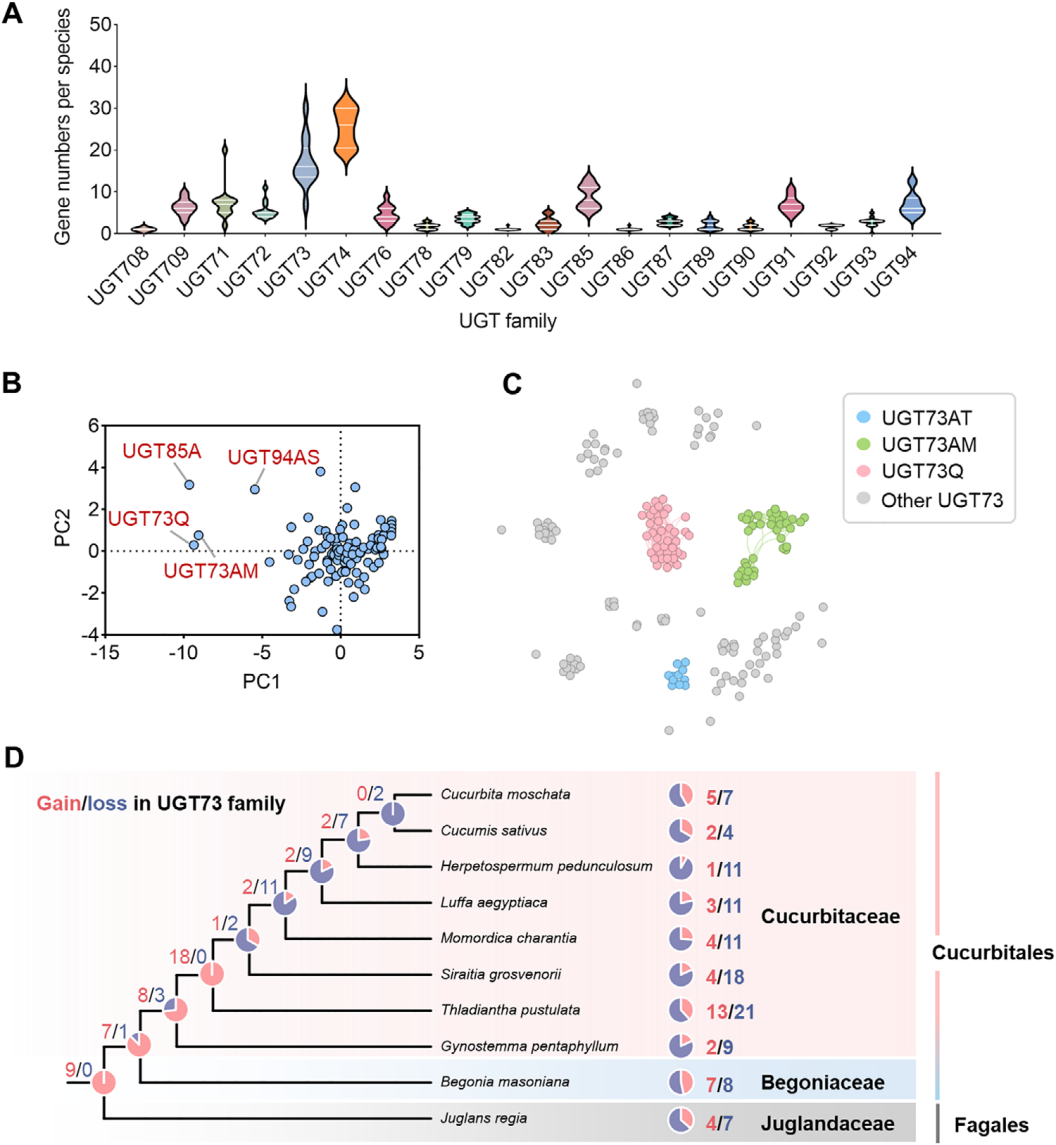
Analysis of UGT glycosyltransferases in Cucurbitaceae species. (A) A violin plot illustrates the distribution of different UGT family members in each Cucurbitaceae species. The width of each violin indicates the data density. The three horizontal lines embedded within each violin mark the first quartile (bottom), median (center), and third quartile (top). (B) PCA analysis highlights four orthogroups, UGT85A (OG00), UGT73Q (OG01), UGT73AM (OG02), and UGT94AS (OG03) significantly deviate from the others. Varying copies across different species may indicate their potential involvement in specialized metabolic pathways. (C) Sequence similarity network (SSN) analysis of 226 cucurbit UGT73 proteins reveals UGT73AM and UGT73Q as the predominant subfamilies of UGT73 glycosyltransferases. (D) Expansion and contraction of the UGT73 family in Cucurbitales and Fagales.

Triterpenoid saponins derived from basal Cucurbitaceae plants exhibited highly diverse glycosylation patterns, which prompted us to explore the driving forces behind the diversity of these saponins. Systematic mining of 13 chromosome‐level Cucurbitaceae genome assemblies gave a total of 1348 UGT‐encoding candidates that could be grouped into 26 UGT families, among which the largest family consisted of UGT74s, followed by UGT73 family (Figure 3A and Table S3). To further understand the lineage‐specific UGT distribution in Cucurbitaceae family, we performed orthologue identification followed by principal components analysis (PCA) of the orthogroup abundance across species (Figure 3B and Table S4). The cucurbit UGTs could be further divided into 113 UGT orthogroups, among which four UGT subfamilies (UGT73AM, UGT73Q, UGT85A, and UGT94AS) showed significant deviation from the others. We noticed that the previously characterized UGT94‐289‐3 from *S. grosvenorii* and GpUGT94AS1 from *G. pentaphyllum* fell precisely within the UGT94AS orthogroup, which therefore strongly implied the potential of other groups participating in similar saponin biosynthesis.

Consequently, we investigated the UGT85A and two UGT73 subfamilies. Sequence similarity network (SSN) analysis of 202 UGT73 proteins revealed that UGT73AM and UGT73Q were the predominant groups within Cucurbitaceae family (Figure 3C). Tracing the gain and loss of UGT73 genes after the divergence of Cucurbitales and Fagales, we observed a significant expansion at the ancestral node of Cucurbitales and Cucurbitaceae, until the speciation of *T. pustulata* and *S. grosvenorii* (Figure 3D). However, all Cucurbitaceae genera exhibited a contraction of the UGT73s, which underpinned the expansion of UGT73AM and UGT73Q as the major UGT73 glycosyltransferases in cucurbits (Figure S6). On the other hand, UGT85A subfamily is widely distributed in plants and plays important roles in both primary and specialized metabolisms. For instance, the functionally diverse UGT85As in tea (*Camellia sinensis*) affect the plant tolerance to abiotic stresses [33], whereas UGT85A191 in *Cistanche tubulosa* is pivotal in the biosynthesis of salidroside [34].

**FIGURE 4:**
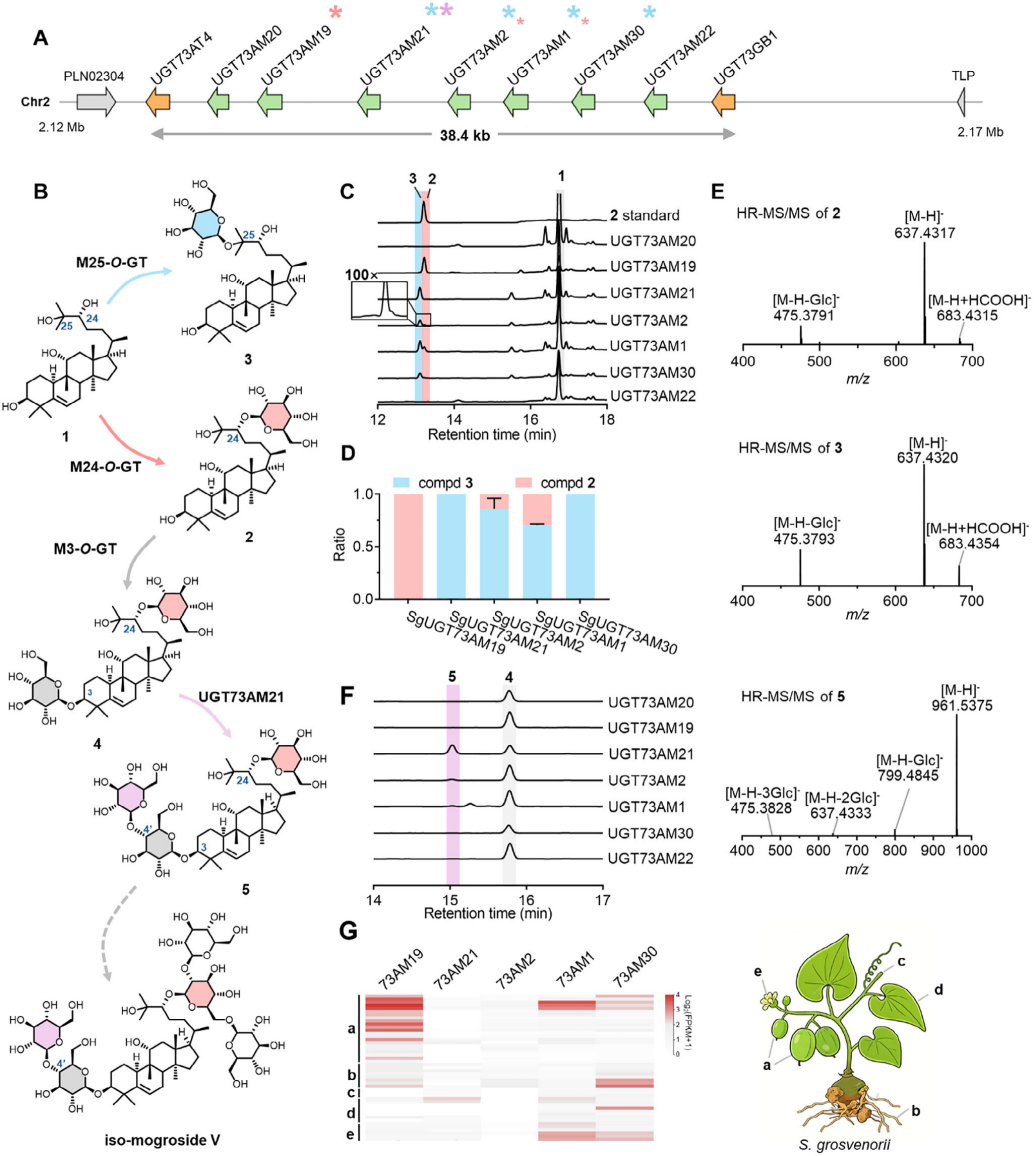
Tandem duplication‐driven neofunctionalized UGT73AMs involved in mogrosides biosynthesis. (A) Tandem array of *UGT73* genes on chromosome 2 (approximately 38.4 kb) of *S. grosvenorii*, flanked by a PLN02304 family protein and a thaumatin‐like protein (TLP). The asterisk denotes the glycosylation activity. Salmon, light blue, and light purple colors indicate mogrol 24‐*O*‐glucosylation, mogrol 25‐*O*‐glucosylation, and β‐(1→4)‐glucosylation activities, respectively. The size of the asterisk is proportional to the glycosylation activity. (B) Elucidated biosynthetic pathway of mogrosides in this work. M3‐O‐GT, mogrol 3‐*O*‐glycosyltransferase; M24‐O‐GT, mogrol 24‐*O*‐glycosyltransferase; M25‐O‐GT, mogrol 25‐*O*‐glycosyltransferase. Solid arrows indicate identified glycosylation steps, and dashed arrows indicate yet unknown steps. (C) High‐performance liquid chromatography‐ultraviolet (HPLC‐UV) chromatograms depicting the in vitro assay of SgUGT73AMs toward mogrol (**1)**. (D) Ratio of products **2** and **3** produced by SgUGT73AMs. All assays were conducted in triplicate, and error bars represent standard deviation (SD). (E) High‐resolution MS/MS spectra of glycosylated products **2**, **3,** and **5**. (F) HPLC‐UV chromatograms illustrating the in vitro enzymatic assay of SgUGT73AMs using compound **4** as substrate. (G) Heatmap showing the gene expression levels of bioactive SgUGT73AMs across five different tissue types: **a**. fruits at 5, 10, 15, 20, 25, and 30 days after flowering (DAF); **b**. roots; **c**. stems; **d**. leaves; **e**. buds. Expression values are expressed as log_2_(FPKM + 1).

To investigate the real role of UGT73AM, UGT73Q, and UGT85A glycosyltransferases in saponin biosynthesis, we first chose *S. grosvenorii*, a representative of NCC saponin‐rich species in the basal Cucurbitaceae as a model species for functional analysis. After examination of our assembled genome, we found that seven SgUGT73AM‐encoding genes formed a tandem duplication that spans ca. 38.4 kb in length on pseudochromosome 2, flanked by *SgUGT73AT4* and *SgUGT73GB1* (Figure 4A), while interestingly on the pseudochromosome 3, five *SgUGT73Q* also presented in a tandem array (ca. 31.2 kb) downstream to two *SgUGT85A* genes (Figure S7A). We then cloned 16 *UGT* genes in these regions, expressed them in *Escherichia coli*, and incubated their crude enzymes with mogrol (**1**), the aglycone of mogrosides. Liquid chromatography‐tandem mass spectrometry (LC‐MS/MS) analysis showed that five novel UGT73AMs, but neither UGT73Q nor UGT85A were active on **1** (Figure 4B,C and Figure S7B,C). Among the active enzymes, SgUGT73AM19, SgUGT73AM21, and SgUGT73AM30 exhibited distinct regio‐selectivity towards different hydroxyls on the **1** skeleton. While SgUGT73AM19 exclusively converted **1** to product **2**, SgUGT73AM21 and SgUGT73AM30 generated **3** as their only product (Figure 4C,D). Product **2** was identified as mogroside IA1 (mogrol 24‐*O*‐glucoside) by match of the LC‐MS/MS results to the standard sample (Figure 4E), and the new peak **3** with *m/z* 683.4386 ([M‐H+HCOOH]^−^ of mogrol monoglucoside) was confirmed as mogrol 25‐*O*‐glucoside by targeted isolation followed by nuclear magnetic resonance (NMR) measurement (Figures S8–S11). The other two enzymes (SgUGT73AM1 and SgUGT73AM2, which exhibit 83%–88% amino acid identity to SgUGT73AM19) produced mixed glycosides of **2** and **3**, with **3** as the major product (Figure 4C,D). A previous study has reported that SgUGT73D5 (SgroChr8G139200.1) can also generate mixed **2** and **3** [22], however, we unfortunately failed to detect any glycosylated products of this enzyme in our experiments (Figure S12). Our results confirmed soluble expression of SgUGT73D5, yet no glycosylation activity toward mogrol was detected under our assay conditions. Therefore, we can only conclude that SgUGT73D5 may lack the glycosylation activity of **1** under our assay conditions. One plausible explanation is that the catalytic activity of SgUGT73D5 might be affected by different expression systems, which involve the expression plasmid construction, reaction mixture (pH, buffer), reaction temperature, etc.

In addition to the abovementioned **1**‐recognizing activities, we further noticed that SgUGT73AM21 unexpectedly converted a bidesmoside substrate, mogroside IIE (M2E, **4**), to a trisaccharide product **5** (Figure 4F). LC‐MS/MS analysis of the reaction mixture implied the addition of a glucose moiety on **4** ([M‐H]^−^ = *m/z* 961.5375) (Figure 4E), and NMR experiments concluded a β‐1, 4‐glycosidic linkage of the added glucosyl to the C3‐sugar of **4** (Figures S13–S16). A cross peak between H‐1*′′′*/C‐4*′* (δ_H_ 4.4/δ_C_ 80.9) in heteronuclear multiple bond correlation (HMBC) spectrum clearly supported the C3‐sugar chain elongation activity of SgUGT73AM21. Notably, such 1, 4‐glycosidic linkage accomplished by UGT has rarely been documented in plant secondary metabolisms. The first example of β‐1, 4‐glycosyltransferase was AsTG1 from oats (Avena species), which belongs to the glycoside hydrolase family 1 (GH1) enzymes and has been shown to form the β‐1, 4‐glycosidic bond in Avenacin A‐1 [4]. Until very recently, UGTs catalyzing 1, 4‐glycosidic linkages have been identified in saponin biosynthesis, including UGT91AQ1 from Quillaja saponaria and UGT79L3 from Saponaria officinalis [6, 35]. Furthermore, the formation of the β‐1, 4‐glycosidic bond in QA‐TriF(Q)RXX in soapbark is also catalyzed by a GH1 enzyme, SoGH1.

To further understand the substrate spectrum of SgUGT73AM21, we then tested its activities toward six key intermediates in the mogroside biosynthetic pathway (Figure S17). According to the LC‐MS/MS results, SgUGT73AM21 was found capable of converting mogroside III (**6**) and mogroside IIIE (**9**) to corresponding glucosylated products **7** ([M‐H]^−^ = *m/z* 1123.5903) and **10** ([M‐H]^−^ = *m/z* 1123.5898), respectively, but not active on mogroside IIIA2 (**8**), siamenoside I (**11**), mogroside IV (**12**), and mogroside IVA (**13**). These results suggested that SgUGT73AM21 could only recognize intermediates bearing disaccharide at the C24 and monosaccharide at the C3 of **1**, while any extra sugars on C3‐glucosyl or C24‐sophorosyl/gentiobiosyl probably created steric hindrance to abolish its glycosylation activities (Figure S17C). We therefore speculated that SgUGT73AM21 catalyzed the β‐1, 4‐glycosylation of C3‐sugar prior to the formation of C24‐trisaccharide chain in the biosynthesis of iso‐mogroside V (a rare mogroside in *S. grosvenorii*) (Figure 4B), which stands for an early branch point of *iso*‐mogroside V and mogroside V biosynthetic pathway. Moreover, we also individually tested other SgUGT73AMs towards **4**, but only weak activities of SgUGT73AM1 and SgUGT73AM2 were detected (Figure 4F).

As for *SgUGT73AT4* and *SgUGT73GB1* adjacent to the *SgUGT73AM*s, neither of them was detected active toward mogroside substrates (Figure S18), however, we found that SgUGT73AT4 could catalyze hederagenin (**14**), a common aglycone of pentacyclic terpenoids, to produce compound **15** with *m/z* 679.4029 ([M‐H+HCOOH]^−^). After sample preparation from scale‐up reaction, **15** was characterized as hederagenin‐28‐*O*‐glucoside by ^1^H‐NMR analysis, suggesting that SgUGT73AT4 was a hederagenin 28‐*O*‐glucosyltransferase (Figures S19 and S20). Unfortunately, the function of SgUGT73GB1 still remains unclear.

Mapping the transcriptomics data to the genomic region of *SgUGT73AM* tandem duplication revealed a differential expression pattern of five bioactive UGT73AM‐encoding genes across tissues (Figure 4G). SgUGT73AM19 was predominantly expressed in fruit, whereas SgUGT73AM30 and SgUGT73AM1 were highly expressed in both fruit and root, hinting at potential roles in mogroside and cucurbitacin glycosylation, respectively. These results identify multiple tandemly duplicated UGT73AM enzymes with distinct in vitro activities, suggesting functional divergence among paralogs, although definitive in vivo functional assignment still requires further genetic evidence.

### 2.3 UGT73AM Tandem Duplications Contribute to Diversity of Saponins in Cucurbits

**FIGURE 5:**
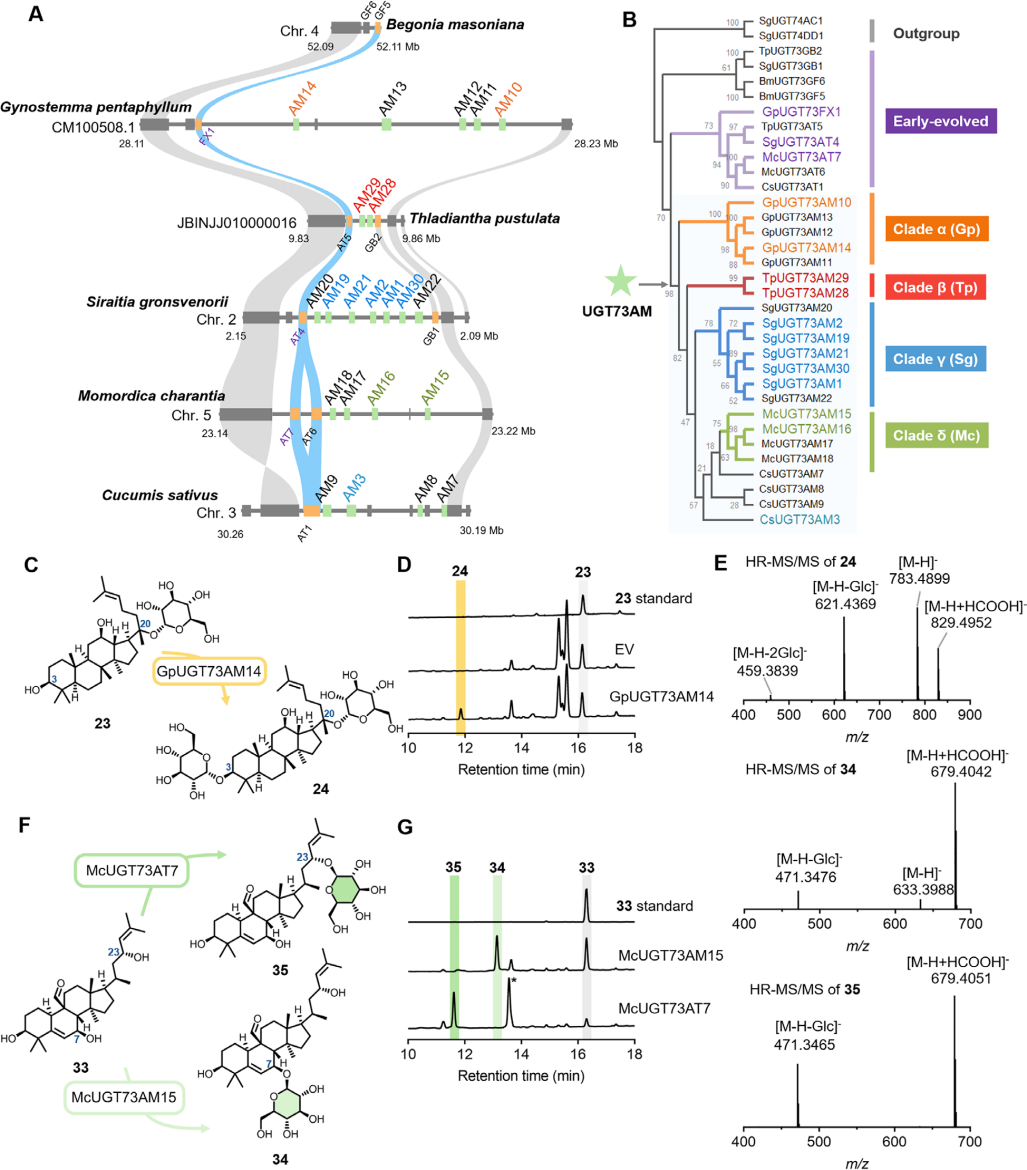
Comparative genomics and functional characterization of UGT73 tandem duplicates in Cucurbitaceae family. (A) Microsynteny analysis of *UGT73* tandem duplications in *B. masoniana* (Begoniaceae) and five Cucurbitaceae species (*G. pentaphyllum*, *T. pustulata*, *S. grosvenorii*, *M. charantia*, and *C. sativus*). (B) Phylogenetic tree of UGT73s involved in the UGT73 tandem duplications. (C) Characterization of GpUGT73AM14 through in vitro assays and (D) HPLC‐UV chromatograms of GpUGT73AM14‐mediated glycosylation. EV, empty vector. (E) High‐resolution MS/MS spectra of the glycosylated products **24**, **34,** and **35**. (F) Characterization of McUGT73AT7 and McUGT73AM15 through in vitro assays and (G) HPLC‐UV chromatograms of McUGT73 reaction mixtures. The asterisk (*) marks impurity.

The discovery of tandem duplicated *SgUGT73AM* facilitating mogrosides biosynthesis greatly encouraged us to explore more UGT73AM orthologues that might also participate in other saponin biogenesis within the Cucurbitaceae family. Through microsynteny analysis of five Cucurbitaceae species (*G. pentaphyllum*, *T. pustulata*, *S. grosvenorii*, *M. charantia*, and *C. sativus*) and *Begonia masoniana* (Begoniaceae family, as outgroup) (Figure 5A), a highly conserved genomic segment harboring *UGT73* tandem duplicates was found in various cucurbits. We therefore constructed a maximum likelihood (ML) tree of 32 involved UGT73s and two UGT74 enzymes to clarify the phylogeny of these glycosyltransferases (Figure 5B). According to the constructed tree, the emergence of UGT73AM after splitting from the early evolved clade (UGT73GB/AT/FX subfamilies) led to four monophyletic clades (α, β, γ and δ), each corresponding to its respective source species (Figure 5B) in the basal Cucurbitaceae (implying their UGT73AM duplicates emerged after speciation), while the *C. sativus* UGT73AMs appeared to be a paraphyletic group probably caused by insufficient sampling of UGT73s from the lately evolved cucurbits.

Clade α consisted of GpUGT73AM10∼14 solely from *G. pentaphyllum* (Figure 5B). Given that the characteristic metabolites of *G. pentaphyllum* are DM‐derived ginsenoside saponins, we proposed a potential role of the GpUGT73AMs active in the protopanaxadiol (PPD, **18**) and protopanaxatriol (PPT, **19**)‐type ginsenoside pathways. Although none of these GpUGT73AMs showed glycosylation activity on PPD (**18**), we detected a new peak of **20** ([M‐H+HCOOH]^−^ = *m/z* 679.4029) produced by incubating GpUGT73AM10 with PPT (**19**) (Figure S21). The product was neither ginsenoside F1 (**21**) nor ginsenoside Rh1 (**22**), two well‐known PPT‐type monoglucosides, while NMR measurement confirmed **20** as PPT 3‐*O*‐glucoside due to the key HMBC correlation of H‐1*′*/C3 (δ_H_ 5.02/δ_C_ 89.9) (Figures S22–S24). Subsequently, GpUGT73AM14 was identified as a versatile enzyme that glycosylated both PPD‐type ginsenoside CK (**23**) and PPT‐type **21**, **22** to corresponding glycosylated products **24, 25,** and **26**, respectively (Figure 5C; Figures S25 and S26). The PPD‐type product **24** and **25** were purified and assigned by NMR as ginsenoside F2 and ginsenoside F1 3‐*O*‐glucoside, respectively (Figures S28–S32). Although the low abundance of **26** hampered the NMR verification, it was considered as ginsenoside Rh1 3‐*O*‐glucoside because its retention time was inconsistent with ginsenoside Rg1 (**27**) and Rf (**28**) that possess 6‐*O*‐glucosyl or 20‐*O*‐ glucosyl groups (Figure S25). Also inspired by the sugar chain elongation activity of SgUGT73AM21, we further questioned if these GpUGT73AM glycosyltransferases could perform glycosylation on the sugar moiety of PPD/PPT monoglucosides (**21**‐**23**, **29**). Interestingly, none of the GpUGT73AMs exhibited activity against **22**, whereas GpUGT73FX1 co‐located in tandem duplicated UGT73 region, did, producing a putative diglucoside product **30** (Figures S25–S27). Nevertheless, the amount of **30** was too low to be fully elucidated by NMR analysis.

Syntenic to the GpUGT73AM/SgUGT73AM‐encoding region, *T. pustulata UGT73*s duplication harbors a *UGT73AT*, a *UGT73GB* and two *UGT73AM* copies (Clade β). TpUGT73AM28 and TpUGT73AM29 both showed trace glycosylation activities towards quillaic acid (QA, **31**), the aglycone of dubioside sapogenin in *T. pustulata*, leading to product **32** with *m/z* 647.3768, which was tentatively identified as QA‐monoglucoside (Figure S33). On the other hand, the *M. charantia UGT73* duplication has evolved four *UGT73AM* and two *UGT73AT* copies (Clade δ) (Figure 5A,B). We identified McUGT73AM15 by in vitro enzymatic assay, in which momordicine I [MomI (**33**), a bitter‐tasting aglycone in *M. charantia*] was glycosylated to product **34** with *m/z* 679.4042 ([M‐H+HCOOH]^−^ of momordicine I monoglucoside) (Figure 5D). According to the 2D‐NMR analysis, HMBC correlation of H‐1*′*/C‐7 (δ_H_ 4.24/δ_C_ 73.4) supported compound **34** to be MomI‐7‐*O*‐glucoside (formal name: momordicine IV) (Figure 5D; Figures S34–S36). Additionally, McUGT73AT7 could catalyze momordicine I to produce a new compound **35** with *m/z* = 679.4051. Large scale purification followed by NMR measurements confirmed that **35** was MomI‐23‐*O*‐glucoside (formal name: momordicine II) based on the key HMBC correlation of H‐1*′*/C23 (δ_H_ 4.27/δ_C_ 76.7) (Figures S37–S39). These findings clearly demonstrated that the *M. charantia UGT73* duplication contributed to the biosynthesis of anti‐diabetic saponins in bitter melon.

Cucumber (a lately evolved gourd) also possesses a *UGT73AM* tandem duplication (Figure 5A). We noted that a previously characterized enzyme, CsUGT73AM3 located within this duplication, functioned as a cucurbitacin C 3‐*O*‐glucosyltransferase [18]. In this study, we further elucidated that it could also glucosylate both **1** and cucurbitacin IIb (CuIIb, **36**) at the C25 position (Figures S40–S45), which expanded the substrate spectrum of this enzyme.

Positive selection analysis revealed that the Cucurbitaceae UGT73AM branch underwent positive selection with a value for *p* = 0.017 (Figure S46). Bayesian analysis estimated the divergence between *UGT73AT/FX*s and the specialized *UGT73AM*s occurred approximately 62 ± 9 million years ago (MYA) (Figure S47), which coincided with the emergence of the Cucurbitaceae family [8]. Successively, clade α (*G. pentaphyllum*), clade β (*T. pustulata*), and clade γ (*S. grosvenorii*) appeared in the respective genera about 48, 37, and 34 MYA. Unlike the early‐evolved *UGT73AT/FX* clade diverged continuously from Eocene to Miocene (55–10 MYA), the specialized *UGT73AM*s in each species experienced a relatively late expansion during Miocene (23–5 MYA) (Figure S47). For the *S. grosvenorii*‐specific clade γ, we proposed a three‐step evolutionary trajectory of seven *UGT73AM* copies (Figure S47A). The ancestors of SgUGT73AM20 and SgUGT73AM22 diverged first, remaining inactive to **1** (but unknown to other substrates), while the common ancestor of the rest underwent neofunctionalization, acquiring C24‐*O*‐ and C25‐*O*‐glycosylation abilities (Figure 4 and Figure S47). We suspected that the glycosylation products of the original ancestral enzymes were likely a mixture of C24/C25‐*O*‐glucosides (such as those of SgUGT73AM1 and SgUGT73AM2), but subsequently these *SgUGT73AM*s evolved to copies with regiospecific substrate recognition (SgUGT73AM19 specialized for producing **2**, while SgUGT73AM21/ SgUGT73AM30 for producing **3**) (Figure 4C,D). This could be an interesting example of how tailoring enzymes underwent initial neofunctionalization followed by sub‐functionalization during evolution. On the other hand, neofunctionalizations constantly emerged in SgUGT73AMs, as evidenced by the rarer capability of 1, 4‐glycosidic bond formation in addition to C25‐*O*‐glycosylation that SgUGT73AM21 has lately evolved (Figure 4F). Through such sophisticated evolution, *S. grosvenorii* had developed the capability of not only producing major mogrosides via 24‐*O*‐glycosylation, but also the minor mogrosides like iso‐mogroside V, indicating that the diversification of UGTs plays a key role in shaping the metabolic diversity.

Among other Cucurbitaceae UGT73AMs, GpUGT73AMs in clade α underwent thrice gene duplication events. The earliest diverged GpUGT73AM10 could only glycosylate the aglycone **19**, while the more recently diverged GpUGT73AM14 had a wider substrate spectrum (Figure 5C; Figures S25 and S26). Similarly, the earliest diverged McUGT73AM15 (Clade δ) in *M. charantia* could accept the aglycone MomI, but McUGT73AM16∼18 might have other specific substrates that remain unknown. (Figure 5F and Figure S47). Notably, the *McUGT73AT*s also experienced duplication to give McUGT73AT7 as MomI 23‐*O*‐glycosyltransferase (Figure 5F), which suggested additional duplication events other than UGT73AM's could also diversify saponin metabolism in bitter melon. The clade β (*T. pustulata*) suffered only one duplication event, generating 2 copies of *UGT73AM*s with slight activity, which is consistent with the poor saponins diversity of *T. pustulata*. These results suggested that the lineage‐specific tandem duplication of UGT73s provided a basis for functional innovation, through which neofunctionalized UGTs respectively contribute to the diversity of saponins in Cucurbitaceae family.

### 2.4 A Conserved UGT73‐Containing Syntenic Region Repeatedly Facilitated Triterpenoid Glycosylation in Core Eudicots

**FIGURE 6:**
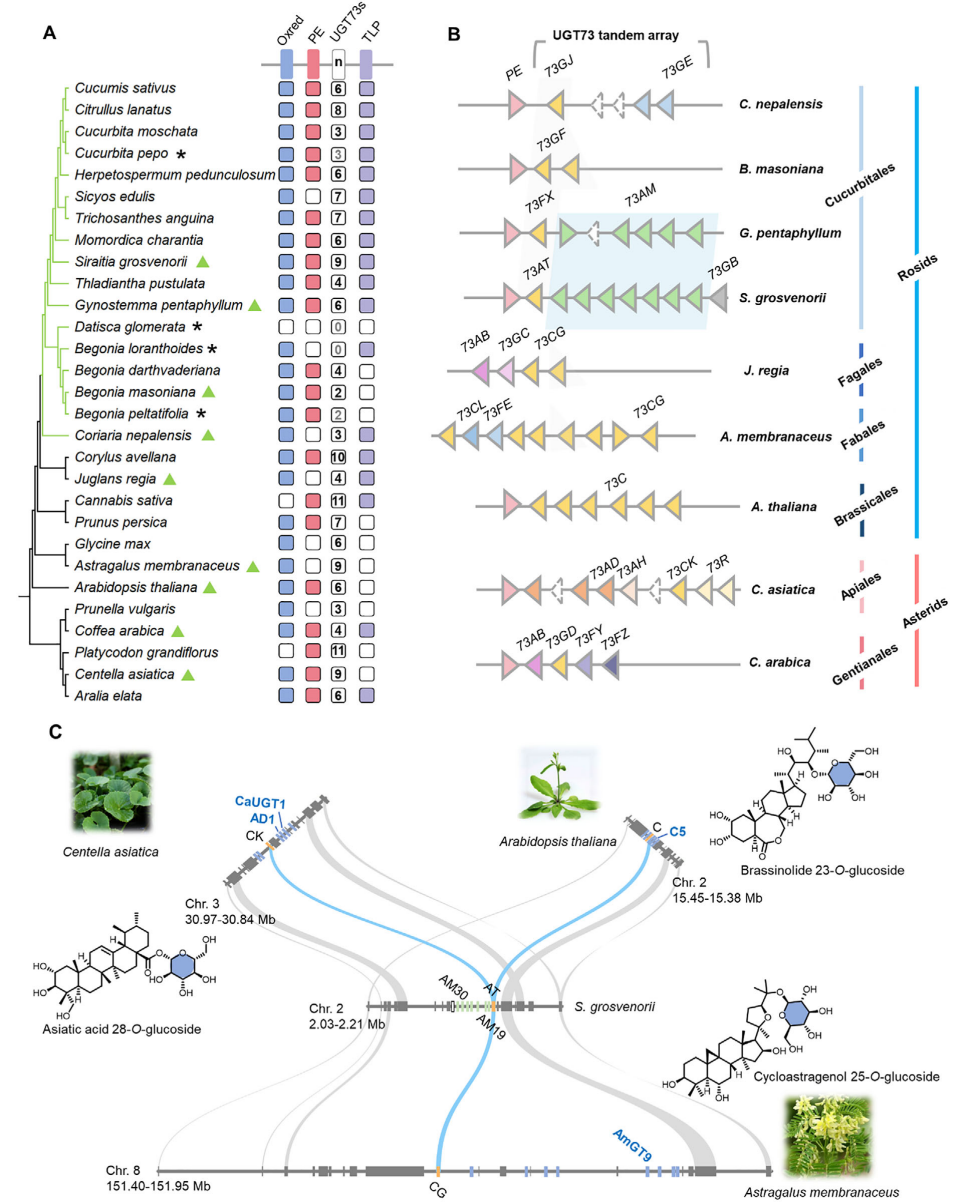
Phylogenetic and comparative genomic analysis of UGT73 tandem arrays in core eudicots. (A) “UGT73 tandem array” in core eudicots. Numbers in the squares of UGT indicate the number of UGT73 copies. Conserved genes flanking UGT73 tandem array are oxidoreductase (Oxred), pectinesterase (PE), and thaumatin_like proteins (TLP). A detailed version is shown in Figure S48. Green branches represent Cucurbitales species, including the Cucurbitaceae, Begoniaceae, Datiscaceae, and Coriariaceae family plants. Asterisks denote species with lower‐quality genome assemblies. Green triangles mark selected high‐quality genomes; their corresponding UGT73 tandem array regions are detailed in panel B. (B) Genomic organization of “UGT73 tandem array” in representative species of rosids and asterids. (C) Microsynteny analysis of UGT73 tandem arrays in *Siraitia grosvenorii* (mogrosides producer) and three other saponin‐rich species (*Centella asiatica*, *Arabidopsis thaliana*, and *Astragalus membranaceus*). UGT73 enzymes previously validated are labeled in blue font. The sky‐blue ribbons indicate SgUGT73AT4 orthologs, and purple squares represent other UGT73s.

The UGT73 family members had also been shown to glycosylate various types of saponin in many angiosperm plants [36]. Consequently, we were interested in the possibility of a wider distribution of saponin‐producing UGT73 tandem duplications. To determine whether the newly identified UGT73 duplications are unique to Cucurbitaceae, we first searched the genomes of 17 species within the Cucurbitales order (11 from Cucurbitaceae family, 4 from Begoniaceae family, 1 from Coriariaceae family, and 1 from Datiscaceae family). Comparative genomic analyses by JCVI [37] captured a syntenic block harboring genes encoding “oxidoreductase (Oxred)‐pectinesterase (PE)‐UGT73 glycosyltransferase‐thaumatin_like proteins (TLP)” (Figure 6A). We designated the UGT73 duplicates structure in this conserved genomic region the “UGT73 tandem array”. Other gene loci encoding methyltransferase (MT), chromosome segregation protein (SMC), phosphatidylinositol phosphate kinase (PIPK), and major intrinsic protein (MIP) could also be found in this region, varying among different species (Figure S48).

In the Cucurbitales “UGT73 tandem array”, the first one or two *UGT* copies immediately downstream to *PE* belong to the UGT73AT/GF/GJ subfamily, followed by proximal duplication typically comprising another 2–7 *UGT* copies (Figure 6B). Phylogeny analysis demonstrated that the *UGT73AT/FX‐UGT73AM* combination was Cucurbitaceae‐specific, while in *Coriaria nepalensis* (Coriariaceae family) this UGT73 duplication consisted of *UGT73GJ*‐*UGT73GE* (Figure S49). *Begonia masoniana* (Begoniaceae family), however, held a tandem duplication uniformly composed of *UGT73GF*.

Next, we speculated a more universal distribution of UGT73 tandem arrays beyond Cucurbitales. After examination of the closely related Fagales and Fabales order using *PE*‐*UGT73*s‐*TLP* as a syntenic anchor, we observed a ubiquitous existence of the “UGT73 tandem array” counterparts in a variety of species, including *Juglans regia* (Juglandaceae family), *Corylus avenella* (Betulaceae family), *Glycine max* (Fabaceae family), and *Astragalus membranaceus* (Fabaceae family) (Figure 6A,B). If expanded to the entire rosids plants, corresponding *UGT73* tandem duplications could also be found in species within the Rosales (i.e., *Prunus persica*, Rosaceae family/*Cannabis sativa*, Cannabaceae family) and Brassicales orders (i.e., *Arabidopsis thaliana*, Brassicaceae family). The *Platycodon grandiflorus* UGT73 and *C. sativa* UGT73 tandem array both encompass 11 copies, the highest number recorded in this region (Figure 6A). Unlike those in the Cucurbitales, the homologous UGT73 array in *G. max* and *A. thaliana* uniformly evolved to be a single subfamily (UGT73CG in *G. max* and UGT73C in *A. thaliana*). Although most of the *UGT73* duplicates found here have not been characterized, we noticed several examples that contributed to the triterpenoid biosynthesis in Fabaceae and Brassicaceae family. One is AmGT9 from *A. membranaceus* (Fabaceae) that modifies the side chain of cycloastragenol (CA), being characterized as CA‐25‐*O*‐glycosyltransferase [38] (Figure 6C). Another case is that AtUGT73C5 and AtUGT73C6 in the tandem duplicated UGT73Cs of *A. thaliana* catalyze the C23‐*O*‐glucosylation of brassinolide [39]. Corresponding UGT73Cs were reported to be responsible for the biosynthesis of hederagenin and oleanolic acid cellobioside, which render *B. vulgaris* (a Brassicaceae family plant) resistant to insect pests [40, 41].

Functional “UGT73 tandem array” could also be seen in asterids. We found that both UGT73AD1 and CaUGT1 (renamed as UGT73AD3) located in the corresponding region on *Centella asiatica* chromosome 3 (Apiaceae family) have been identified to add glucose moiety to asiatic acid at C28 position, generating asiatic acid‐28‐*O*‐glucoside in asiaticosides biosynthetic pathway [42, 43]. Likewise, UGT73AD2 in the pseudochromosome 9 of *A. elata* (Araliaceae family), could convert echinocystic acid to echinocystic acid‐28‐*O*‐glucoside [24]. Recently reported Pvul_UGT73‐2 capable of glycosylating oleanolic acid, also resided in the corresponding region of *Prunella vulgaris* chromosome 1 (Lamiaceae family) [44]. Similarly, PgGT2 from *Platycodon grandiflorum* (Campanulaceae) found in the syntenic region specifically converts platycodigenin to platycodigenin‐3‐*O*‐glucoside, the key intermediates of platycodins [45]. Surprisingly, it has been reported that PgUGT72 (another UGT73 adjacent to PgGT2 on *P. grandiflorum* genome) lacks the same glycosylation activity [46].

Although the *UGT73* tandem array was universally detected in core eudicots, we could not find its counterpart in the ANA grade (Amborellales‐Nymphaeales‐Austrobaileyales) of the basal eudicots, nor in magnoliids, monocots, and gymnosperm, suggesting its late origin specific to the ancestor of core eudicots plants. In summary, within this lineage‐specific genomic architecture, the expanded and diverged UGT73 copies may have recurrently served as a source of functional innovation, potentially facilitating the biosynthesis of diverse triterpenoid glycosides (saponins) and contributing to the distinct metabolic phenotypes observed across eudicot plants.

### 2.5 The α4 Helix is Crucial for Regio‐Specificity of SgUGT73AM30

To understand the molecular mechanism underlying regio‐specific glycosylation of the discovered UGT73s, we chose SgUGT73AM30 from *S. grosvenorii* as a representative for further investigation due to its strict 25‐*O*‐glycosylation activity toward mogrol (**1**). The optimal temperature, pH, and metal ions for the enzymatic assay of SgUGT73AM30 were first determined (Figure S50). Kinetic analysis revealed that SgUGT73AM30 showed higher *K*_m_ (38.72 µM) and *V*_max_ (76.06 nmol·min^−^^1^·mg^−^^1^) for **1**, while SgUGT73AM19 possessed higher affinity (lower *K*_m_ of 26.99 µM) but moderately *V*_max_ (62.10 nmol·min^−^^1^·mg^−^^1^). The activity of SgUGT73AM19 was comparable to the previously reported mogrol 24‐*O*‐glycosyltransferase, SgUGT74DD1. (Figure S51). To thoroughly clarify the substrate specificity of SgUGT73AM30, a substrate library consisting of 15 triterpenoids (10 aglycones and 5 mogrosides) was screened, showing that SgUGT73AM30 could only catalyze the mono‐glycosylation of five substrates (**1**, **36**, **38**, **39**, **43**) (Figure S52). Despite structural variations, a key commonality across these substrates is that neither the C24 nor the C25 hydroxyl group is glycosylated. NMR characterization definitively identified the glycosylation site as the C25 position, yielding C25‐*O*‐glucosides in each case (Figure S53). For example, SgUGT73AM30 is able to glycosylate compound CuIIb **(36)**, resulting in the formation of CuIIb 25‐*O*‐glucoside (Figures S41–S45). For mogrosides substrates, SgUGT73AM30 could glycosylate **38** and **39** at the C‐25 hydroxyl groups, generating products **45** and **46**, respectively. Product **46**, identified through NMR analysis, was mogroside IIA2 25‐*O*‐glucoside (Figures S54–S58). Although sufficient pure **45** could not be obtained for NMR elucidation, it was presumed to be mogroside IIB. Additionally, SgUGT73AM30 displayed the ability to catalyze cycloastragenol (CA, **43**) to CA 25‐*O*‐glucoside (**44**) (Figures S59 and S60f).

**FIGURE 7:**
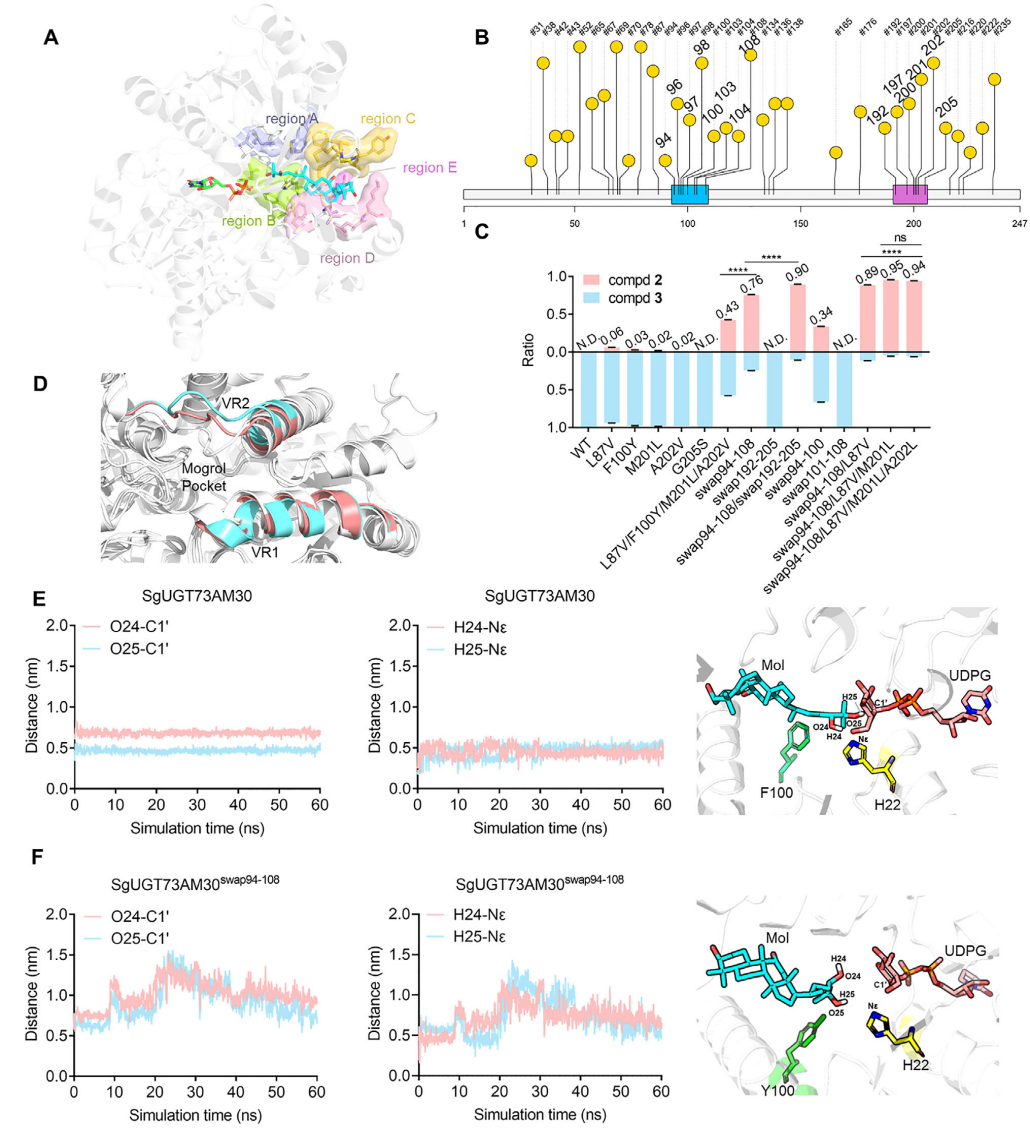
Structural insights into the regioselectivity mechanism of SgUGT73AM30. (A) Overall structure of the SgUGT73AM30 complex with mogrol and UDP. (B) Sequence alignment of SgUGT73AM30 and SgUGT73AM19 highlighting two highly variable regions. (C) Regioselectivity of SgUGT73AM30 mutants (*n* = 3 biological replicates). ****, *P* < 0.0001; ns, no significant difference, (Two‐tailed Student's *t*‐test). N.D., not detected. (D) Structural alignment of variable regions 1 (VR1) and 2 (VR2) in SgUGT73AM30 (white), SgUGT73AM19 (salmon), and SgUGT73AM30^swap94‐108^ (cyan). The 60 ns Molecular Dynamics (MD) simulations of I SgUGT73AM30‐Mogrol‐UDPG complex and (F) SgUGT73AM30^swap94‐108^‐Mogrol‐UDPG complex.

To further understand how SgUGT73AM30 accommodates different substrates but regio‐selectively glycosylates the C25‐hydroxyl, we solved the complex crystal structure of SgUGT73AM30/mogrol/UDP at 2.6 Å (PDB ID: 26FX, Figure 7A). SgUGT73AM30 possessed a typical GT‐B fold of plant UGT structures, i.e., two Rossmann‐fold (β/α/β) domains connected by a linker loop separating the N‐terminal domain (NTD, residues 1–247) and C‐terminal domain (CTD, residues 248–496) for respective binding of sugar receptor and donor. The NTD contained 10 α‐helices and 9 β‐sheets (α1‐10 and β1‐9), whereas the CTD contained 12 α‐helices and 6 β‐sheets (α11‐22 and β10‐15) (Figure S61). In the resolved structure, triterpenoid binding pocket around mogrol in 5 Å was formed by a series of hydrophobic residues, which could be grouped into 5 regions [region A (A19/L87/F100/C129), region B (L294/F392/A393/W420), region C (M201/A202/F204/G205/Y206/M208), region D (F154/M158/L161/K162) and region E (K195/S196)] (Figure 7A). A proper orientation of the mogrol C25 hydroxyl toward the diphosphate group of UDP well explained the C25‐*O*‐selectivity of SgUGT73AM30 (Figure S62A). Furthermore, substitutions of His22 and Asp127 with alanine completely abolished the glycosylation activity, indicating once again their key roles in substrate deprotonation (Figure S62B,C).

SgUGT73AM19, which shares 85.7% amino acid identity with SgUGT73AM30 and is located in the same tandem duplication cluster, yet surprisingly exhibited a nearly complete C24‐O‐glycosylation activity. It therefore served as a perfect control for studying the structural differences influencing their regio‐selectivity. Comparison of the AlphaFold‐predicted SgUGT73AM19 structure with the SgUGT73AM30 crystal structure revealed a root mean square deviation (RMSD) of only 0.604 Å (Figure S63), highlighting that subtle structural differences between two enzymes have a significant impact on their glycosylation products. We then aligned the amino acid sequences of SgUGT73AM19 and SgUGT73AM30, and noticed five different residues (L87, F100, M201, A202, and G205 in SgUGT73AM30) in the mogrol‐binding pocket (Figure 7B). Unfortunately, single mutagenesis of the former four sites could just slightly alter its regio‐selectivity, while mutation of G205 to Ser failed to show any change (Figure 7C). Although the quadruple mutant SgUGT73AM30^L87V/F100Y/M201L/A202V^ could shift the C25‐*O*‐/C24‐*O*‐glycosylation ratio to 57.4% vs. 42.6% (Figure 7C), it was still far from achieving the strict selectivity of SgUGT73AM19, suggesting a more sophisticated substrate‐recognizing mechanism remains uncovered.

We next scrutinized the NTD sequences of SgUGT73AM19 and SgUGT73AM30 and figured out two highly variable regions VR1 (residues 94–108) and VR2 (residues 192–205) (Figure 7B). Grafting VR1 and VR2 of SgUGT73AM19 onto that of SgUGT73AM30 gave two chimeric enzymes (designated SgUGT73AM30^swap94‐108^ and SgUGT73AM30^swap192‐205^) with the regio‐selectivity of the former significantly altered, while the latter hardly changed (Figure 7C). Likewise, the chimeric SgUGT73AM19^swap94‐108^ created by swap the VR1 region with SgUGT73AM30 had a C25‐*O*‐/C24‐*O*‐glycosylation activity altered to 33.3%/66.7% (Figure S64). These results indicated VR1 located on α4 helix was crucial for regio‐selectivity of these enzymes. Further splitting of the SgUGT73AM30 VR1 region into VR1N (94‐100) and VR1C (101‐108) and their swapping to SgUGT73AM19 failed to achieve the effect of the chimeric SgUGT73AM19^swap94‐108^, which demonstrated that the whole motif determined the regio‐selectivity of SgUGT73AM30 (Figure 7C). Finally, combining VR1 and L87V/F100Y/M201L/A202V mutations could significantly increase the proportion of the C24‐*O*‐glycosylation product. The most excellent mutant SgUGT73AM30^swap94‐108/L87V/M201L^ exhibited a high regio‐selectivity of 95.4% C24‐*O*‐glycosylation over 4.6% C25‐*O*‐glycosylation, which was comparable to the naturally evolved SgUGT73AM19 (Figure 7C). Upon comparison of these structures, we considered that the SgUGT73AM30 variants exhibited slight conformational changes in the VR1 region and generated a subtle outward shift, thereby aligning more closely with the conformation of wildtype SgUGT73AM19 (Figure 7D).

To further explain the regioselectivity mechanism of VR1 region, we performed a 60 ns molecular dynamics (MD) simulation to compare the SgUGT73AM30 /SgUGT73AM30^swap94‐108^‐UDPG‐compound **1** ternary complex structures, denoted as model‐A and model‐B, respectively. In these models, the plot of RMSD values ultimately reached a relatively stable plateau, indicating a steady state of structure was attained (Figure S65). The terminus hydroxyl in the side chain of **1** had significantly different orientations in the two models (Figure 7E,F). During the simulation, the distance between the oxygen atom of C25‐hydroxyl and the anomeric carbon C1’ of UDP‐sugar [d(O25‐C1’)] was significantly shorter than the d(O24‐C1’) (the oxygen of C24‐hydroxyl to UDP‐sugar) in model A, while these distances were much undistinguishable in model B, supporting the enzymatic assay results. Furthermore, the distances between the Nε atom of His22 in the catalytic dyad and the H atom of the terminal hydroxyl group (H24 and H25) did not differ significantly in the two models, suggesting that it is likely not the decisive factor for regioselectivity. The conformational changes in the VR1 region led to a larger substrate binding pocket, and the F100Y mutation also reduces the hydrophobic interactions experienced by the **1** side chain, resulting in a more flexible side chain conformation which ultimately affects the regioselectivity of glycosylation reaction. The Root Mean Square Fluctuation (R.M.S.F.) values also indicated that VR1 exhibited higher flexibility (Figure S66). The above results indicated that subtle changes in the VR1 region are crucial for the regioselectivity of SgUGT73AM30.

Previous studies on SgUGT94‐289‐3 identified a single residue, H96 located in the Nα4 helix (corresponding to VR1 of SgUGT73AM30), which was important to orient glucose moiety of mogroside III or siamenoside I for β(1‐6) glucosylation [47]. We have also tried to determine whether a specific residue in SgUGT73AM30 could be responsible for regioselectivity, but the abovementioned results suggested that conformational changes of the entire VR1 region were key to determining regioselectivity. The substrate‐binding pocket of SgUGT73AM30 exhibited spatial positioning center differing from *Glycyrrhiza uralensis* UGT73P12 (PDB ID: 7C2X), implying a potential dual‐pocket binding mode reminiscent of SgUGT94‐289‐3 [47] (Figure S67). Such structural features might introduce conformational flexibility, thereby complicating molecular docking simulations of SgUGT73AM30. Although the enzymatic activity SgUGT73AM19^swapVR1^ chimeric protein also demonstrated the crucial role of VR1 counterpart in controlling its regioselectivity, the lack of a resolved crystal structure of the SgUGT73AM19‐mogrol complex limited us to further interpreting the underlying mechanistic details. Altogether, the α4 helix was shown to be critical for regioselectivity in the SgUGT73AM30/19 pair, whether this region plays a similarly decisive role in other UGT73 enzymes remains to be investigated.

## 3 Discussion

Triterpenoid saponins represent a major class of specialized metabolites in Cucurbitaceae plants, featuring remarkable structural diversity and significant economic value. Despite their importance, the molecular mechanism underlying the structural diversification of Cucurbitaceae saponins, particularly those from basal lineages, has not been fully elucidated. To date, only the biosynthetic pathways of ginsenosides, mogrosides, and classical cucurbitacins have been partially studied in a few basal genera (i.e., *Gynostemma*, *Hemsleya*, and *Siraitia*) using transcriptomics‐based approaches [20, 48] or based on poorly assembled contig‐level genomes [22], whereas a vast majority of tailoring modifications remained unknown. In contrast to the abundant genomic data available for cultivated gourds, the lack of high‐quality genome assembly and comprehensive annotations in basal Cucurbitaceae species prevents us from better understanding of the genetic basis for their metabolic diversity. To address this gap, a chromosome‐level genome of *S. grosvenorii* (Clade IV) with improved annotations was obtained in the present work, which enabled comparative genomics across the cucurbit taxa and renewed our knowledge of how the Cucurbitaceae plants have evolved the capacity for specialized saponin metabolisms.

Recently, comparative genomics has been serving as a powerful tool for unraveling specialized metabolism in plants [49]. Through comparative genomics analysis across the Cucurbitaceae family and the entire core eudicots, we found an array of tandem duplicated UGT73 glycosyltransferases key to the saponin diversity of various species. Although plant UGTs involved in glycosides biosynthesis have been extensively surveyed [3, 50], there are few real‐world cases illustrating how tandem duplications drive the neofunctionalization of UGTs to achieve the diversification of glycosylation. It is generally accepted that duplicated gene copies arising from whole‐genome multiplications (WGMs) are often functionally redundant and tend to be eventually lost in post‐WGM genome evolution [51], while those generated by tandem duplication associated with specialized metabolism are more likely to be retained [52]. Why and how those tandem duplicates escape the fate of gene loss has been a topic of interest for decades. A “dosage‐balance” model in mammalian genomes may offer an explanation. This model proposes that differential expression of retained duplicates maintains a delicate stoichiometric equilibrium, thereby allowing isozymes to respond dynamically to drastic environmental changes through an appropriate but dynamically changing dosage [53]. Nevertheless, for the immobile plants, rapid responsiveness seems less critical. Instead, plants might have preventively utilized tandem gene duplication during evolution to expand their genetic repertoire, creating more structurally diverse metabolites to fortify the arsenal of defensive chemicals against environmental challenges [2]. Our research here provides a living example of tandem duplications that gave rise to neofunctionalized enzymes and enhanced metabolic diversity. As shown in Section 2, we proved SgUGT73AM21 acting as a versatile enzyme not only retained ancestral 25‐*O*‐glycosylation but also acquired the ability to catalyze an unusual β‐(1→4) glycosylation diverting metabolic flux towards the biosynthesis of rare mogrosides. In parallel, its close paralog SgUGT73AM19 evolved stringent 24‐*O*‐glycosylation activity toward mogrol (**1**) albeit via proposed sub‐functionalization. This branching of pathway highlights how neofunctionalized UGTs redirect carbon flux toward distinct mogroside chemotypes, potentially enhancing stress adaptation in *S. grosvenorii*. The crystal structure of SgUGT73AM30 in complex with **1** revealed precise positioning of the C25 hydroxyl group toward the glucose moiety of UDPG, providing a structural basis for its catalytic activity. Sequence alignment and mutagenesis data directly establish that the VR1 region of the α4 helix determines the regioselectivity difference between SgUGT73AM30 and SgUGT73AM19. This provides a clear mechanistic example of how sequence divergence in a tandemly duplicated UGT pair can alter product specificity. However, whether similar structural determinants govern regioselectivity divergence in other UGT73 paralogs across eudicots, and whether these in vitro activities link to in vivo saponin profiles, remain open questions.

Parallel investigations in basal Cucurbitaceae genera also revealed lineage‐specific neofunctionalization events within the UGT73AM subfamily. For instance, GpUGT73AM14 (*G. pentaphyllum*) evolved multifunctionality to catalyze 3‐*O*‐glycosylation of PPD/PPT‐type saponins, whereas McUGT73AM15 (*M. charantia*) specifically mediated 7‐*O*‐glycosylation of momI. Phylogenetic analysis of UGT73AM members indicated that tandem duplication events occurred after speciation, suggesting that distinct glycosyltransferases evolved in response to lineage‐specific metabolic phenotype. This evolutionary trajectory matches recent findings in terpene synthase (TPS) gene families, where lineage‐specific duplications following ancestral origins have driven functional divergence [54]. The retained and expanded TPS repertoire across multiple species exhibits distinct catalytic specificities and bears signatures of positive selection, directly linking their diversification to ecological adaptation. Collectively, these examples underscore the intricate evolutionary mechanisms that shape plant specialized metabolism and highlight the remarkable flexibility with which nature crafts chemical diversity.

Beyond evolutionary insights, genomic studies offer another key advantage—the reliable identification of tandem duplicated genes involved in specialized metabolism. For this, an example comes from previous studies on *Centella asiatica*, where two independent research groups characterized CaUGT73AD1 and CaUGT1 (CaUGT73AD3) both capable of glycosylating the C28 carboxyl group of inipor acid [42, 43]. Although these genes share 82% amino acid sequence similarity and exhibit highly correlated expression patterns, they were reported five years apart due to the lack of genomic context in earlier transcriptome‐based analyses. Intriguingly, they are located merely ∼2 kb apart in the genome (Figure 6C). This case illustrates that genomic analysis could have efficiently revealed their tandem duplication structure, accelerated functional discovery, and reduced redundant efforts in characterizing specialized enzymes. The prevalence of such oversights also highlights a broader issue: Historically, genomics research has prioritized sequencing depth and assembly contiguity, often at the expense of annotation accuracy. Throughout this study, we frequently encounter errors and omissions in UGT annotations, necessitating extensive manual curation before sequence analysis and biochemical characterization (Figure S68). These challenges underscore an urgent need to improve the quality of gene annotation and to develop next‐generation methods for genome annotation. On the other hand, the high‐quality genomic assemblies generated here pave the way for deeper mechanistic investigations. For instance, single‐cell transcriptomics (scRNA‐seq) is increasingly employed to dissect plant specialized metabolism at single‐cell resolution, yet the accuracy of cell‐type annotation hinges critically on the availability of a well‐annotated reference genome. Looking ahead, we envision integrating single‐cell transcriptomics into a pan‐genomics initiative across the Cucurbitaceae family to capture the full spectrum of its biochemical diversity.

In conclusion, this study unraveled how tandem duplication drove functional innovation in plant specialized metabolism. By assembling the *S. grosvenorii* genome and analyzing UGT73 evolution across Cucurbitaceae, we demonstrated that neofunctionalization of tandem‐duplicated UGT73 enzymes expanded triterpenoid saponin diversity, particularly through the emergence of regiospecific glycosylation and rare sugar chain elongation activity. The crystal structure of SgUGT73AM30 revealed the molecular basis for its regio‐selectivity of vicinal diols. These findings not only provide a paradigm for tandem duplication‐driven metabolic diversification in plants but also offer deep insights for engineering glycosylation pathways to produce valuable bioactive compounds. The conserved nature of these UGT73 arrays across eudicots suggests broader applicability of this evolutionary mechanism, opening new avenues for synthetic biology applications in specialized metabolism.

## 4 Experimental Section

### 4.1 Plant Materials, Plasmids, Strains, and Chemicals

Fresh *S. grosvenorii* plants were collected from Wutong Town (25°22′35.8″N, 110°4′0.4″E), Guilin, Guangxi, China, and cultivated in a controlled growth chamber (16 h light/8 h dark cycle, 24°C). Target *UGT* genes were PCR‐amplified and initially cloned into pUC‐19 vector, followed by subcloning into the expression vector pET‐28a(+). *Escherichia coli* DH10B and JM109(DE3) strains were used for plasmid propagation and protein expression, respectively, cultured in LB medium supplemented with 50 µg/mL kanamycin. PCR amplification was performed with PrimeSTAR Max Premix (Takara Bio, Japan). Purification of DNA fragments and extraction of plasmid DNA were performed using AxyPrep Plasmid Miniprep Kit (Axygen Biosciences, U.S.A.). DNA assembly was achieved by pEASY‐Basic Seamless Cloning and Assembly Kit (TransGen Biotech, China) via homologous recombination. All chemical reference standards used in this study were commercially obtained from Wuhan ChemFaces Biochemical Co., Ltd. (China) and Shanghai Yuanye Bio‐Technology Co., Ltd. (China), with their detailed information provided in Table S10.

### 4.2 Genome Survey, Sequencing, and Assembly

Genome size and heterozygosity of *S. grosvenorii* were estimated through *k*‐mer analysis (*k* = 19) using Jellyfish v2.2.10 [55] for *k*‐mer counting and GenomeScope 2.0 [56] for modeling.

The total genomic DNA was extracted from fresh leaves of an individual *S. grosvenorii* plant, which was used to construct SMRT bell library for genome sequencing. PacBio Revio sequencing generated 1, 321, 616 high‐fidelity circular consensus sequencing (CCS) reads (average length: 20.9 kb) after adapter trimming and quality filtering with SMRT Link v13.0. The CCS data were assembled using hifiasm v0.19.8‐r603 [57], during which the allelic contigs were differentiated and duplications removed by purge_dups v1.2.5 [58] to generate a draft genome. High‐throughput chromosome conformation capture (Hi‐C) sequencing for chromosome‐level assembly was performed using standard procedure. Original reads from Hi‐C library were filtered by Fastp v0.24.0 [59] and HiCUP software and aligned to draft genome, generating valid interaction pairs, which further guided assembly of chromosome‐level genome with the help of ALLHiC v0.9.8 [60], 3D‐DNA v201008 [61], Juicer v1.6 [62] and Juciebox v1.11.08 [63]. The final genome assembly was polished manually. Visualization of Hi‐C matrix was performed by HiCExplorer v3.7.5 [64]. Assembly completeness was assessed using BUSCO v5.4.7 [65], while contiguity was indicated by LTR Assembly Index (LAI) calculated with LTR_retriever v3.0.1 [66].

### 4.3 Genome Annotation and Transcriptomics Analysis

Gene prediction was performed based on transcriptome data, *de novo* prediction, and homologous protein. RNA‐seq data were mapped to genome via hisat2 v2.2.1 [67], reconstructing transcripts by stringtie v2.2.1 [68]. *De novo* prediction was accomplished with augustus v3.5.0 [69], genscan v1.0, and glimmerhmm v3.0.4 [70]. Furthermore, protein sequences from five Cucurbitaceae species were submitted to iniport v0.13 [71] to for homologous prediction. Gene function was annotated through diamond v2.1.8 [72] against Uniprot, Pfam and NR database. For annotation of repeat sequences, RepeatModeler [73] was used to ab initio prediction based on genome sequence. LTR elements were identified by LTR_Finder [74], LTR_harvest v1.6.5 [75] and LTR_retriever v3.0.1.

We also performed RNA sequencing of fruits (*n* = 2), stems (*n* = 3), leaves (*n* = 3), and roots (*n* = 3) of *S. grosvenorii*. High‐quality total RNA was extracted using the TRIzol reagent (Invitrogen, CA, USA) according to the classical protocol. The libraries were then constructed using VAHTS Universal V6 RNA‐seq Library Prep Kit and sequenced on an Illumina Novaseq 6000 platform. Paired‐end reads (150 bp) were generated and first filtered using Fastp to remove the low‐quality reads. All RNA‐seq clean reads, including our data and publicly available datasets (PRJNA773651, PRJEB23466, and PRJNA1209606), were uniformly re‐aligned to the reference genome using HISAT2. Gene expression levels were then quantified with the Stringtie‐Quantify plugin in Tbtools [76] based on Fragments Per Kilobase of transcript per Million mapped reads (FPKM). This unified pipeline was used to analyze the resulting gene expression patterns.

### 4.4 Syntenic and Phylogenetic Analysis

Plant genomes with annotations were sourced from NCBI, CuGenDBv2 [77], FigShare (https://figshare.com/), or the IMP [78] database. Macro‐ and micro‐synteny analyses of the investigated plant genomes were conducted using the JCVI toolkit [37]. Initially, BLASTP was applied to calculate pairwise similarities between the protein sequences of the plant species. Subsequently, McScan (Python version) was employed with default parameters to identify syntenic gene pairs and visualize the resulting syntenic blocks. Criteria for defining tandem duplicates: A “UGT73 tandem array” was defined as two or more UGT73 genes located within 10 consecutive gene models on the same scaffold/chromosome (intergenic distance ≤ 400 kb), and having the conserved syntenic anchors Oxred‐PE‐UGT‐TLP (or partial retention of these markers) within 100 kb upstream/downstream. The identified UGT73 tandem arrays are summarized in Table S13.

UGT family members were identified in genome through HMMER [79] using UGT conserved domain PF00201 (https://www.ebi.ac.uk/interpro/), followed by primarily classification via pUGTdb database (https://pugtdb.biodesign.ac.cn/) [80]. Orthogroup analysis was performed by Orthofinder [81] with default parameters. The multiple sequence alignment of UGT amino acid sequences was performed using the MUSCLE v5 algorithm, followed by trimming to remove false sequences or poorly aligned regions via trimAL v.1.5.0 [82]. The phylogenetic trees of UGTs were generated by MEGA12 with bootstrap tests [83] of 1000 replications.

### 4.5 Positive Selection Analysis

The amino acid sequences of UGT73 subfamily genes were aligned by MUSCLE and further reversely translated to codon alignment by pal2nal. To ensure the correct phylogeny of species, the evolutionary tree obtained from TimeTree was set as template, and positive selection analysis was performed by CodeML [84]. Briefly, we chose the branch‐site model, of which the background branch shared the same omega values among sites, and the foreground branch was applied to different values. For the null model and alternative model, (fixed_omega, omega) were set to (1, 1) and (0, 1.5), respectively. The *p* value for foreground branch was tested via a chi‐square test.

The NEXUS format of UGT73 multiple alignments generated by Clustal Omega (MEGA12) were subjected to Bayesian Evolutionary Analysis Sampling Trees (BEAST, v10.5.0) [85] as input. The Yule model with a relaxed clock and normal prior distribution was used to estimate the divergence time of UGT73. Begoniaceae‐Cucurbitaceae split time (about 56.6–91.0 MYA) set by TimeTree (http://timetree.org/) was used to calibrate the divergence time.

### 4.6 Cloning and Heterologous Expression of UGT Candidates

UGT candidates were cloned from genomic DNA or cDNA of corresponding plants and ligated into *Nde*I/*Bam*HI sites of pET‐28a (+) plasmid. Sequenced plasmids were transformed into *E. coli* JM109(DE3). For heterologous expression, 0.3 mL overnight seed were inoculated into 10 mL Luria‐Bertani medium in 100 mL Erlenmeyer flask at 37°C. Isopropyl β‐d‐1‐thiogalactopyranoside (IPTG, 0.1 mm) was added to the culture when OD_600_ reached 0.5. The cultures were then incubated at 16°C, 180 rpm, and maintained for 18 h. After harvest by centrifugation (7000 rpm, 2 min) at 4°C, the cells were resuspended in 0.5 mL lysis buffer (20 mm Tris‐HCl, pH 8.0, 300 mm NaCl, 1 mm phenylmethylsulphonyl fluoride). Subsequently, the suspension was lysed by ultrasonication (50 W, 1 min) and centrifuged (8000 rpm, 20 min) to remove cell debris. The crude protein extracts were directly used in enzymatic assays.

### 4.7 Protein Purification

The overnight cultures of *E. coli* JM109(DE3) transformants were inoculated (2:100) into 6 L of LB with kanamycin selection. When OD_600_ reached 0.5, a final concentration of 0.1 mm IPTG was added to induce protein expression. The cultures grew further at 16°C for 18 h. The cells were harvested, resuspended in lysis buffer, and subsequently crashed by sonification. ProteinIso Ni‐NTA Resin (TransGen Biotech, China) was used to purify the soluble His_6_‐tagged protein according to the manufacturer's protocol. The crude extracts and purified UGTs were analyzed by SDS‐PAGE. For crystallization, the protein was further purified by anion exchange chromatography and size exclusion chromatography using a HiTrap Q FF column (Cytiva, Sweden) and Superdex 200 Increase 10/300 GL column (Cytiva, Sweden), respectively. Protein concentration was determined using the Bradford assay (Sangon Biotech) in triplicate measurements.

### 4.8 In Vitro Enzymatic Assay

The in vitro enzymatic assay was conducted by the addition of 100 µm UDPG and 50 µm substrates at 30°C overnight. Crude lysates were used for qualitative screening, while purified enzymes were used for quantitative comparisons. After quenching with *n*‐butanol, concentrated to dry and re‐dissolved in 100 µL methanol, 20 µL or 1 µL of filtered mixture was analyzed using HPLC‐UV and liquid chromatography–mass spectrometry (LC‐MS), respectively.

The kinetic parameters of SgUGT73AM19, SgUGT73AM30, and SgUGT74DD1 were determined in a 200 µL reaction solution consisting of 20 mm Tris‐HCl (pH 8.0), 300 mm NaCl, and 10 µg purified enzymes. The concentration of UDPG was fixed at 2 mm. Mogrol (**1**) concentration varied from 2.5 to 200 µm. The reactions were incubated for 60–90 min at 30°C and quenched by the addition of *n*‐butanol followed by analysis using HPLC‐UV. The enzyme kinetic parameters were calculated by Origin 20224 based on three independent experiments.

### 4.9 Metabolites Analysis

HPLC‐UV analysis was performed using Dionex Ultimate 3000 (Thermo Scientific, U.S.A.) equipped with HC‐C18 column (4.6 × 250 mm, 5 µm, Agilent, U.S.A.). The mobile phase consisted of water (A) and acetonitrile (B). Three different methods were adopted. Method 1 was used to detect mogrol monoglucoside following a linear gradient program: 25% to 60% B (11 min), 95% B maintained for 3 minutes. Method 2 was set as follows: 20% to 40% B (14 min), 95% B maintained 3 min to analyze mogrosides with at least two sugar moieties. Method 3 was set to detect other glycoside products: 20% to 100% B (16 min), 100% B maintained for 5 min. The temperature of column oven maintained at 40°C with a flow rate of 1.0 mL/min. The ultraviolet detection wavelength was set at 210 nm.

UPLC‐ESI‐HRMS data were acquired using Agilent 6545 UHPLC‐Q‐TOF LC/MS (Agilent, U.S.A.) or Q Exactive hybrid quadrupole‐Orbitrap mass spectrometer (Thermo Scientific, U.S.A.) equipped with a ZORBAX 300SB‐C18 column (2.1 × 100 mm, 3.5 µm, Agilent, U.S.A.) The mobile phase consisted of water (A) and acetonitrile (B). A linear gradient was set as follows: 20% B to 100% B (15 min), 100% B maintained for 3 min. The flow was 0.3 mL/min, and the injection volume was 1 µL. The mass acquisition was performed in negative ionization mode with full scan (100–1500).

### 4.10 Large Scale Preparation of Glycoside Products

Cell lysates utilized in the scaled‐up synthesis of glycoside products were prepared as described above, albeit with some adjustments. Briefly, the cell culture volume was enlarged to 200 mL, yielding 5 mL of lysate generated from 10 minutes of sonication. For the scaled‐up reactions, 5–10 mg of substrates and 2 equivalents of UDPG were combined in a total volume of 5 mL cell lysates. The reactions were incubated at 30°C for 24 h and quenched by the addition of 5 mL of methanol. Subsequently, the mixtures were dried via a rotary evaporator and dissolved in 1 mL of methanol. The crude extracts were subjected to a SilGreen HPLC column (250 × 20 mm, 5 µm, 12 nm) and separated using a semi‐preparative Ultimate 3000 HPLC system (DIONEX, U.S.A.) with a mobile phase of 30%–80% methanol. Each fraction was checked for the target compound using HPLC‐UV analyses. The target fractions were combined and dried, yielding purified compounds for NMR analyses. The ^1^H, ^13^C, and 2D NMR spectra of the glycoside products were recorded using an Avance III 400 and 500 MHz spectrometer (Bruker, Germany). Chemical shifts (ppm) were referenced to the solvent peaks at δ_H_/δ_C_ = 3.31/49.0 ppm (methanol‐*d*_4_), δ_H_/δ_C_ = 7.58/135.9 ppm (pyridine‐*d*_5_).

### 4.11 Crystallization, Data Collection, and Structure Determination of SgUGT73AM30

To obtain a crystal structure of SgUGT73AM30 in complex with mogrol (**1**), the protein was incubated with a 20‐fold molar excess of substrate prior to crystallization. Crystals in complex with **1** were grown at 18°C using the Hanging Drop vapor diffusion method. SgUGT73AM30‐mogrol‐UDP complex crystals were successfully obtained in a reservoir solution containing 0.23 M ammonium fluoride and 19% (w/v) polyethylene glycol 3350. After growth for 2 days, the crystals designated for data collection were cryoprotected with 20% ethylene glycol.

Data collection for the SgUGT73AM30‐mogrol‐UDP complex crystal was carried out at the Shanghai Synchrotron Radiation Facility (SSRF) beamline BL19U1 and processed using the HKL3000 program suite. The initial model was constructed using the CCP4 suite, followed by refinement using the phenix program, and finalized via Coot. All structural representations in the figures were generated using PyMOL.

### 4.12 Protein Structure Prediction, Molecular Docking, and Site‐Directed Mutagenesis

The structures of SgUGT73AM19 and SgUGT73AM30^swap94‐108^ were predicted by AlphaFold3 server. Determination of active site pocket and molecular docking of substrates (UDPG and/or **1**) with SgUGT73AM30 and SgUGT73AM30swap^94‐108^ were performed with Autodock‐vina v1.2.0 [86]. Residues within 4 Å of **1** were selected, and different residues in two enzymes were identified based on pairwise sequence alignment visualized by ESPript 3.2 [87] (Figure S69). Site‐directed mutagenesis was carried out using PCR with primer pairs containing mutation sites. The plasmids pET28a‐SgUGT73AM30 and pET28a‐SgUGT73AM19 were used as templates.

### 4.13 Molecular Dynamics Simulations and Analysis

Molecular dynamics (MD) simulations were performed using GROMACS v2021.3. The protein topology was generated with the AMBER99SB‐ILDN force field. Partial atomic charges of the substrate ligands were derived using the RESP2 method at the B3LYP/def2‐TZVP level, and the corresponding ligand topology files were generated using Sobtop. Each system was solvated in a truncated octahedral box filled with TIP3P water molecules, maintaining a minimum distance of 1 nm between the solute and the box boundary. Appropriate numbers of Na^+^ and Cl^−^ ions were added to neutralize the total charge of the system. Energy minimization was carried out using 2500 steps of the steepest descent algorithm followed by 2500 steps of the conjugate gradient method. The minimized system was subsequently equilibrated under an NVT ensemble for 100 ps and then under an NPT ensemble for an additional 100 ps at 300 K. After equilibration, a 60 ns production MD simulation was conducted under periodic boundary conditions. Long‐range electrostatic interactions were treated using the Particle Mesh Ewald (PME) method, with a cutoff distance of 1.0 nm for non‐bonded interactions. The integration time step was set to 2 fs, the system pressure was maintained at 1 bar, and trajectories were saved every 10 ps.

### 4.14 Statistical Analysis

Enzyme regioselectivity was determined based on the ratio of the C25‐*O*‐and C24‐*O*‐glycosylated products, calculated as the HPLC‐UV peak area of a specific product divided by the sum of both products. The sample size for all quantitative experiments in this study was *n* = 3, representing three independent biological replicates. Data are presented as the mean ± SD. For comparisons between two experimental groups, an unpaired and two‐tailed Student's t‐test was employed. Statistical significance was defined as *P* < 0.05, with exact P‐values indicated directly in the graphs using symbols (*, *P* < 0.05; **, *P* < 0.01; ***, *P* < 0.001; ****, *P* < 0.0001; ns, no significant difference). All statistical analyses and graphical representations were performed using GraphPad Prism 9 (GraphPad Software, USA).

## Author Contributions

**Guangyi Wang**: methodology, investigation, visualization, writing – original draft. **Yanchen Zhang**: investigation. **Xuehui Dai**: methodology, investigation. **Haili Liu**: investigation. **Yuhan Wu**: investigation. **Xiaowei Zhang**: investigation. **Yong Wang**: conceptualization, writing – review and editing, supervision, funding acquisition. **Yuwei Sun**: conceptualization, writing – review and editing, supervision, funding acquisition. **Chenfei Tian**: investigation. **Jiayu Lu**: investigation. **Zhaotao Yan**: investigation. **Yiming Yang**: investigation. **Hongkai Fan**: investigation. **Mengmeng Li**: methodology, investigation, visualization.

## Funding

This work is financially supported by the National Key R&D Program of China (No. 2024YFA0919900), National Natural Science Foundation of China (No. 32570312 and No. 32571708), the State Key Laboratory of Plant Trait Design, the Key Laboratory of Plant Carbon Capture (CNTD004), Chinese Academy of Sciences, Shanghai Municipal Science and Technology Major Project, and the 2025 Key Technology Research and Development Program: Synthetic Biology Project (25HC2810300).

## Conflicts of Interest

The authors declare no conflicts of interest.

## Supporting information




**Supporting File**: advs76159‐sup‐0001‐SuppMat.pdf.

## Data Availability

The raw sequencing data and assembled genome described in this work have been deposited in the China National Center for Bioinformation (CNCB, https://www.cncb.ac.cn). Specifically, the chromosome‐level genome assembly of *S. grosvenorii* is available under BioProject accession number PRJCA063234, with raw sequencing data accessible under CRA042162 and the assembled genome under GWHJIFB00000000.1. Additionally, nucleotide sequences of all functionally characterized UGTs have been deposited in GenBank under accession numbers PZ345139–PZ345155. All other materials described in this study are available from the corresponding author upon reasonable request.
