# Supplementary material for: Tandem Duplication‐Driven Neofunctionalization of UDP‐Glycosyltransferases Shapes the Diversification of Triterpenoid Saponins in the Cucurbitaceae
Source: Adv Sci (Weinh). 2026 Jun 18;13(51):e76159. doi: 10.1002/advs.76159 (PMC13336362; doi:10.1002/advs.76159)
Supplement: Supplementary file 1 [file ADVS-13-e76159-s001.pdf]

# Supporting Information

**Tandem duplication-driven neo-functionalization of UDP-glycosyltransferases shapes the diversification of triterpenoid saponins in the Cucurbitaceae**

*Guangyi Wang, Mengmeng Li, Xuehui Dai, Yanchen Zhang, Yuhan Wu, Zhaotao Yan, Chenfei Tian, Hongkai Fan, Haili Liu, Xiaowei Zhang, Yiming Yang, Jiayu Lu, Yuwei Sun*, Yong Wang**

1. Supporting Figures S1-S69
2. Supporting Tables S1-S13

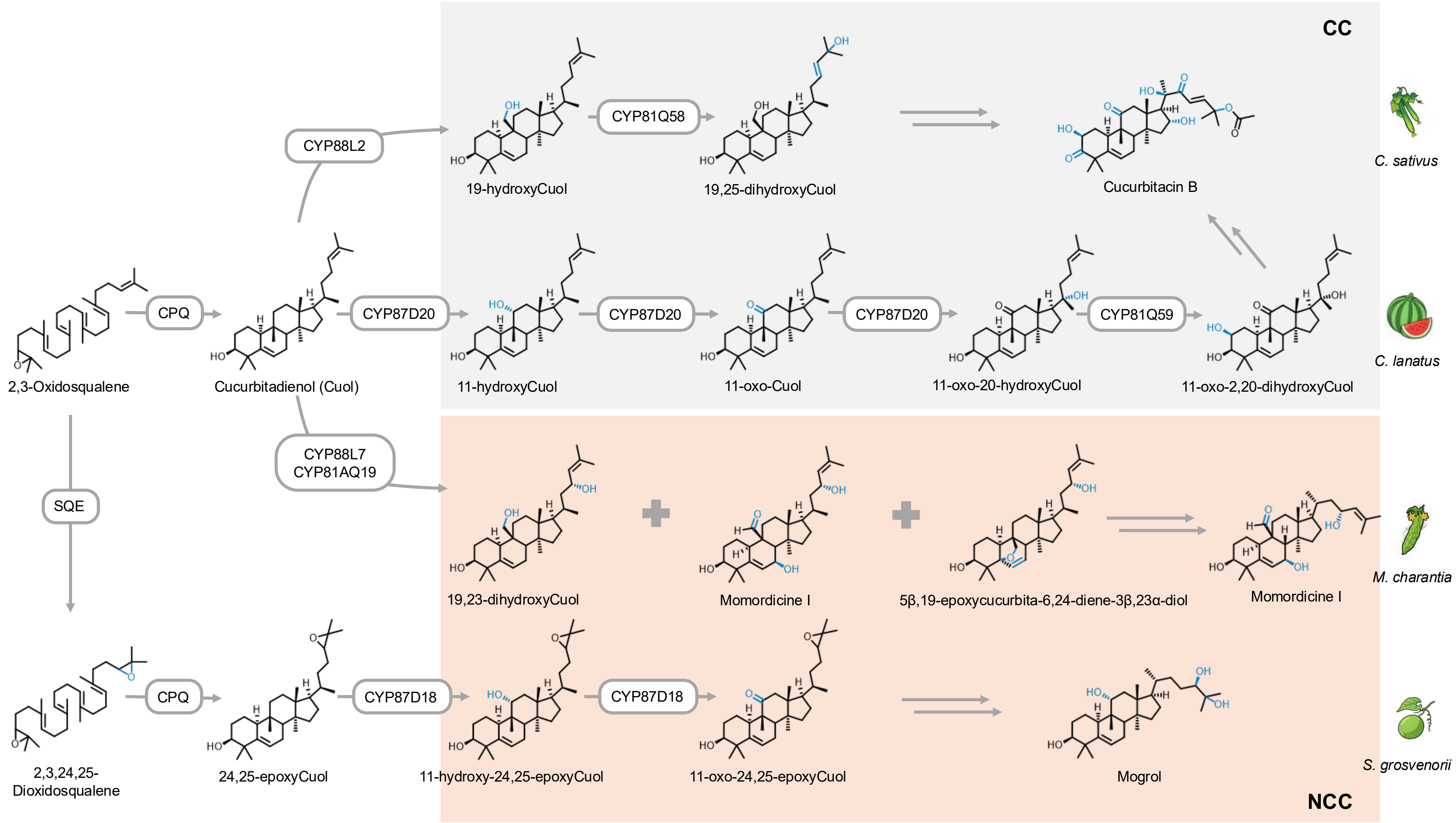


**Figure S1. Previously characterized oxidation steps in the Cucurbitaceae family.**

CC, Classical cucurbitacins; NCC, non-canonical cucurbitacins

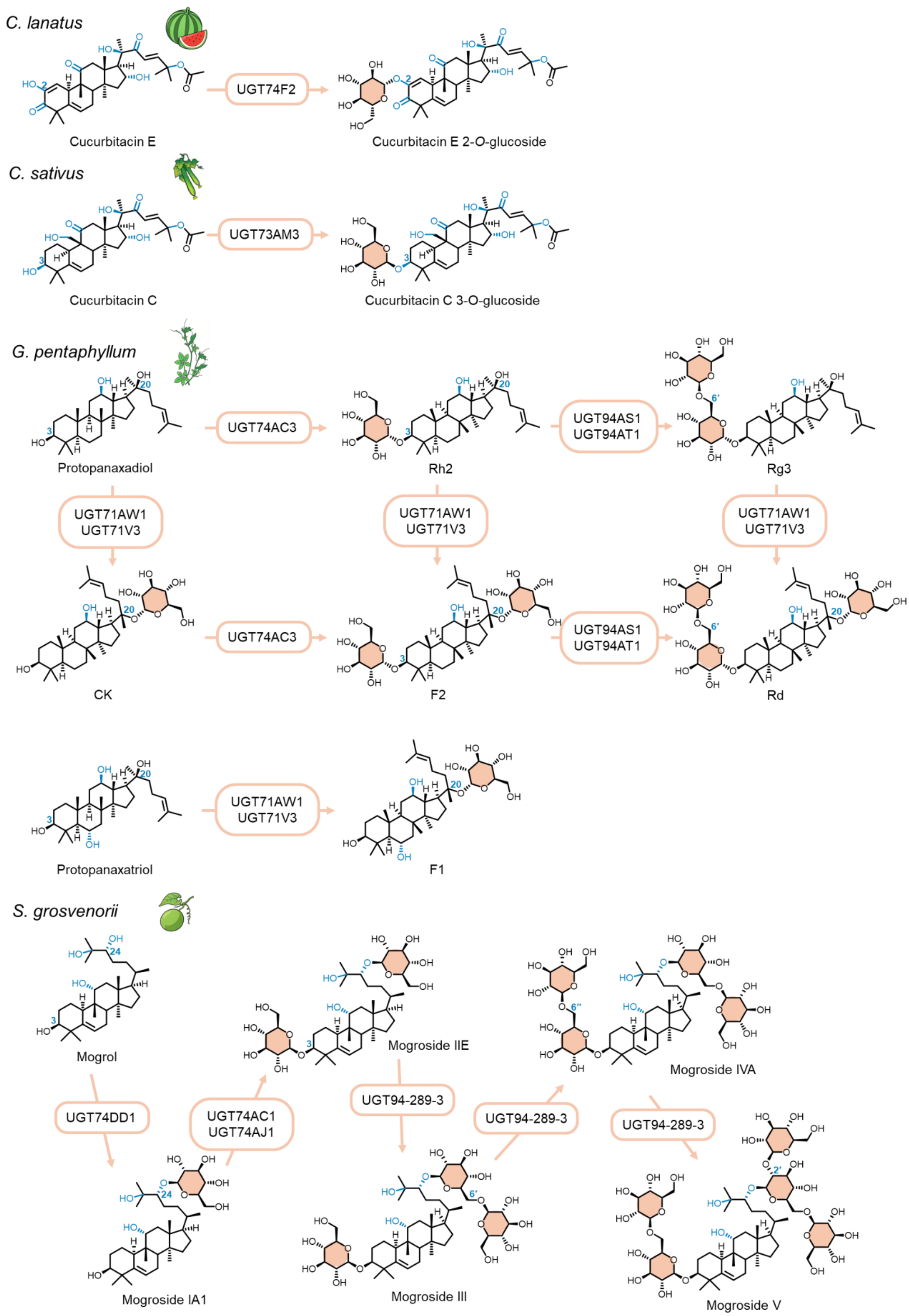


**Figure S2. Reported Cucurbitaceae UGTs participating in saponins biosynthesis.**

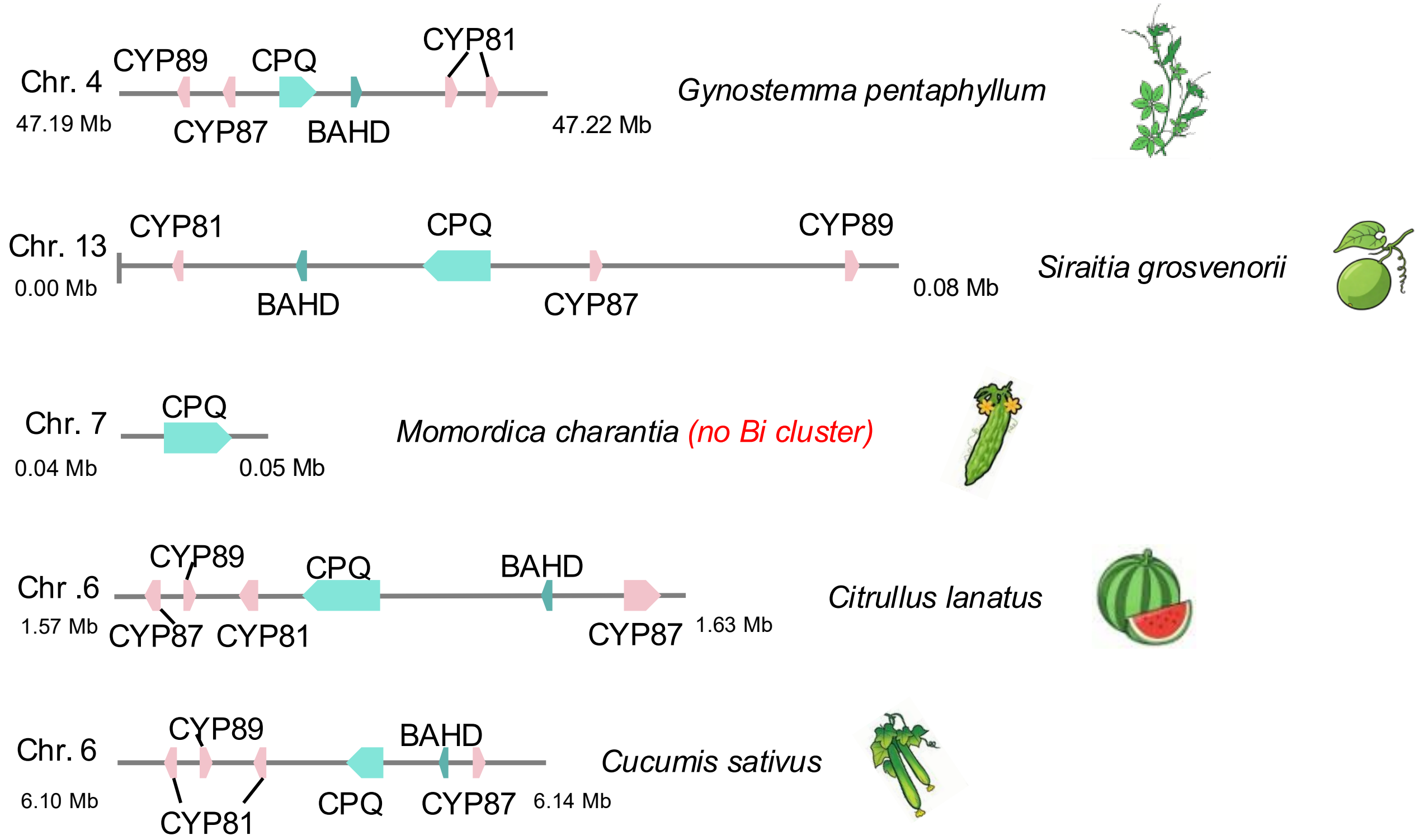


**Figure S3. The cucurbitacin biosynthetic gene clusters (BGCs) in different Cucurbitaceae species.** The cucurbitacin BGCs are present in most of the cucurbits, except for bitter melon

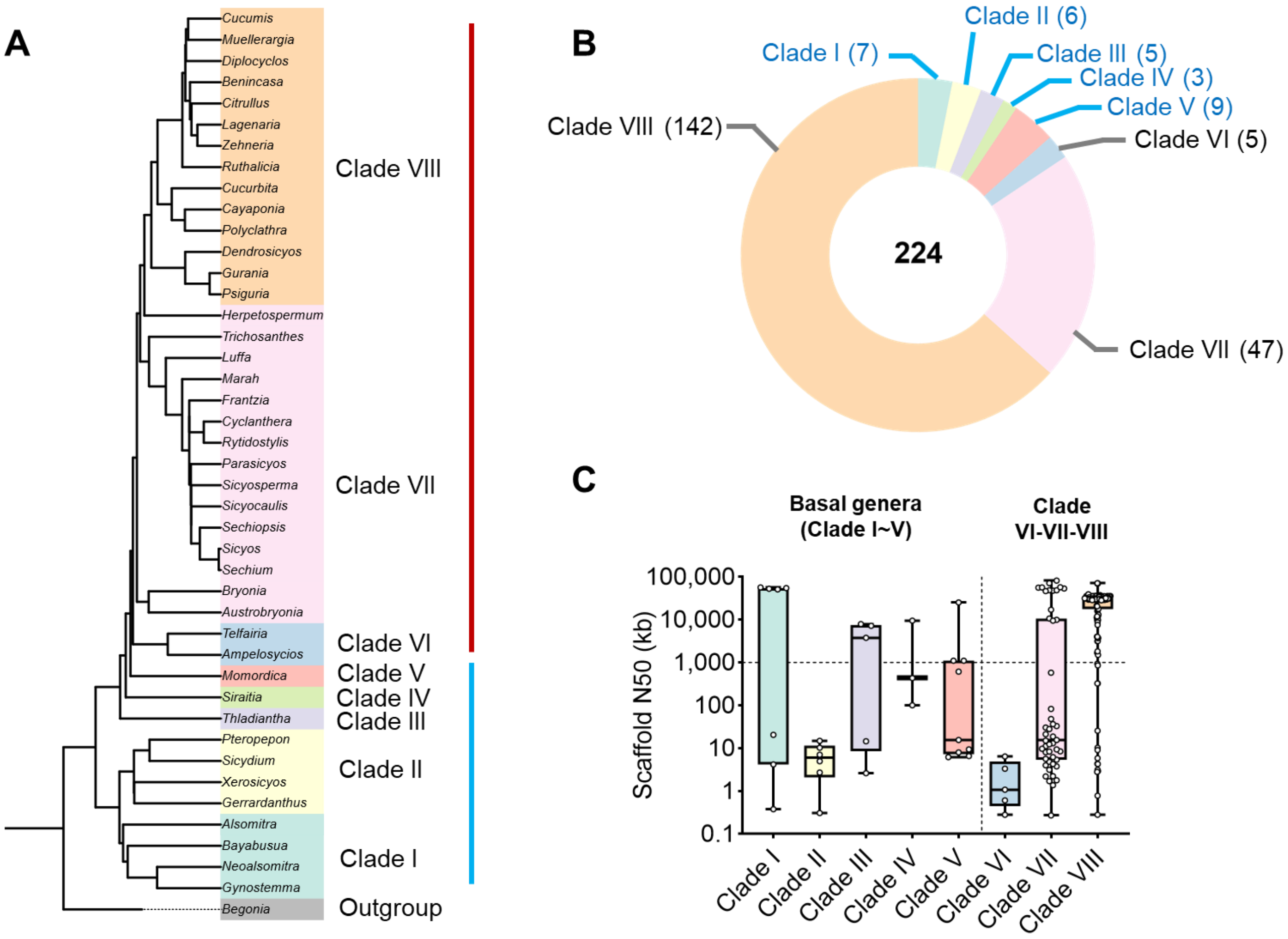


**Figure S4. Sequenced genomes of Cucurbitaceae family plants.** (**A**) Phylogenetic tree of Cucurbitaceae genera having their genomes sequenced. Blue line, basal genera (Clade I~V); Red line, late-diverged clades (Clade VI-VIII). (**B**) The pie chart shows the number of whole genome sequencing projects in different clades. The basal genera clades are indicated in blue. (**C**) Scaffold N50 of the assembly represents the quality of Cucurbitaceae genomes. Those with scaffold N50>1000 kb (1 Mb) is considered well assembled. Box plots show the median (center line), interquartile range (box), and full data range (whiskers). Sample sizes (n) for each clade are: I (7), II (6), III (5), IV (3), V (9), VI (5), VII (47), VIII (142).

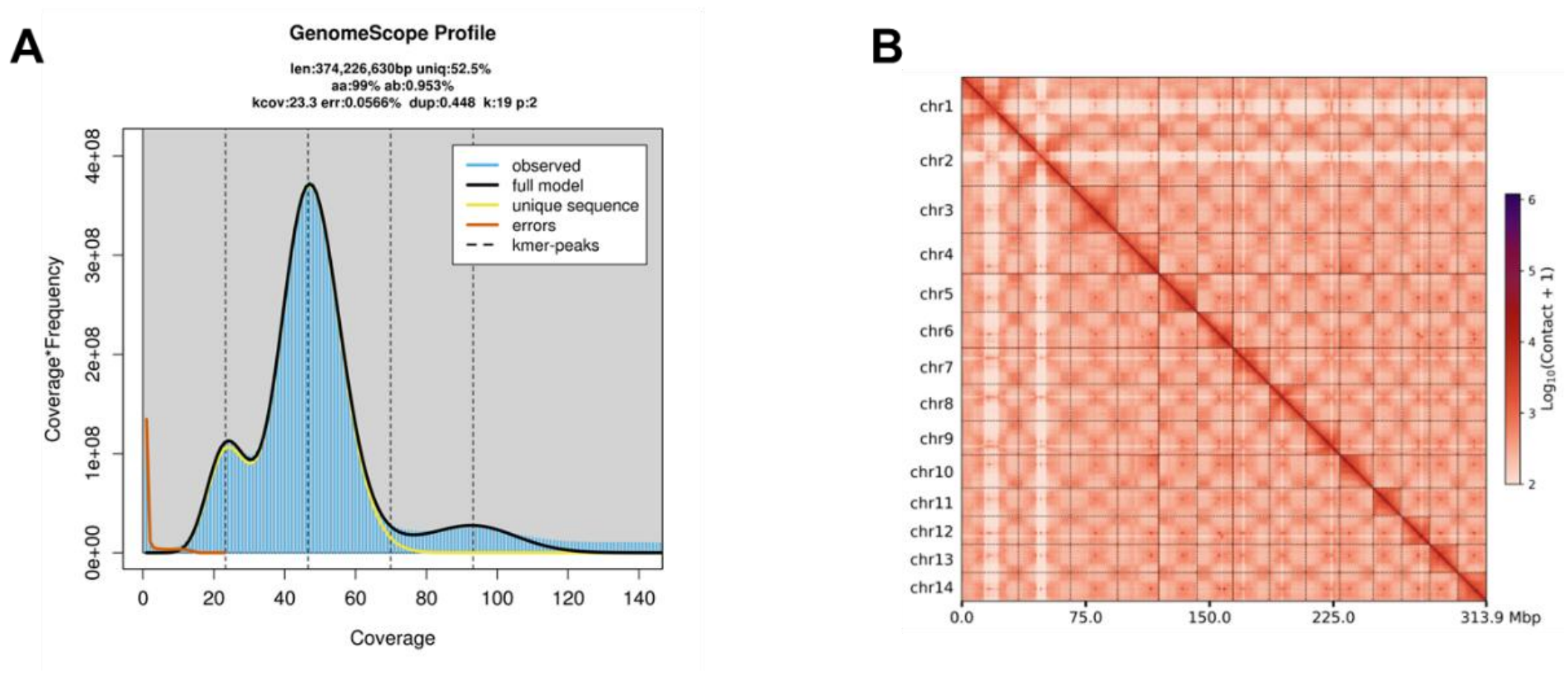


**Figure S5. Estimation of genome size and chromatin interactions in *S. grosvenorii*.** (**A**) Genome size estimation of *S. grosvenorii* based on 19-mer frequency analysis. (**B**) Hi-C interaction heatmap of the *S. grosvenorii* chromosomes.

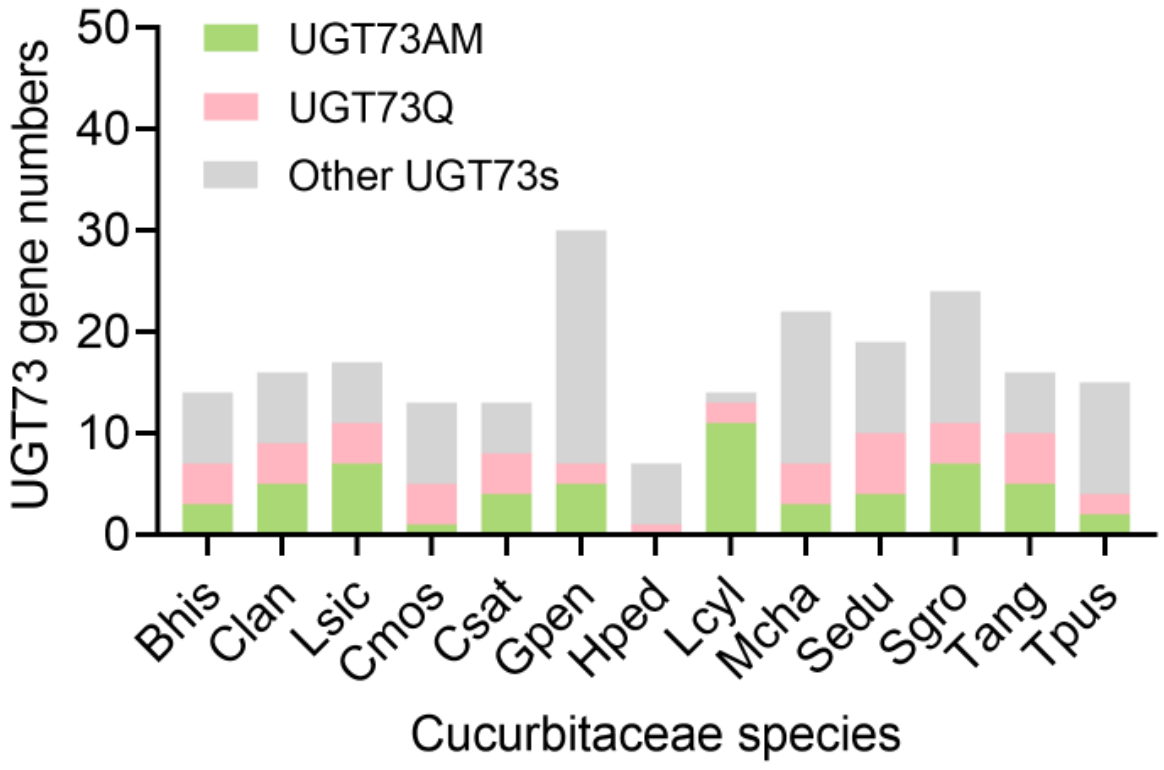


| | Bhis | Clan | Lsic | Cmos | Csat | Gpen | Hped | Lcyl | Mcha | Sedu | Sgro | Tang | Tpus |
|---|---|---|---|---|---|---|---|---|---|---|---|---|---|
| UGT73 | 14 | 16 | 17 | 13 | 13 | 30 | 7 | 14 | 22 | 19 | 24 | 16 | 15 |
| UGT73AM | **3** | **5** | **7** | **1** | **4** | **5** | **0** | **11** | **3** | **4** | **7** | **5** | **2** |
| UGT73Q | **4** | **4** | **4** | **4** | **4** | **2** | **1** | **2** | **4** | **6** | **4** | **5** | **2** |
| Others | 7 | 7 | 6 | 8 | 5 | 23 | 6 | 1 | 15 | 9 | 13 | 6 | 11 |

**Figure S6. Distribution of *UGT73AM* and *UGT73Q* subfamilies genes across Cucurbitaceae species.** The table below the column graph lists the gene numbers of the UGT73 family for each analyzed species. Abbreviations: *Benincasa hispida* (Bhis), *C. lanatus* (Clan), *Lagenaria siceraria* (Lsic), *Cucurbita. moschata* (Cmos), *C. sativus* (Csat), *G. pentaphyllum* (Gpen), *H. pedunculosum* (Hped), *Luffa aegyptiaca* (Lcyl), *M. charantia* (Mcha), *Sechium edule* (Sedu), *S. grosvenorii* (Sgro), *Trichosanthes anguina* (Tang) and *T. pustulata* (Tpus).

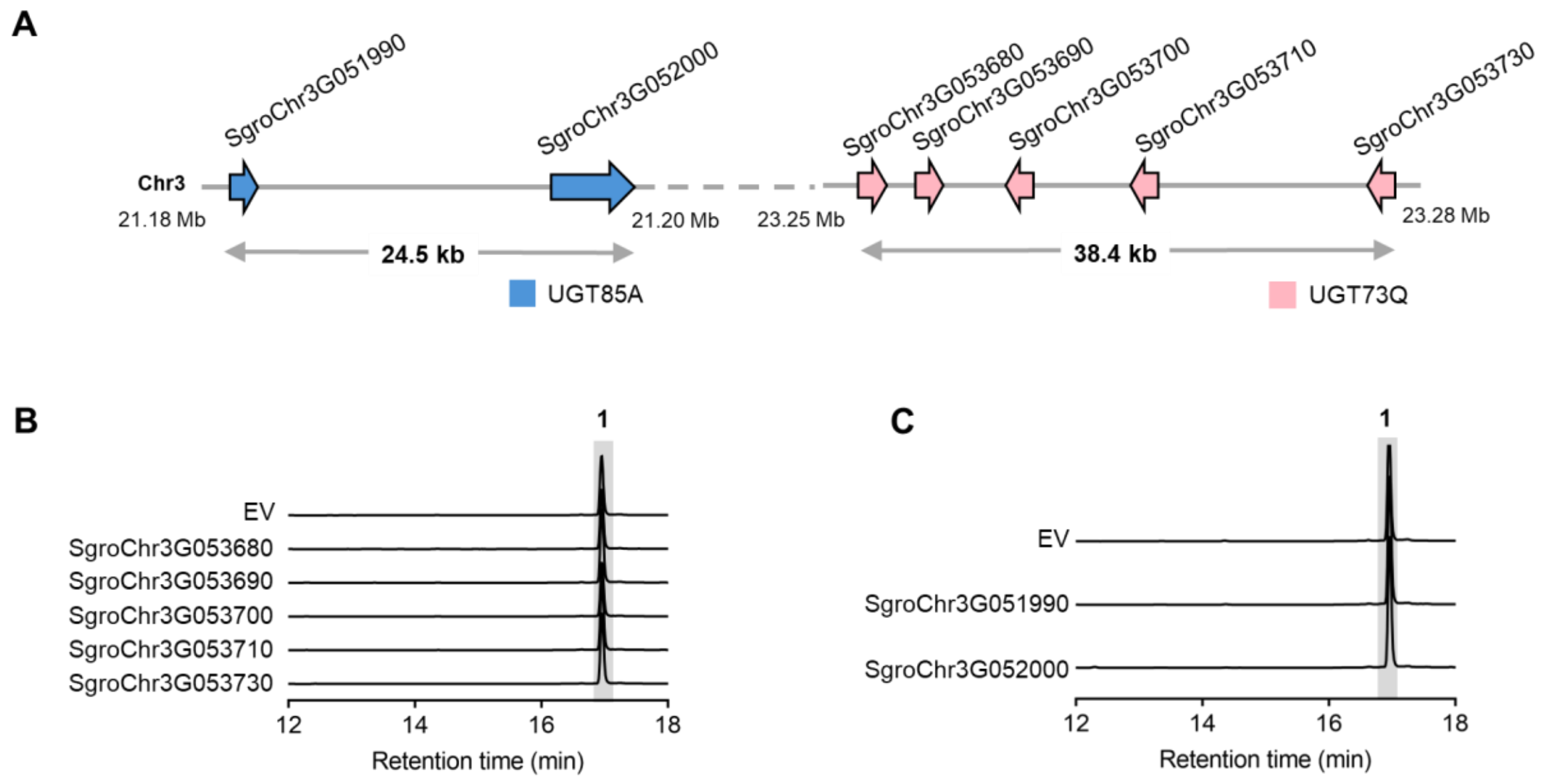


**Figure S7. Screening of UGT85A and UGT73Q candidates.** (**A**) Tandem array of *UGT73Q* and *UGT85A* genes on chromosome 3. (**B**) HPLC chromatograms of the in vitro assay of SgUGT73Q toward mogrol (**1**) (C) HPLC chromatograms of the in vitro assay of SgUGT85A toward mogrol (**1**). EV, empty vector (pET28a).

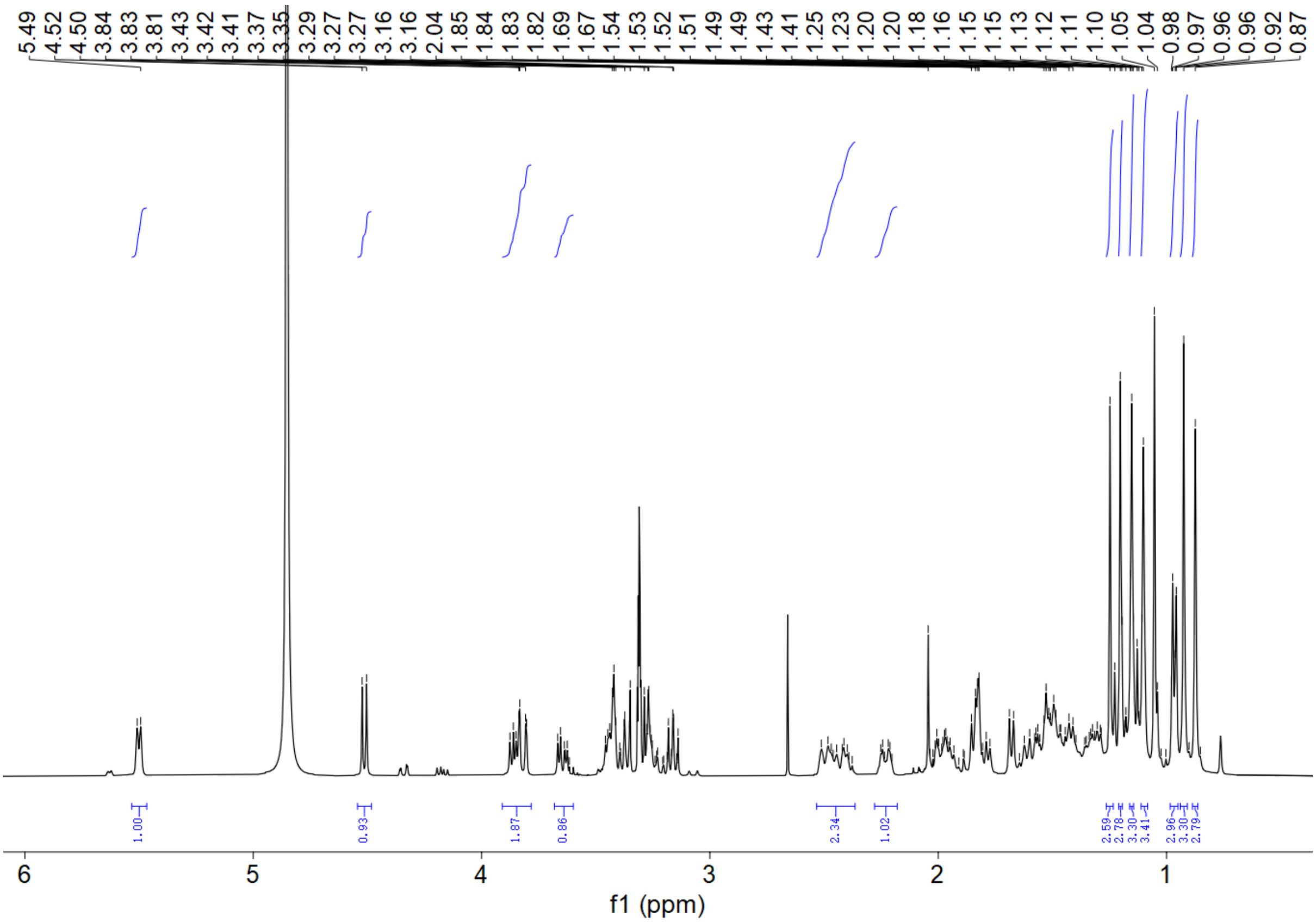

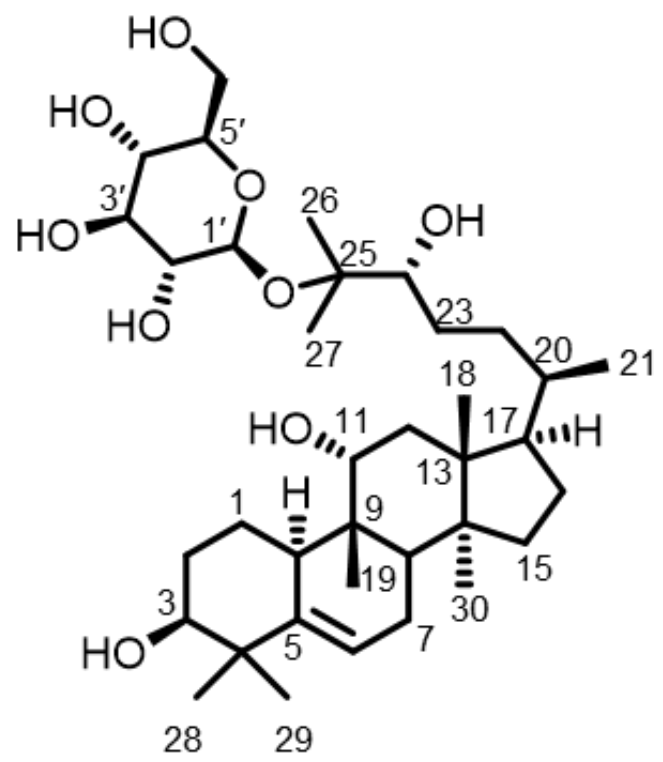

**Figure S8. $^{1}$H (400 MHz, methanol-*d*4) NMR spectrum of mogrol 25-*O*-glucoside (3).**

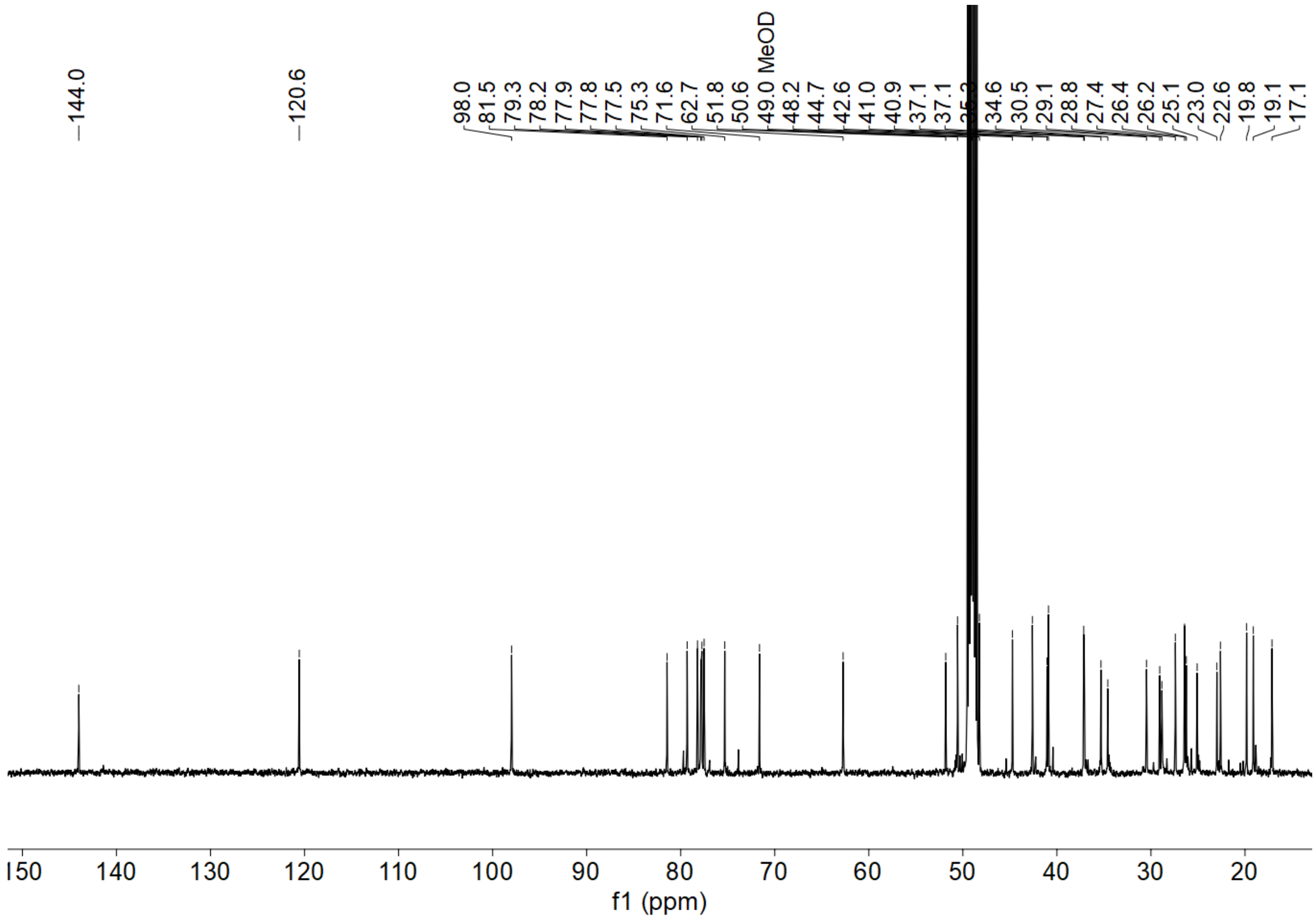

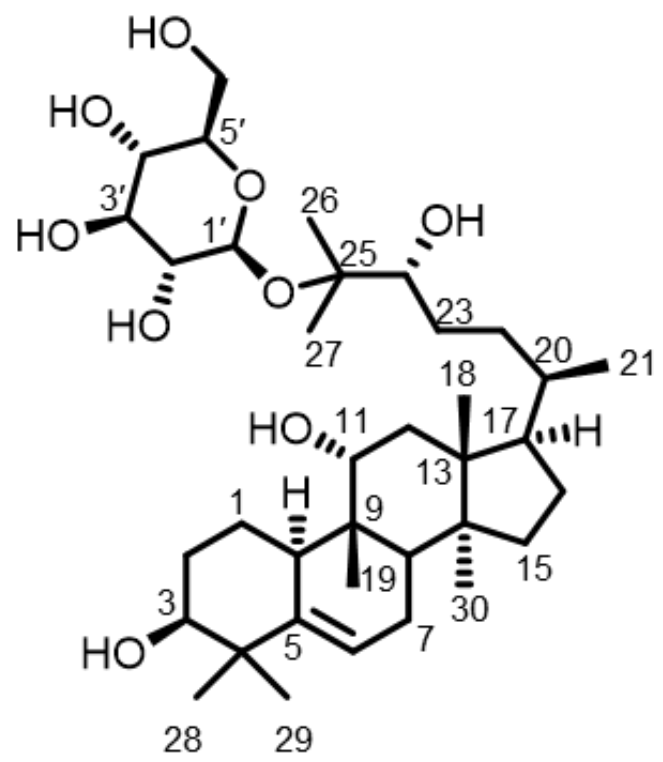

**Figure S9. $^{13}$C (125 MHz, methanol-*d*$_4$) NMR spectrum of mogrol 25-*O*-glucoside (3).** $\delta_C$ (ppm): 26.4 (C-1); 30.5 (C-2); 77.5 (C-3); 42.6 (C-4); 144.0 (C-5); 120.6 (C-6); 25.1 (C-7); 44.7 (C-8); 40.9 (C-9); 37.1 (C-10); 79.3 (C-11); 41.1 (C-12); 48.2 (C-13); 50.6 (C-14); 35.3 (C-15); 28.8 (C-16); 51.8 (C-17); 17.1 (C-18); 26.2 (C-19); 37.1 (C-20); 19.1 (C-21); 34.6 (C-22); 29.1 (C-23); 77.9 (C-24); 81.5 (C-25); 22.6 (C-26); 23.0 (C-27); 26.4 (C-28); 27.4 (C-29); 19.8 (C-30); 98.0 (C-1′); 75.3 (C-2′); 78.2 (C-3′); 71.6 (C-4′); 77.8 (C-5′); 62.7 (C-6′).

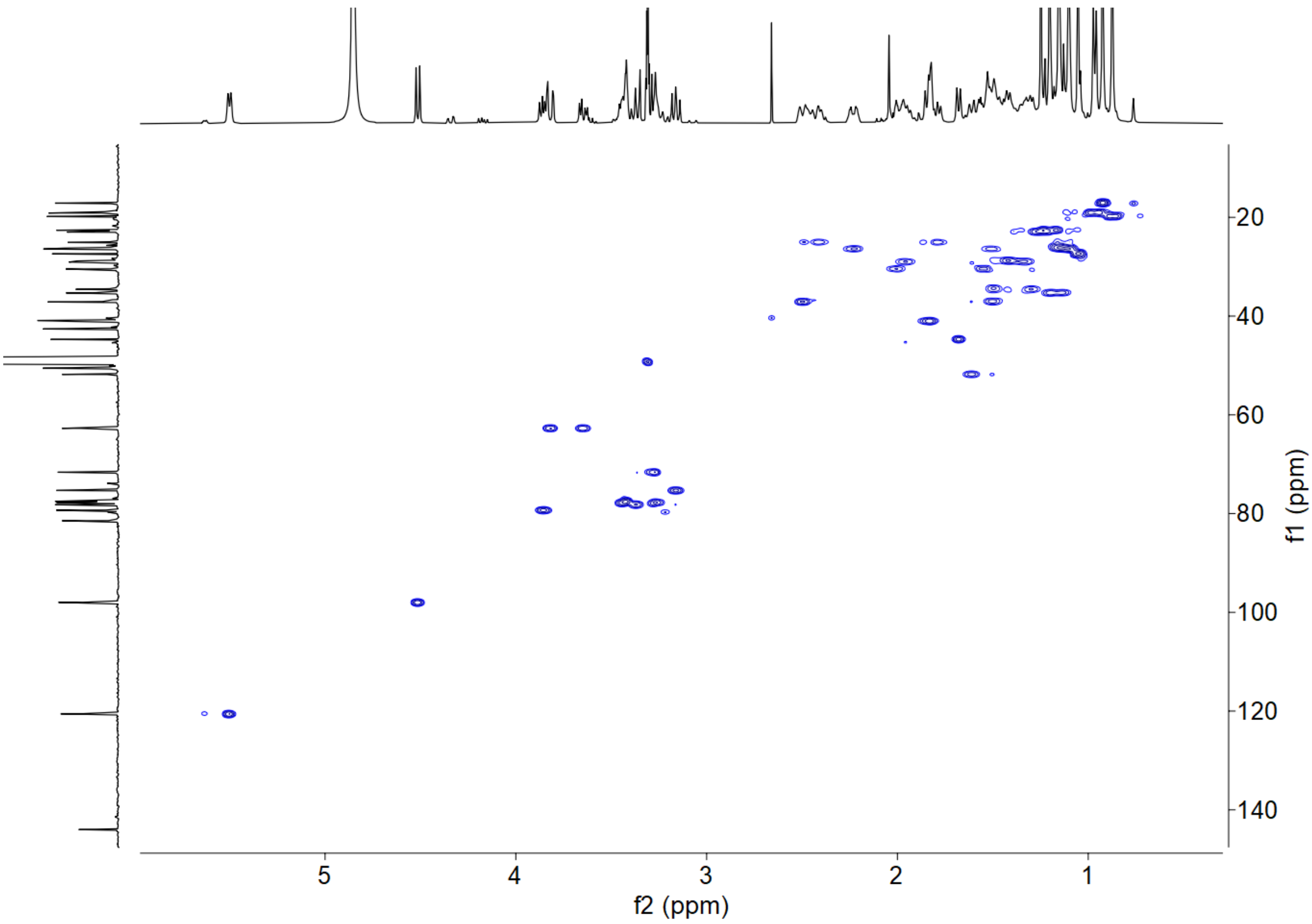


**Figure S10. HSQC spectrum of mogrol 25-*O*-glucoside (3).**

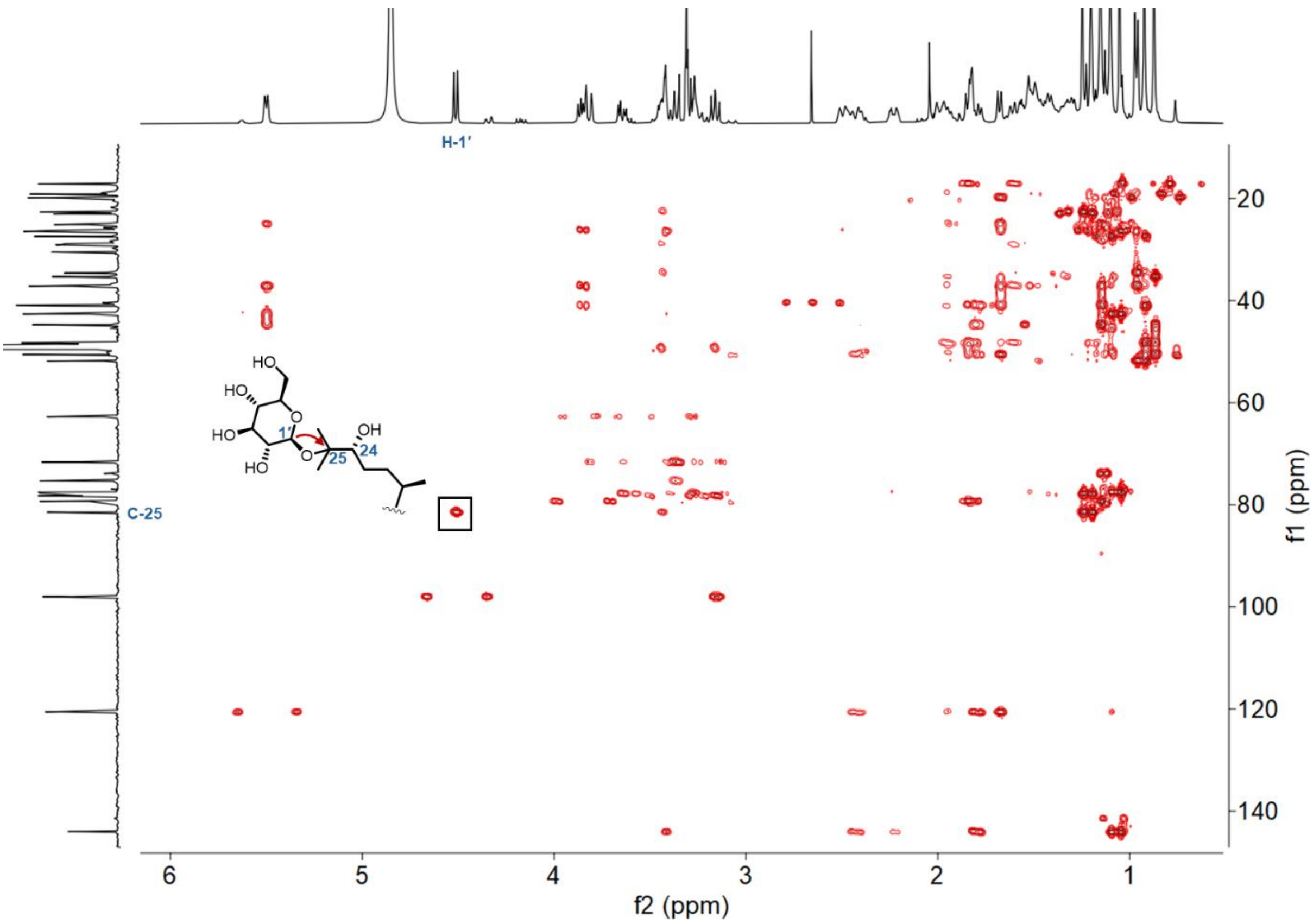


**Figure S11. HMBC spectrum of mogrol 25-*O*-glucoside (3).**

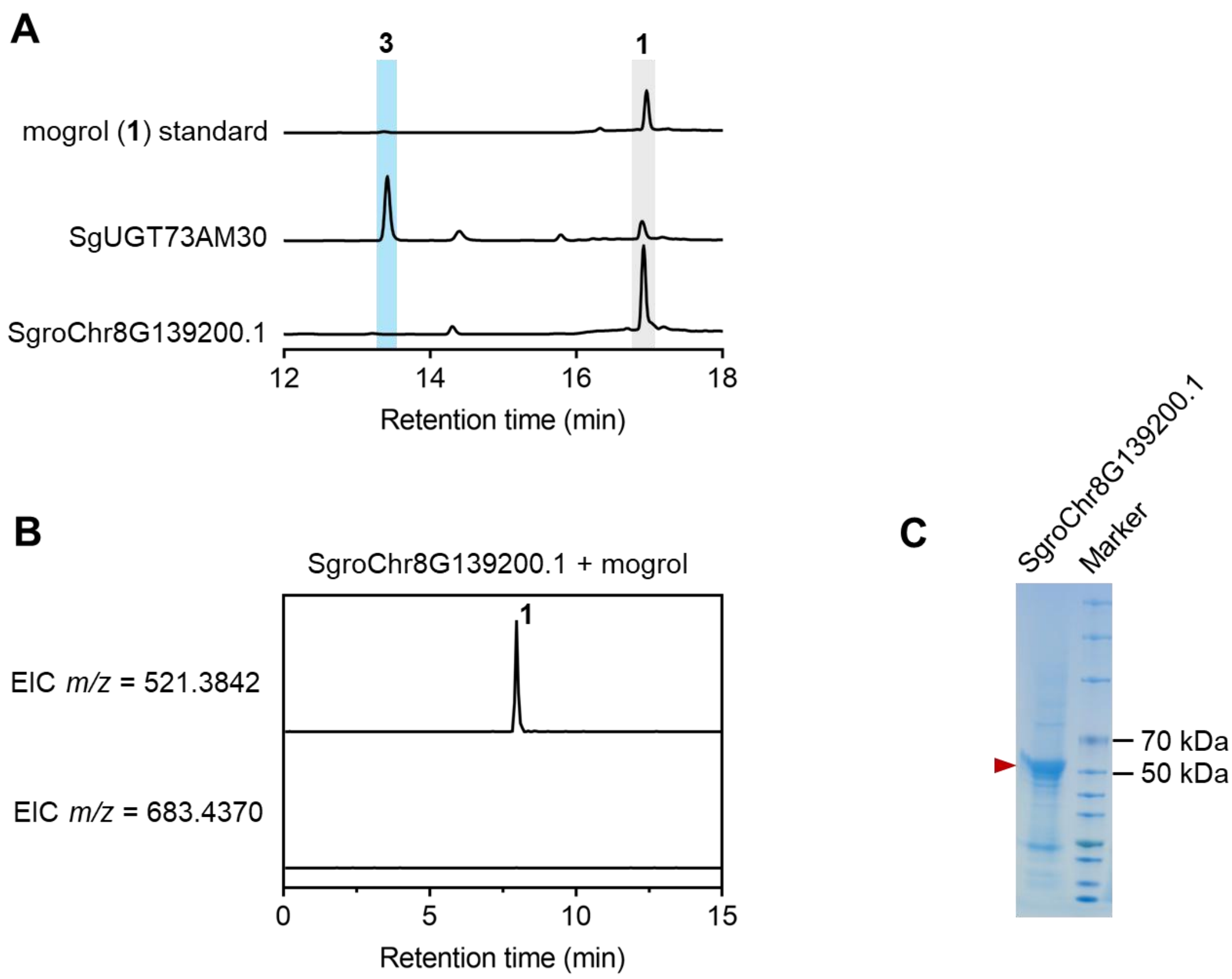


**Figure S12. Functional characterization of the previously reported SgUGT73D5 (annotated as SgroChr8G139200.1).** (**A**) HPLC-UV chromatogram of SgUGT73D5, showing no detectable glycosylation activity toward substrate **1**. (**B**) LC-MS analysis of SgUGT73D5, confirming the absence of product formation from substrate **1**. (**C**) SDS-PAGE analysis of purified SgUGT73D5 protein.

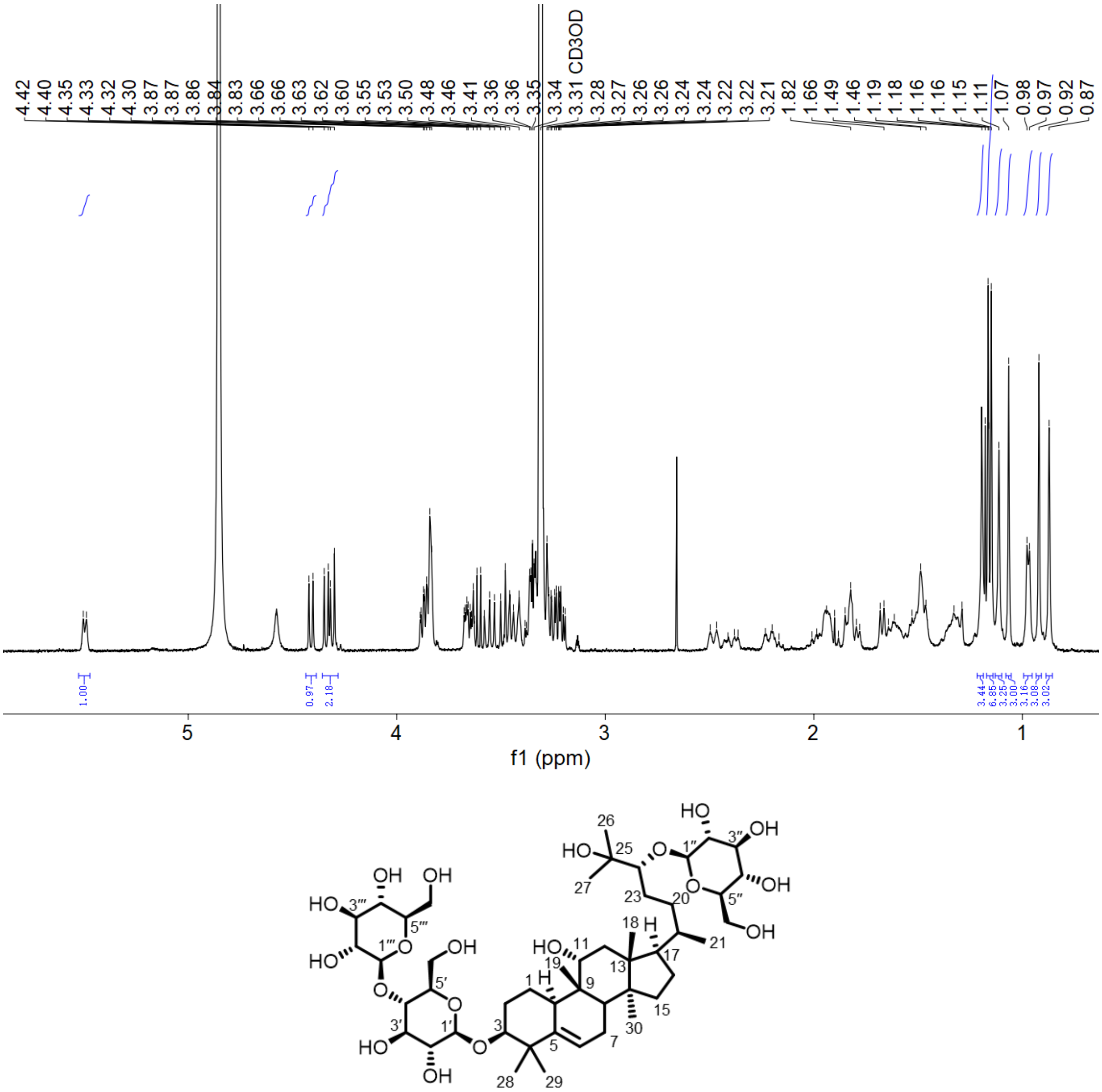

**Figure S13. ¹H (400 MHz, methanol-$d_4$) NMR spectrum of mogroside IIE 4′-*O*-glucoside (5).**

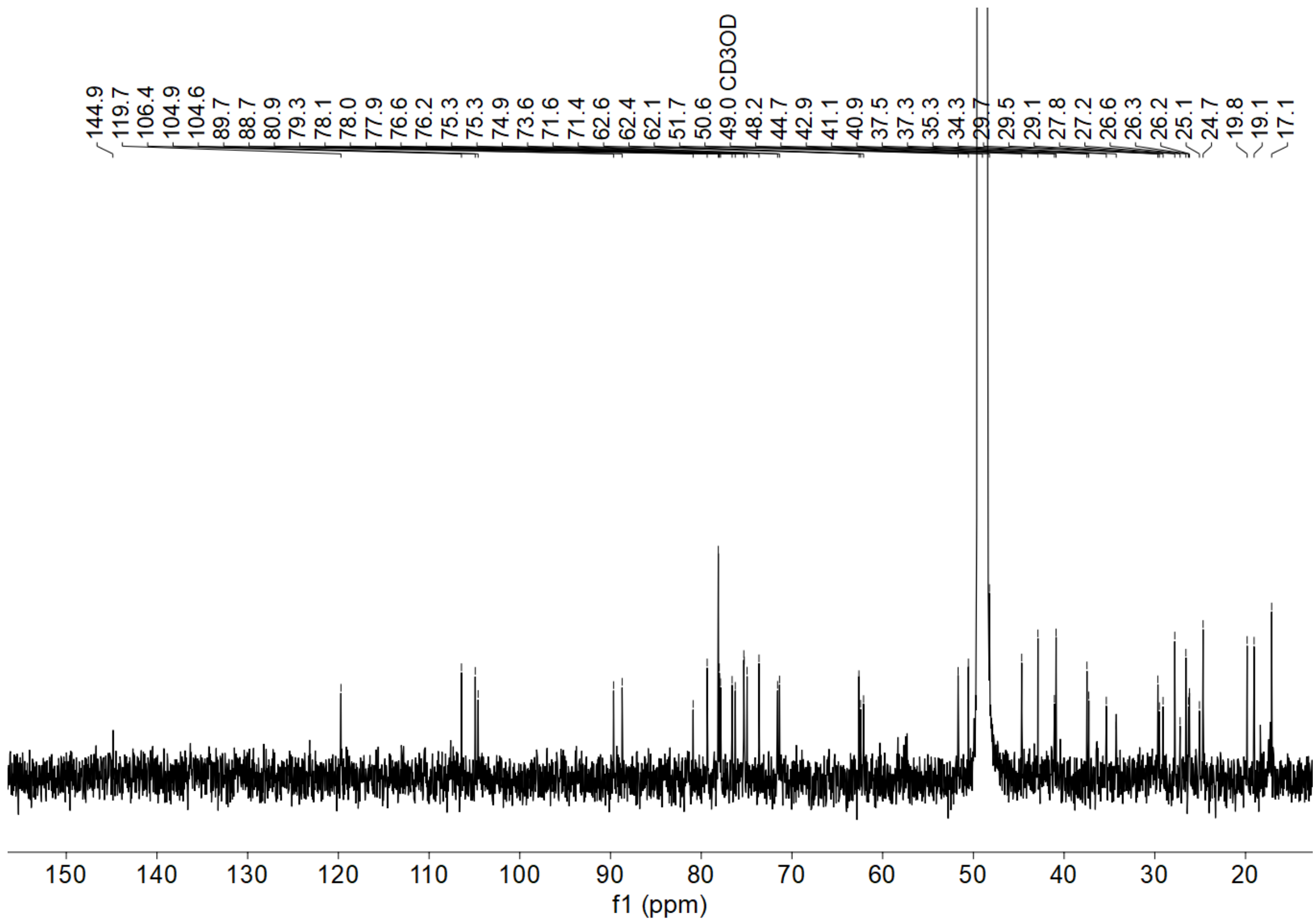

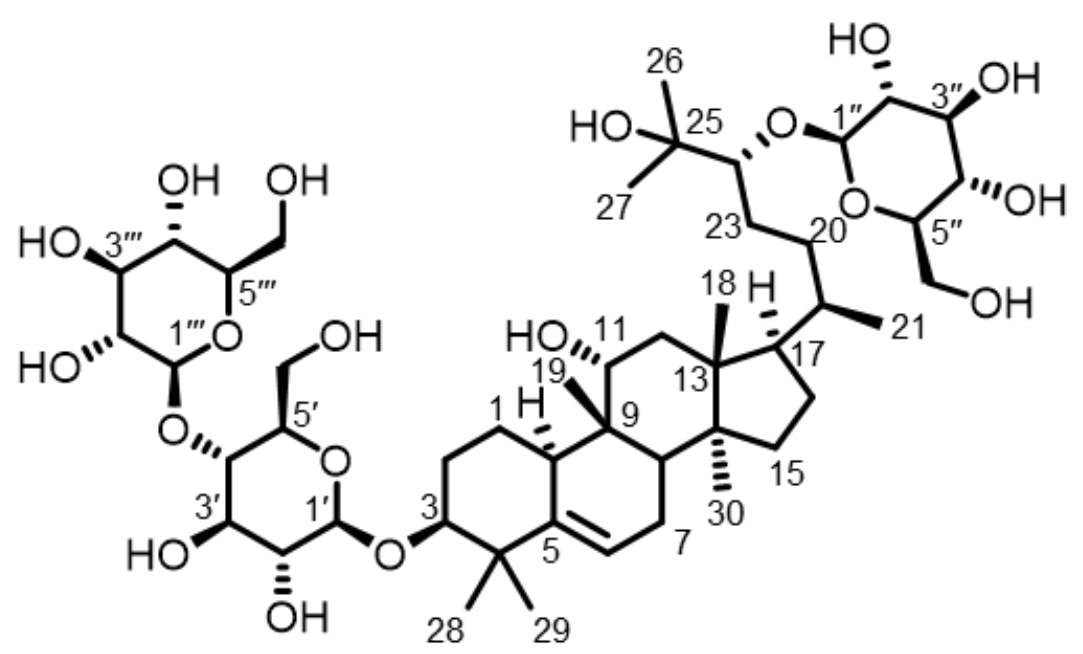

**Figure S14. $^{13}$C (125 MHz, methanol-$d_4$) NMR spectrum of mogroside IIE 4′-*O*-glucoside (5).** $\delta_C$ (ppm): 27.2 (C-1); 29.5 (C-2); 88.7 (C-3); 42.9 (C-4); 144.9 (C-5); 119.7 (C-6); 25.1 (C-7); 44.7 (C-8); 40.9 (C-9); 37.5 (C-10); 79.4 (C-11); 41.1 (C-12); 48.2 (C-13); 50.6 (C-14); 35.4 (C-15); 29.1 (C-16); 51.7 (C-17); 17.2 (C-18); 26.2 (C-19); 37.3 (C-20); 19.1 (C-21); 34.3 (C-22); 29.7 (C-23); 89.7 (C-24); 73.6 (C-25); 26.6 (C-26); 24.7 (C-27); 27.8 (C-28); 26.3 (C-29); 19.8 (C-30); 106.4 (C-1′); 75.3 (C-2′); 76.6(C-3′); 80.9 (C-4′); 78.1 (C-5′); 62.6 (C-6′); 104.9 (C-1″); 75.3 (C-2″); 78.0 (C-3″); 71.6 (C-4″); 78.1 (C-5″); 62.4 (C-6″); 104.6 (C-1‴); 75.0 (C-2‴); 77.9 (C-3‴); 71.4 (C-4‴); 76.3 (C-5‴); 62.1 (C-6‴).

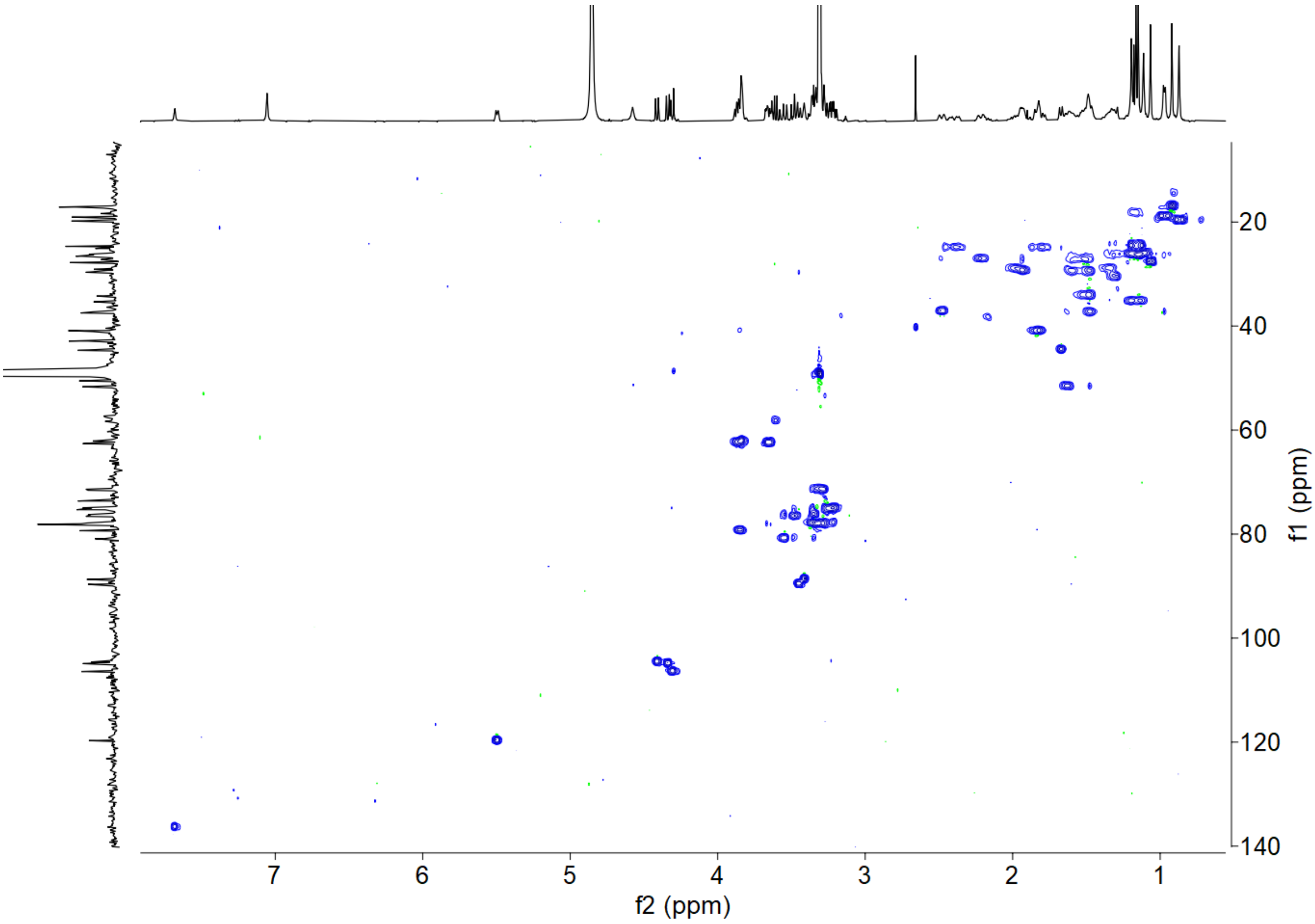


**Figure S15. HSQC spectrum of mogroside IIE 4′-*O*-glucoside (5).**

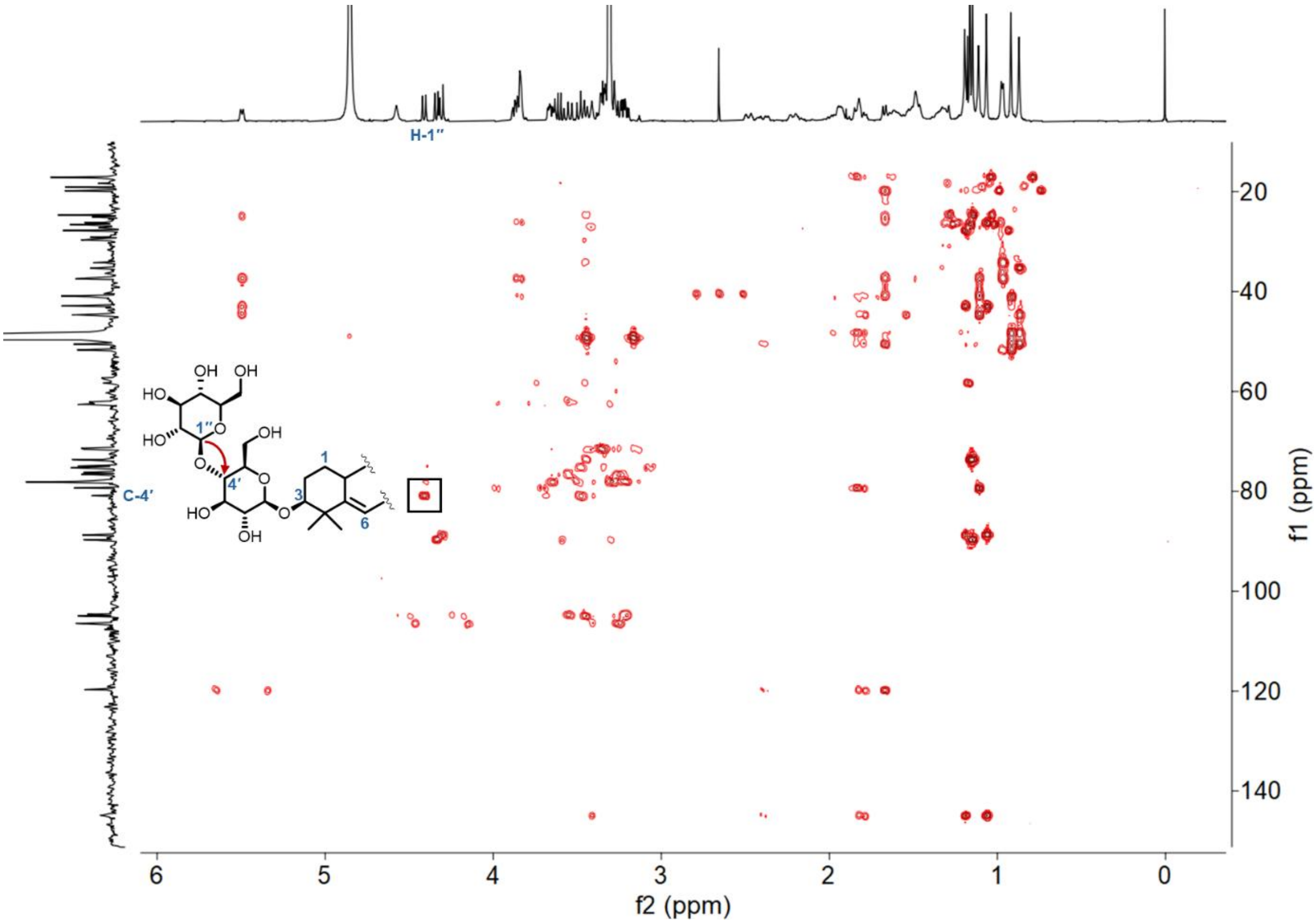


**Figure S16. HMBC spectrum of mogroside IIE 4′-*O*-glucoside (5).**

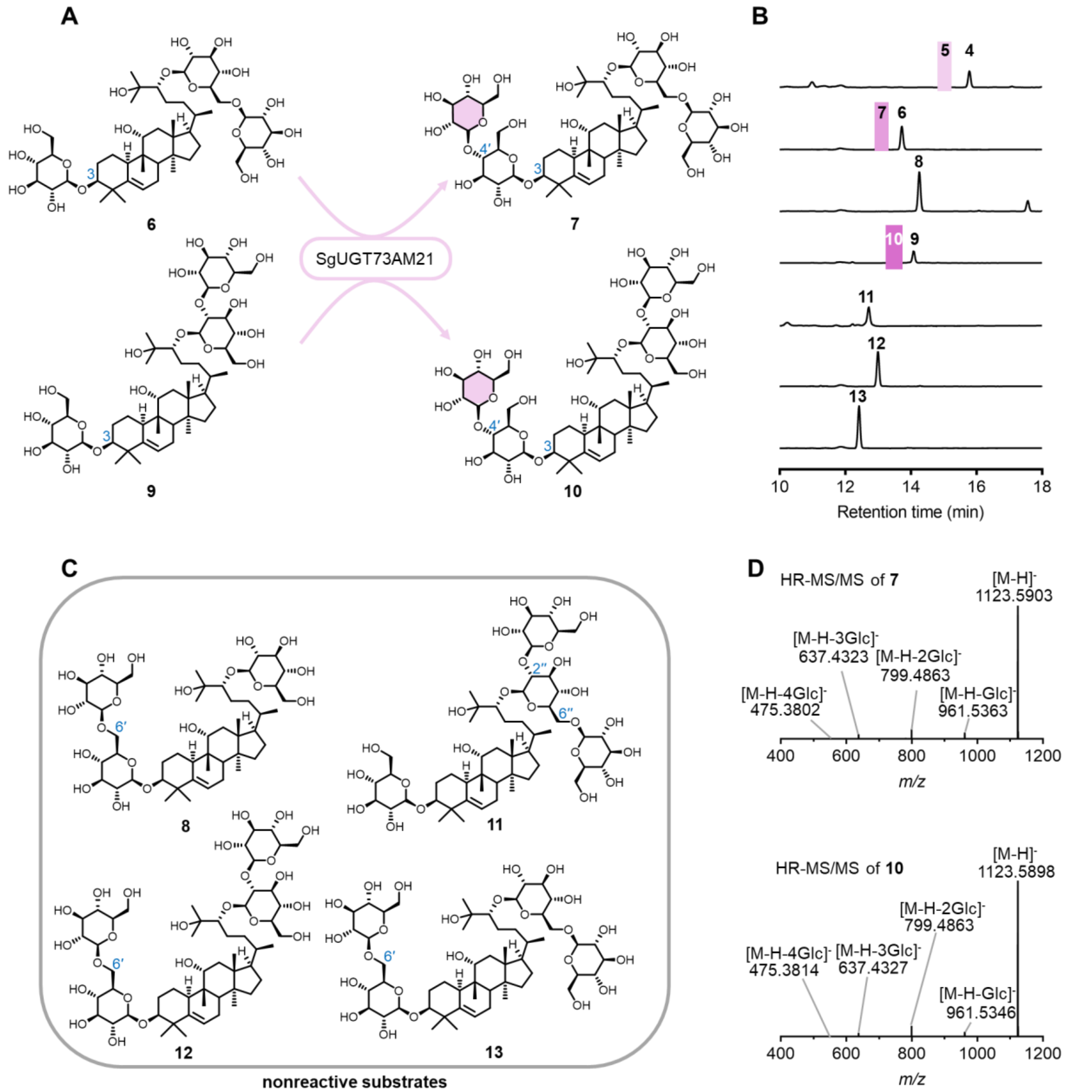


**Figure S17. Substrate spectrum of SgUGT73AM21.** (**A**) Proposed glycosylation reactions of SgUGT73AM21 towards **6** and **9**. (**B**) HPLC chromatograms showing the *in vitro* assay of SgUGT73AM21 toward **4**, **6**, **8**, **9**, **11**-**13**. (C) Chemical structures of the substrates **8**, **11~13** not recognized by SgUGT73AM21. (**D**) HR-MS/MS spectra of glycosylated products **7** and **10**.

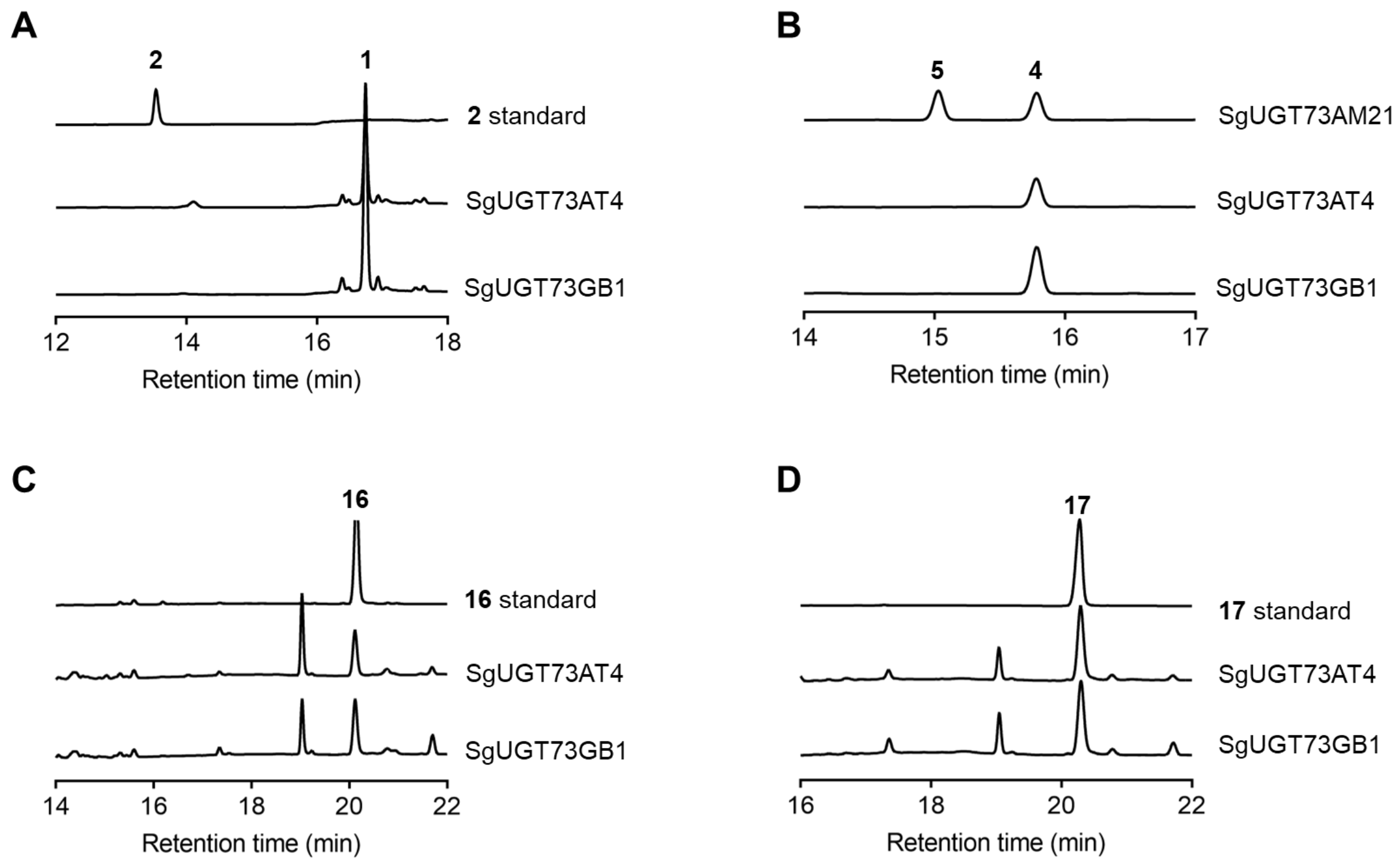


**Figure S18. Functional characterization of SgUGT73AT4 and SgUGT73GB1 towards substrates 1 (A), 4 (B), 16 (C) and 17 (D).** Neither of the enzymes mentioned above exhibit any activity toward these substrates.

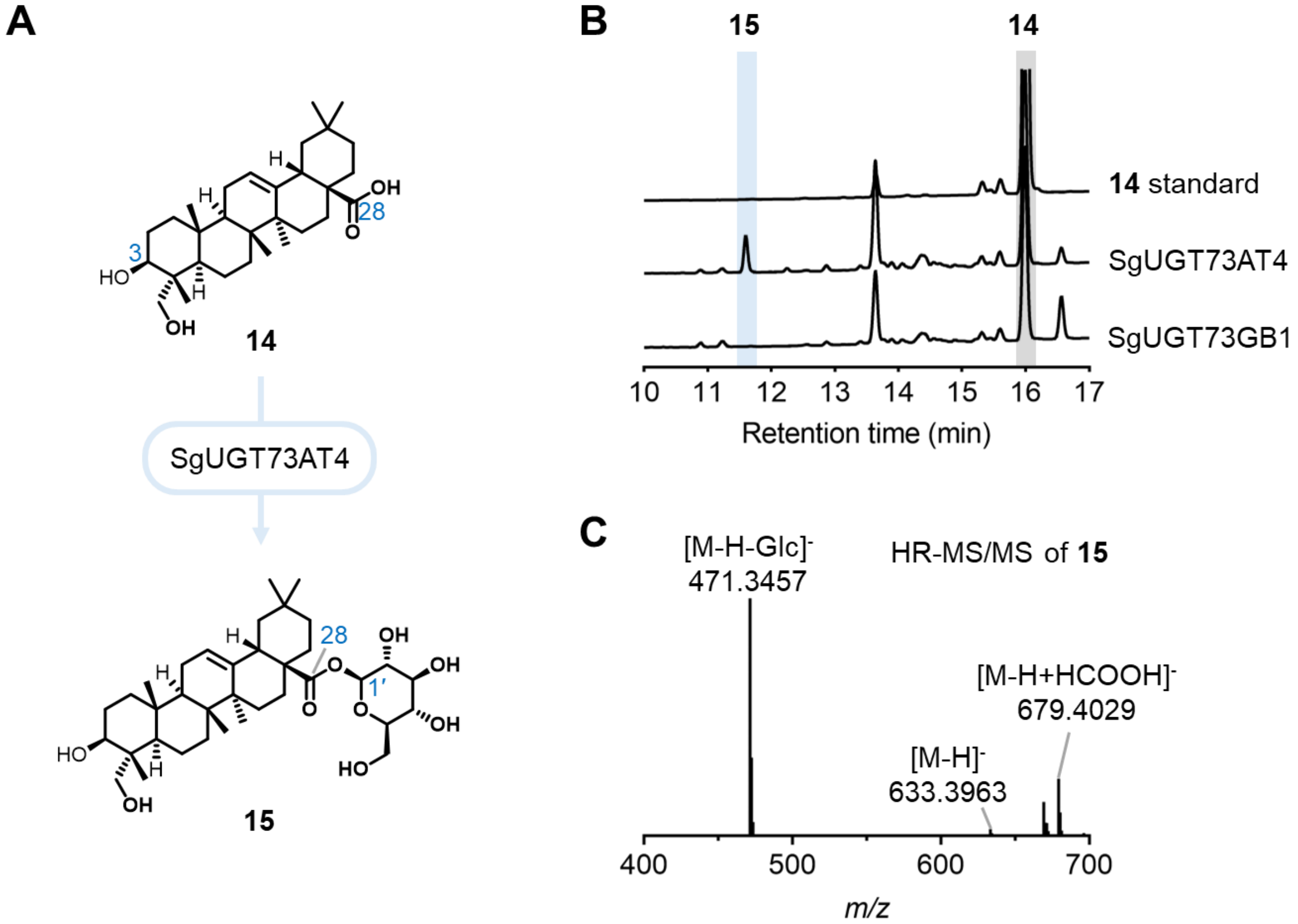


**Figure S19. Function of SgUGT73AT4.** (**A**) Characterized glycosylation reactions of SgUGT73AT4 towards **14**. (**B**) HPLC chromatograms showing the *in vitro* assay of SgUGT73AT4 and SgUGT73GB1 toward **14**. (C) HR-MS/MS spectra of the glycosylated products **15**.

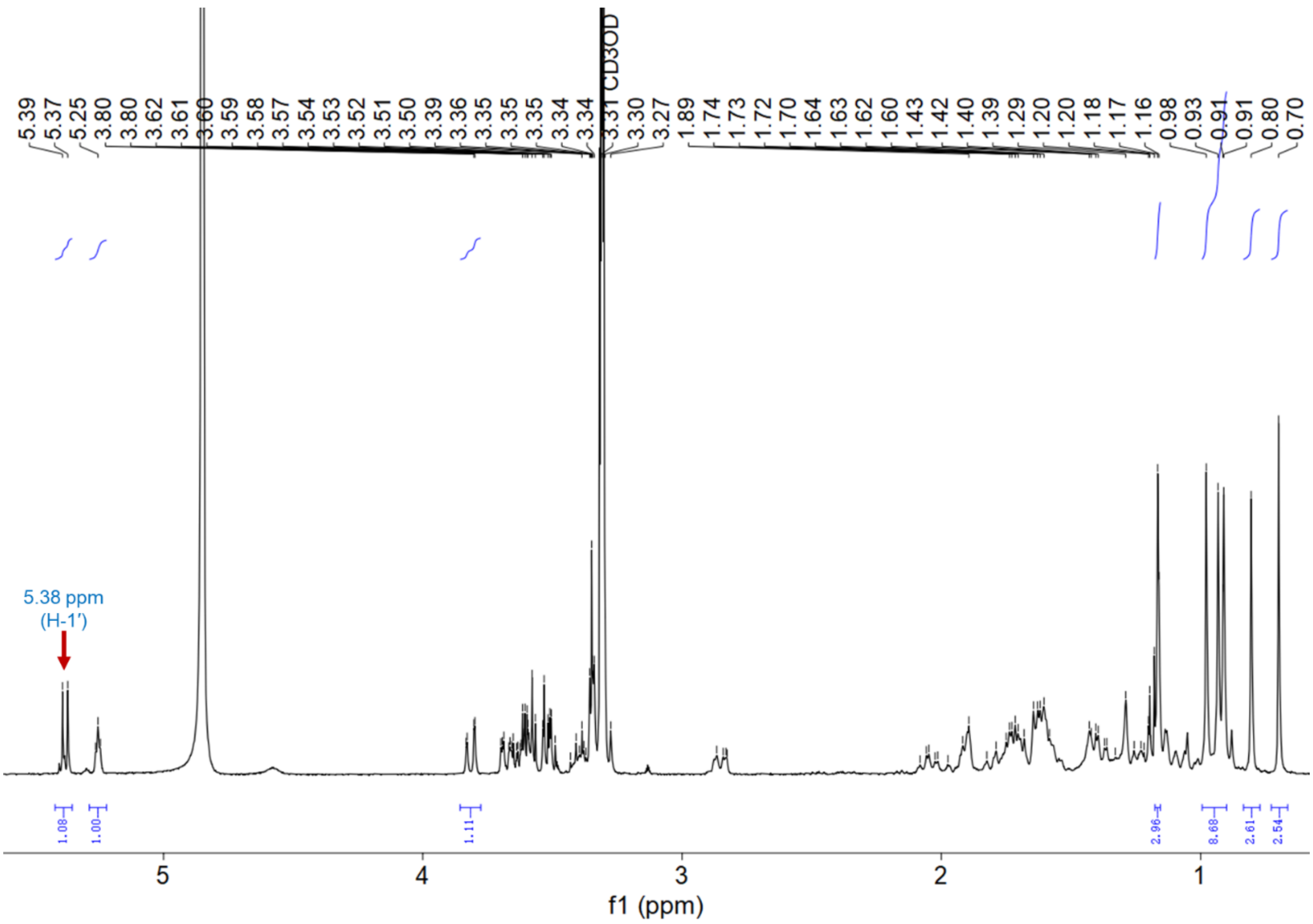



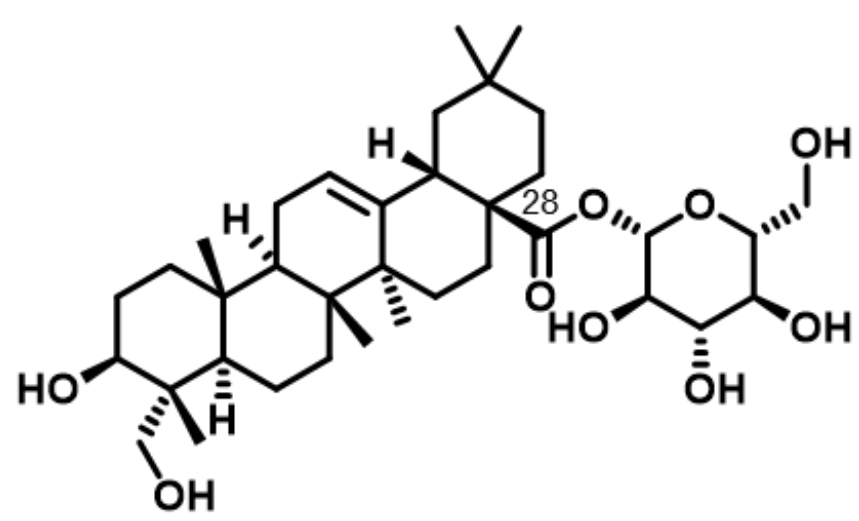



**Figure S20. $^1$H (400 MHz, methanol-$d_4$) NMR spectrum of hederagenin 28-*O*-glucoside (15).** The chemical shift of anomeric proton of C28-*O*-β-D-glucose and C3-*O*-β-D-glucose is 5.34 and 4.39 ppm in methanol-$d_4$, respectively.

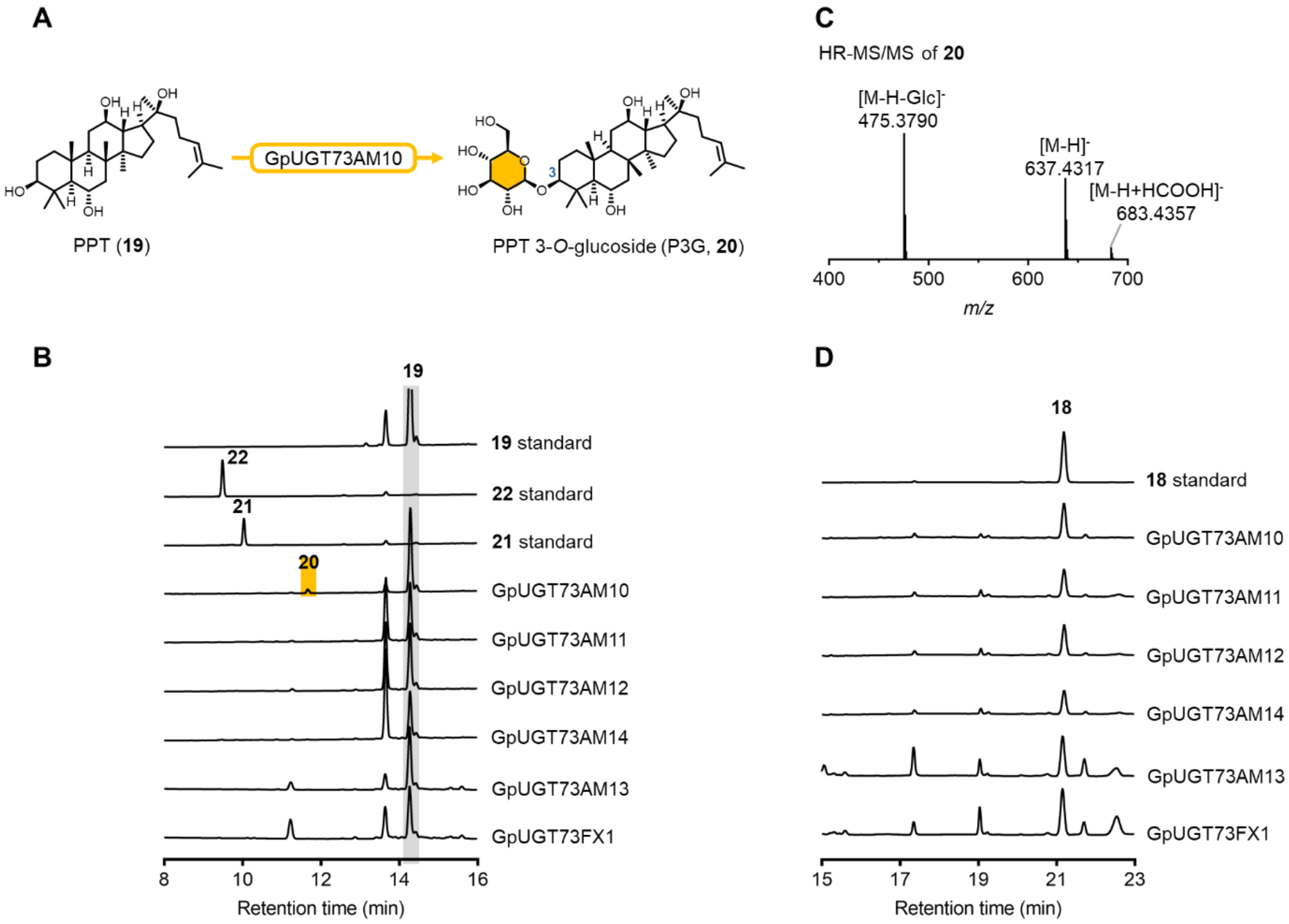


**Figure S21. Function of GpUGT73AM10.** (**A**) Identified glycosylation reaction of GpUGT73AM10 towards PPT (**19**). (**B**) HPLC chromatograms depicting the *in vitro* assay of GpUGT73AMs and GpUGT73FX1 toward **19**. (C) HR-MS/MS spectra of the glycosylated product **20**. (**D**) HPLC chromatograms revealed that GpUGT73s exhibited no activity against PPD (**18**) *in vitro*.

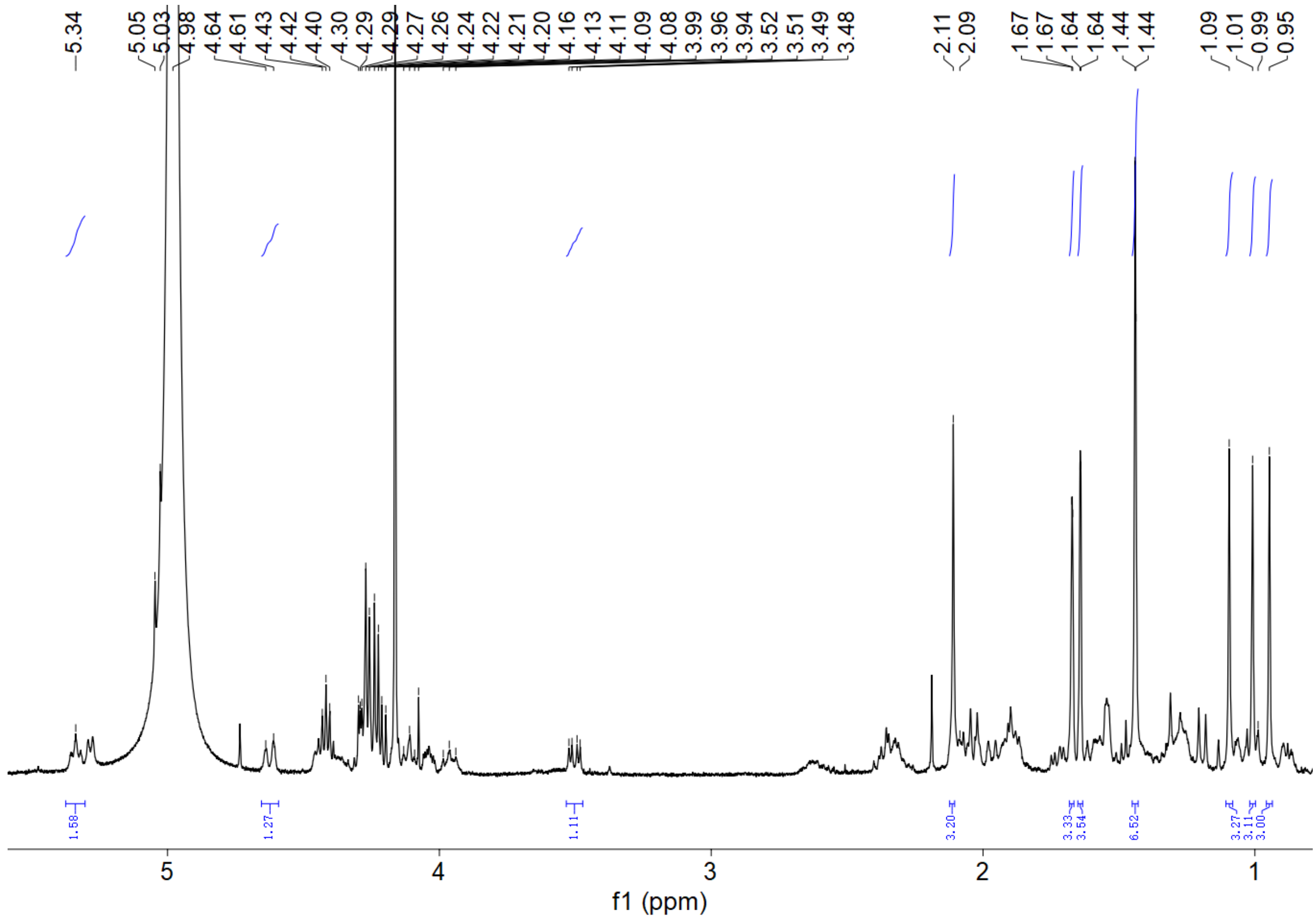


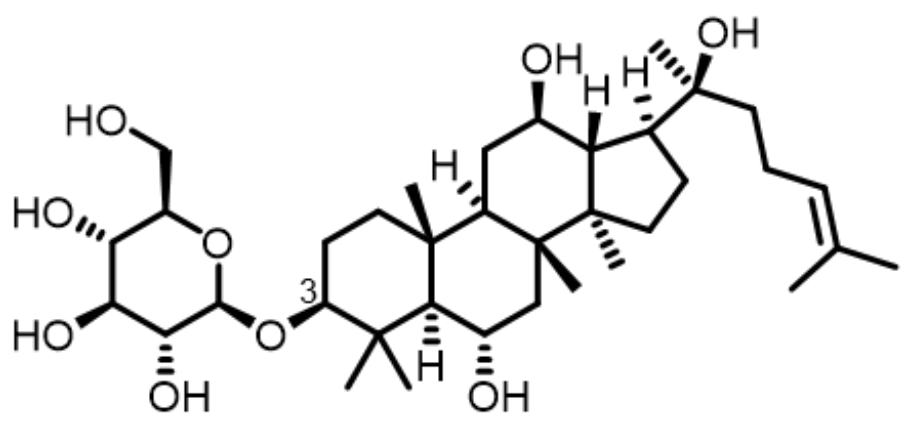


**Figure S22. $^{1}$H (400 MHz, pyridine-*d*5) NMR spectrum of protopanaxatriol 3-*O*-glucoside (20).**

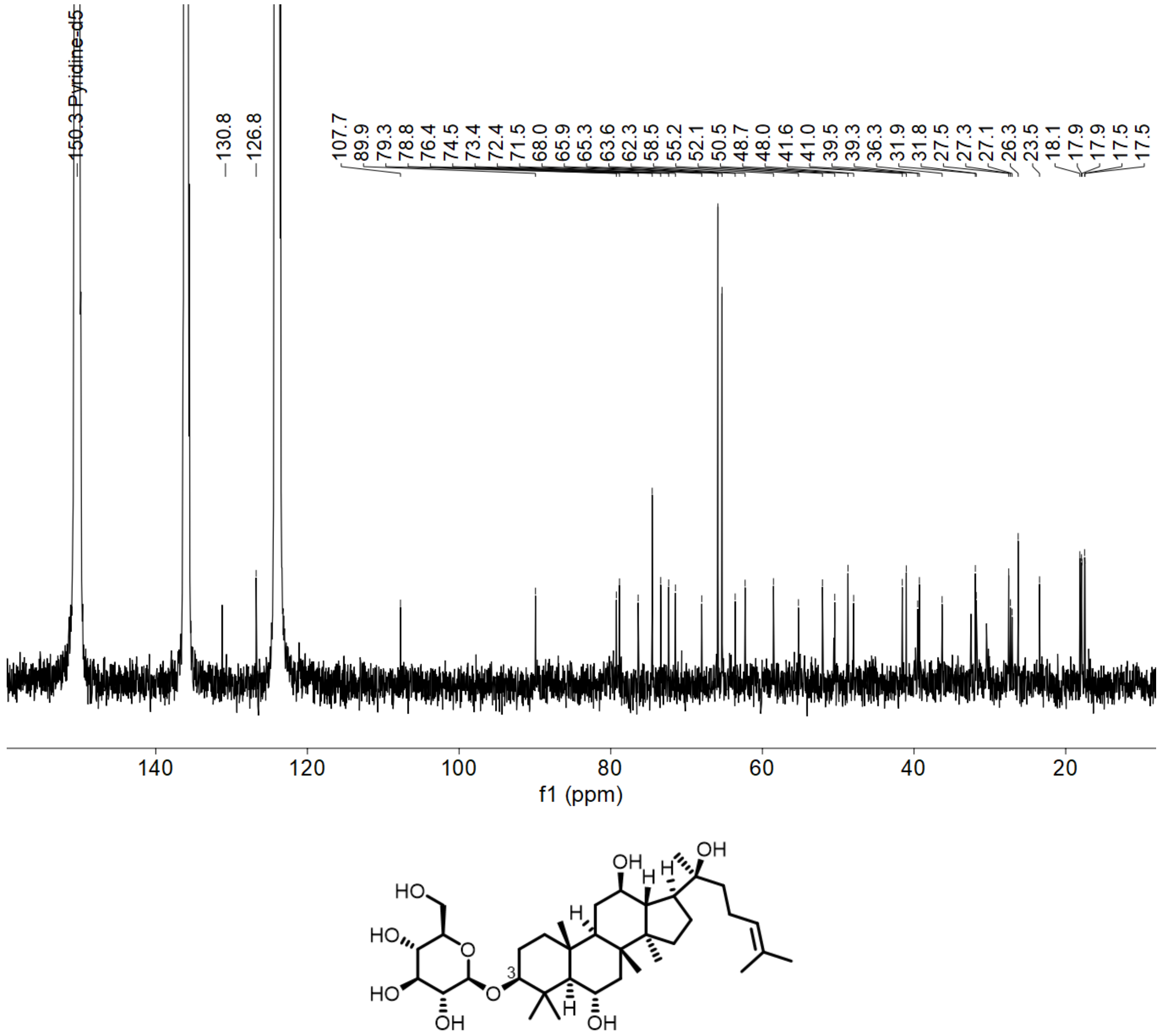


**Figure S23.** **$^{13}$C (125 MHz, pyridine-$d_5$) NMR spectrum of protopanaxatriol 3-*O*-glucoside (20).**

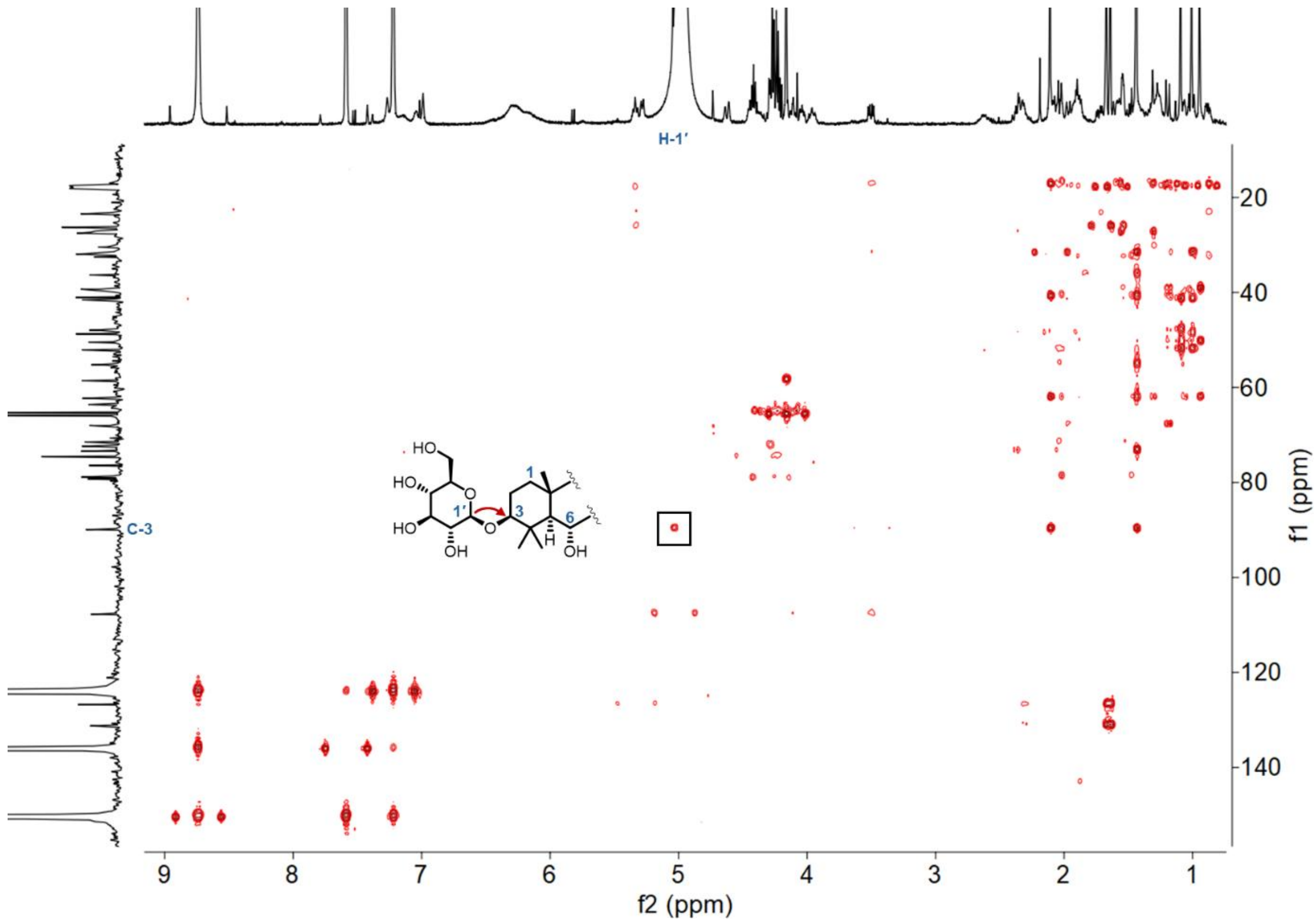


**Figure S24. HMBC spectrum of protopanaxatriol 3-*O*-glucoside (20).**

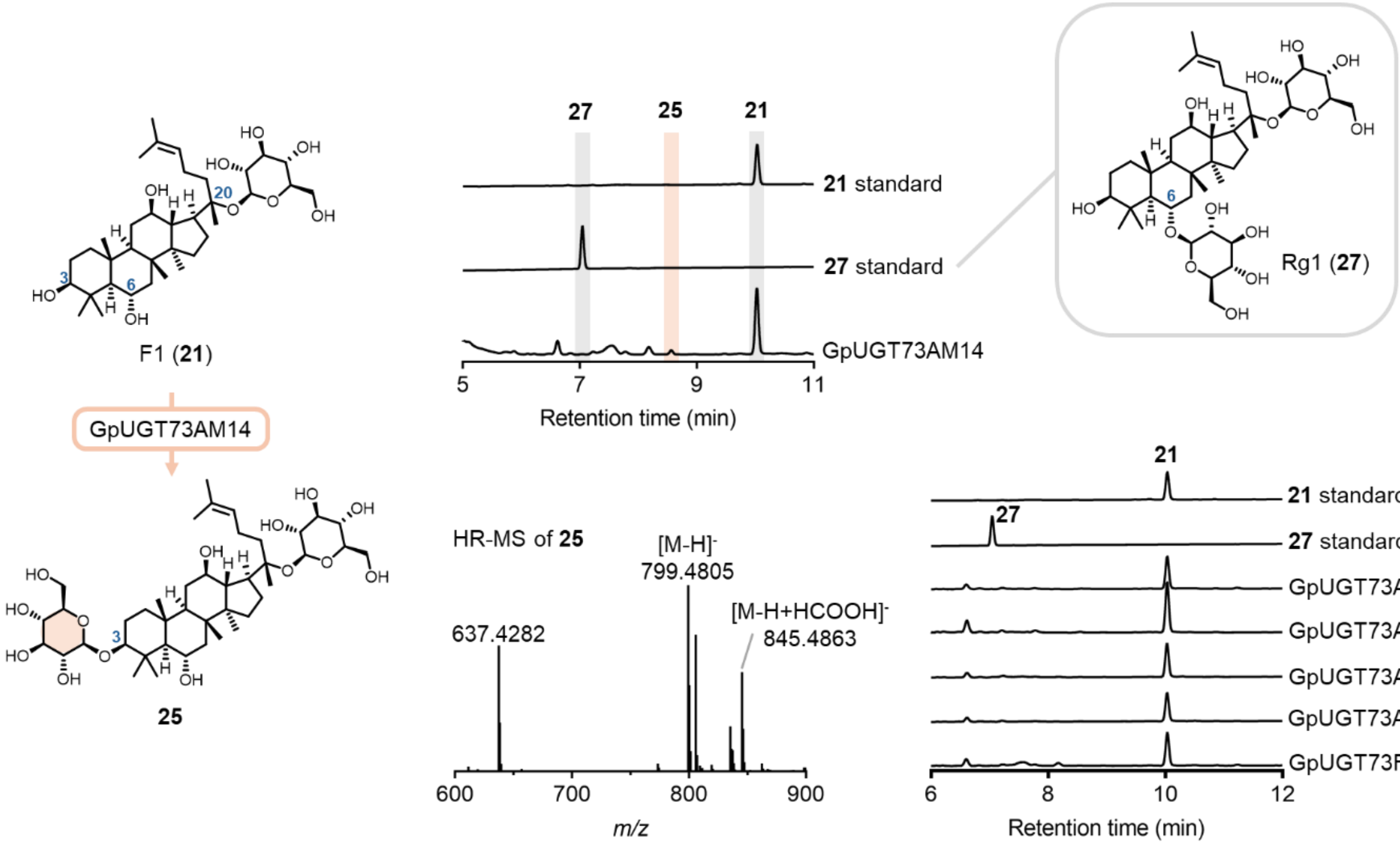


**Figure S25. GpUGT73AM14 as ginsenoside F1 3-*O*-glycosyltransferase.** The versatile GpUGT73AM14 possesses ginsenoside F1 (**21**) 3-*O*-glycosyltransferase activity, whereas the remaining GpUGT73s were not active.

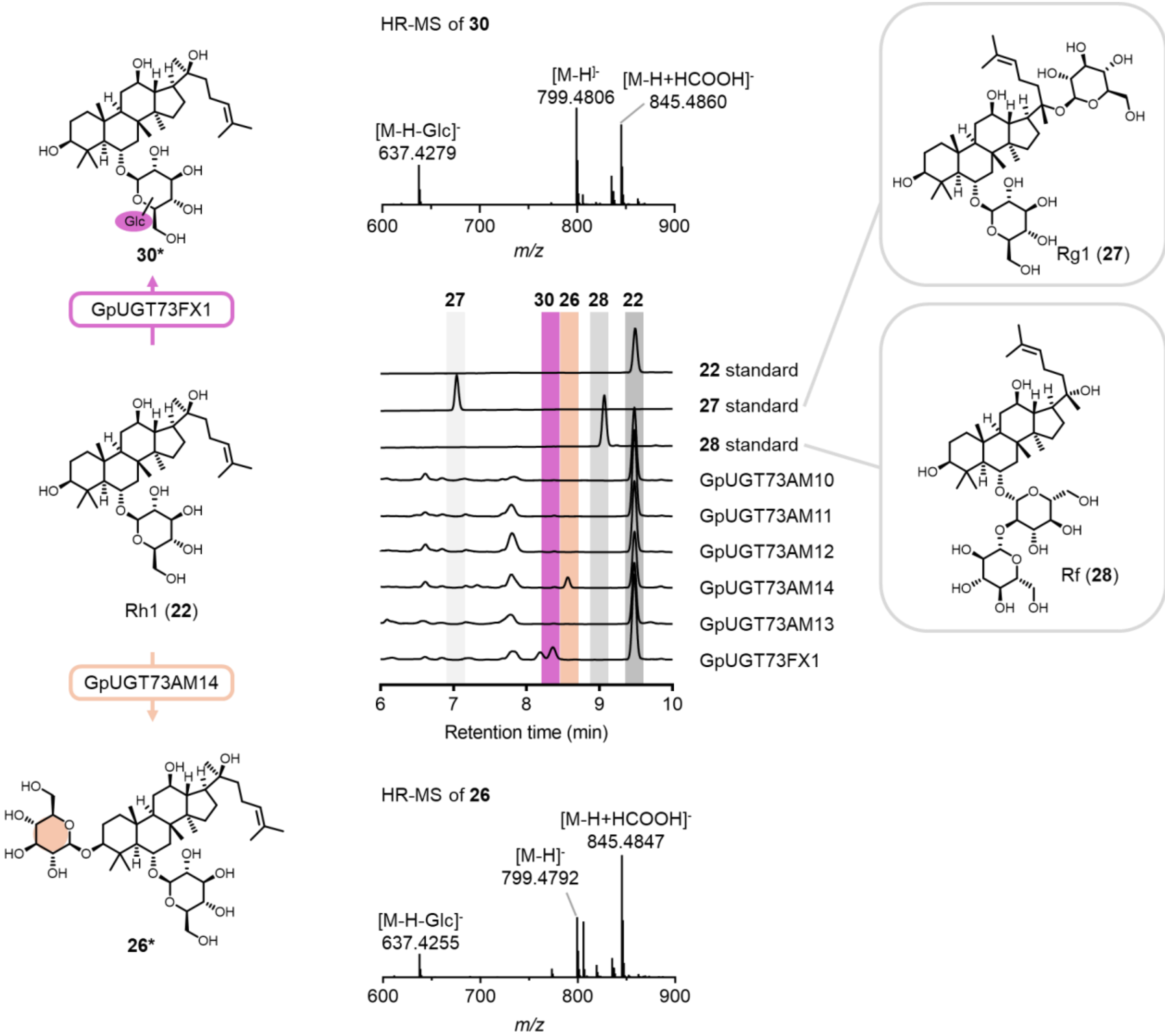


**Figure S26. *In vitro* biochemical characterization of GpUGT73FX1 and GpUGT73AM14 towards ginsenoside Rh1.** Unfortunately, neither product could be purified enough for NMR confirmation. The activity of GpUGT73AM14 as ginsenoside F1 3-*O*-glycosyltransferase suggested product **26** might be ginsenoside Rh1 3-*O*-glucoside, while product **30**, based on comparison with standards, was putatively identified as di-glucoside with an extended sugar chain at C-6 position.

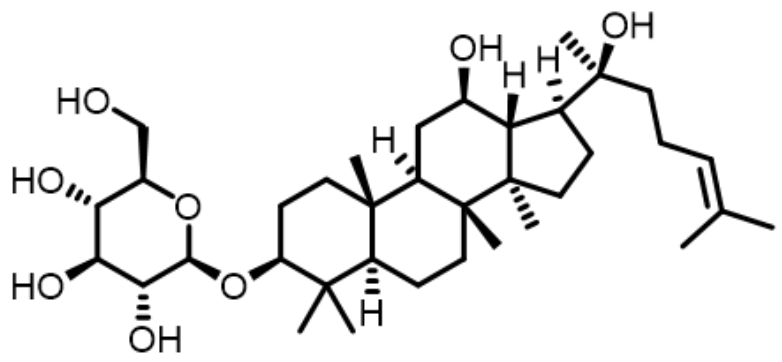


Rh2 (**29**)

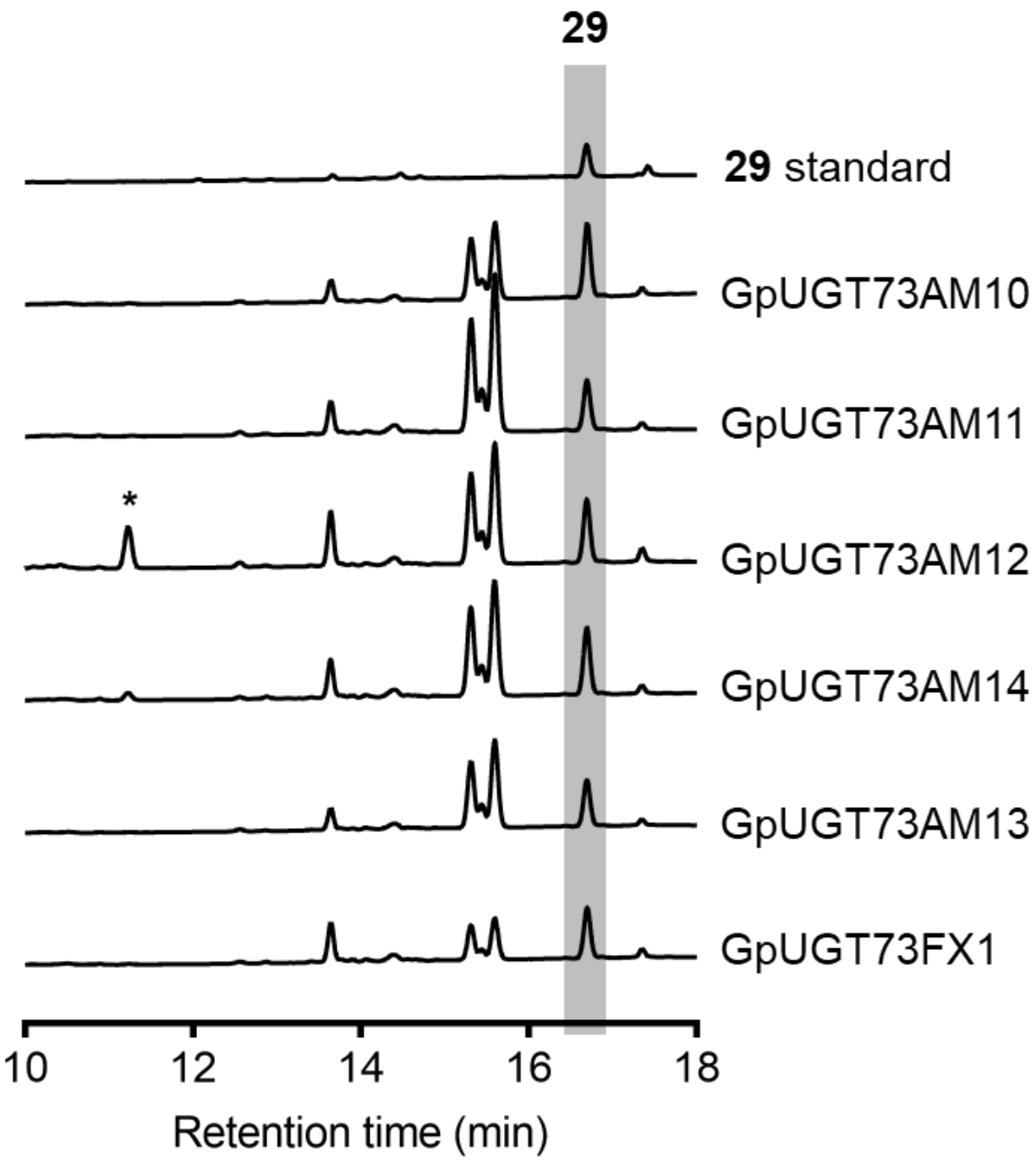


**Figure S27. *In vitro* assay of GpUGT73AMs and GpUGT73FX1 toward ginsenoside Rh2.** None of the UGT73s exhibit glycosylation activity against the ginsenoside Rh2 (**29)**. The asterisk (*) indicates impurity.

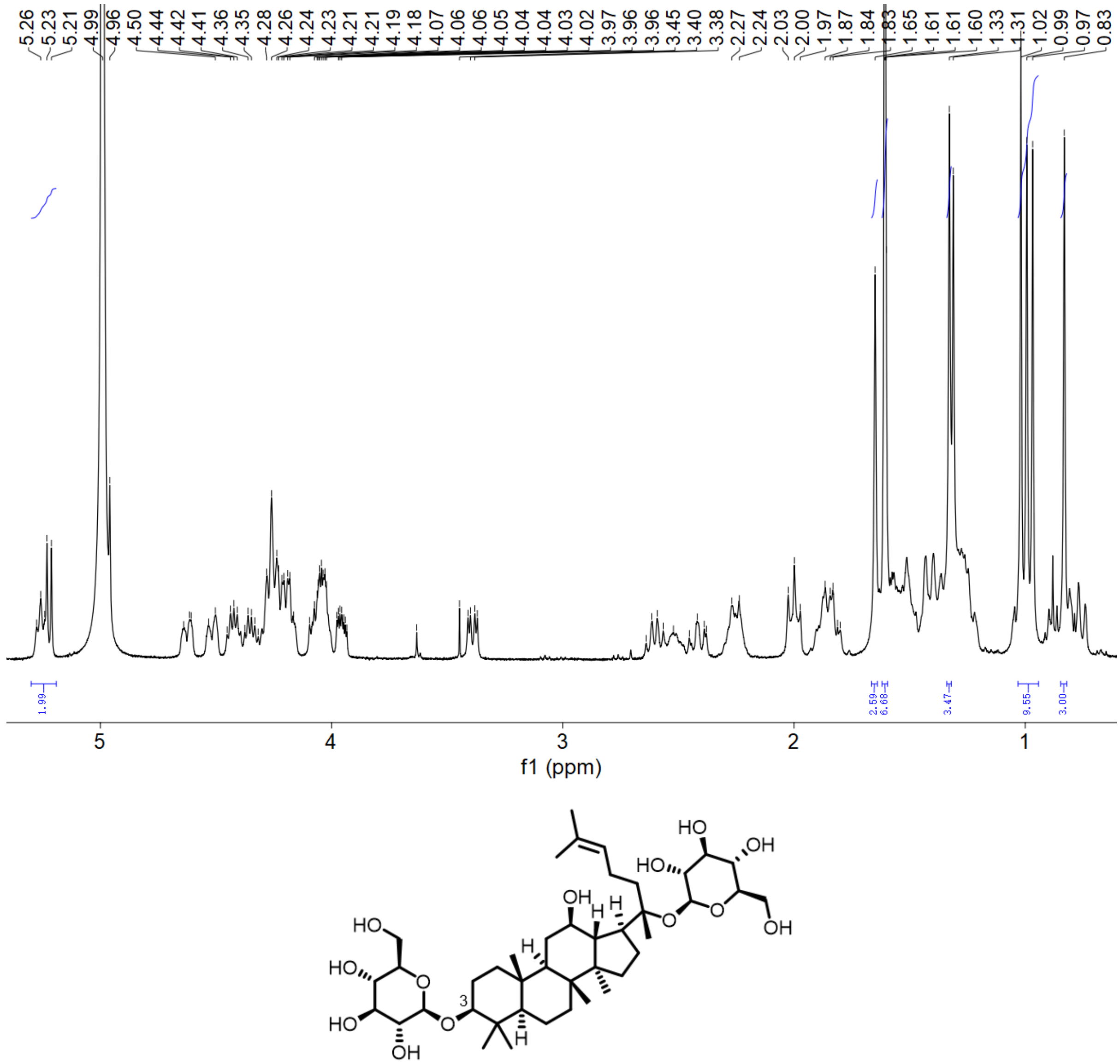

**Figure S28. $^{1}$H (400 MHz, pyridine-*d*$_5$) NMR spectrum of ginsenoside F2 (24).**

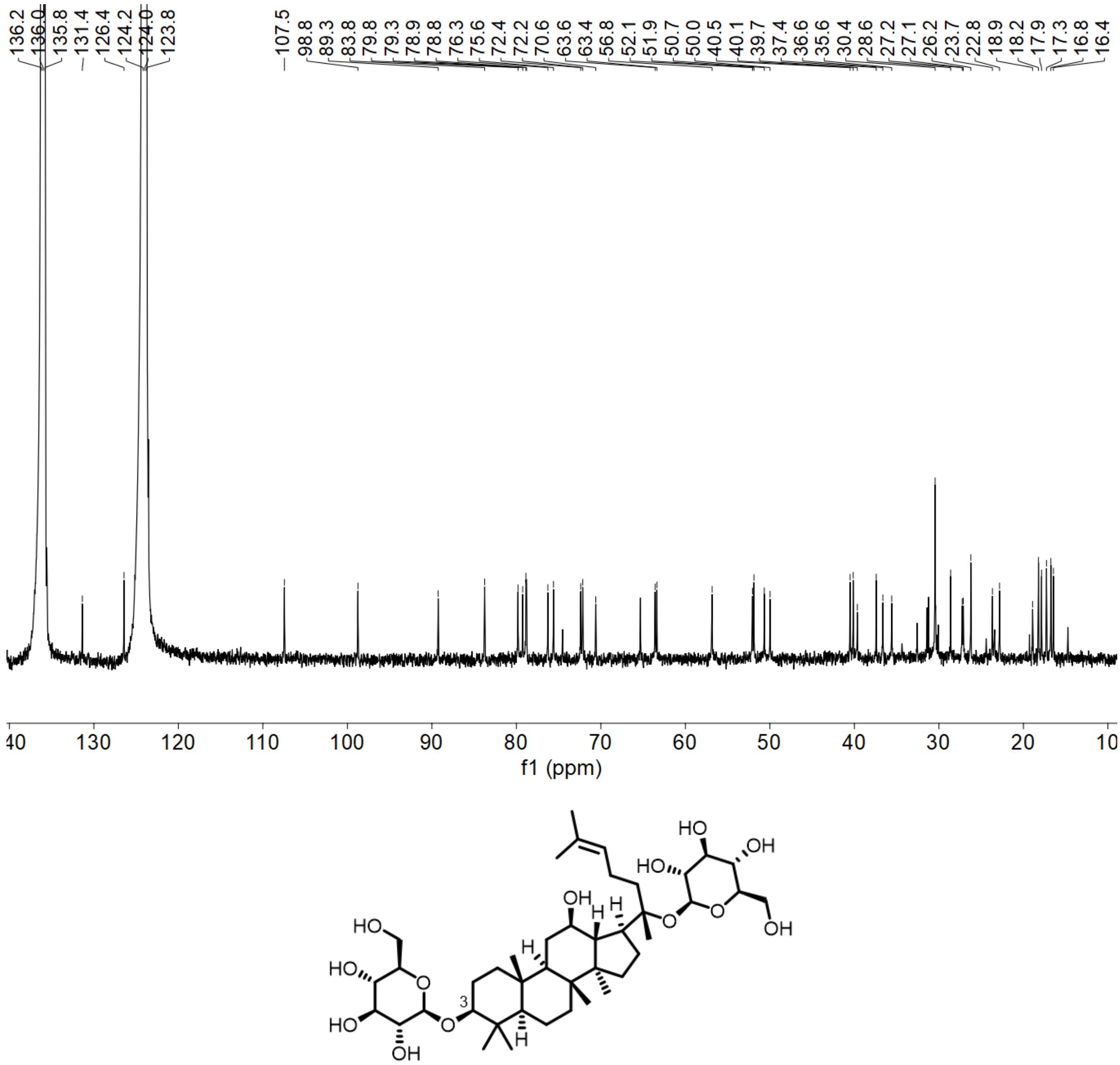


**Figure S29. $^{13}$C (125 MHz, pyridine-$d_5$) NMR spectrum of ginsenoside F2 (24).**

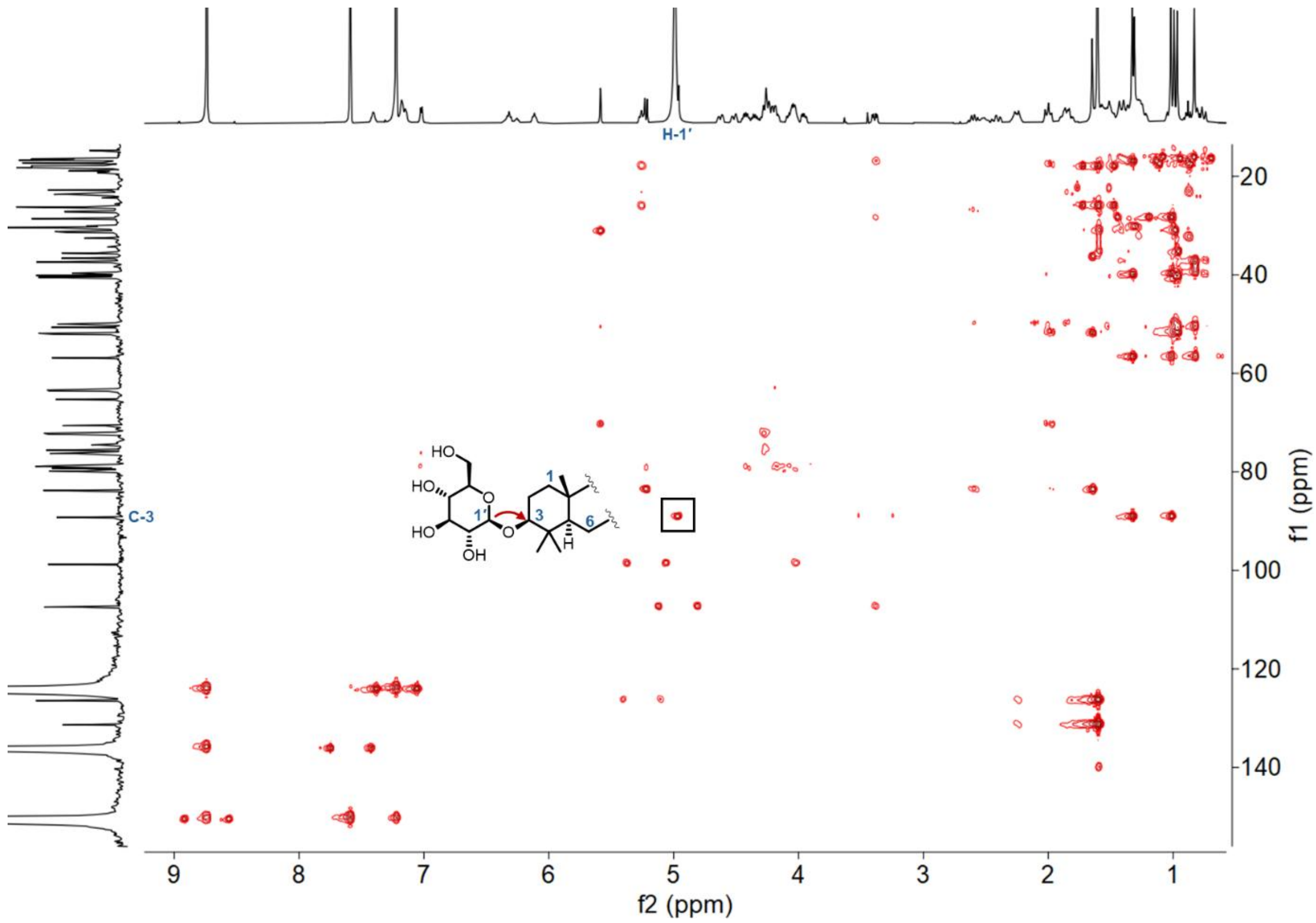


**Figure S30. HMBC spectrum of ginsenoside F2 (24).**

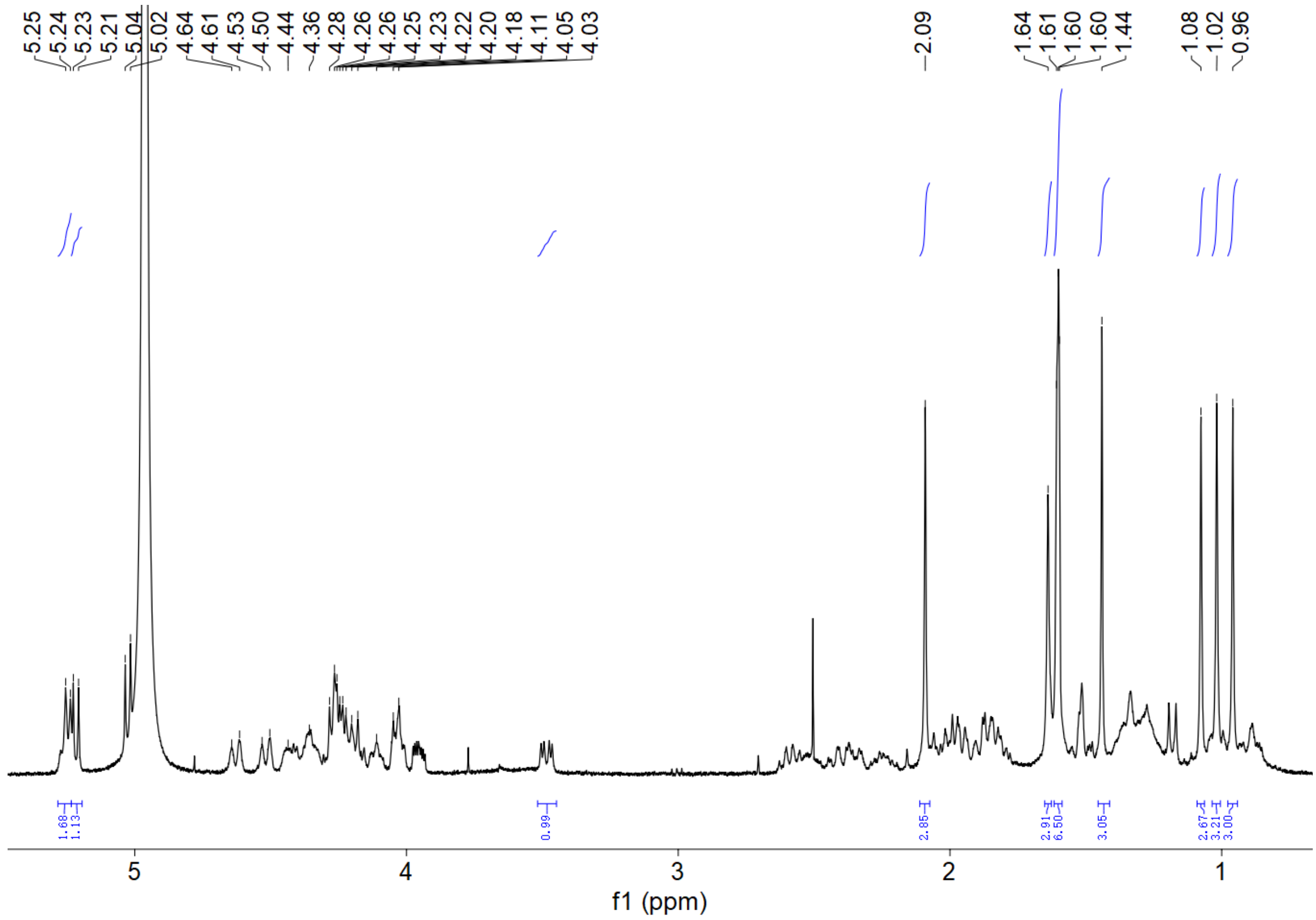


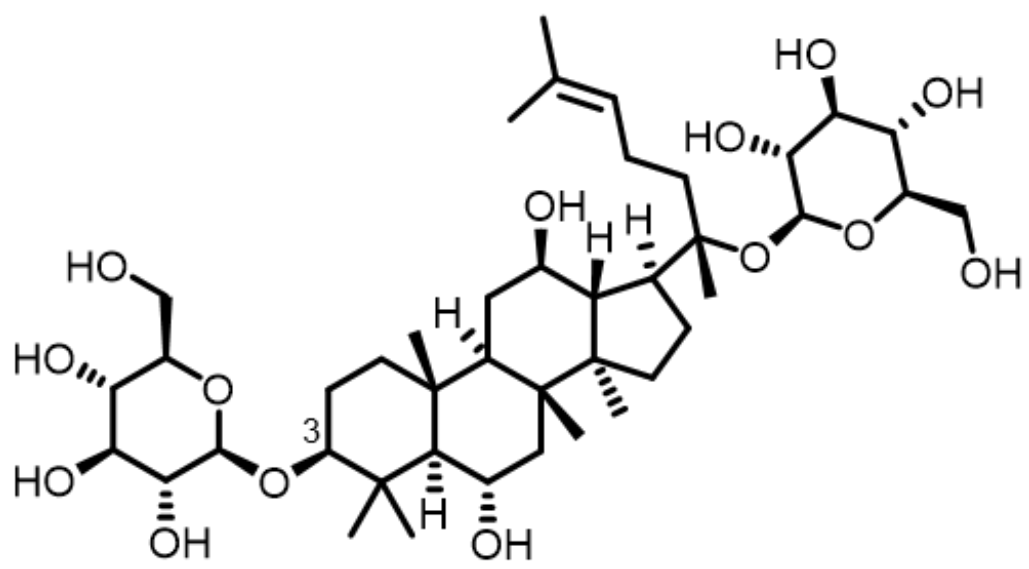


**Figure S31.** **$^{1}$H (400 MHz, pyridine-*d*$_5$) NMR spectrum of ginsenoside F1 3-*O*-glucoside (25).**

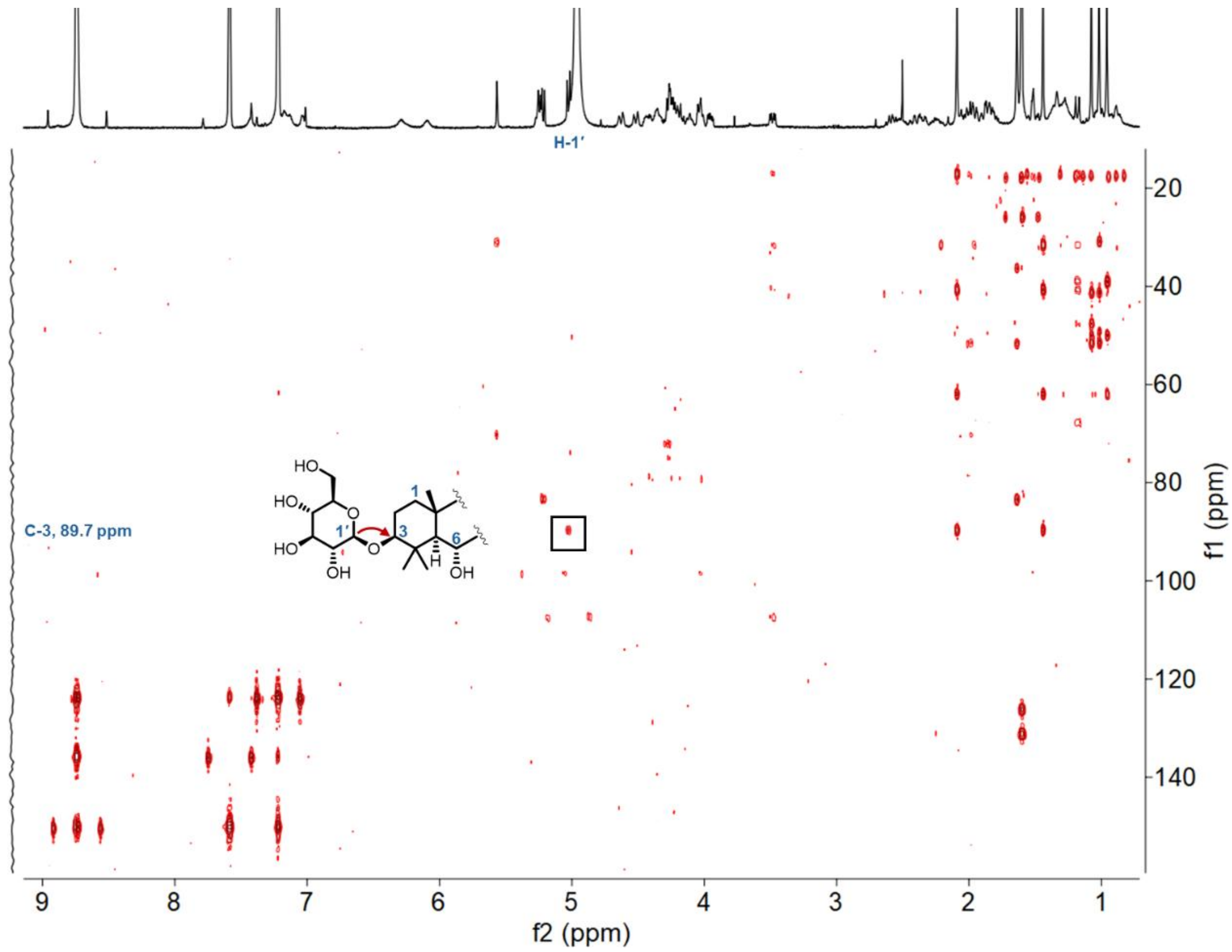


**Figure S32. HMBC spectrum of ginsenoside F1 3-*O*-glucoside (25).**

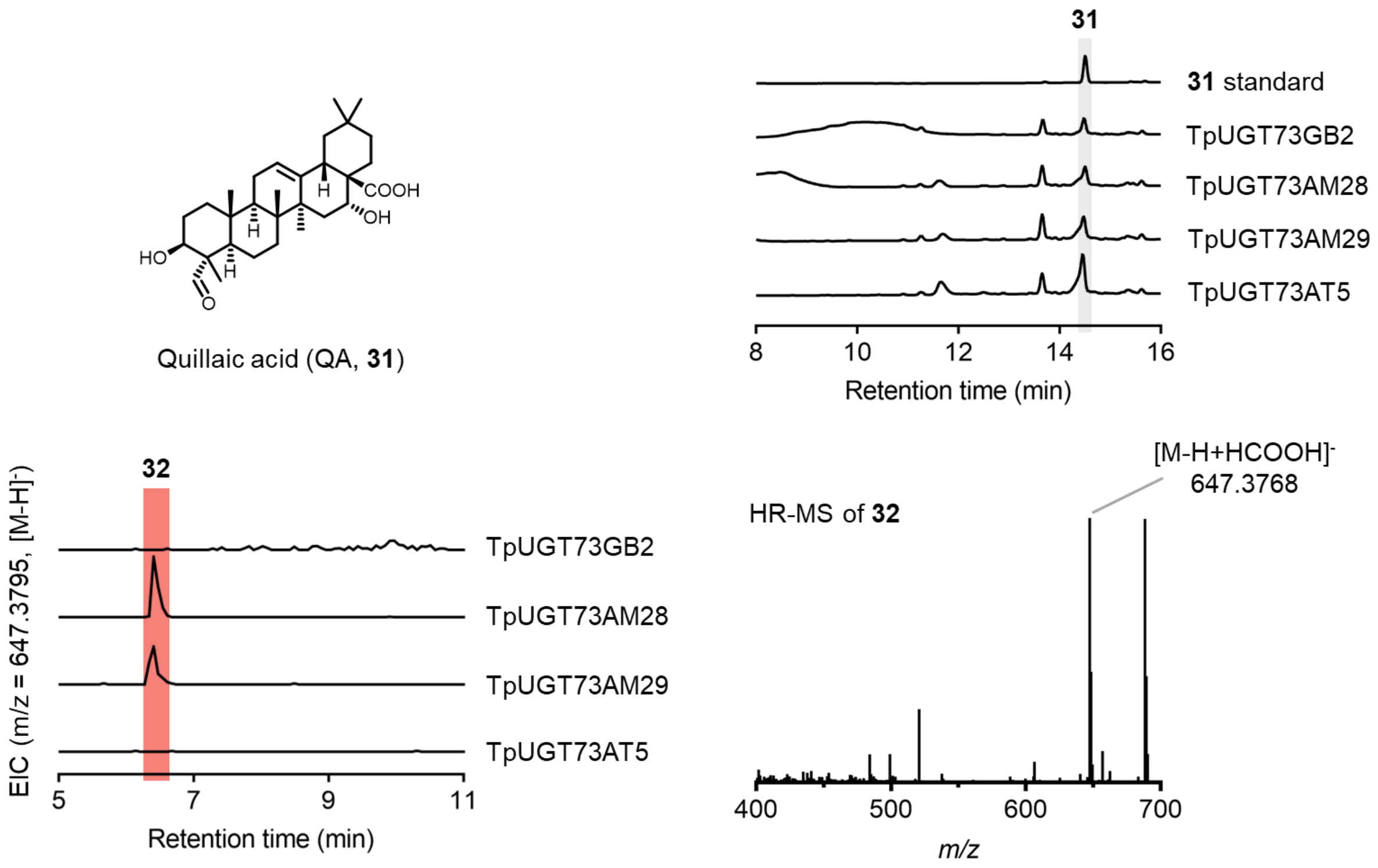


**Figure S33. *In vitro* biochemical assay of TpUGT73s.** TpUGT73AM28 and TpUGT73AM29 exhibited slight glycosylation towards QA (**31**), which could only be detected by LC-MS.

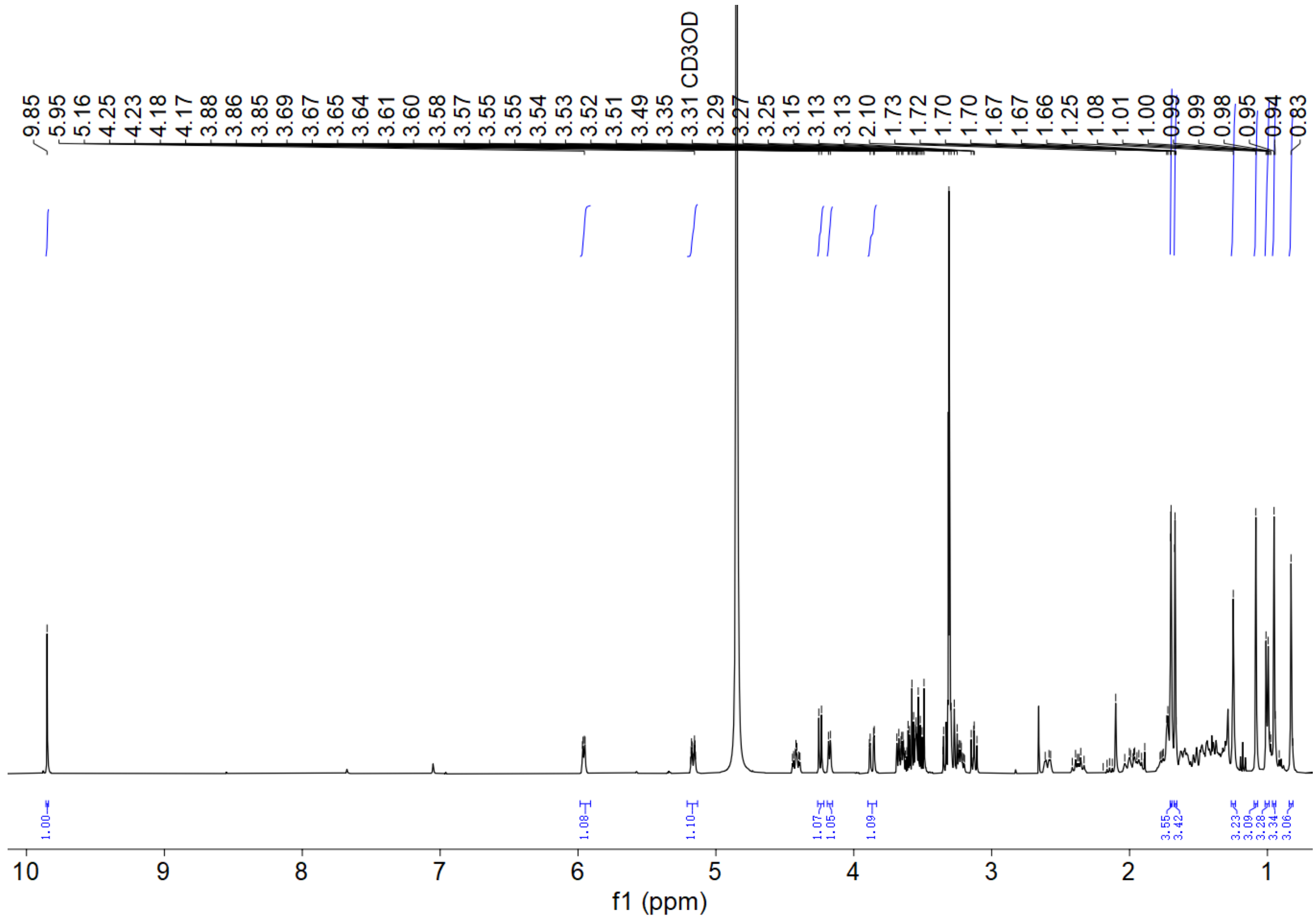

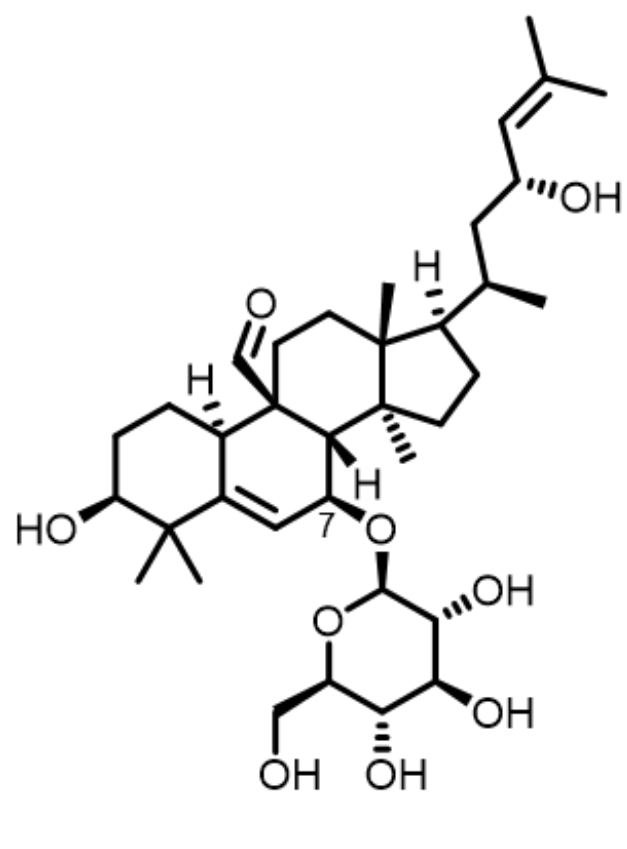

**Figure S34.** **$^{1}$H (400 MHz, methanol-*d*$_4$) NMR spectrum of momordicine IV (34).**

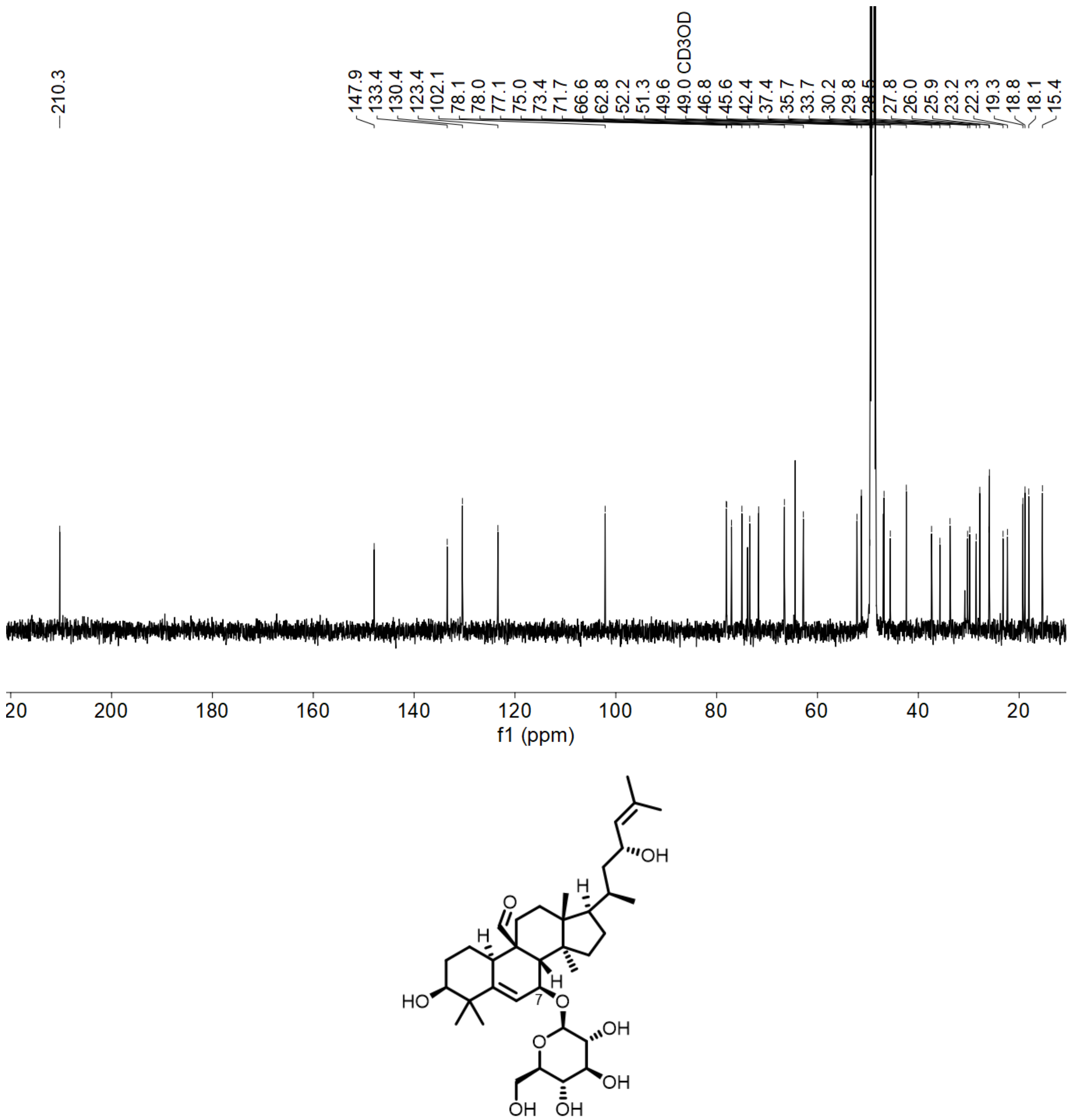


**Figure S35. $^{13}$C (125 MHz, methanol-*d*₄) NMR spectrum of momordicine IV (34).**

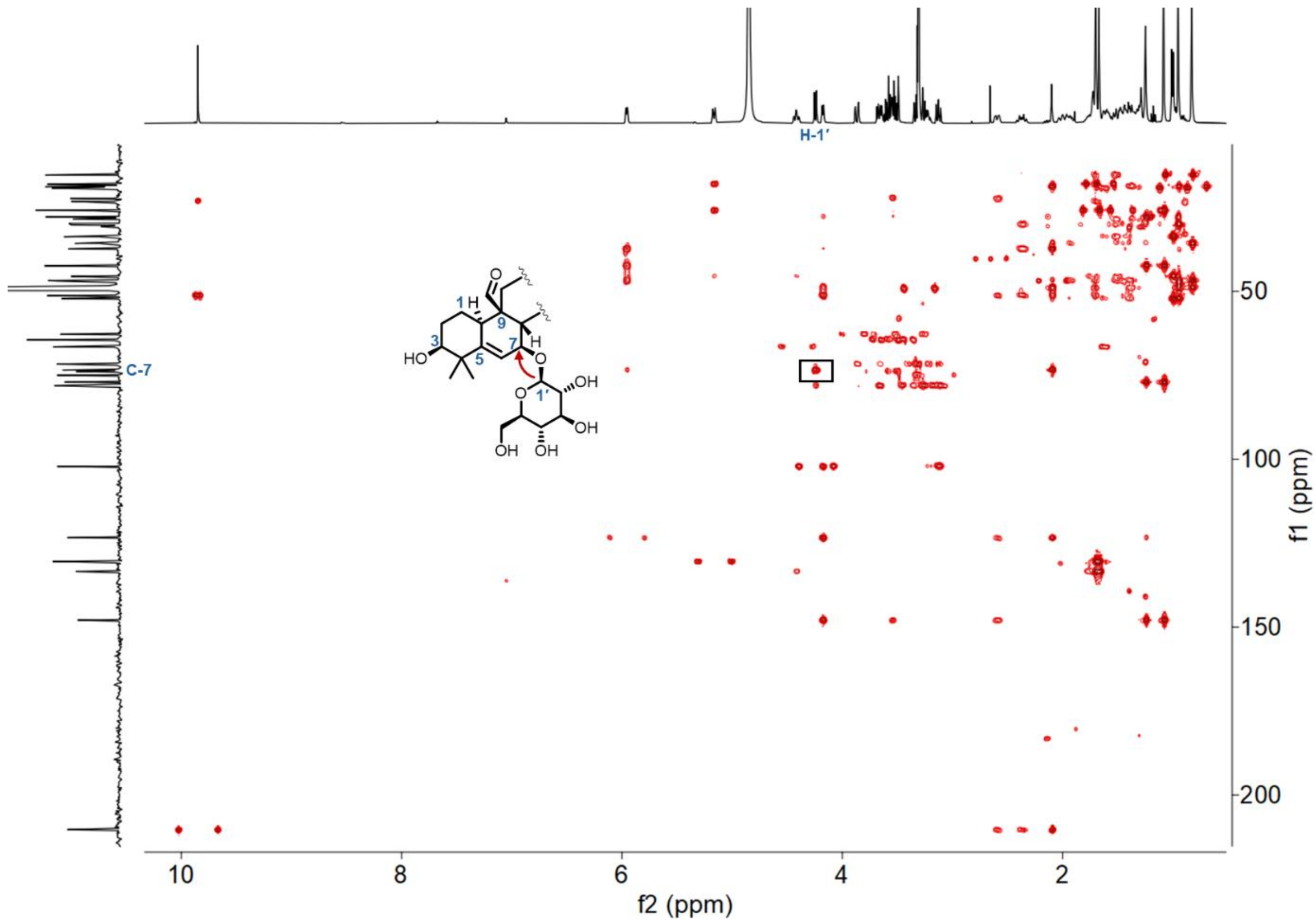


**Figure S36. HMBC spectrum of momordicine IV (34).**

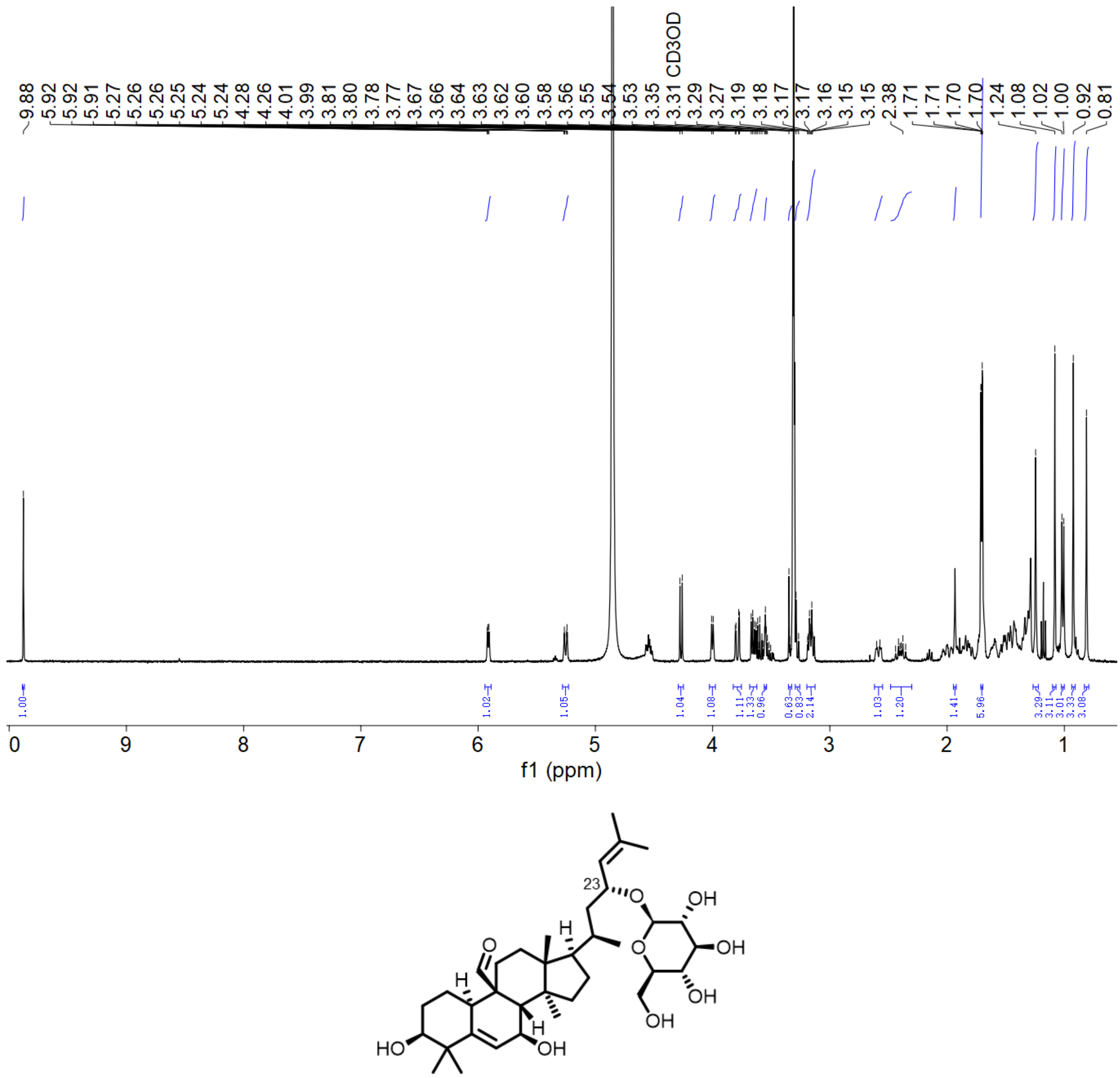


**Figure S37.** **$^{1}$H (400 MHz, methanol-*d*$_4$) NMR spectrum of momordicine II (35).**

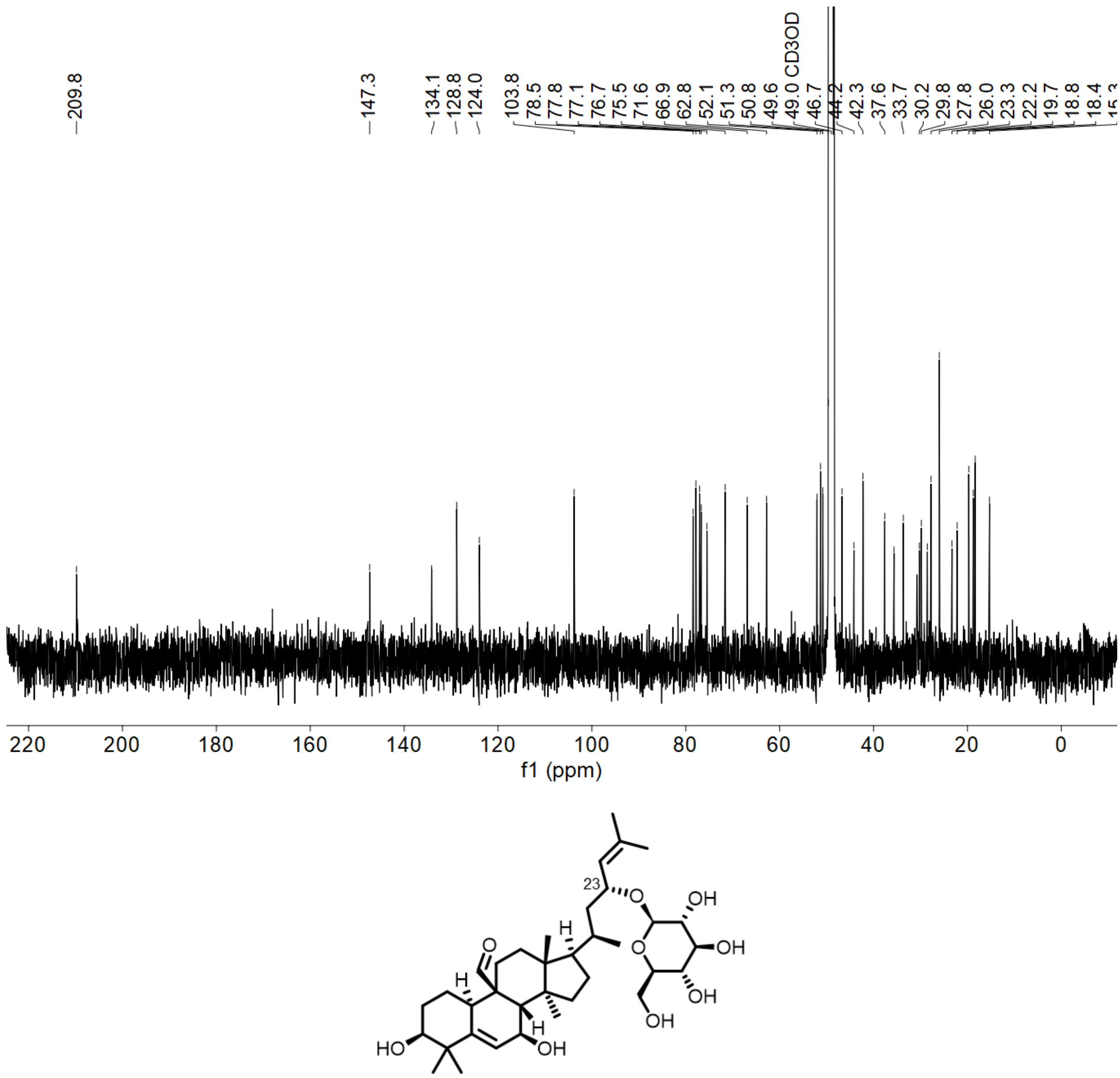


**Figure S38.** **$^{13}$C (125 MHz, methanol-*d₄*) NMR spectrum of momordicine II (35).**

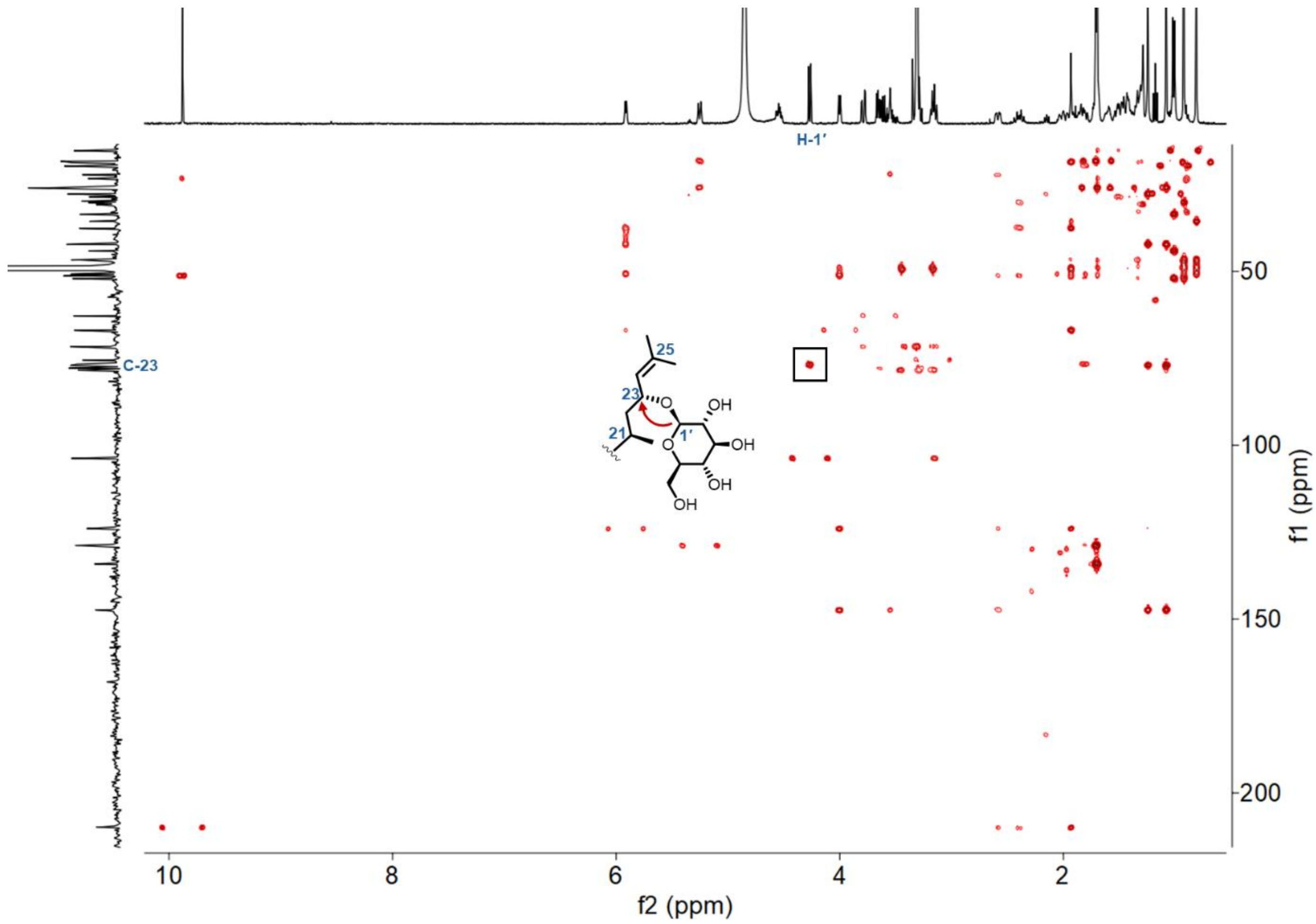


**Figure S39. HMBC spectrum of momordicine II (35).**

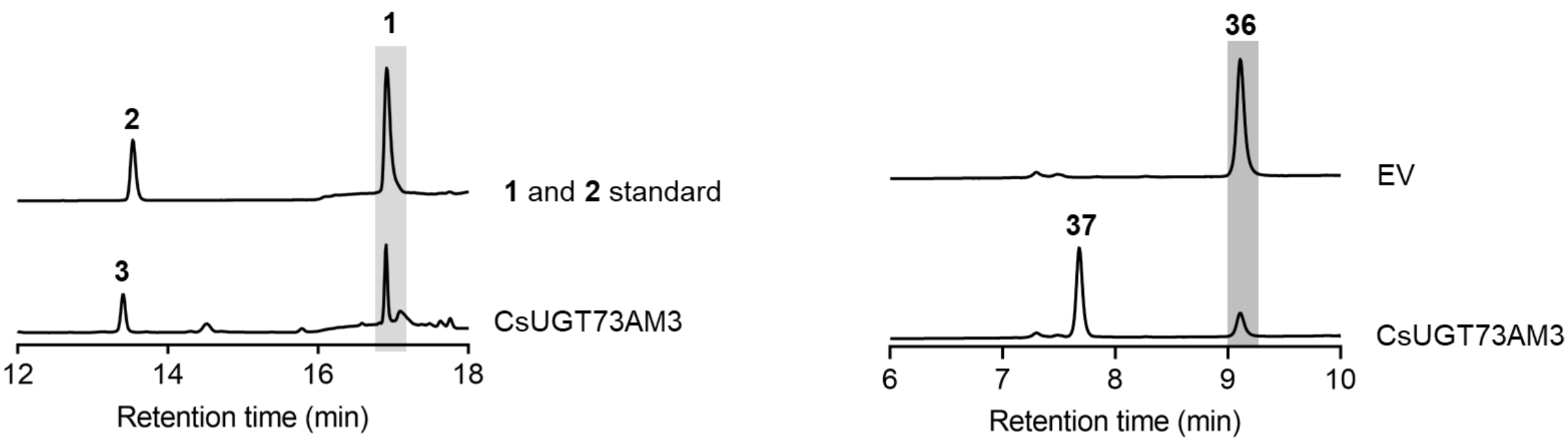


**Figure S40.** ***In vitro*** **assay of CsUGT73AM3 with mogrol (1) and cucurbitacin IIb (36).**

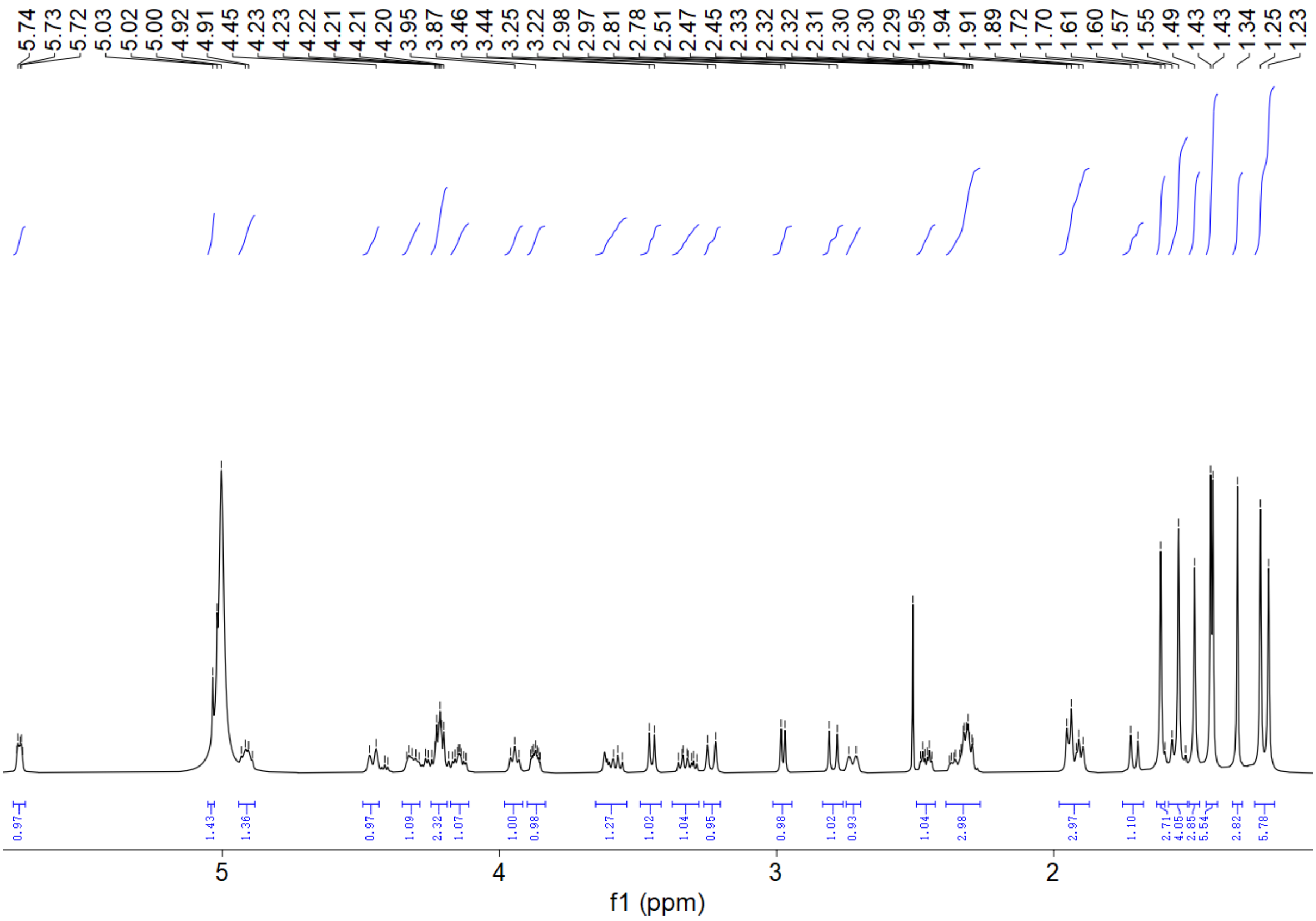

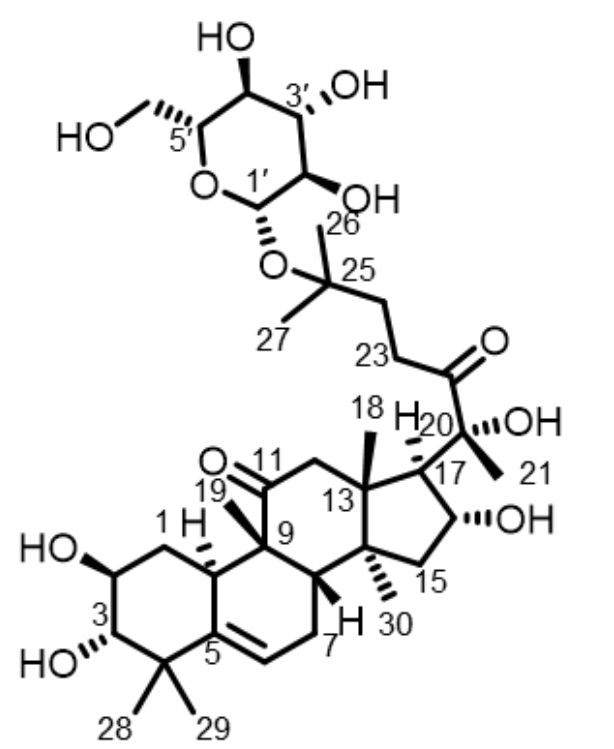

**Figure S41. $^{1}$H (400 MHz, pyridine-*d*5) NMR spectrum of cucurbitacin IIb 25-*O*-glucoside (37).**

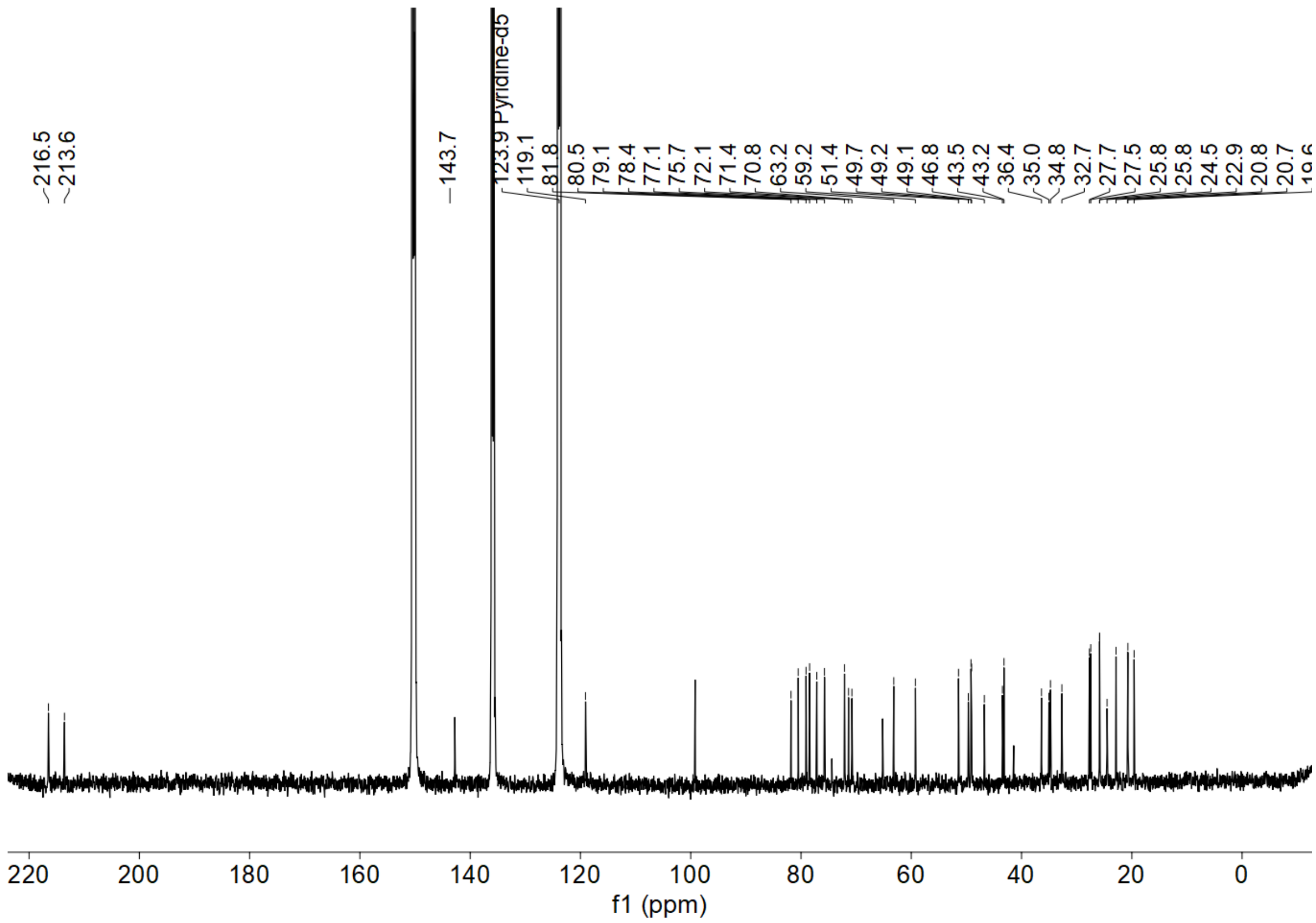

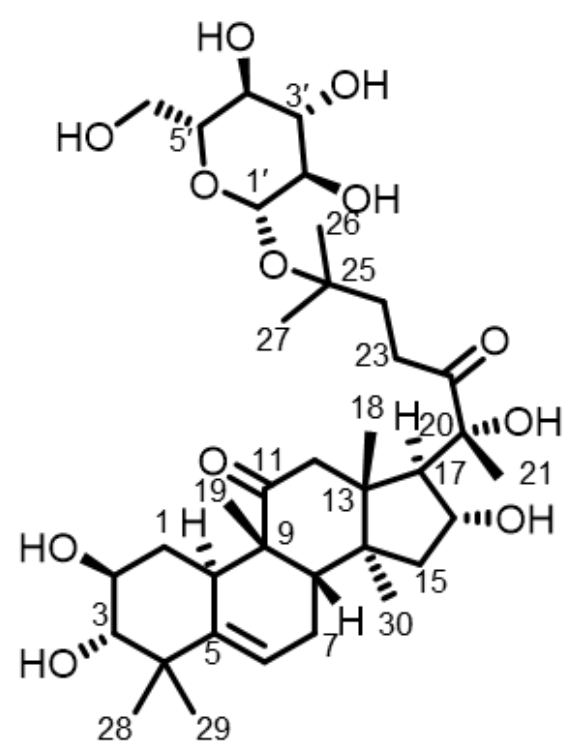

**Figure S42. $^{13}$C (125 MHz, pyridine-$d_5$) NMR spectrum of cucurbitacin IIb 25-*O*-glucoside (37).** $\delta_C$ (ppm): 35.0 (C-1); 71.4 (C-2); 81.8 (C-3); 43.5 (C-4); 143.7 (C-5); 119.1 (C-6); 22.9 (C-7); 43.2 (C-8); 49.1 (C-9); 34.8 (C-10); 213.6 (C-11); 49.2 (C-12); 49.7 (C-13); 51.4 (C-14); 46.8 (C-15); 70.8 (C-16); 59.2 (C-17); 20.7 (C-18); 20.8 (C-19); 80.5 (C-20); 24.5 (C-21); 216.5 (C-22); 32.7 (C-23); 36.4 (C-24); 77.1 (C-25); 27.7 (C-26); 27.5 (C-27); 19.6 (C-28); 25.9 (C-29); 25.8 (C-30); 99.6 (C-1′); 75.7 (C-2′); 79.1 (C-3′); 72.1 (C-4′); 78.4 (C-5′); 63.2 (C-6′).

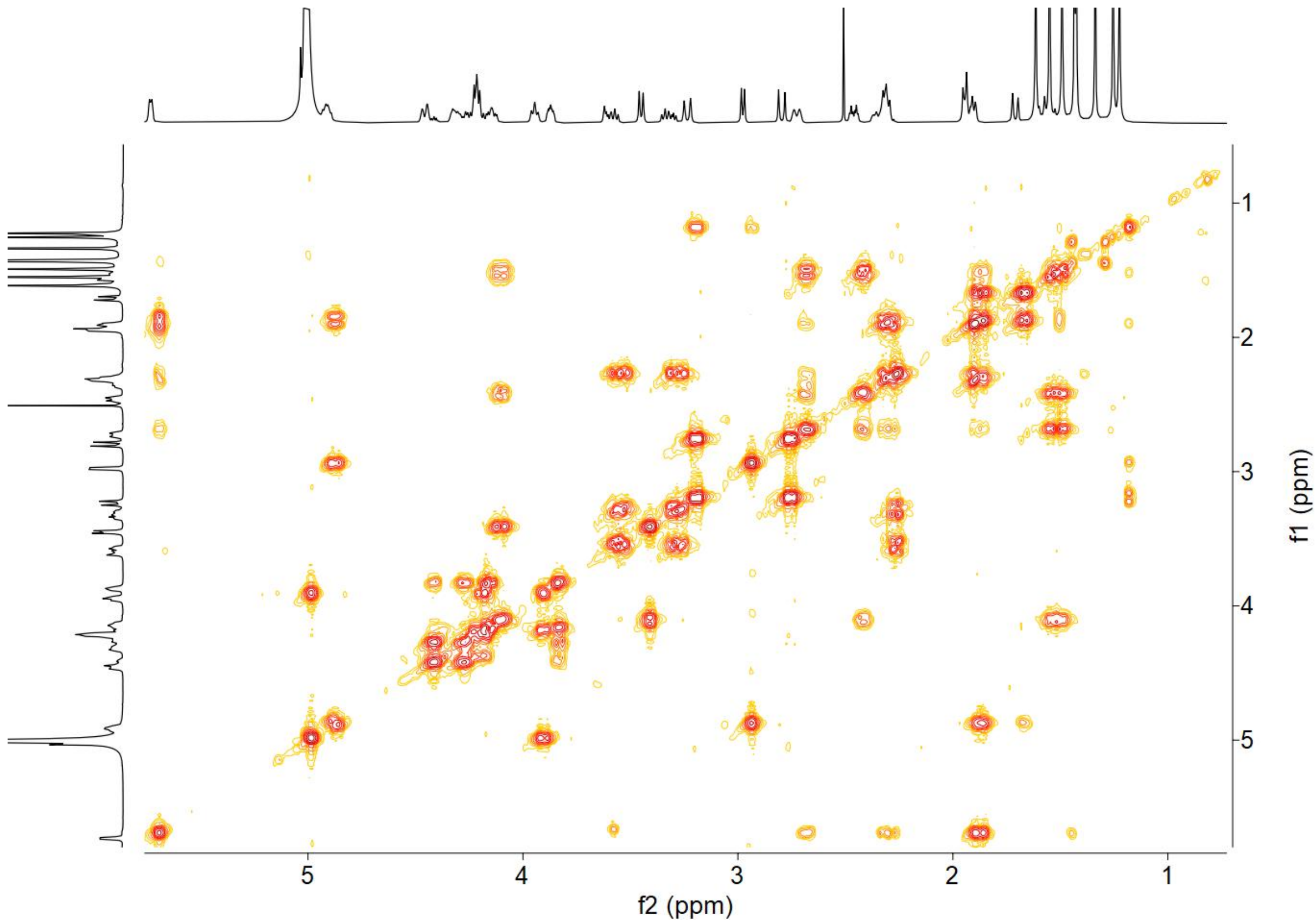


**Figure S43. H-H COSY spectrum of cucurbitacin IIb 25-*O*-glucoside (37).**

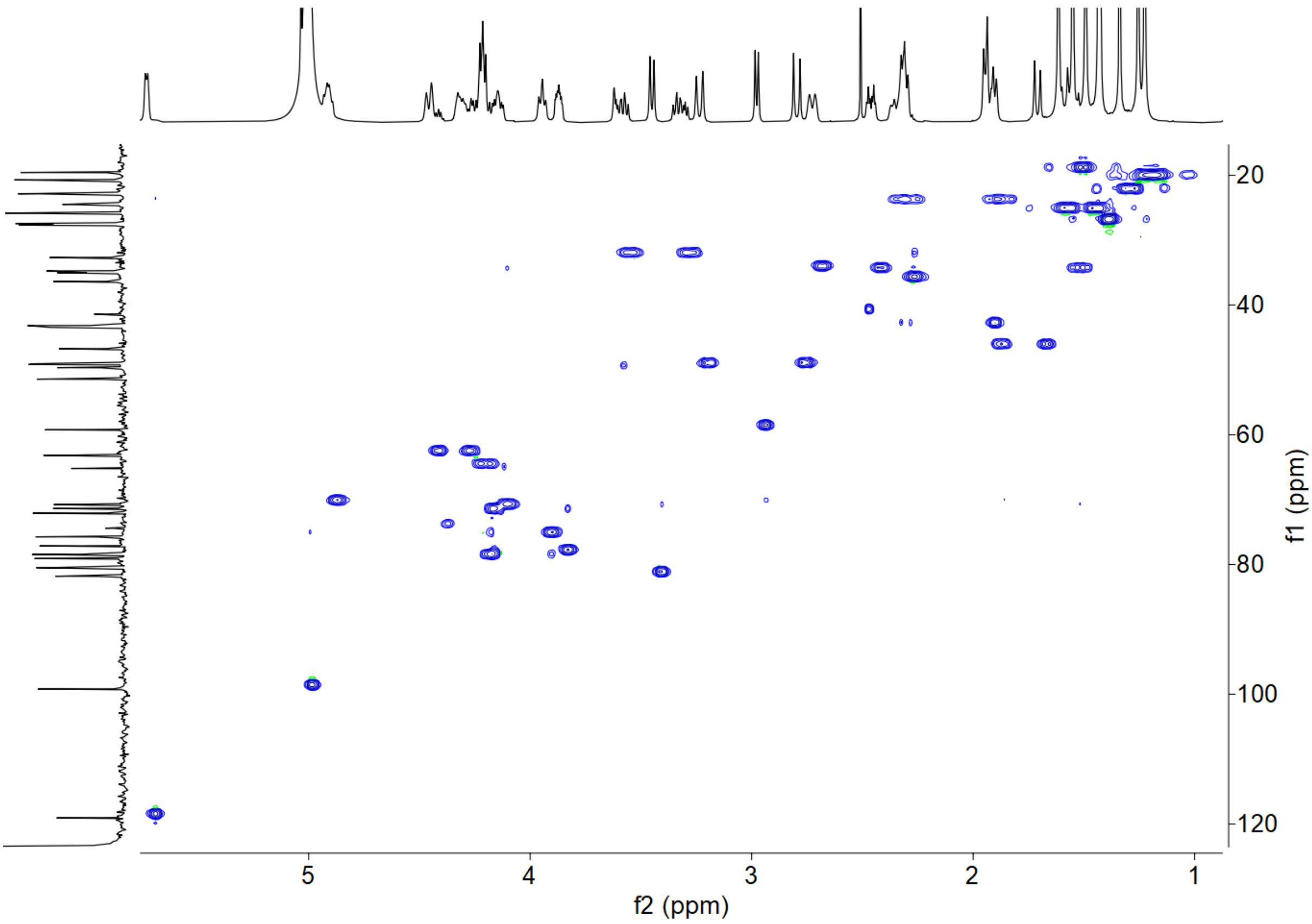


**Figure S44. HSQC spectrum of cucurbitacin IIb 25-*O*-glucoside (37).**

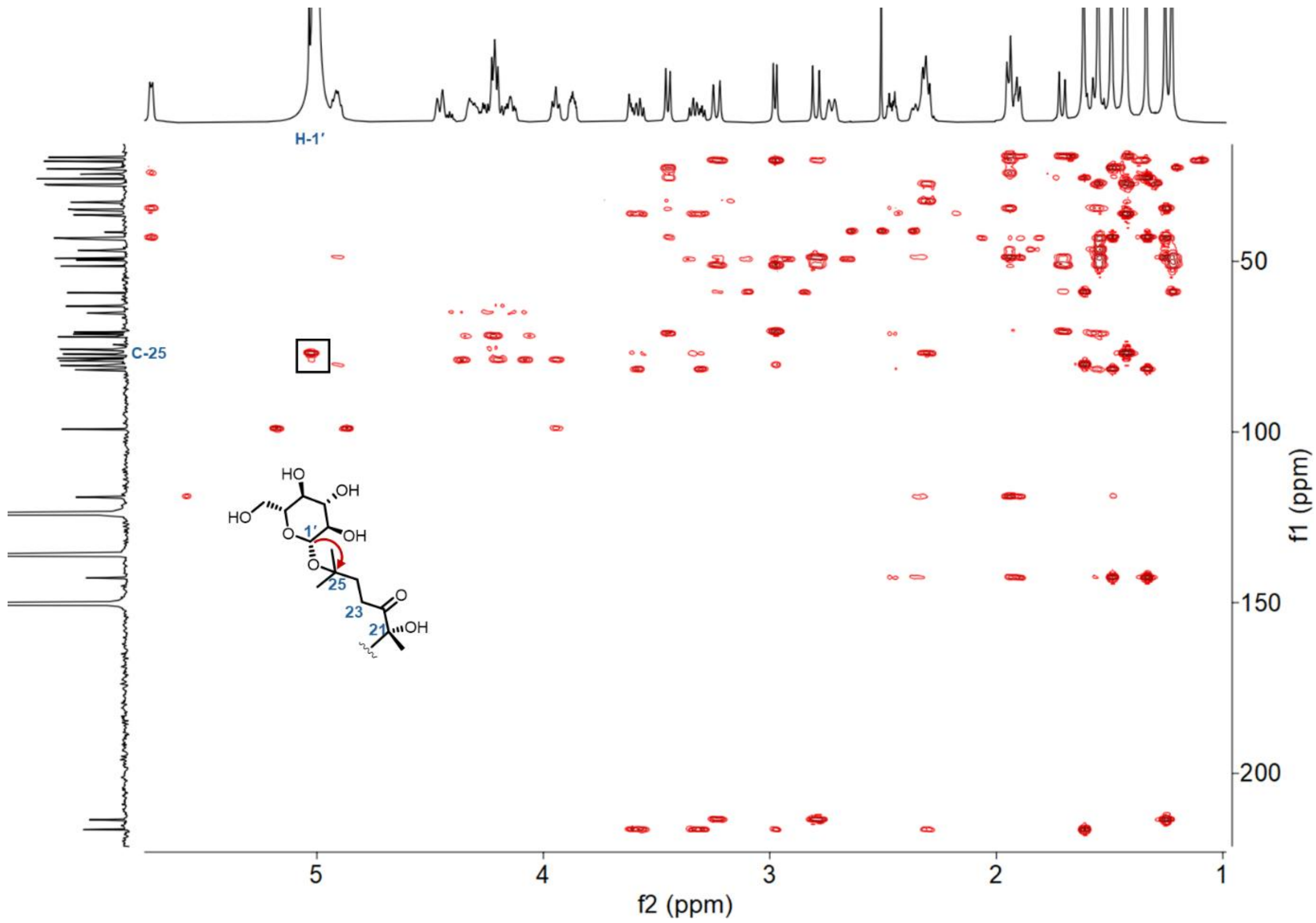


**Figure S45. HMBC spectrum of cucurbitacin IIb 25-*O*-glucoside (37).**

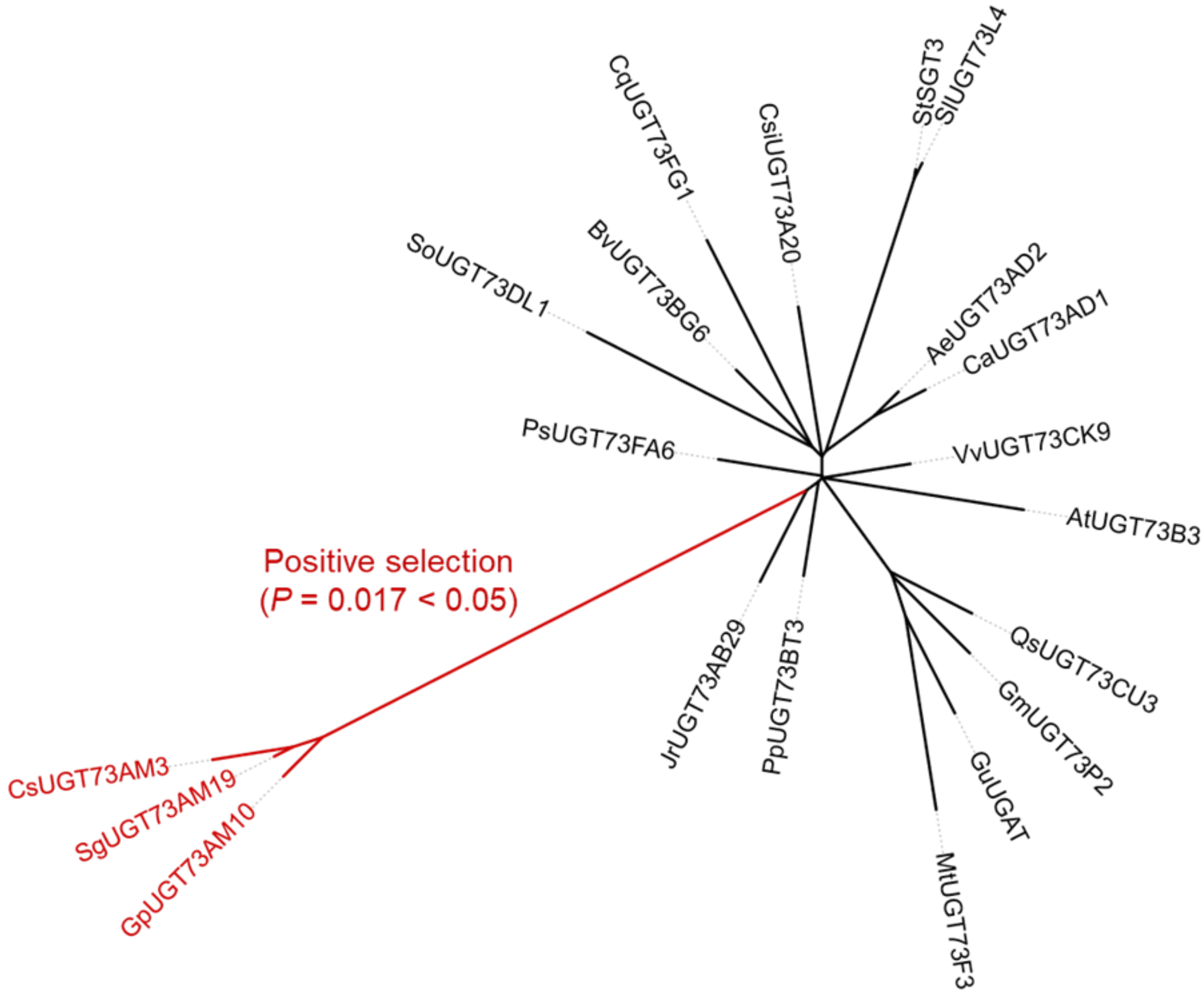


**Figure S46. Maximum likelihood tree of functional characterized UGT73.** Positive selection of Cucurbitaceae UGT73AMs were detected (p = 0.017). Leaf labels correspond to the following NCBI accession numbers: StSGT3 (ABB84472), SlUGT73L4 (ADQ37966), CsiUGT73A20 (ALO19886), GmUGT73P2 (BAI99584), GuUGAT (ANJ03631), MtUGT73F3 (ACT34898), AtUGT73B3 (AAM47999), CqUGT73FG1 (XM_021858666.1), SoUGT73DL1 (OR426405), CaUGT73AD1 (ALD84259), AeUGT73AD2 (UMX47351) and QsUGT73CU3 (WEU75099). The other UGT73s amino acid sequences are listed in Table S10.

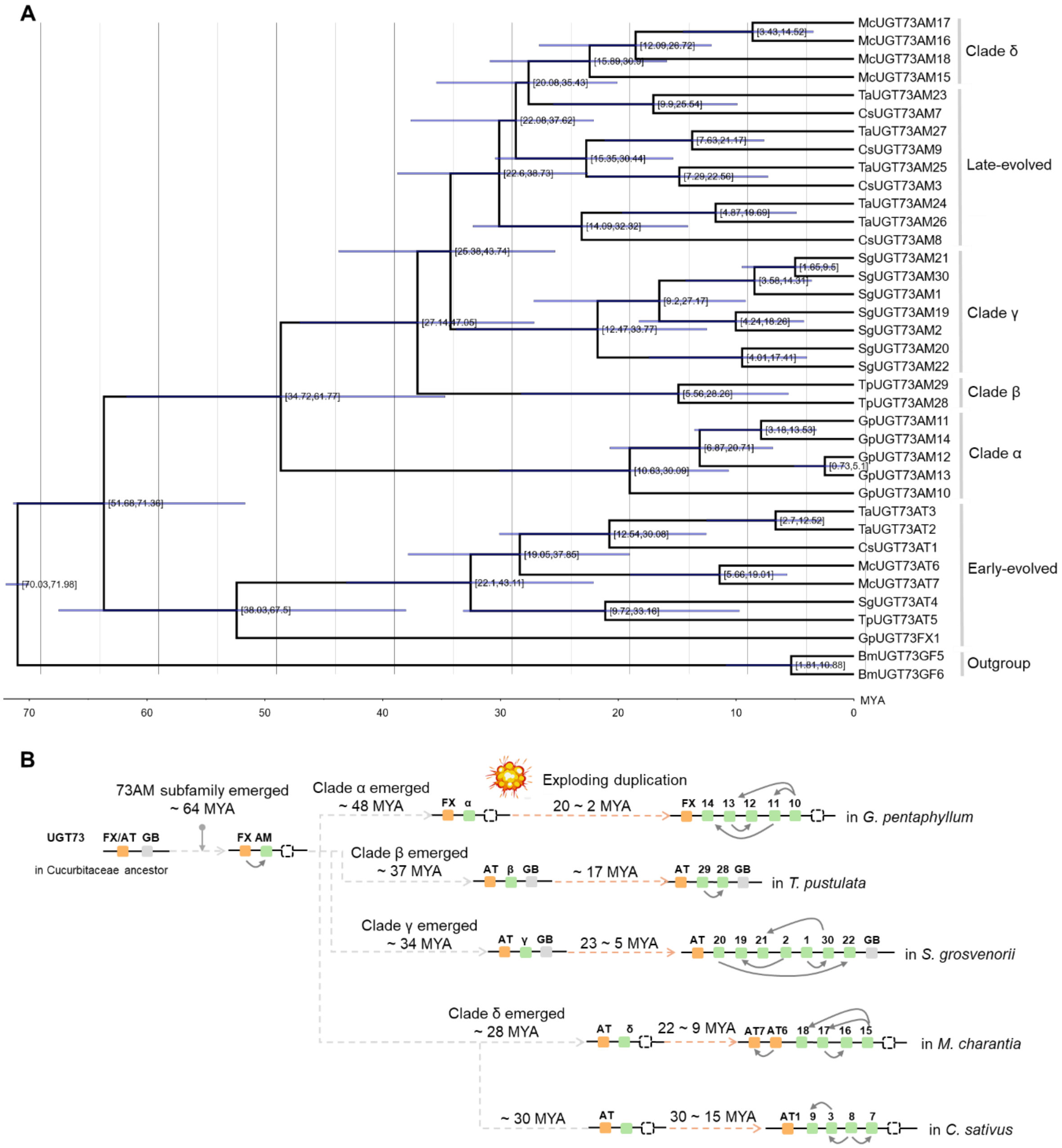


**Figure S47. Origin and evolution of UGT73AMs in Cucurbitaceae.** (**A**) Bayesian phylogeny of Cucurbitaceae UGT73AMs. The divergence time between BmUGT73GF and Cucurbitaceae UGT73AMs was calibrated to 70.5 million years ago (MYA), based on the Begoniaceae-Cucurbitaceae divergence time obtained from TimeTree (https://timetree.org). (**B**) Proposed evolutionary pathway of UGT73AMs in Cucurbitaceae plants.

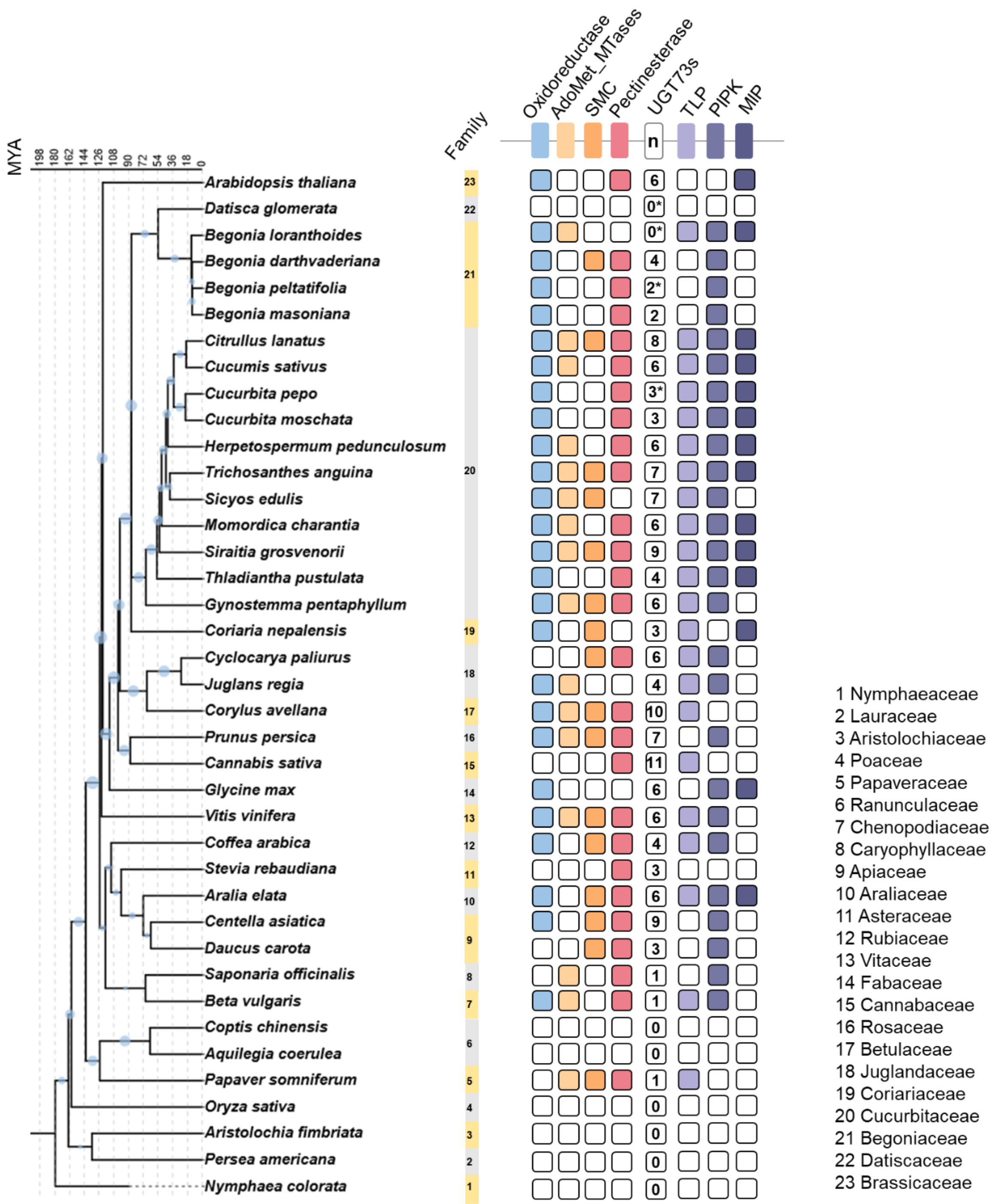


**Figure S48. Distribution of 'UGT73 tandem array' across angiosperms species.** The asterisk (*) indicated that the number of tandem duplicates of UGT73 is uncertain due to gaps in the genomic region or low quality of genome assemblies. Blue circles at nodes indicate bootstrap support values, with circle size proportional to the value ranging from 13 to 98. SMC, chromosome segregation protein; TLP, thaumatin-like proteins; PIPK, phosphatidylinositol phosphate kinase; MIP, major intrinsic protein.

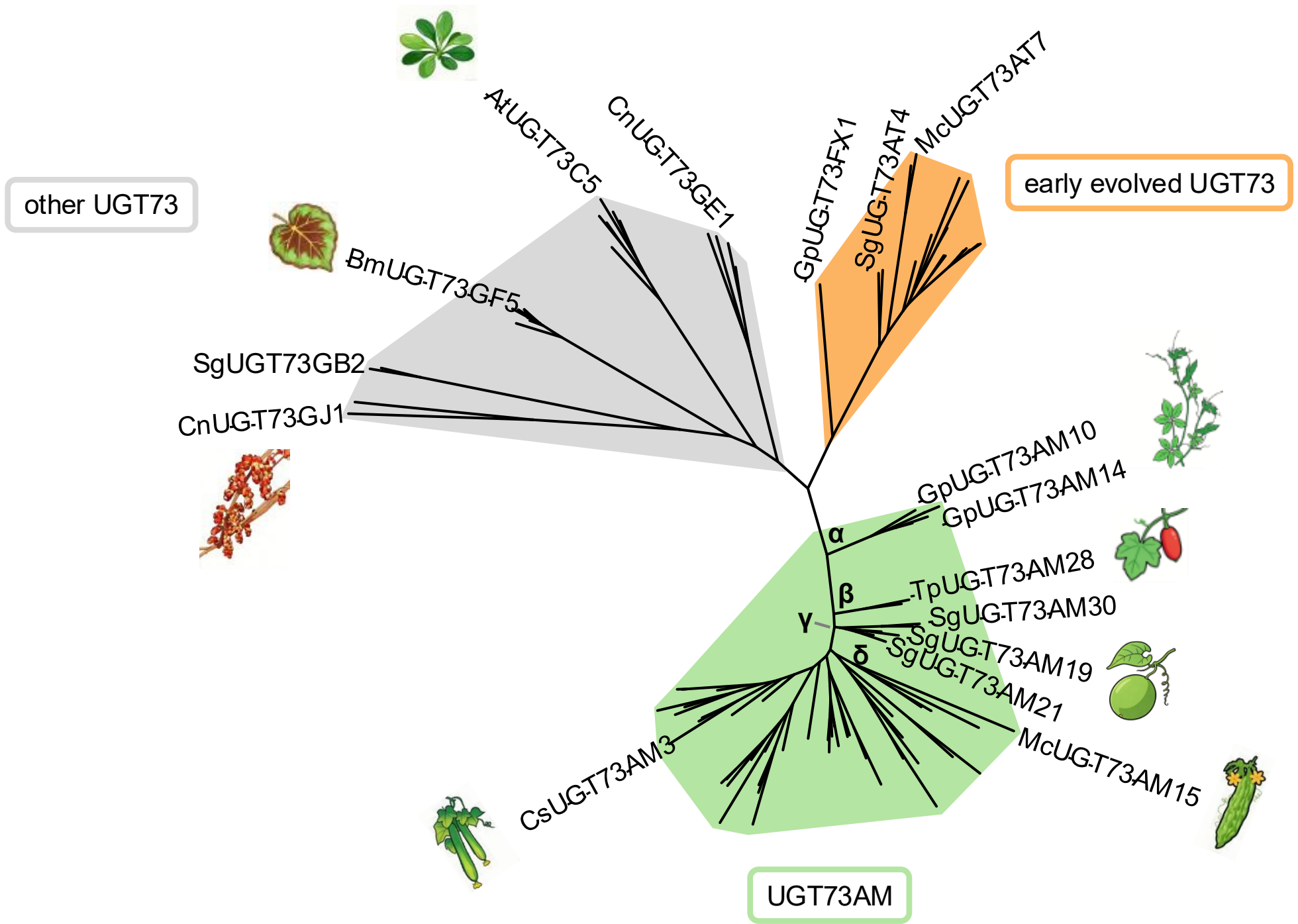


| | Cucurbitales | | | Brassicales |
|---|---|---|---|---|
| | Begoniaceae | Coriariaceae | Cucurbitaceae | Brassicaceae |
| UGT73AM | × | × | ○ | × |
| UGT73AT | × | × | ○ | × |
| UGT73GB | × | × | ○ | × |
| UGT73FX | × | × | ○ | × |
| UGT73GE | × | ○ | × | × |
| UGT73GF | ○ | × | × | × |
| UGT73GJ | × | ○ | × | × |
| UGT73C | × | × | × | ○ |

**Figure S49. Phylogeny of UGT73s in Cucurbitales (AtUGT73Cs were used as outgroup).** Generally, the UGT73 subfamilies listed here were found specific to each family.

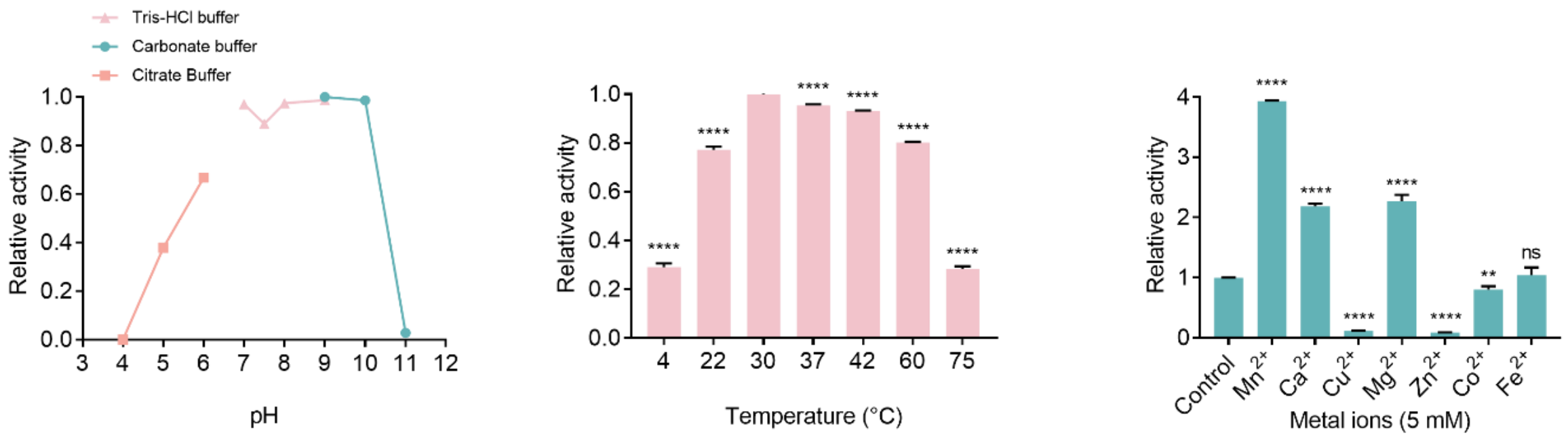


**Figure S50. Optimization of SgUGT73AM30 activity.** (**A**) The optimal pH for enzymatic reaction was 8~10. pH 8 was used in subsequent experiments. Tris-HCl buffer, 20 mM Tris base; Carbonate buffer, 50 mM $Na_2HPO_4$-$NaH_2PO_4$; Citrate buffer, 50 mM Citric Acid- Sodium Citrate. (**B**) The optimal temperature was determined as 30°C. (**C**) Influence of different divalent metal ions on the reactions. Copper and zinc ions significantly inhibited enzymatic activity; on the contrary, manganese, calcium and magnesium ions improved the activity. UDPG and mogrol (**1**) were used as the sugar donor and acceptor, respectively. Data are presented as the mean ± SD of three independent biological replicates (*n*=3). Statistical significance versus the control group (relative activity defined as 1.0) was determined by unpaired two-tailed Student's t-test (**, $P < 0.01$, ****, $P < 0.0001$; ns, not significant difference).

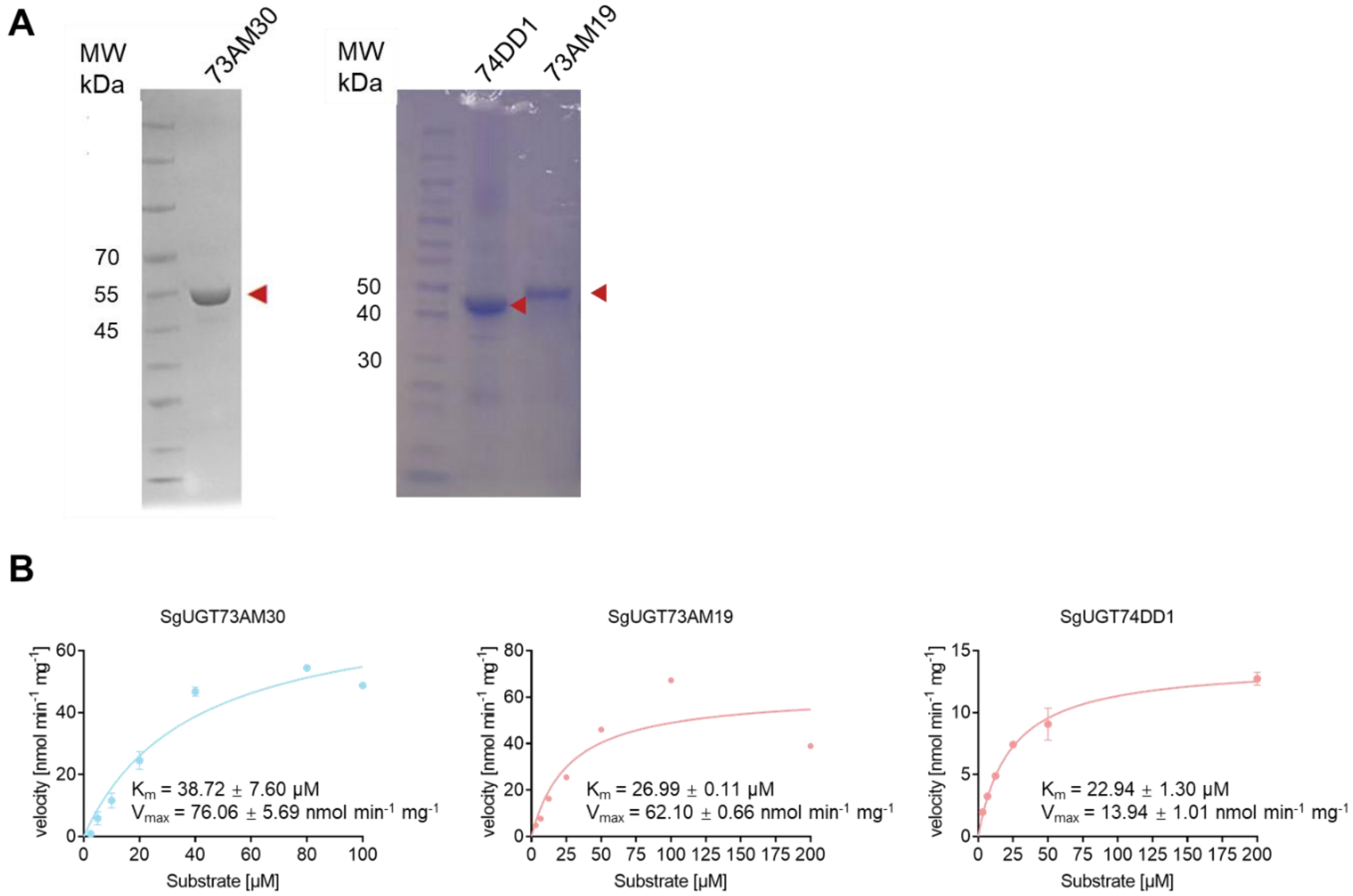


**Figure S51. Enzyme expression and kinetics of SgUGT73AM30, SgUGT73AM19 and SgUGT74DD1.** (**A**) SDS-PAGE analysis of the $His_6$-tagged proteins purified by nickel magnetic beads. **(B)** Determination of kinetic parameters for SgUGT73AM30, SgUGT73AM19 and SgUGT74DD1. The kinetic curves (initial velocity vs. substrate concentration) were fitted to the Michaelis-Menten equation. The derived parameters (Km and Vmax) are presented as mean ±SD from three independent experiments.

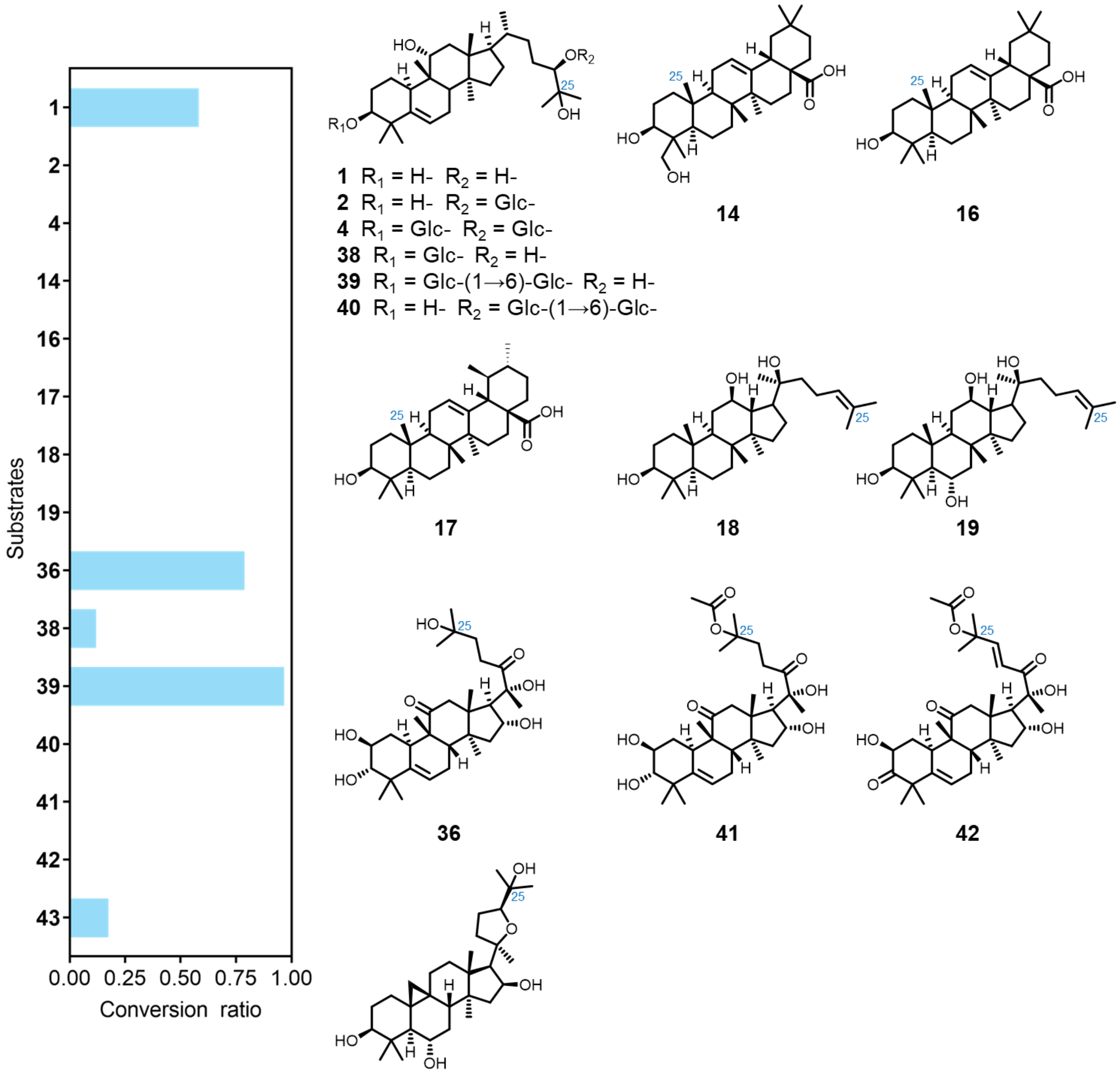


**Figure S52. Screening the substrates of SgUGT73AM30.** The substrate library consists of 15 compounds including mogrol (**1**), mogroside IA1 (**2**), mogroside IIE (**4**), hederagenin (**14**), oleanolic acid (**16**), ursolic acid (**17**), (20*S*)-protopanaxadiol (**18**), (20*S*)-protopanaxatriol (**19**), cucurbitacin IIb (**36**), mogroside IE1 (**38**), mogroside IIA2 (**39**), mogroside IIA1 (**40**), cucurbitacin IIa (**41**), cucurbitacin B (**42**), cycloastragenol (**43**).

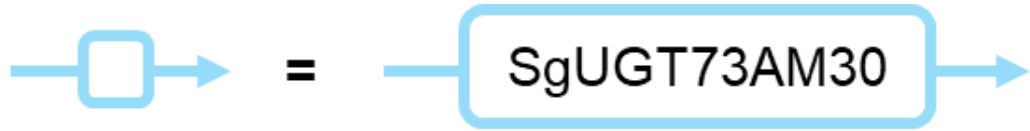


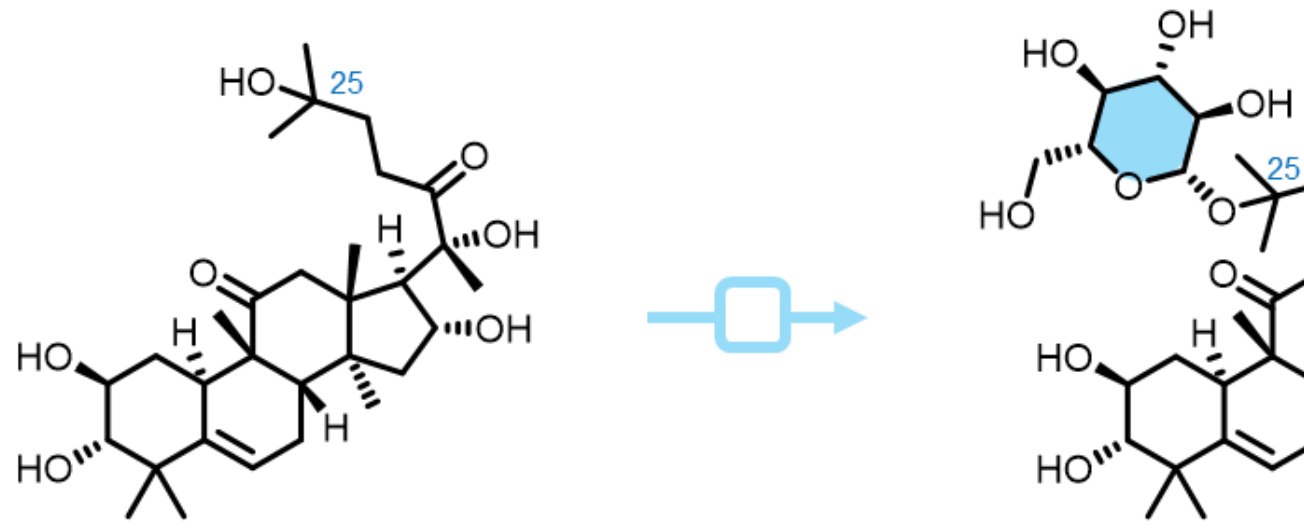

36 37

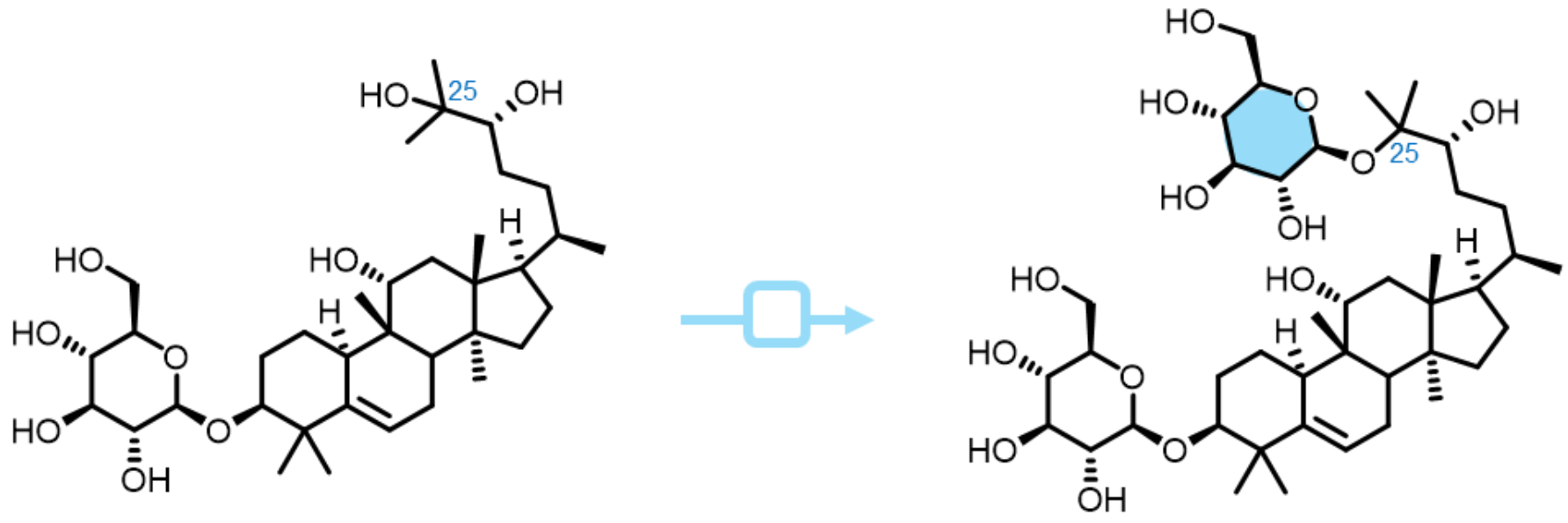

38 44*

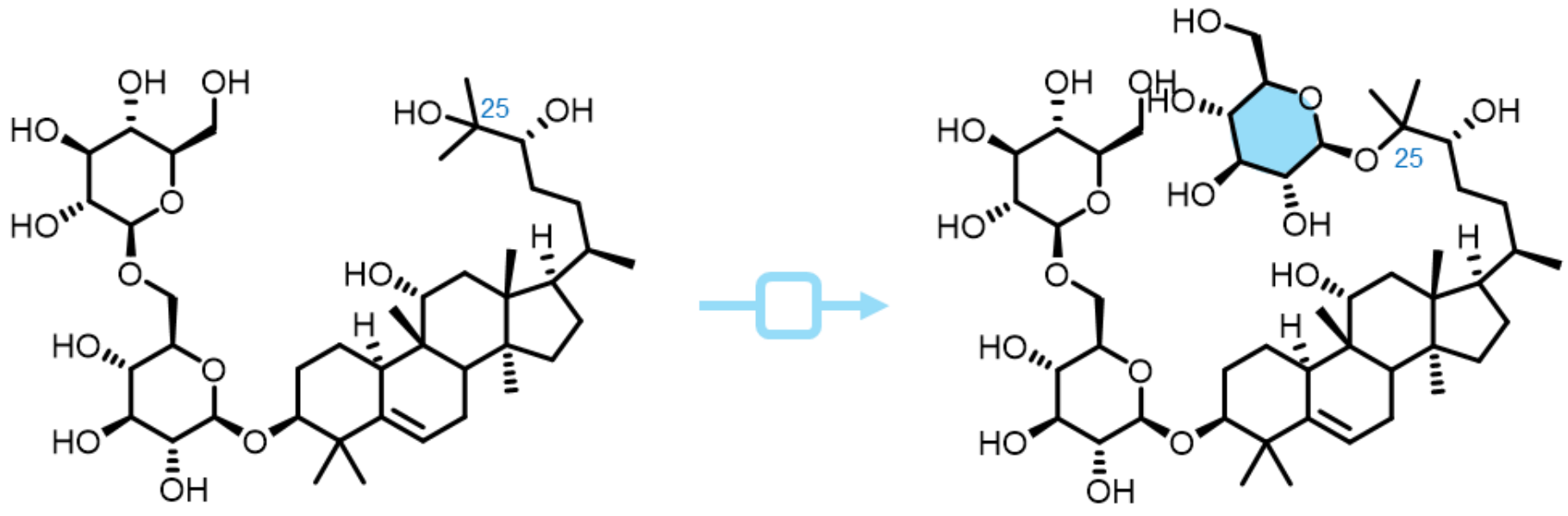

39 45

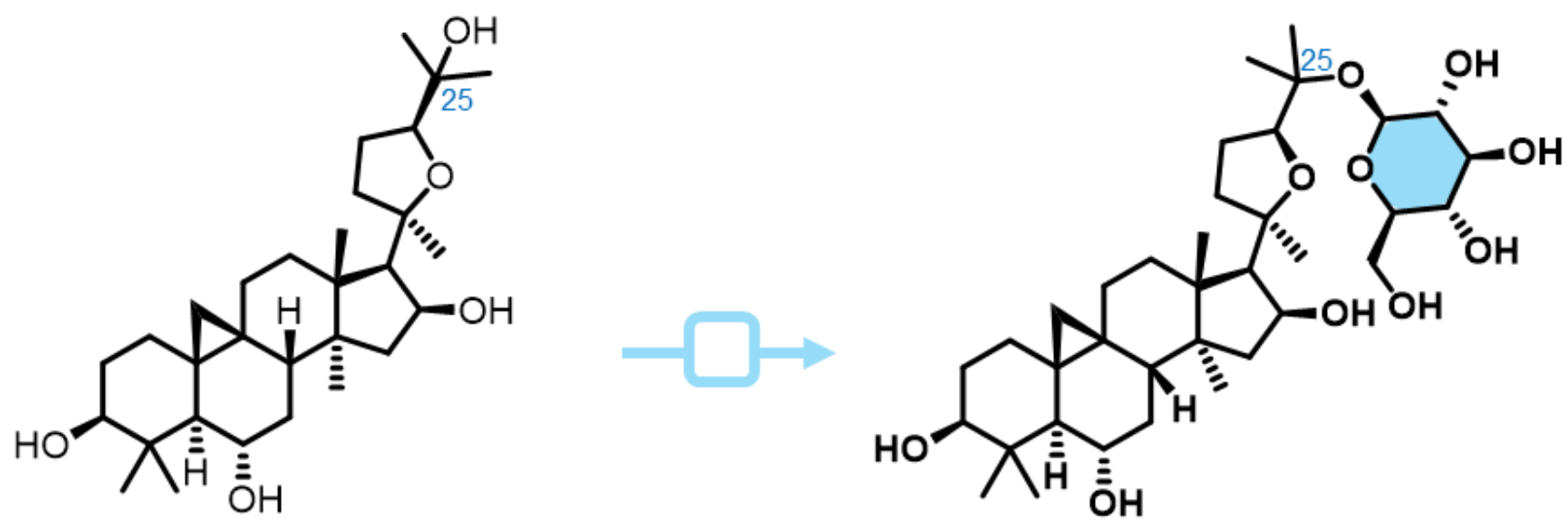

43 46

**Figure S53. Characterized glycosylation activity of SgUGT73AM30.** An asterisk denotes that NMR data are not available for this product.

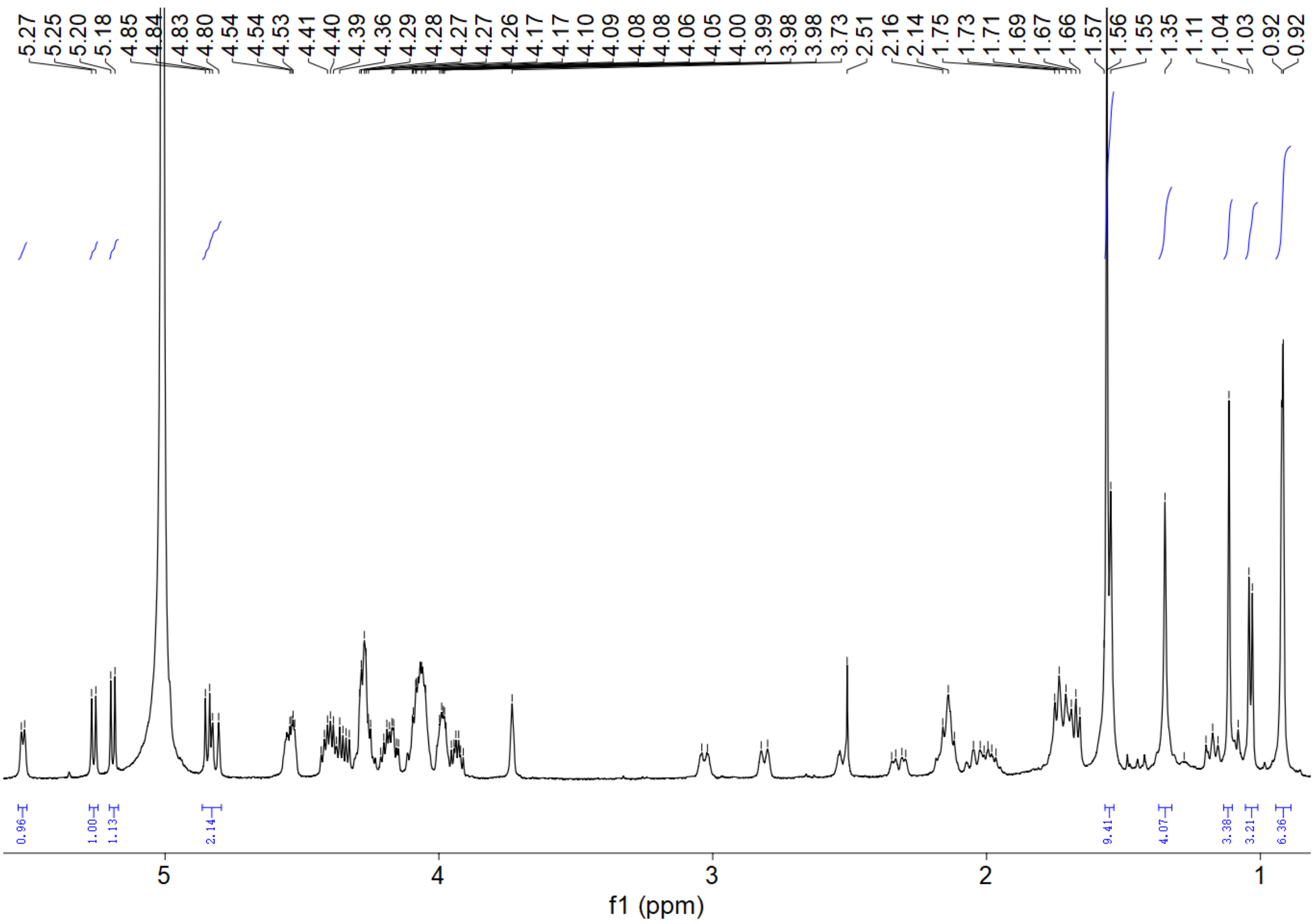

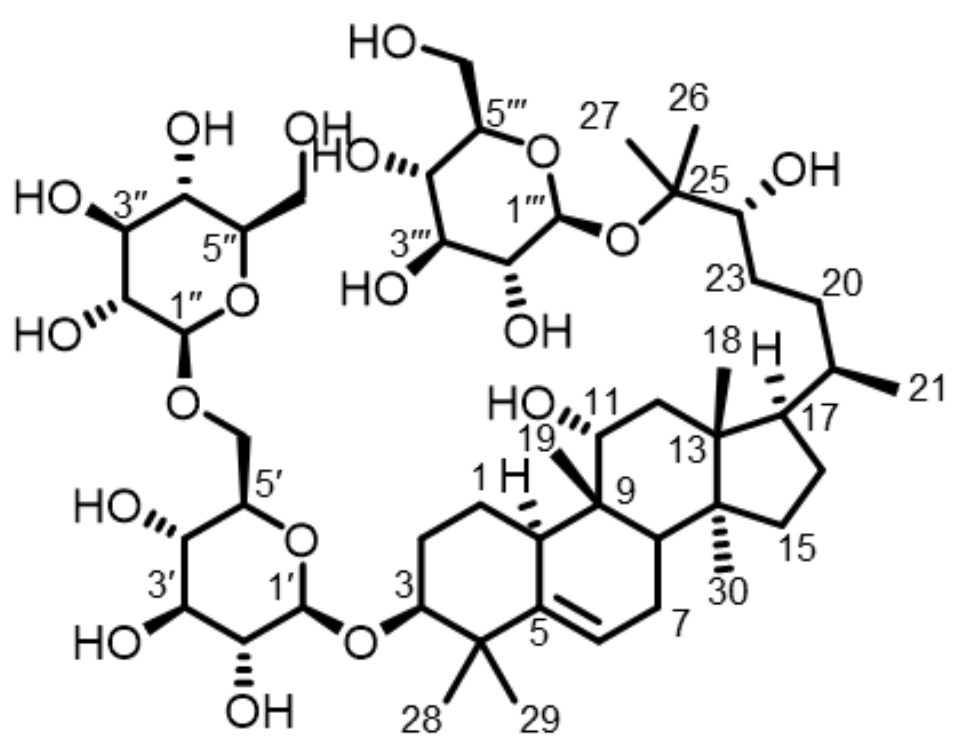

**Figure S54. $^{1}$H (400 MHz, pyridine-$d_5$) NMR spectrum of mogroside IIA2 25-*O*-glucoside (45).**

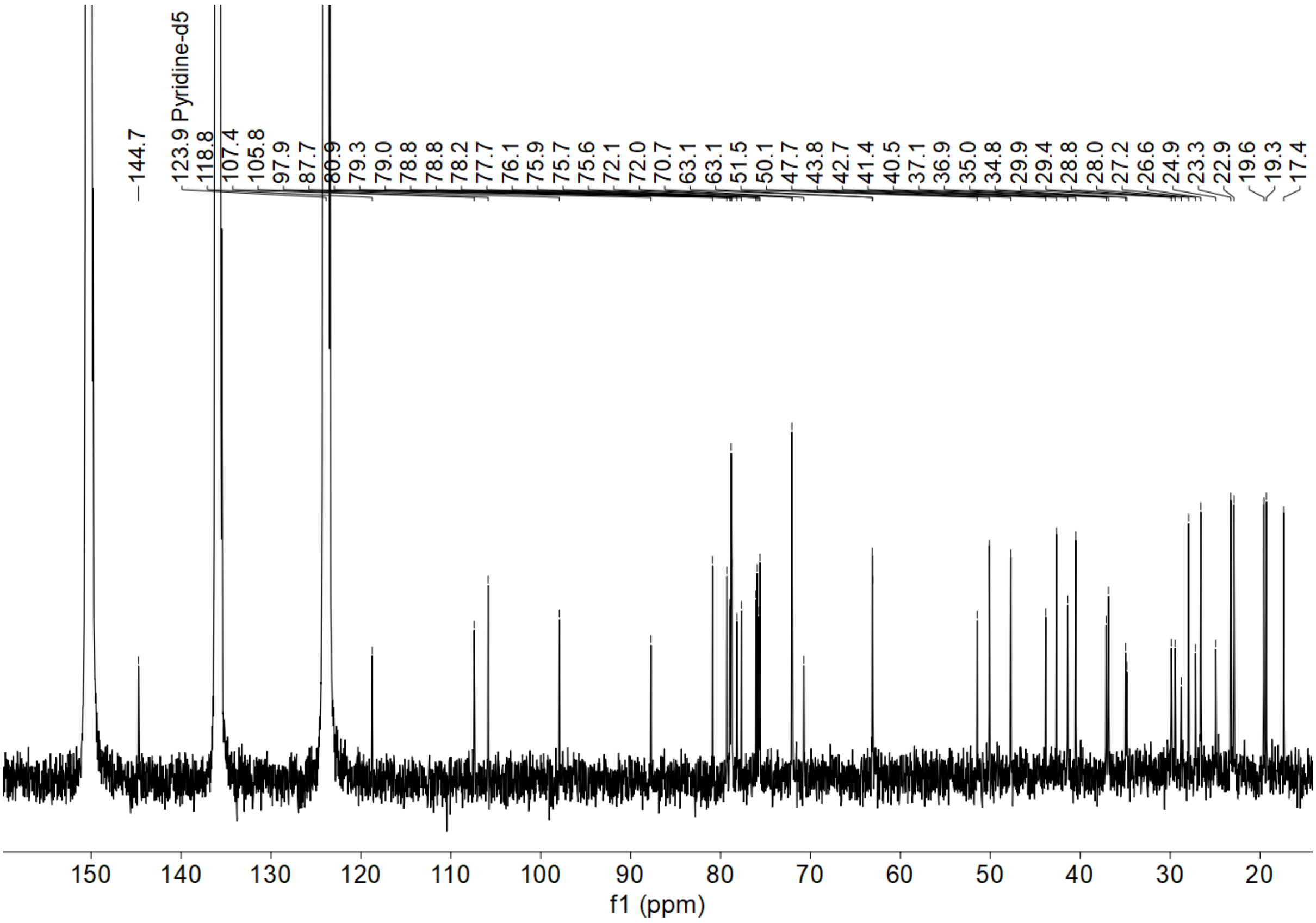

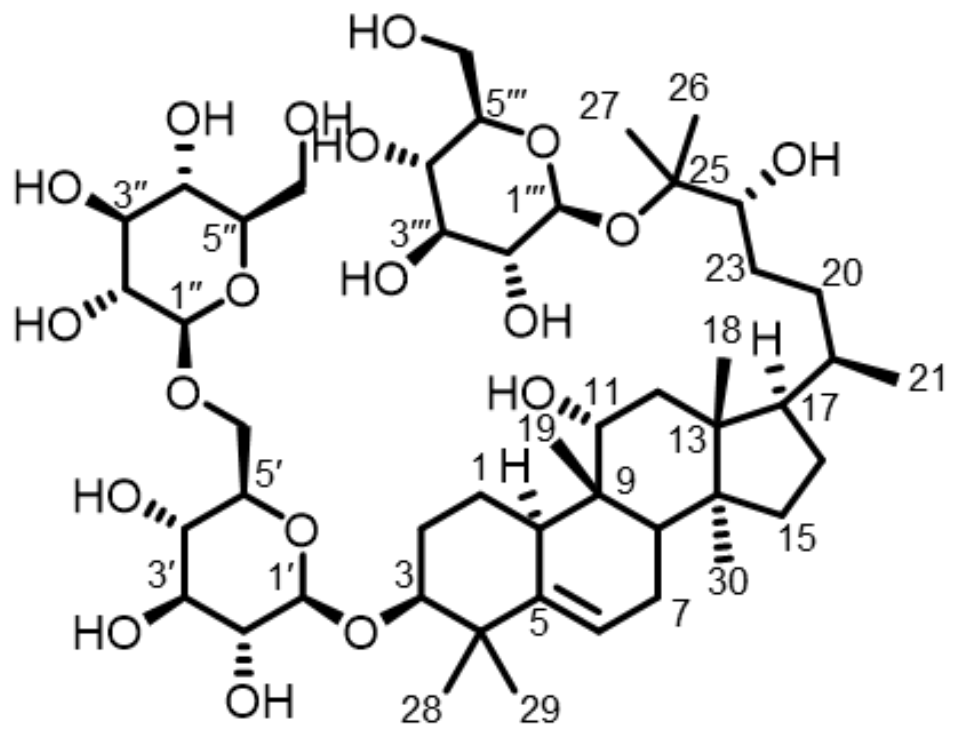

**Figure S55. $^{13}$C (125 MHz, pyridine-$d_5$) NMR spectrum of mogroside IIA2 25-*O*-glucoside (45).** $\delta_C$ (ppm): 27.2 (C-1); 29.9 (C-2); 87.7 (C-3); 42.7 (C-4); 144.7 (C-5); 118.8 (C-6); 24.9 (C-7); 43.8 (C-8); 40.5 (C-9); 37.1 (C-10); 77.7 (C-11); 41.4 (C-12); 47.7 (C-13); 50.1 (C-14); 35.0 (C-15); 28.8 (C-16); 51.5 (C-17); 17.4 (C-18); 26.6 (C-19); 36.9 (C-20); 19.3 (C-21); 34.8 (C-22); 29.4 (C-23); 75.7 (C-24); 80.9 (C-25); 22.9 (C-26); 23.3 (C-27); 28.0 (C-28); 26.6 (C-29); 19.6 (C-30); 107.4(C-1′); 75.9 (C-2′); 78.8 (C-3′); 72.0 (C-4′); 78.2 (C-5′); 70.7 (C-6′); 105.8 (C-1″); 75.6 (C-2″); 78.8 (C-3″); 72.1 (C-4″); 78.8 (C-5″); 63.1 (C-6″); 97.9 (C-1‴); 76.1 (C-2‴);79.0 (C-3‴); 72.1 (C-4‴); 79.3 (C-5‴); 63.1 (C-6‴).

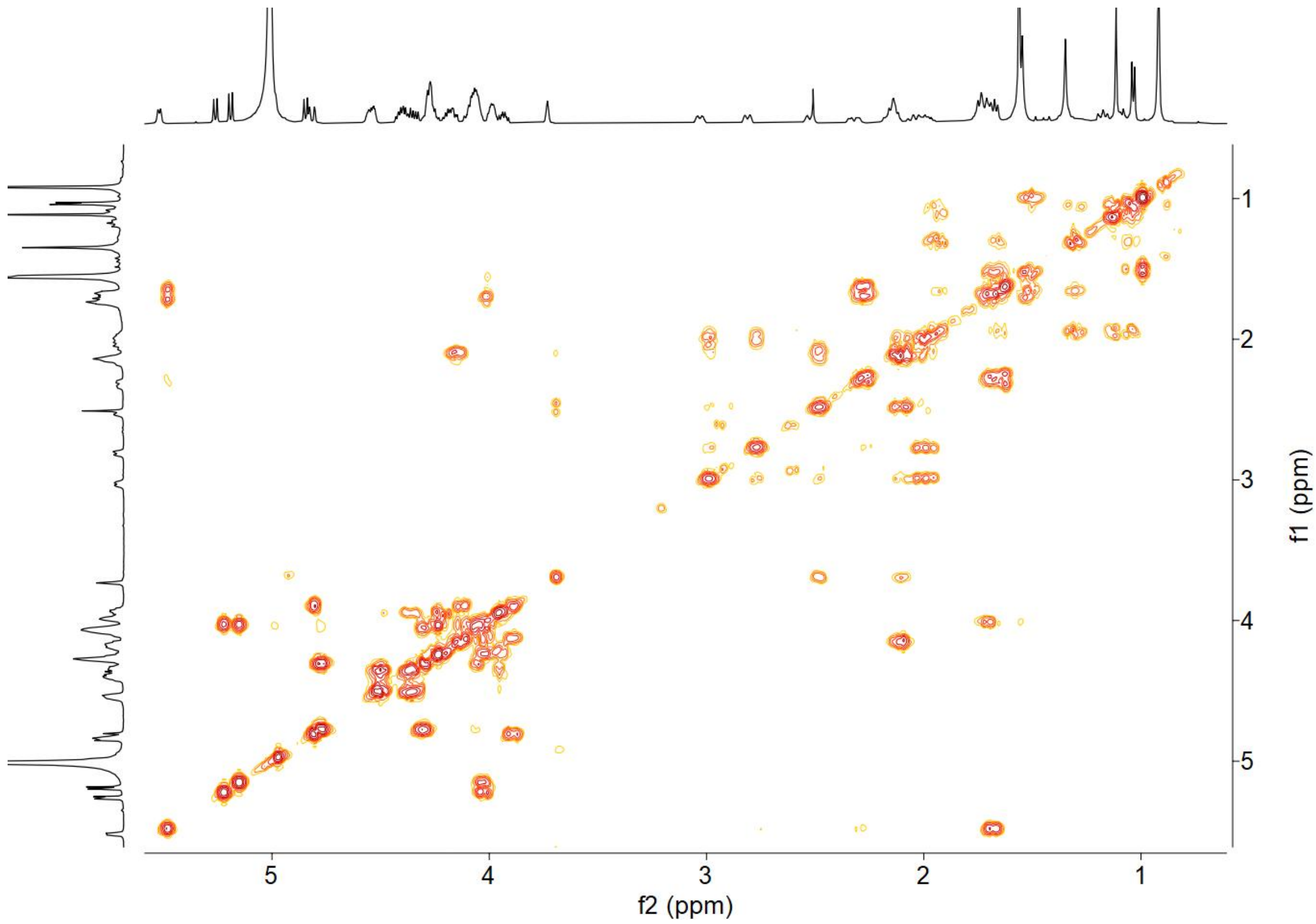


**Figure S56. H-H COSY spectrum of mogroside IIA2 25-*O*-glucoside (45).**

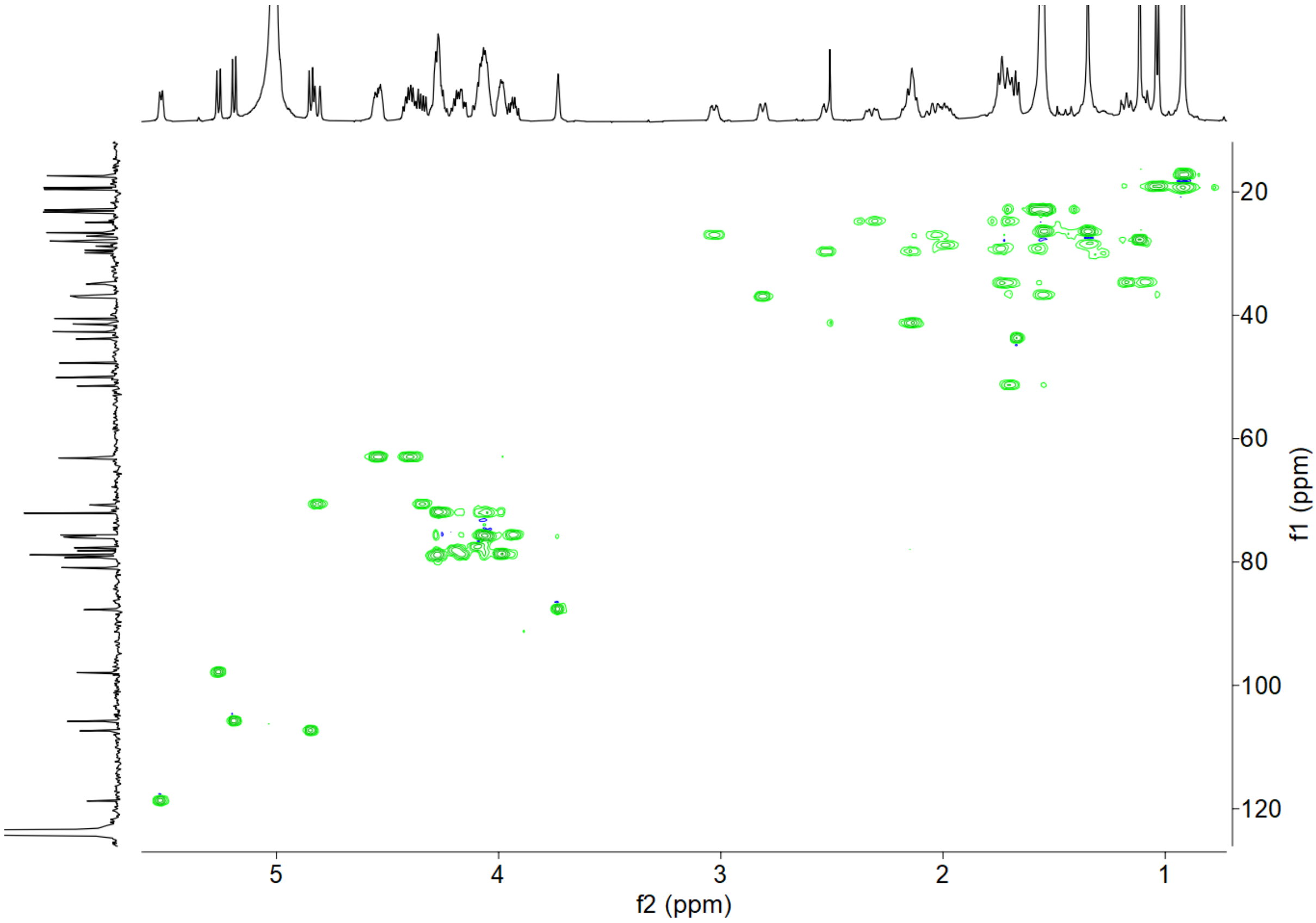


**Figure S57. HSQC spectrum of mogroside IIA2 25-*O*-glucoside (45).**

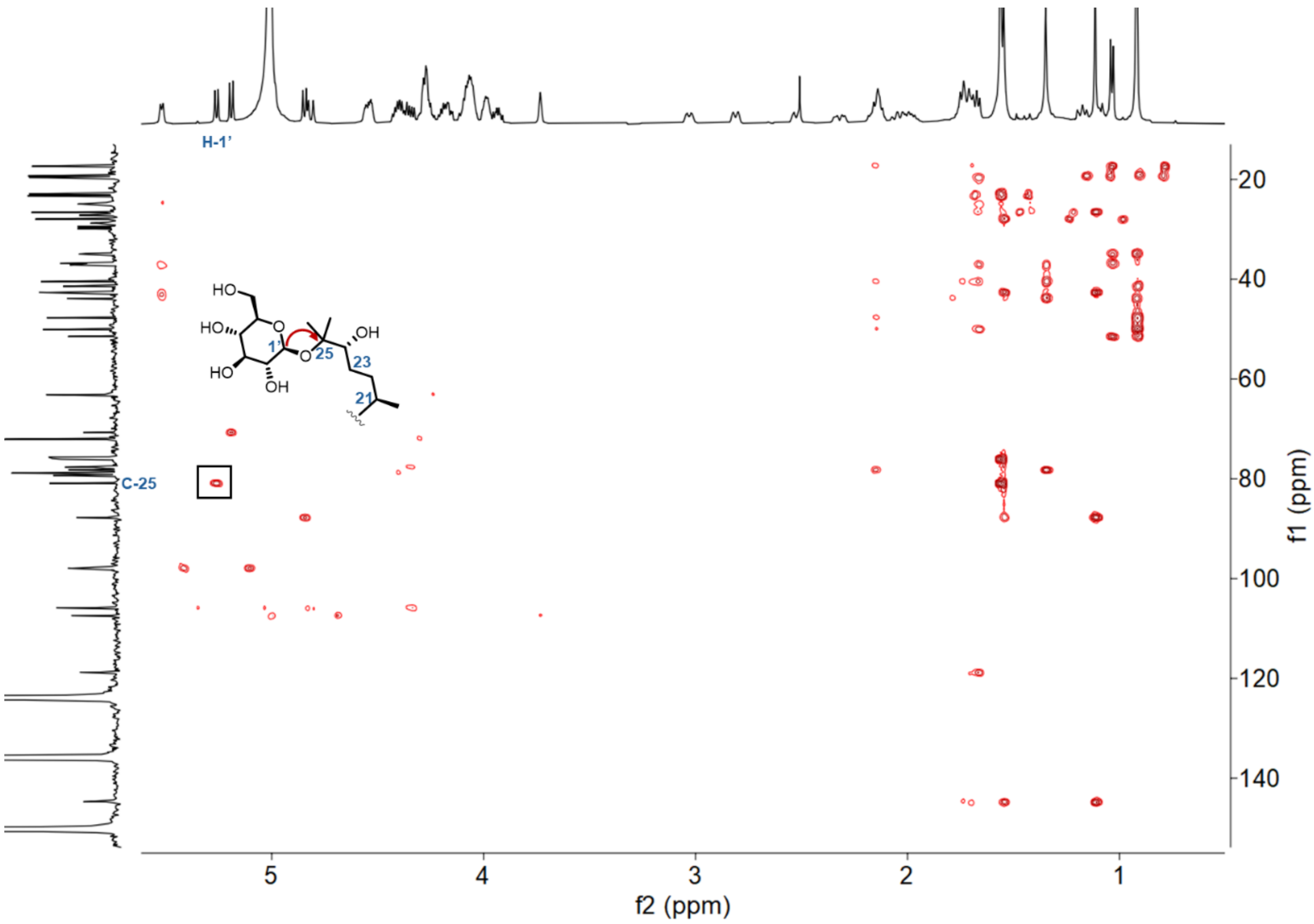


**Figure S58. HMBC spectrum of mogroside IIA2 25-*O*-glucoside (45).**

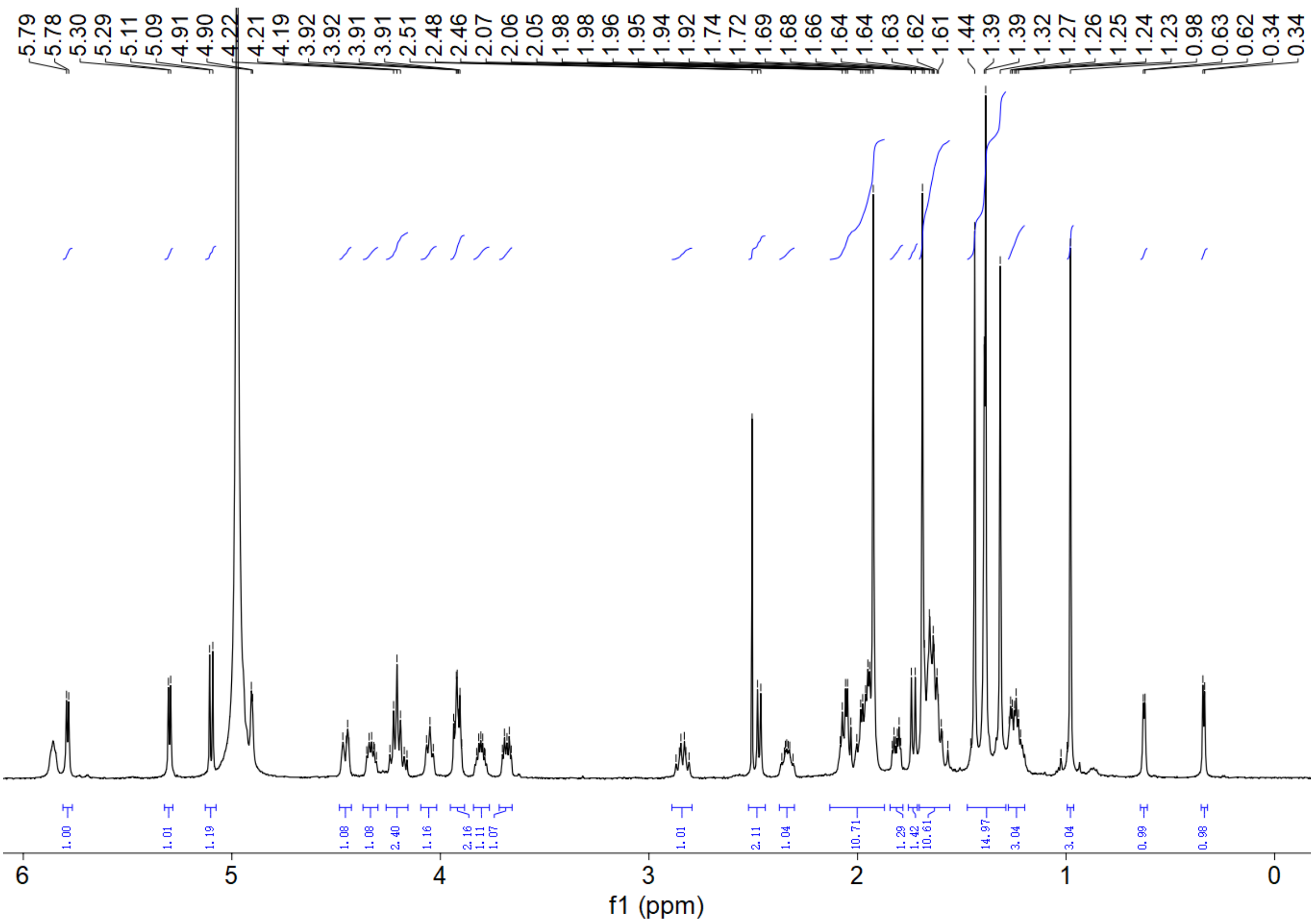

**Figure S59. $^{1}$H (400 MHz, pyridine-*d*5) NMR spectrum of cycloastragenol 25-*O*-glucoside (46).**

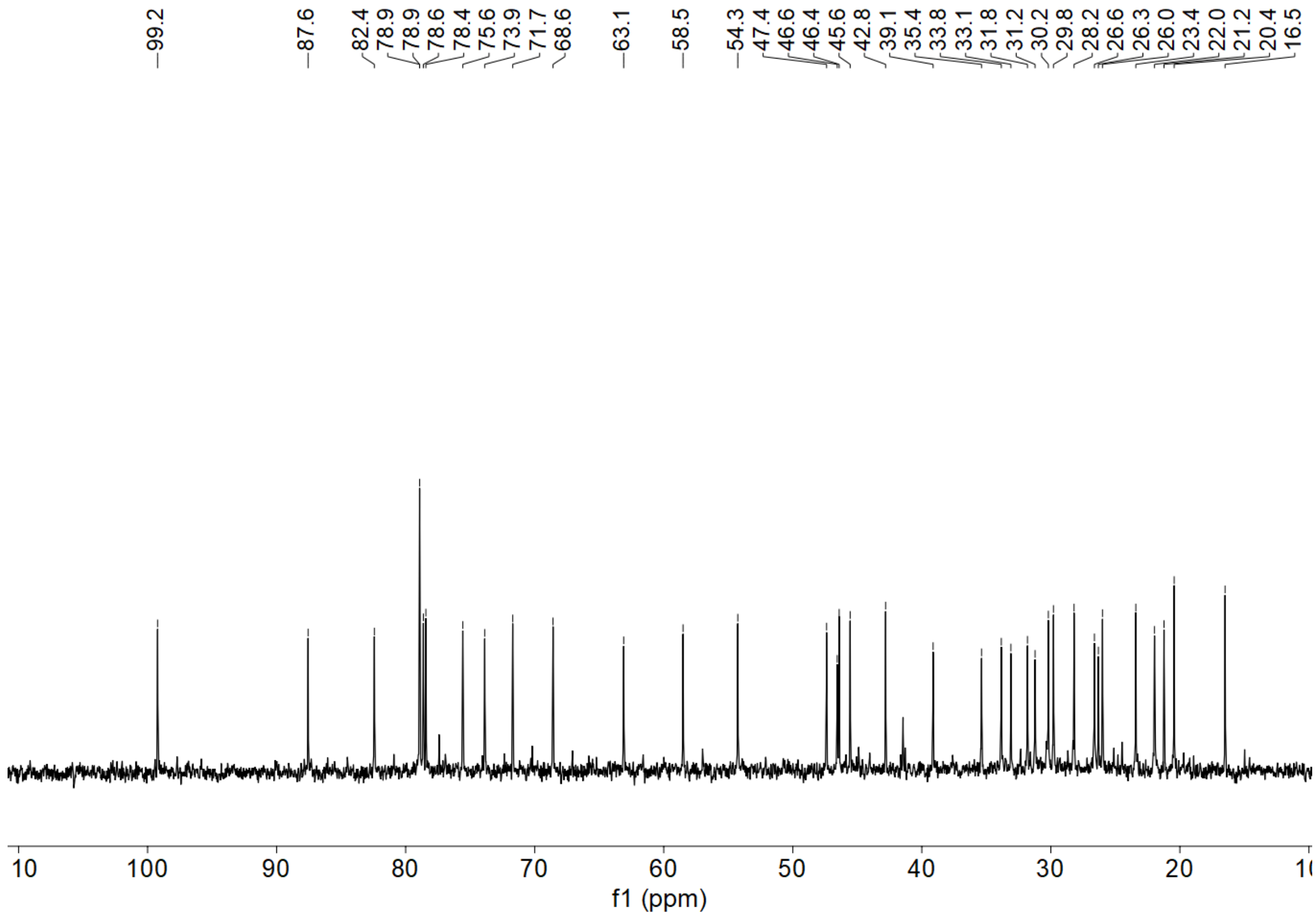


**Figure S60. $^{13}$C (125 MHz, pyridine-$d_5$) NMR spectrum of cycloastragenol 25-*O*-glucoside (46).**

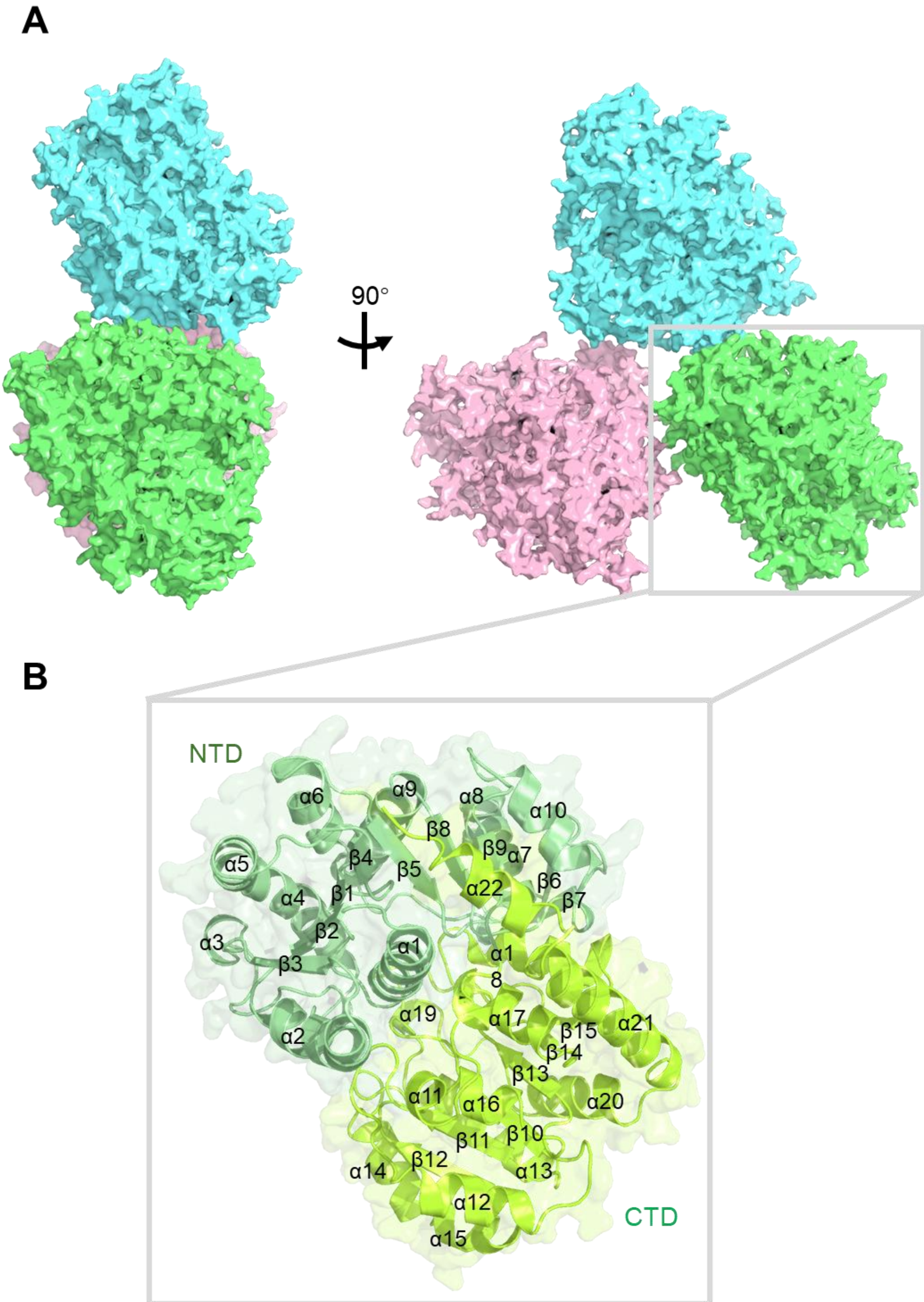


**Figure S61. Overview of SgUGT73AM30 crystal structure.** (**A**) A homotrimer crystal structure of SgUGT73AM30. (**B**) The monomeric protein structure consists of NTD domain and CTD domain, containing 22 α-helices and 15 β-sheets.

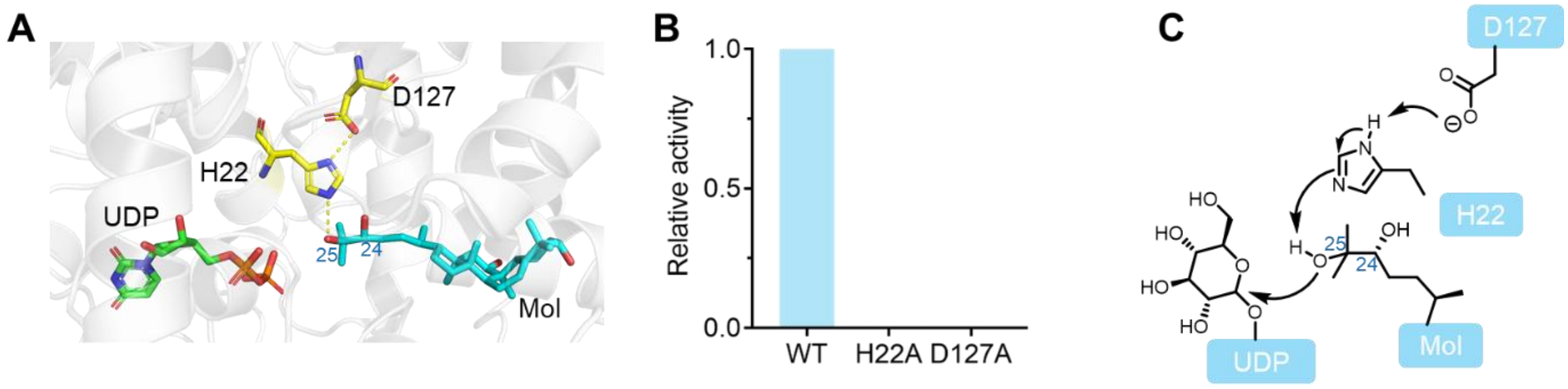


**Figure S62. H22-D126-aided deprotonation of C25-hydroxyl in mogrol (Mol, 1).**

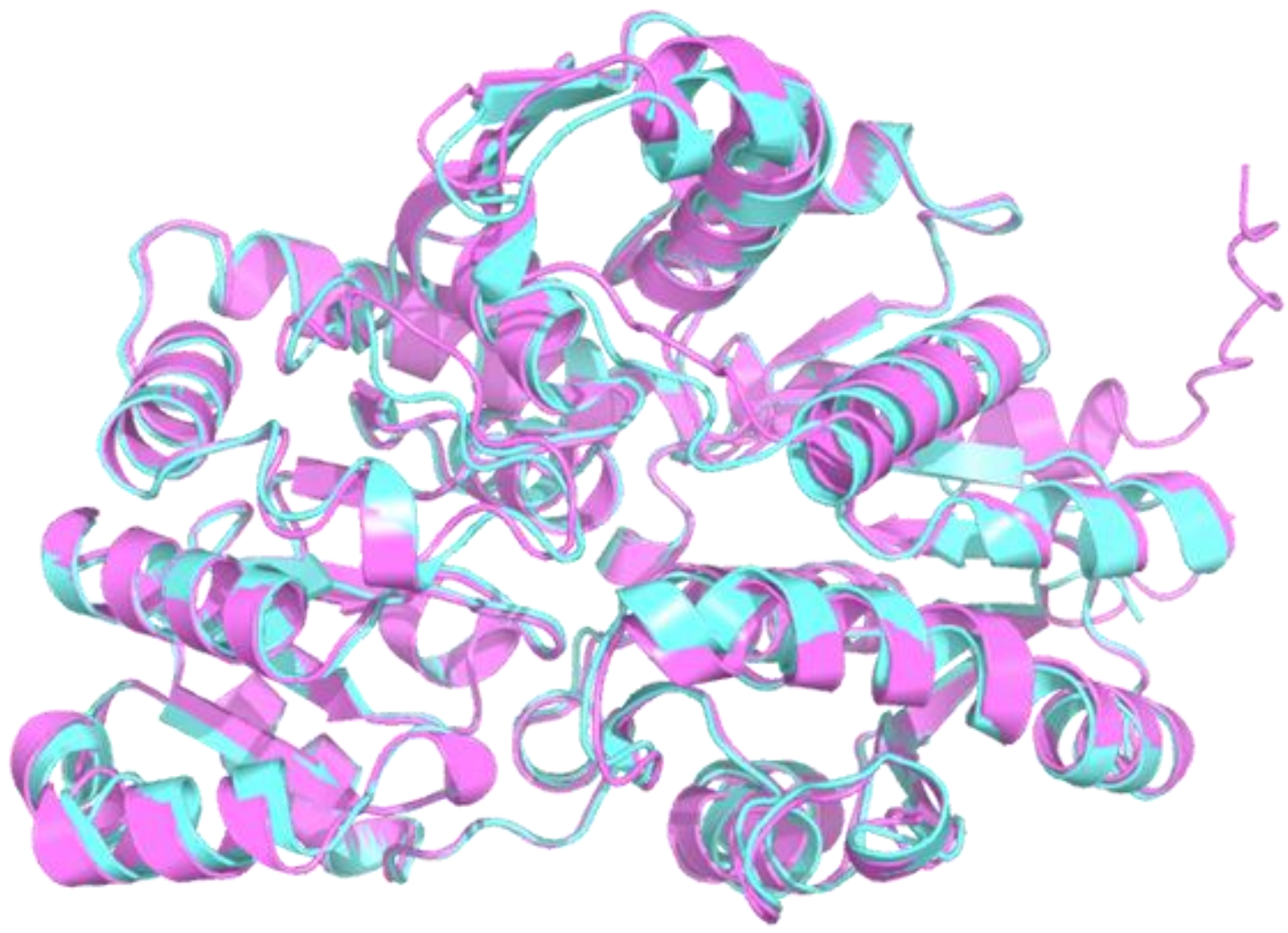

R.M.S.D. = 0.604 Å

**Figure S63. Comparison of the AlphaFold-predicted SgUGT73AM19 structure (purple) with the crystal structure of SgUGT73AM30 (cyan).**

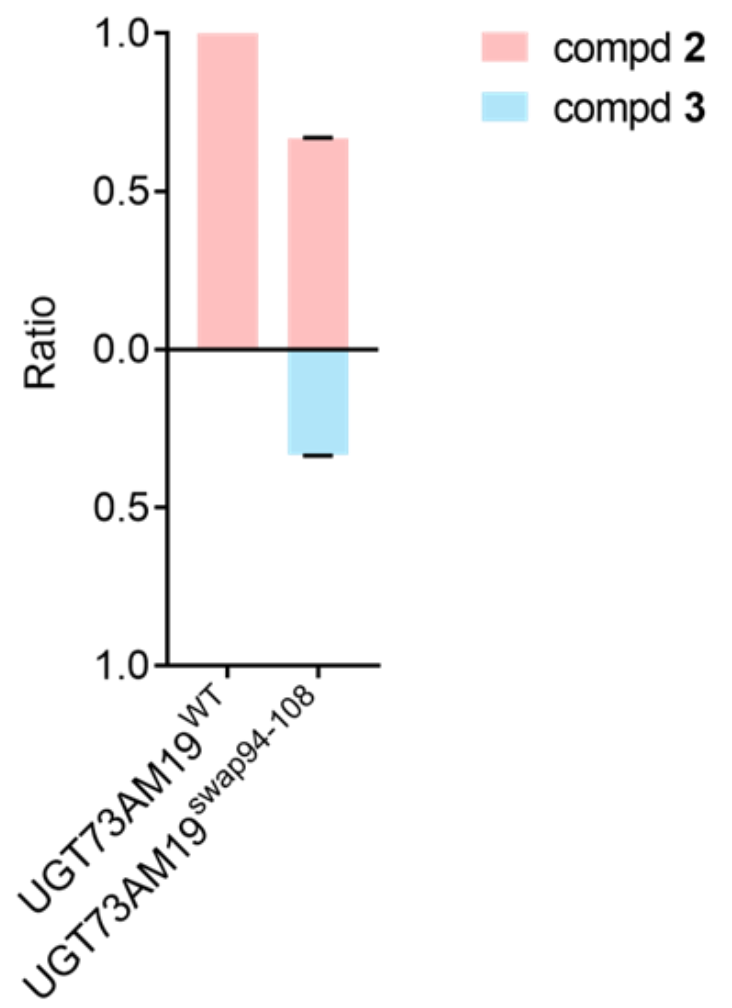


| Protein | Sequences |
| --- | --- |
| UGT73AM19$^{swap94-108}$ | MASPRHTPHFLLFPFMAQGHMIPMIDLARLLAQRGVIITIITTPHN<br>AARYHSVLARAIDSGLHIHVLQLQFPCKEGGLPEGCENVDLLPSL<br>**GSASTFFRATCLLYD**PSEKLFEELIPRPTCIISDMCLPWTMRIALK<br>YHVPRLVFYSLSCFFLLCMRSLKNNLALISSKSDSEFVTFSDLPD<br>PVEFLKSELPKSTDEDLVKFSYEMGEADRQSYGVILNLFEEMEP<br>KYLAEYEKERESPERVWCVGPVSLCNDNKLDKAERGNKASIDE<br>YKCIRWLDGQQPSSVVYVSLGSLCNLVTAQIIELGLGLEASKKPFI<br>WVIRRGNITEELQKWLVEYDFEEKIKGRGLVILGWAPQVLILSHP<br>AIGCFLTHCGWNSSIEGISAGVPMVTWPLFADQVFNEKLIVQILRI<br>GVSVGTETTMNWGEEEEKGVVVKREKVREAIEIVMDGDEREER<br>RERCKELAETAKRAIEEGGSSHRNLTMLIEDIIHGGGLSYEKGSC<br>R* |

**Figure S64. *In vitro* assay of SgUGT73AM19$^{swap94-108}$.** The resulting chimeric sequence is shown above, with the swapped segment highlighted in bold. Data are presented as the mean ± SD ($n$ = 3 biological replicates).

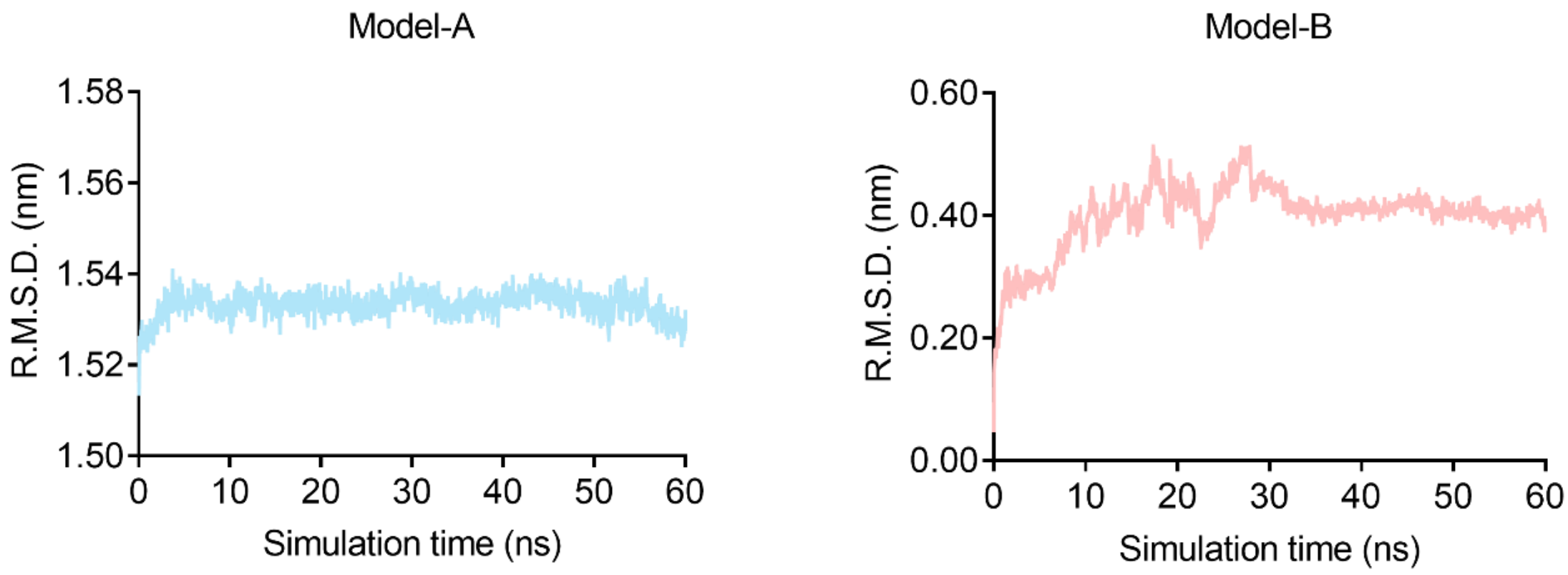


**Figure S65. The plot of R.M.S.D values over 60 ns for Model-A and Model-B.**

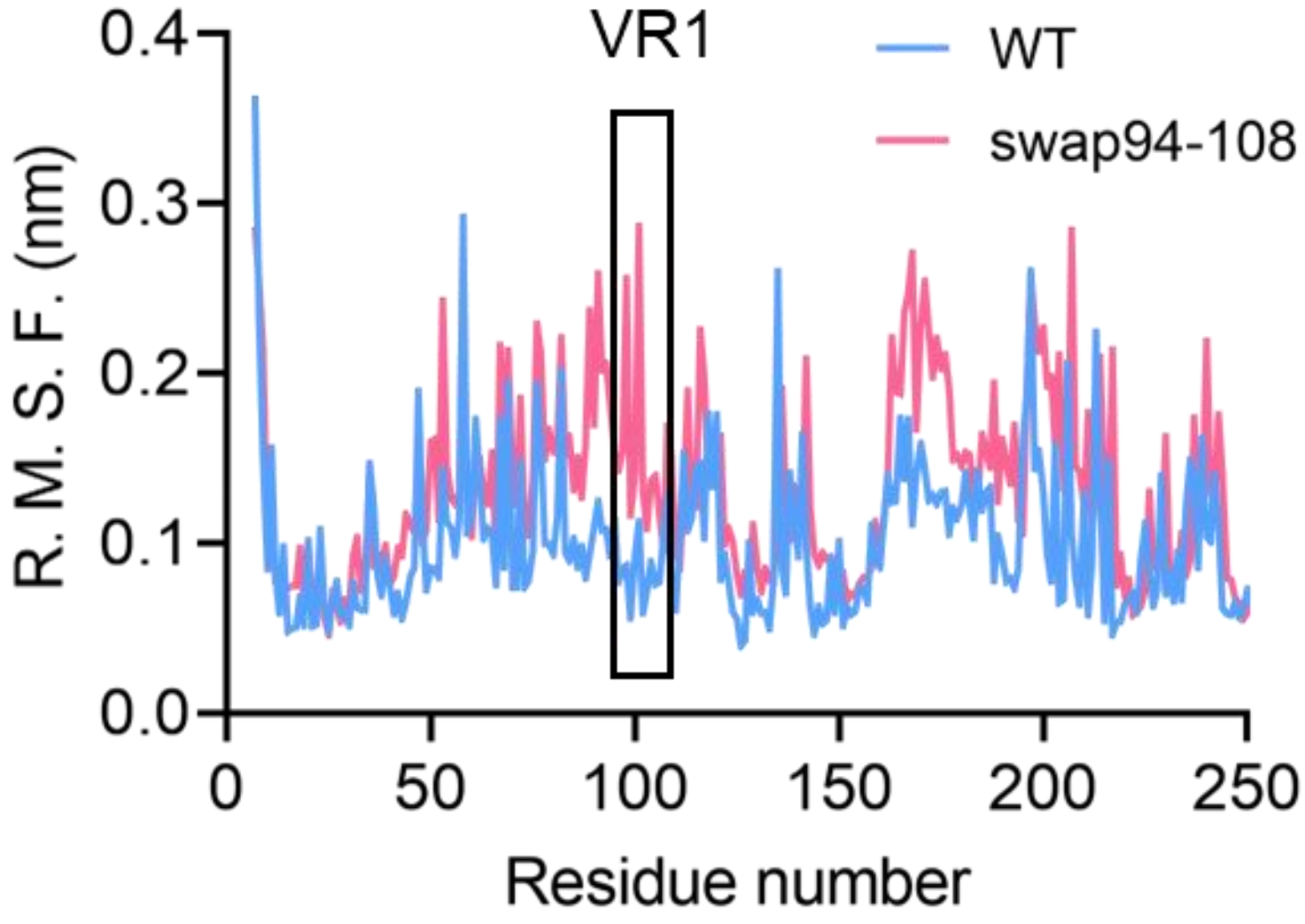


**Figure S66. The plot of R.S.M.F. values of NTD domain in Model-A and Model-B.**

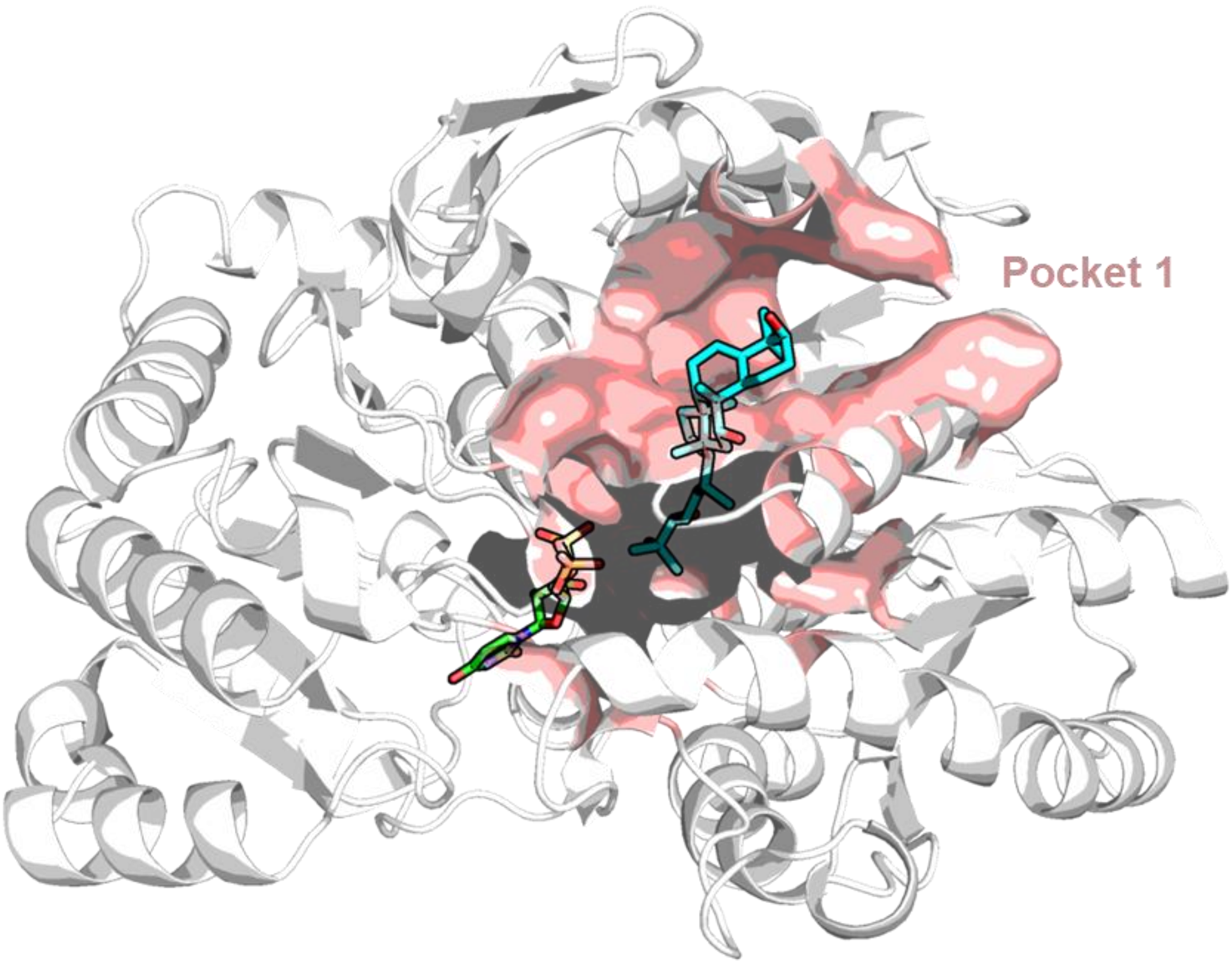


SgUGT73AM30-mogrol-UDP complex

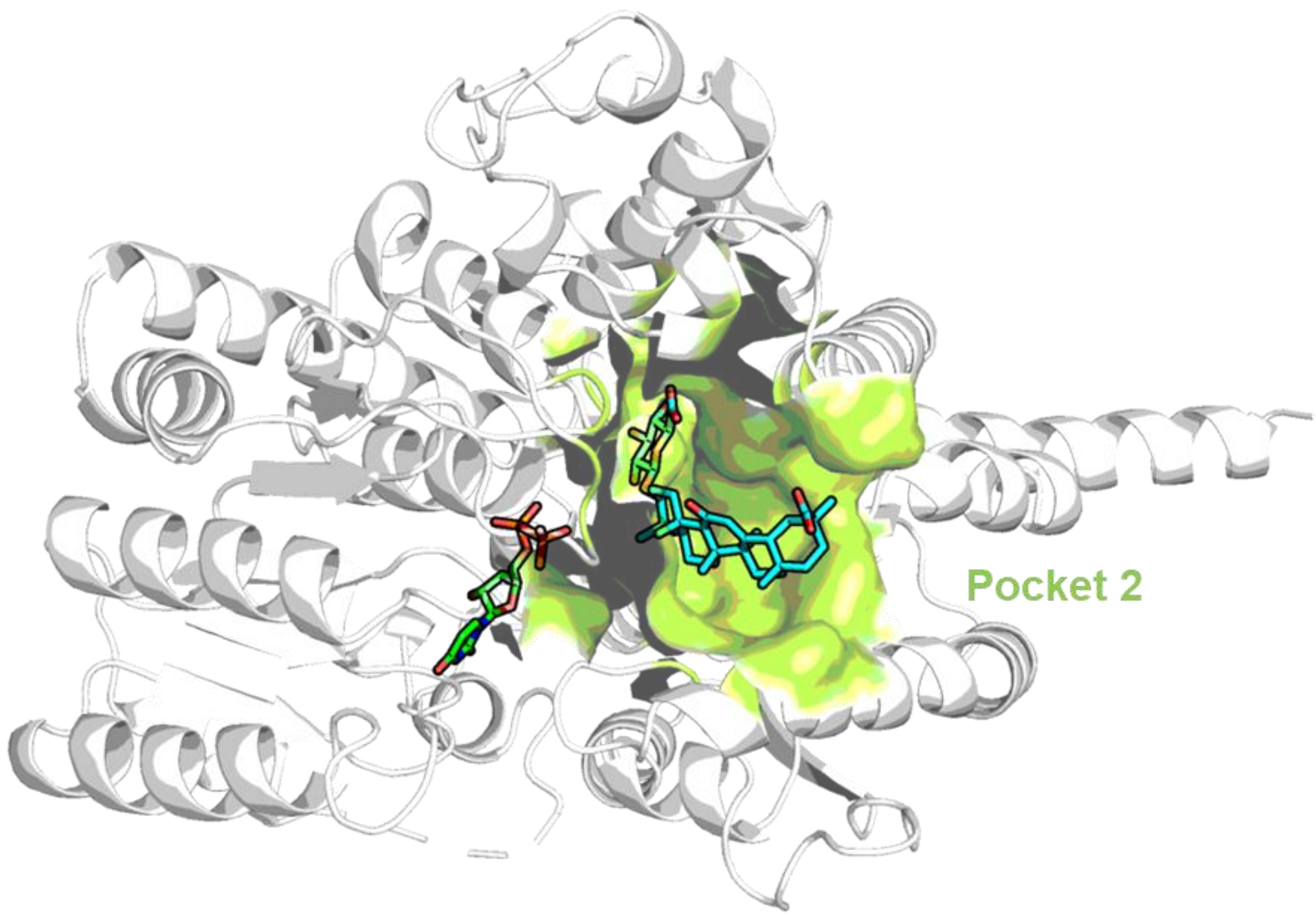


GuUGT73P12-glycyrrhetinic acid 3-*O*-monoglucuronide-UDP complex
(PDB ID: 7C2X)

**Figure S67. Structural divergence of UGT73 substrate-binding pockets.**

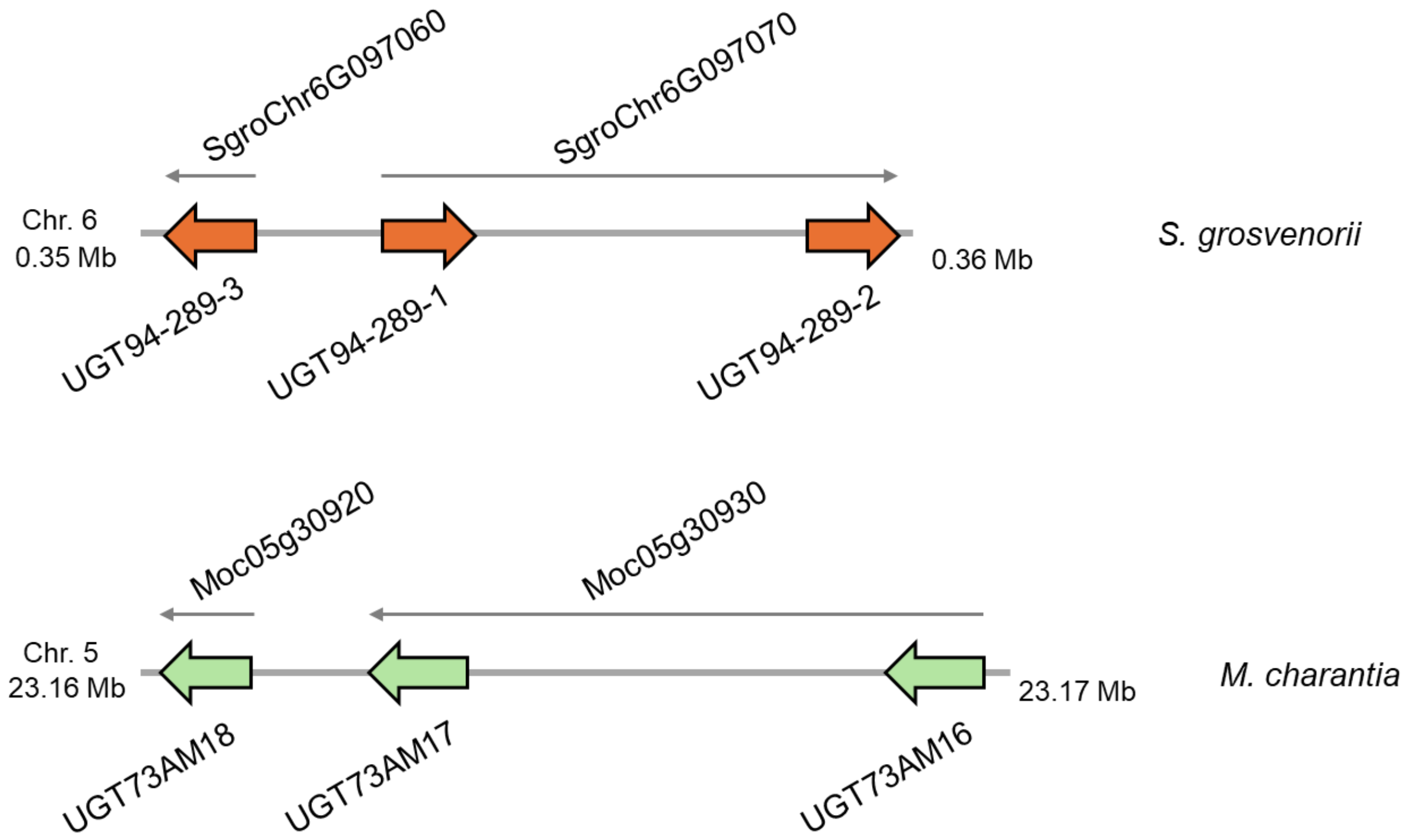


**Figure S68. Representative cases of genomic annotation discrepancy.** Gray arrows denote the originally annotated gene models, while colored arrows indicate the actual gene arrangements as revealed by manual curation.

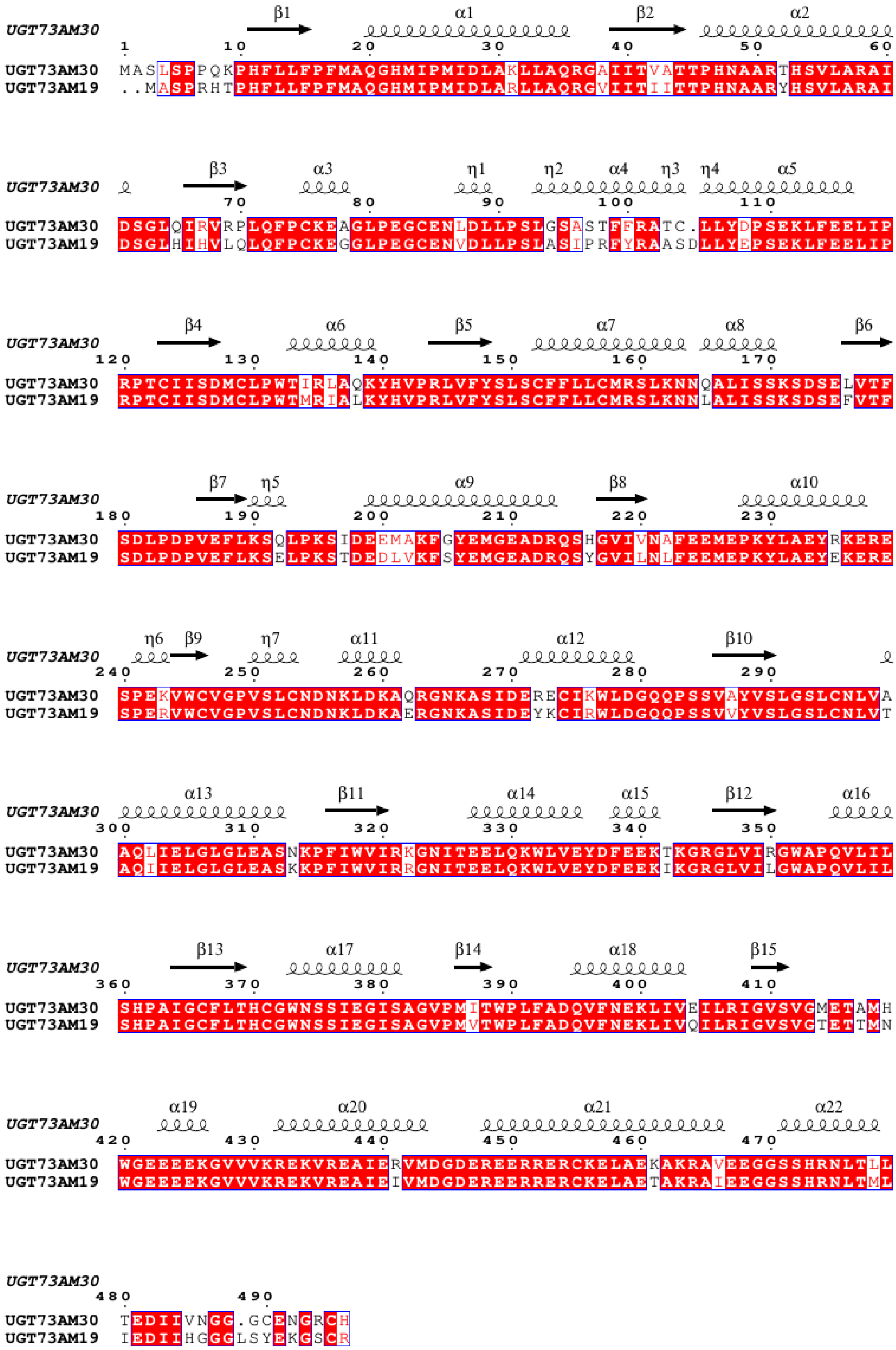


**Figure S69. Pairwise sequence alignment of SgUGT73AM30 and SgUGT73AM19.**

**Table S1. Statistics of different versions of *S. grosvenorii* genome**

| | *S. grosvenorii* genome assembly | | |
|---|---|---|---|
| | Version 1 | Version 2 | Version 3 |
| Pseudochromosomes | / | / | 14 |
| Total scaffolds | 12,772 | 4,128 | 42 |
| Assembly size (Mb) | 420.15 | 469.52 | 315.62 |
| Scaffold N50 (Mb) | 0.10 | 0.43 | 21.88 |
| Longest scaffold (Mb) | 0.80 | 7.68 | 34.40 |
| Contig N50 (Mb) | 0.03 | / | / |
| GC content (%) | 33.39 | 33.49 | 33.64 |
| Protein | Unpublished | 30,565 | 21,096 |
| BUSCO (%) | Unpublished | 89.2 | 98.7 |
| Reference | (*22*) | (*28*) | This study |

**Table S2. BUSCO evaluation o *S. grosvenorii* and *T. pustulata* gene annotation.**

| | Annotation BUSCO quality (%) | |
|---|---|---|
| | *S. grosvenorii* | *T. pustulata* |
| Complete BUSCOs | 98.9 | 91.6 |
| Complete Single-Copy BUSCOs | 97.6 | 90.6 |
| Complete Duplicated BUSCOs | 1.3 | 1.0 |
| Fragmented BUSCOs | 0.4 | 4.6 |
| Missing BUSCOs | 0.7 | 3.8 |
| Total BUSCO groups searched | 100.0 | 100.0 |

**Table S3. Distribution of UGT family genes in Cucurbitaceae species identified by HMM-based search.**

| | Bhis | Clan | Lsic | Cmos | Csat | Gpen | Hped | Lcyl | Mcha | Sedu | Sgro | Tang | Tpus |
|---|---|---|---|---|---|---|---|---|---|---|---|---|---|
| UGT94 | 4 | 6 | 9 | 5 | 6 | 13 | 4 | 8 | 8 | 12 | 7 | 5 | 6 |
| UGT93 | 3 | 3 | 3 | 5 | 3 | 2 | 0 | 4 | 3 | 2 | 2 | 3 | 3 |
| UGT92 | 1 | 1 | 2 | 2 | 2 | 2 | 1 | 2 | 2 | 2 | 2 | 2 | 1 |
| UGT91 | 6 | 5 | 6 | 7 | 8 | 11 | 5 | 9 | 4 | 10 | 7 | 6 | 7 |
| UGT90 | 1 | 2 | 2 | 2 | 1 | 1 | 1 | 3 | 1 | 1 | 2 | 1 | 3 |
| UGT89 | 3 | 3 | 3 | 1 | 1 | 1 | 3 | 1 | 2 | 1 | 4 | 2 | 1 |
| UGT87 | 2 | 3 | 4 | 3 | 2 | 4 | 3 | 3 | 2 | 3 | 2 | 4 | 2 |
| UGT86 | 0 | 1 | 1 | 2 | 1 | 1 | 1 | 1 | 1 | 1 | 1 | 1 | 1 |
| UGT85 | 6 | 11 | 11 | 13 | 13 | 6 | 5 | 6 | 7 | 11 | 8 | 11 | 7 |
| UGT83 | 2 | 3 | 3 | 2 | 0 | 1 | 1 | 5 | 2 | 3 | 5 | 1 | 2 |
| UGT82 | 1 | 1 | 1 | 1 | 1 | 1 | 1 | 1 | 1 | 2 | 1 | 1 | 1 |
| UGT79 | 3 | 2 | 3 | 3 | 5 | 2 | 5 | 3 | 4 | 4 | 4 | 4 | 5 |
| UGT78 | 1 | 1 | 2 | 2 | 2 | 1 | 2 | 2 | 2 | 1 | 3 | 3 | 1 |
| UGT76 | 2 | 3 | 3 | 4 | 4 | 9 | 4 | 6 | 6 | 7 | 4 | 6 | 2 |
| UGT74 | 19 | 21 | 20 | 30 | 23 | 30 | 24 | 30 | 28 | 26 | 27 | 32 | 20 |
| UGT73 | 14 | 16 | 17 | 13 | 13 | 30 | 7 | 14 | 22 | 19 | 24 | 16 | 15 |
| UGT72 | 5 | 5 | 4 | 11 | 4 | 5 | 4 | 5 | 5 | 7 | 8 | 5 | 4 |
| UGT71 | 8 | 5 | 7 | 5 | 8 | 20 | 8 | 2 | 7 | 8 | 6 | 8 | 5 |
| UGT709 | 5 | 5 | 7 | 6 | 4 | 10 | 6 | 9 | 6 | 8 | 7 | 7 | 3 |
| UGT708 | 1 | 1 | 1 | 1 | 1 | 1 | 1 | 1 | 1 | 1 | 1 | 1 | 1 |

**Table S4. Distribution of UGT-related orthogroups in Cucurbitaceae species identified by OrthoFinder analysis.**

| Orthogroup | Bhis | Clan | Lsic | Cmos | Csat | Gpen | Hped | Lcyl | Mcha | Sedu | Sgro | Tang | Tpus |
|---|---|---|---|---|---|---|---|---|---|---|---|---|---|
| OG00 | 2 | 5 | 6 | 6 | 8 | 2 | 2 | 2 | 2 | 1 | 2 | 4 | 4 |
| OG01 | 4 | 4 | 4 | 4 | 4 | 2 | 0 | 2 | 4 | 6 | 4 | 5 | 2 |
| OG02 | 3 | 5 | 7 | 3 | 4 | 0 | 1 | 0 | 3 | 4 | 7 | 3 | 0 |
| OG03 | 3 | 2 | 5 | 2 | 6 | 5 | 1 | 1 | 3 | 4 | 1 | 1 | 1 |
| OG04 | 1 | 2 | 2 | 2 | 1 | 3 | 1 | 3 | 1 | 5 | 2 | 2 | 1 |
| OG05 | 3 | 3 | 3 | 1 | 1 | 1 | 3 | 1 | 2 | 1 | 4 | 2 | 1 |
| OG06 | 2 | 2 | 0 | 2 | 2 | 2 | 2 | 4 | 1 | 2 | 1 | 4 | 1 |
| OG07 | 2 | 2 | 3 | 2 | 2 | 2 | 2 | 2 | 1 | 1 | 1 | 3 | 1 |
| OG08 | 1 | 1 | 2 | 5 | 1 | 0 | 3 | 4 | 2 | 1 | 1 | 1 | 1 |
| OG09 | 2 | 4 | 3 | 2 | 2 | 1 | 1 | 1 | 1 | 2 | 1 | 1 | 1 |
| OG10 | 2 | 1 | 2 | 4 | 1 | 2 | 1 | 1 | 1 | 1 | 3 | 1 | 1 |
| OG11 | 1 | 2 | 2 | 3 | 1 | 2 | 1 | 1 | 1 | 1 | 1 | 1 | 4 |
| OG12 | 2 | 1 | 2 | 2 | 2 | 0 | 1 | 2 | 4 | 1 | 2 | 1 | 0 |
| OG13 | 1 | 2 | 2 | 2 | 1 | 1 | 1 | 2 | 1 | 3 | 1 | 2 | 0 |
| OG14 | 1 | 1 | 1 | 1 | 1 | 1 | 2 | 3 | 2 | 0 | 2 | 2 | 2 |
| OG15 | 2 | 2 | 1 | 2 | 1 | 1 | 1 | 2 | 1 | 2 | 1 | 2 | 1 |
| OG16 | 2 | 1 | 2 | 2 | 1 | 0 | 2 | 2 | 0 | 2 | 2 | 2 | 1 |
| OG17 | 1 | 1 | 1 | 2 | 1 | 5 | 1 | 1 | 0 | 2 | 1 | 1 | 1 |
| OG18 | 1 | 1 | 1 | 1 | 1 | 2 | 1 | 2 | 3 | 2 | 1 | 2 | 0 |
| OG19 | 0 | 1 | 1 | 0 | 0 | 7 | 1 | 2 | 3 | 0 | 1 | 2 | 0 |
| OG20 | 2 | 2 | 2 | 2 | 2 | 0 | 0 | 2 | 1 | 1 | 1 | 2 | 1 |
| OG21 | 1 | 2 | 1 | 2 | 2 | 2 | 1 | 1 | 1 | 1 | 1 | 1 | 2 |
| OG22 | 0 | 1 | 1 | 1 | 0 | 0 | 1 | 2 | 0 | 5 | 2 | 3 | 1 |
| OG23 | 0 | 0 | 0 | 1 | 1 | 6 | 1 | 2 | 1 | 2 | 1 | 1 | 1 |
| OG24 | 2 | 2 | 1 | 1 | 1 | 1 | 2 | 1 | 2 | 1 | 1 | 1 | 1 |
| OG25 | 1 | 1 | 1 | 3 | 1 | 2 | 0 | 0 | 2 | 1 | 1 | 2 | 1 |
| OG26 | 1 | 0 | 2 | 0 | 1 | 8 | 0 | 0 | 0 | 0 | 0 | 2 | 2 |
| OG27 | 2 | 1 | 1 | 0 | 2 | 0 | 2 | 0 | 3 | 2 | 1 | 2 | 0 |
| OG28 | 1 | 1 | 1 | 1 | 1 | 2 | 2 | 1 | 1 | 1 | 1 | 2 | 1 |

| Orthogroup | Bhis | Clan | Lsic | Cmos | Csat | Gpen | Hped | Lcyl | Mcha | Sedu | Sgro | Tang | Tpus |
|---|---|---|---|---|---|---|---|---|---|---|---|---|---|
| OG29 | 1 | 1 | 1 | 3 | 1 | 0 | 0 | 2 | 2 | 1 | 1 | 1 | 2 |
| OG30 | 1 | 1 | 1 | 1 | 0 | 1 | 0 | 3 | 1 | 2 | 4 | 0 | 1 |
| OG31 | 1 | 1 | 1 | 2 | 0 | 3 | 1 | 1 | 1 | 2 | 1 | 1 | 1 |
| OG32 | 1 | 1 | 1 | 1 | 1 | 2 | 1 | 1 | 1 | 2 | 1 | 1 | 1 |
| OG33 | 1 | 1 | 1 | 0 | 1 | 2 | 2 | 1 | 1 | 1 | 1 | 1 | 1 |
| OG34 | 1 | 1 | 2 | 1 | 2 | 0 | 1 | 0 | 1 | 2 | 1 | 1 | 1 |
| OG35 | 0 | 1 | 1 | 1 | 0 | 3 | 1 | 1 | 2 | 2 | 1 | 0 | 1 |
| OG36 | 1 | 1 | 0 | 1 | 1 | 1 | 1 | 3 | 1 | 1 | 1 | 1 | 1 |
| OG37 | 1 | 1 | 1 | 1 | 1 | 1 | 1 | 1 | 1 | 1 | 1 | 2 | 1 |
| OG38 | 2 | 1 | 1 | 2 | 1 | 0 | 1 | 0 | 1 | 2 | 1 | 1 | 1 |
| OG39 | 0 | 0 | 0 | 2 | 0 | 0 | 2 | 2 | 0 | 5 | 1 | 2 | 0 |
| OG40 | 3 | 2 | 3 | 0 | 6 | 0 | 0 | 0 | 0 | 0 | 0 | 0 | 0 |
| OG41 | 1 | 2 | 2 | 1 | 0 | 0 | 1 | 2 | 1 | 1 | 1 | 1 | 1 |
| OG42 | 1 | 1 | 1 | 1 | 1 | 2 | 1 | 1 | 1 | 1 | 1 | 1 | 1 |
| OG43 | 1 | 1 | 1 | 1 | 1 | 1 | 1 | 1 | 1 | 2 | 1 | 1 | 1 |
| OG44 | 1 | 1 | 1 | 0 | 0 | 2 | 0 | 1 | 1 | 4 | 1 | 0 | 1 |
| OG45 | 1 | 1 | 1 | 1 | 1 | 1 | 0 | 0 | 1 | 2 | 1 | 2 | 1 |
| OG46 | 1 | 1 | 1 | 1 | 0 | 2 | 1 | 1 | 1 | 1 | 1 | 1 | 1 |
| OG47 | 1 | 1 | 1 | 1 | 1 | 1 | 2 | 1 | 0 | 1 | 1 | 1 | 1 |
| OG48 | 1 | 1 | 1 | 1 | 1 | 0 | 1 | 1 | 1 | 1 | 3 | 1 | 0 |
| OG49 | 0 | 0 | 0 | 1 | 1 | 2 | 1 | 1 | 1 | 2 | 1 | 2 | 1 |
| OG50 | 1 | 1 | 1 | 1 | 1 | 1 | 1 | 1 | 1 | 1 | 1 | 1 | 1 |
| OG51 | 1 | 2 | 2 | 0 | 1 | 0 | 0 | 1 | 1 | 1 | 2 | 1 | 1 |
| OG52 | 1 | 1 | 1 | 1 | 1 | 1 | 1 | 1 | 1 | 1 | 1 | 1 | 1 |
| OG53 | 1 | 1 | 1 | 1 | 1 | 1 | 1 | 1 | 1 | 1 | 1 | 1 | 1 |
| OG54 | 0 | 1 | 1 | 1 | 0 | 2 | 1 | 1 | 1 | 2 | 1 | 1 | 1 |
| OG55 | 0 | 1 | 1 | 2 | 1 | 1 | 1 | 1 | 1 | 1 | 1 | 1 | 1 |
| OG56 | 1 | 1 | 0 | 1 | 1 | 2 | 1 | 1 | 1 | 1 | 1 | 1 | 0 |
| OG57 | 0 | 0 | 2 | 1 | 1 | 0 | 0 | 0 | 1 | 2 | 0 | 2 | 3 |
| OG58 | 1 | 1 | 1 | 0 | 0 | 2 | 0 | 3 | 1 | 0 | 1 | 0 | 2 |
| OG59 | 1 | 1 | 1 | 2 | 1 | 1 | 0 | 0 | 1 | 1 | 1 | 1 | 1 |

| Orthogroup | Bhis | Clan | Lsic | Cmos | Csat | Gpen | Hped | Lcyl | Mcha | Sedu | Sgro | Tang | Tpus |
|---|---|---|---|---|---|---|---|---|---|---|---|---|---|
| OG60 | 1 | 1 | 0 | 0 | 0 | 1 | 0 | 4 | 1 | 2 | 1 | 0 | 1 |
| OG61 | 1 | 0 | 1 | 2 | 1 | 0 | 1 | 1 | 2 | 1 | 1 | 0 | 1 |
| OG62 | 0 | 0 | 0 | 1 | 1 | 2 | 1 | 1 | 1 | 2 | 0 | 2 | 1 |
| OG63 | 2 | 1 | 0 | 1 | 2 | 2 | 0 | 0 | 1 | 0 | 1 | 0 | 1 |
| OG64 | 0 | 2 | 0 | 2 | 1 | 0 | 1 | 0 | 1 | 1 | 1 | 1 | 1 |
| OG65 | 1 | 0 | 1 | 1 | 1 | 0 | 1 | 0 | 1 | 1 | 1 | 1 | 2 |
| OG66 | 0 | 1 | 1 | 1 | 1 | 0 | 1 | 1 | 1 | 2 | 1 | 1 | 0 |
| OG67 | 1 | 1 | 0 | 0 | 1 | 2 | 1 | 1 | 1 | 0 | 1 | 1 | 1 |
| OG68 | 0 | 0 | 0 | 1 | 1 | 4 | 1 | 1 | 1 | 0 | 1 | 1 | 0 |
| OG69 | 0 | 1 | 1 | 2 | 1 | 0 | 1 | 2 | 1 | 0 | 1 | 1 | 0 |
| OG70 | 0 | 1 | 1 | 1 | 0 | 1 | 1 | 1 | 1 | 1 | 1 | 1 | 1 |
| OG71 | 0 | 0 | 0 | 1 | 0 | 0 | 0 | 1 | 0 | 5 | 1 | 2 | 0 |
| OG72 | 0 | 0 | 1 | 0 | 2 | 0 | 1 | 1 | 1 | 1 | 1 | 1 | 1 |
| OG73 | 1 | 1 | 0 | 1 | 1 | 0 | 1 | 1 | 1 | 1 | 1 | 0 | 1 |
| OG74 | 1 | 1 | 1 | 1 | 1 | 1 | 1 | 1 | 1 | 0 | 0 | 1 | 0 |
| OG75 | 0 | 0 | 0 | 0 | 0 | 7 | 0 | 1 | 1 | 0 | 0 | 0 | 1 |
| OG76 | 1 | 0 | 0 | 0 | 0 | 2 | 1 | 1 | 1 | 1 | 1 | 1 | 1 |
| OG77 | 1 | 1 | 1 | 0 | 0 | 1 | 0 | 1 | 0 | 1 | 1 | 0 | 3 |
| OG78 | 0 | 0 | 1 | 1 | 1 | 0 | 1 | 1 | 1 | 0 | 2 | 2 | 0 |
| OG79 | 0 | 0 | 1 | 2 | 1 | 0 | 1 | 1 | 1 | 0 | 1 | 1 | 0 |
| OG80 | 0 | 0 | 0 | 0 | 0 | 3 | 0 | 2 | 2 | 0 | 1 | 0 | 1 |
| OG81 | 0 | 0 | 0 | 0 | 1 | 1 | 1 | 1 | 1 | 1 | 1 | 2 | 0 |
| OG82 | 0 | 1 | 1 | 1 | 1 | 0 | 1 | 0 | 1 | 1 | 0 | 1 | 0 |
| OG83 | 1 | 1 | 1 | 0 | 1 | 0 | 1 | 1 | 0 | 0 | 1 | 1 | 0 |
| OG84 | 0 | 0 | 0 | 3 | 0 | 0 | 0 | 2 | 0 | 1 | 1 | 1 | 0 |
| OG85 | 0 | 1 | 1 | 0 | 1 | 0 | 0 | 0 | 1 | 0 | 1 | 2 | 0 |
| OG86 | 0 | 0 | 1 | 1 | 1 | 0 | 0 | 1 | 1 | 0 | 1 | 1 | 0 |
| OG87 | 0 | 0 | 0 | 0 | 0 | 0 | 0 | 2 | 0 | 2 | 2 | 1 | 0 |
| OG88 | 0 | 0 | 0 | 0 | 0 | 1 | 0 | 1 | 1 | 0 | 1 | 0 | 2 |
| OG89 | 1 | 0 | 1 | 0 | 1 | 0 | 1 | 0 | 0 | 1 | 1 | 0 | 0 |
| OG90 | 0 | 0 | 0 | 0 | 0 | 0 | 2 | 0 | 1 | 1 | 1 | 1 | 0 |

| Orthogroup | Bhis | Clan | Lsic | Cmos | Csat | Gpen | Hped | Lcyl | Mcha | Sedu | Sgro | Tang | Tpus |
|---|---|---|---|---|---|---|---|---|---|---|---|---|---|
| OG91 | 0 | 0 | 0 | 0 | 0 | 1 | 0 | 1 | 2 | 0 | 1 | 0 | 1 |
| OG92 | 0 | 0 | 0 | 0 | 0 | 6 | 0 | 0 | 0 | 0 | 0 | 0 | 0 |
| OG93 | 0 | 0 | 0 | 1 | 0 | 0 | 1 | 1 | 1 | 1 | 0 | 0 | 1 |
| OG94 | 0 | 0 | 0 | 0 | 0 | 5 | 0 | 0 | 0 | 0 | 0 | 0 | 0 |
| OG95 | 0 | 0 | 0 | 0 | 0 | 1 | 0 | 0 | 1 | 0 | 1 | 0 | 1 |
| OG96 | 0 | 0 | 0 | 0 | 0 | 4 | 0 | 0 | 0 | 0 | 0 | 0 | 0 |
| OG97 | 0 | 0 | 0 | 0 | 0 | 2 | 0 | 0 | 1 | 0 | 1 | 0 | 0 |
| OG98 | 0 | 0 | 0 | 0 | 0 | 1 | 0 | 0 | 1 | 0 | 1 | 0 | 1 |
| OG99 | 0 | 0 | 0 | 0 | 0 | 0 | 0 | 1 | 1 | 0 | 1 | 0 | 1 |
| OG100 | 0 | 0 | 0 | 0 | 0 | 0 | 0 | 1 | 0 | 0 | 2 | 1 | 0 |
| OG101 | 0 | 0 | 0 | 0 | 0 | 1 | 0 | 0 | 0 | 0 | 1 | 0 | 1 |
| OG102 | 0 | 0 | 0 | 0 | 0 | 0 | 1 | 0 | 1 | 0 | 0 | 0 | 1 |
| OG103 | 1 | 0 | 0 | 1 | 0 | 0 | 1 | 0 | 0 | 0 | 0 | 0 | 0 |
| OG104 | 0 | 0 | 0 | 0 | 0 | 0 | 0 | 0 | 1 | 0 | 1 | 0 | 1 |
| OG105 | 0 | 0 | 0 | 0 | 0 | 3 | 0 | 0 | 0 | 0 | 0 | 0 | 0 |
| OG106 | 0 | 0 | 0 | 0 | 0 | 1 | 0 | 1 | 0 | 0 | 1 | 0 | 0 |
| OG107 | 0 | 0 | 0 | 1 | 0 | 0 | 0 | 0 | 1 | 0 | 1 | 0 | 0 |
| OG108 | 0 | 0 | 0 | 0 | 0 | 0 | 0 | 0 | 1 | 0 | 1 | 0 | 1 |
| OG109 | 0 | 0 | 0 | 0 | 0 | 1 | 0 | 0 | 0 | 0 | 1 | 1 | 0 |
| OG110 | 0 | 0 | 0 | 0 | 0 | 0 | 1 | 0 | 0 | 1 | 1 | 0 | 0 |
| OG111 | 0 | 0 | 0 | 0 | 0 | 2 | 0 | 0 | 0 | 0 | 0 | 0 | 0 |
| OG112 | 0 | 0 | 0 | 0 | 0 | 0 | 0 | 0 | 0 | 0 | 0 | 0 | 2 |
| OG113 | 0 | 0 | 1 | 1 | 0 | 0 | 0 | 0 | 0 | 0 | 0 | 0 | 0 |
| OG114 | 0 | 0 | 0 | 0 | 0 | 0 | 0 | 0 | 1 | 0 | 0 | 1 | 0 |

**Table S5. Genomic resources used in this study.**

| Species | Genome assembly | ID | Source |
|---|---|---|---|
| *Aquilegia coerulea* | Aquilegia_coerulea_v1 | GCA_002738505.1 | NCBI |
| *Arabidopsis thaliana* | TAIR10 | GCF_000001735.3 | NCBI |
| *Aralia elata* | Aralia elata genome and annotation | 10.5061/dryad.69p8cz937 | Dryad Digital Repository |
| *Aristolochia fimbriata* | ASM1984555v1 | GCA_019845555.1 | NCBI |
| *Begonia darthvaderiana* | ASM2243294v1 | GCA_022432945.1 | NCBI |
| *Begonia loranthoides* | ASM2243306v1 | GCA_022433065.1 | NCBI |
| *Begonia masoniana* | ASM2243297v1 | GCA_022432975.1 | NCBI |
| *Begonia peltatifolia* | ASM2243305v1 | GCA_022433055.1 | NCBI |
| *Beta vulgaris* | EL10.2 | GCF_026745355.1 | NCBI |
| *Cannabis sativa* | ASM2916894v1 | GCF_029168945.1 | NCBI |
| *Centella asiatica* | / | GWHFIFH00000000.1 | CNCB |
| *Citrullus lanatus* | PI_254622 | PI_254622_v1 | Watermelon Genome Database (http://www.watermelondb.cn) |
| *Coffea arabica* | Coffea_Arabica_ET-39_HiFi | GCF_036785885.1 | NCBI |
| *Coptis chinensis* | ASM1568090v1 | GCA_015680905.1 | NCBI |
| *Coriaria nepalensis* | / | 10.6084/m9.figshare.22263274 | figshare |
| *Corylus avellana* | CavTom2PMs-1.0 | GCF_901000735.1 | NCBI |
| *Cucumis sativus* | Chinese Long_v3 | / | CuGenDBv2 |
| *Cucurbita moschata* | Rifu_v1 | / | CuGenDBv2 |
| *Cucurbita pepo* | MU-CU-16_v4.1 | / | CuGenDBv2 |
| *Cyclocarya paliurus* | ASM2985693v1 | GCA_029856935.1 | NCBI |
| *Datisca glomerata* | ASM325502v1 | GCA_003255025.1 | NCBI |
| *Daucus carota* | DH1_v3.0 | GCF_001625215.2 | NCBI |

| Species | Genome assembly | ID | Source |
|---|---|---|---|
| *Glycine max* | Glycine_max_v4.0 | GCF_000004515.6 | NCBI |
| *Gynostemma pentaphyllum* | JGL_1.0 | GCA_020536105.1 | Dr. Ding Huang |
| *Herpetospermum pedunculosum* | / | 10.6084/m9.figshare.21626153.v2 | figshare |
| *Juglans regia* | Walnut_2.0 | GCF_001411555.2 | NCBI |
| *Momordica charantia* | OHB3-1_v2 | / | CuGenDBv2 |
| *Nymphaea colorata* | ASM883128v2 | GCF_008831285.2 | NCBI |
| *Oryza sativa* | IRGSP-1.0 | GCA_001433935.1 | Ensembl Plants |
| *Papaver somniferum* | ASM357369v1 | GCF_003573695.1 | NCBI |
| *Persea americana* | XTBG_Pame_1.0 | GCA_051132755.1 | NCBI |
| *Prunus persica* | Prunus_persica_NCBIv2 | GCF_000346465.2 | NCBI |
| *Saponaria officinalis* | Saponaria_officinalis_var_Norfolk2_v1.0 | GCA_040167595.1 | NCBI |
| *Sicyos edulis* | Chayote | / | CuGenDBv2 |
| *Siraitia grosvenorii* | / | / | this study |
| *Stevia rebaudiana* | / | 10.6084/m9.figshare.14169491 | figshare |
| *Thladiantha pustulata* | ASM4400753v1 | GCA_044007535.1 | NCBI |
| *Trichosanthes anguina* | Snakegourd | / | CuGenDBv2 |
| *Vitis vinifera* | ASM3070453v1 | GCF_030704535.1 | NCBI |

**Table S6. UGT73 subfamily proteins in syntenic UGT73 tandem array.**

| Species | Locations | UGT73AT/FXs | UGT73AMs | UGT73GBs | Other UGT73s |
|---|---|---|---|---|---|
| *Begonia masoniana* | Chr. 4<br>(52.09-52.11 Mb) | × | × | × | BmUGT73GF5<br>BmUGT73GF6 |
| *Gynostemma pentaphyllum* | CM100508.1 (28.11-28.23 Mb) | GpUGT73FX1 | GpUGT73AM10<br>GpUGT73AM11<br>GpUGT73AM12<br>GpUGT73AM13<br>GpUGT73AM14 | × | × |
| *Thladiantha pustulata* | JBINJJ010000016 (9.83-9.86 Mb) | TpUGT73AT5 | TpUGT73AM28<br>TpUGT73AM29 | TpUGT73GB2 | × |
| *Siraitia grosvenorii* | Chr. 2<br>(2.09-2.15 Mb) | SgUGT73AT4 | SgUGT73AM1<br>SgUGT73AM2<br>SgUGT73AM19<br>SgUGT73AM20<br>SgUGT73AM21<br>SgUGT73AM22<br>SgUGT73AM30 | SgUGT73GB1 | × |
| *Momordica charantia* | Chr. 5<br>(23.14-23.22 Mb) | McUGT73AT6<br>McUGT73AT7 | McUGT73AM15<br>McUGT73AM16<br>McUGT73AM17<br>McUGT73AM18 | × | × |
| *Cucumis sativus* | Chr. 3<br>(28.11-28.23 Mb) | CsUGT73AT1 | CsUGT73AM3<br>CsUGT73AM7<br>CsUGT73AM8<br>CsUGT73AM9 | × | × |

**Table S7. Plasmids used in this study.**

| Plasmids | Resistance | Description | Usage | Source |
|---|---|---|---|---|
| pET-28a(+) | Kan | Protein expression vector | Plasmid construction | Novagen (U.S.A.) |
| pUGT73AM1 | Kan | pET-28a-His-SgUGT73AM1 | Candidates validation | this study |
| pUGT73AM2 | Kan | pET-28a-His-SgUGT73AM2 | Candidates validation | this study |
| pUGT73AM19 | Kan | pET-28a-His-SgUGT73AM19 | Candidates validation | this study |
| pUGT73AM20 | Kan | pET-28a-His-SgUGT73AM20 | Candidates validation | this study |
| pUGT73AM21 | Kan | pET-28a-His-SgUGT73AM21 | Candidates validation | this study |
| pUGT73AM22 | Kan | pET-28a-His-SgUGT73AM22 | Candidates validation | this study |
| pUGT73AM30 | Kan | pET-28a-His-SgUGT73AM30 | Candidates validation | this study |
| pUGT73AT4 | Kan | pET-28a-His-SgUGT73AT4 | Candidates validation | this study |
| pUGT73GB1 | Kan | pET-28a-His-SgUGT73GB1 | Candidates validation | this study |
| pUGT73AM10 | Kan | pET-28a-His-GpUGT73AM10 | Candidates validation | this study |
| pUGT73AM11 | Kan | pET-28a-His-GpUGT73AM11 | Candidates validation | this study |
| pUGT73AM12 | Kan | pET-28a-His-GpUGT73AM12 | Candidates validation | this study |
| pUGT73AM13 | Kan | pET-28a-His-GpUGT73AM13 | Candidates validation | this study |
| pUGT73AM14 | Kan | pET-28a-His-GpUGT73AM14 | Candidates validation | this study |
| pUGT73FX1 | Kan | pET-28a-His-GpUGT73FX1 | Candidates validation | this study |
| pUGT73AM15 | Kan | pET-28a-His-McUGT73AM15 | Candidates validation | this study |
| pUGT73AM16 | Kan | pET-28a-His-McUGT73AM16 | Candidates validation | this study |
| pUGT73AM17 | Kan | pET-28a-His-McUGT73AM17 | Candidates validation | this study |
| pUGT73AM18 | Kan | pET-28a-His-McUGT73AM18 | Candidates validation | this study |
| pUGT73AT6 | Kan | pET-28a-His-McUGT73AT6 | Candidates validation | this study |
| pUGT73AT7 | Kan | pET-28a-His-McUGT73AT7 | Candidates validation | this study |

| Plasmids | Resistance | Description | Usage | Source |
|---|---|---|---|---|
| pUGT73AM28 | Kan | pET-28a-His-TpUGT73AM28 | Candidates validation | this study |
| pUGT73AM29 | Kan | pET-28a-His-TpUGT73AM29 | Candidates validation | this study |
| pUGT73AT5 | Kan | pET-28a-His-TpUGT73AT5 | Candidates validation | this study |
| pUGT73GB2 | Kan | pET-28a-His-TpUGT73GB2 | Candidates validation | this study |
| pUGT73AM30opt | Kan | pET-28a-His-SgUGT73AM30opt | Large-scale protein purification | this study |
| pUGT73AM30$^{L87V}$ | Kan | pET-28a-His-SgUGT73AM30opt-L87V | Mutants assessment | this study |
| pUGT73AM30$^{F100Y}$ | Kan | pET-28a-His-SgUGT73AM30opt-F100Y | Mutants assessment | this study |
| pUGT73AM30$^{M201L}$ | Kan | pET-28a-His-SgUGT73AM30opt-M201L | Mutants assessment | this study |
| pUGT73AM30$^{A202V}$ | Kan | pET-28a-His-SgUGT73AM30opt-A202V | Mutants assessment | this study |
| pUGT73AM30$^{G205S}$ | Kan | pET-28a-His-SgUGT73AM30opt-G205S | Mutants assessment | this study |
| pUGT73AM30$^{quard}$ | Kan | pET-28a-His-SgUGT73AM30opt-L87V/F100Y/M201L/A202V | Mutants assessment | this study |
| pUGT73AM30$^{swapVR1}$ | Kan | pET-28a-His-SgUGT73AM30opt-swap94-108 | Mutants assessment | this study |
| pUGT73AM30$^{swapVR2}$ | Kan | pET-28a-His-SgUGT73AM30opt-swap192-205 | Mutants assessment | this study |
| pUGT73AM30$^{swapVR1/VR2}$ | Kan | pET-28a-His-SgUGT73AM30opt-swap192-205/swap192-205 | Mutants assessment | this study |
| pUGT73AM30$^{swap94-100}$ | Kan | pET-28a-His-SgUGT73AM30opt-swap94-100 | Mutants assessment | this study |
| pUGT73AM30$^{swap101-108}$ | Kan | pET-28a-His-SgUGT73AM30opt-swap101-108 | Mutants assessment | this study |
| pUGT73AM30$^{swapVR1/mono}$ | Kan | pET-28a-His-SgUGT73AM30opt-swap94-108-L87V | Mutants assessment | this study |
| pUGT73AM30$^{swapVR1/di}$ | Kan | pET-28a-His-SgUGT73AM30opt-swap94-108-L87V/M201L | Mutants assessment | this study |
| pUGT73AM30$^{swapVR1/tri}$ | Kan | pET-28a-His-SgUGT73AM30opt-swap94-108-L87V/M201L/A202V | Mutants assessment | this study |
| pUGT73AM19$^{swapVR1}$ | Kan | pET-28a-His-SgUGT73AM19-swap92-107 | Mutants assessment | this study |

**Table S8. Primers used in this study.**

| UGT constructs | Primers | Sequences (5' to 3') |
|---|---|---|
| pUGT73AM1 | AM1-F | ATCATCATCACAGCAGCGGCATGCTGATGCACGCTCTCAC |
| | AM1-R | CGGAGCTCGAATTCGGATCCTCAATTAACTATAGTAGGAAAATCATCAGCATCATCC |
| pUGT73AM2 | AM2-F | ATCATCATCACAGCAGCGGCATGGATTCCCCACCCCACA |
| | AM2-R | CGGAGCTCGAATTCGGATCCTCACAACCTCCTCAATGAAGTACCTATATC |
| pUGT73AM19 | AM19-F | ATCATCATCACAGCAGCGGCATGGCTTCTCCTCGCCACAC |
| | AM19-R | CGGAGCTCGAATTCGGATCCTCAGCGACAACTTCCTTTCTCATAA |
| pUGT73AM20 | AM20-F | ATCATCATCACAGCAGCGGCATGGATTCCCCACGCCAC |
| | AM20-R | CGGAGCTCGAATTCGGATCCTCAATCAGCAGTGTGAGGTTTCGA |
| pUGT73AM21 | AM21-F | ATCATCATCACAGCAGCGGCATGGATTCCTCACCCCAAAAACC |
| | AM21-R | CGGAGCTCGAATTCGGATCCCTAACAGTCACAACCTCCTCCTCG |
| pUGT73AM22 | AM22-F | ATCATCATCACAGCAGCGGCATGGATTCCCCACCCCAAAAAC |
| | AM22-R | CGGAGCTCGAATTCGGATCCTTAACTAATAATAATCAGATAAAACTTCCATTCTGACAA |
| pUGT73AM30 | AM30-F | ATCATCATCACAGCAGCGGCATGGCTTCCCTTTCCCCAC |
| | AM30-R | CGGAGCTCGAATTCGGATCCTTAATGGCATCTTCCATTCTCACAACC |
| pUGT73AT4 | AT4-F | ATCATCATCACAGCAGCGGCATGGCTTCCGAATCGAAACCAC |
| | AT4-R | CGGAGCTCGAATTCGGATCCCTAGAATCTGCTTGATGAATGATGCATGATG |
| pUGT73GB1 | GB1-F | ATCATCATCACAGCAGCGGCATGGGCGCTGCTCAGC |
| | GB1-R | CGGAGCTCGAATTCGGATCCCTAAGTTCGCAGGATGTTTGATCC |
| pUGT73AM10 | AM10-F | ATCATCATCACAGCAGCGGCATGGCTTCCCAATGCCTTG |
| | AM10-R | CGGAGCTCGAATTCGGATCCTCAGCAGGTTCCATTAACACAGCC |
| pUGT73AM11 | AM11-F | ATCATCATCACAGCAGCGGCATGGCTTCCCAAAACCACAAG |
| | AM11-R | CGGAGCTCGAATTCGGATCCTCAAAAGGTTCCATTATCACAGCTTG |
| pUGT73AM12 | AM12-F | ATCATCATCACAGCAGCGGCATGGCTTCCCAACACTACAAGC |
| | AM12-R | CGGAGCTCGAATTCGGATCCCTAATCACAGCTTGCTTGATGAGTAAAATC |
| pUGT73AM13 | AM13-F | ATCATCATCACAGCAGCGGCATGGCTTCCCAACACTACAAGC |
| | AM13-R | CGGAGCTCGAATTCGGATCCCTAATCACAGCTTGCTTGATGAGTAAAATC |
| pUGT73AM14 | AM14-F | ATCATCATCACAGCAGCGGCATGGCTTCCCTACACCACAAG |
| | AM14-R | CGGAGCTCGAATTCGGATCCTCAGCAGGTTCCATTATCACAGCTTG |
| pUGT73FX1 | FX1-F | ATCATCATCACAGCAGCGGCATGGCTTCCATTTCCCATTCTC |
| | FX1-R | CGGAGCTCGAATTCGGATCCTCAGAAGTTAGGCCGACTCCAG |
| pUGT73AM15 | AM15-F | ATCATCATCACAGCAGCGGCATGGCCATGGCTTCCCC |
| | AM15-R | CGGAGCTCGAATTCGGATCCTCATGAACAGTTCCCATTCTGACAAC |
| pUGT73AM16 | AM16-F | ATCATCATCACAGCAGCGGCATGGCCACGGCAATGGC |
| | AM16-R | CGGAGCTCGAATTCGGATCCTCAACAGCTTCCATTCTCGCAAC |
| pUGT73AM17 | AM17-F | ATCATCATCACAGCAGCGGCATGGCATCCCCTCACTTTCTTC |

| UGT constructs | Primers | Sequences (5' to 3') |
| --- | --- | --- |
| | AM17-R | CGGAGCTCGAATTCGGATCCTCAGCAGTTTCCATTGTCACAAC |
| pUGT73AM18 | AM18-F | ATCATCATCACAGCAGCGGCATGGCTTCCCCTCACTTTGTG |
| | AM18-R | CGGAGCTCGAATTCGGATCCTCATGATGATCCTCCATTGATGTGG |
| pUGT73AT6 | AT6-F | ATCATCATCACAGCAGCGGCATGGCGCCCCACTTCC |
| | AT6-R | CGGAGCTCGAATTCGGATCCCTAAGCCGGAACAAAATGTTGTTG |
| pUGT73AT7 | AT7-F | ATCATCATCACAGCAGCGGCATGGCTTCGGAAAACGCAGC |
| | AT7-R | CGGAGCTCGAATTCGGATCCCTAAATTTTATTCGGGCCAGAATGTCC |
| pUGT73AM28 | AM28-F | ATCATCATCACAGCAGCGGCATGGCCATGGCTTCTCC |
| | AM28-R | CGGAGCTCGAATTCGGATCCTCAGCAGGAGCTTCCATTTTCAC |
| pUGT73AM29 | AM29-F | ATCATCATCACAGCAGCGGCATGCCTTCCCCCCGAAG |
| | AM29-R | CGGAGCTCGAATTCGGATCCTCAGCAGCTTCCATTCTCAAATCC |
| pUGT73AT5 | AT5-F | ATCATCATCACAGCAGCGGCATGGCTTCCCAACCCGAAG |
| | AT5-R | CGGAGCTCGAATTCGGATCCTCAAACAGGAGCAAAAGTGAATCCAG |
| pUGT73GB2 | GB2-F | ATCATCATCACAGCAGCGGCATGGACGCTGCTCGGC |
| | GB2-R | CGGAGCTCGAATTCGGATCCTTACAATCGATGCCGATTTCTTTTCAC |
| pUGT73AM30$^{\text{L87V}}$ | L87V-F | GCGAAAACGTGGATCTGCTCCCATCCCTG |
| | L87V-R | GAGCAGATCCACGTTTTCGCAGCCCTCCG |
| pUGT73AM30$^{\text{F100Y}}$ | F100Y-F | AGCGCTAGCACCTTTTATCGCGCTACCTGTCTGC |
| | F100Y-R | AAAGGTGCTAGCGCTACCCA |
| pUGT73AM30$^{\text{M201L}}$ | M201L-F | CGAGGAACTGGCGAAATTCGGTTATGAGATGG |
| | M201L-R | TTTCGCCAGTTCCTCGTCGATGCTTTTGG |
| pUGT73AM30$^{\text{A202V}}$ | A202V-F | GGAAATGGTGAAATTCGGTTATGAGATGGGTG |
| | A202V-R | GAATTTCACCATTTCCTCGTCGATGCT |
| pUGT73AM30$^{\text{G205S}}$ | G205S-F | GAAATTCTCCTATGAGATGGGTGAGGCAGA |
| | G205S-R | CTCATAGGAGAATTTCGCCATTTCCTCGT |
| pUGT73AM30$^{\text{swapVR1}}$ | swapVR1-F | ATCACTTGCTGCTCTGTAGAATCTGGGTATGGAAGCCAGGGATGGGAGCAGATCC |
| | swapVR1-R | CAGAGCAGCAAGTGATCTCCTTTACGAACCGTCGGAGAAGTTGTTCG |
| pUGT73AM30$^{\text{swapVR2}}$ | swapVR2-F | GTCTTCATCGGTGGATTTAGGTAGCTCAGATTTCAGGAATTCTACCGGAT |
| | swapVR2-R | ATCCACCGATGAAGACTTGGTGAAGTTTAGTTATGAGATGGGTGAGGCAG |
| pUGT73AM30$^{\text{swap94-100}}$ | swap94-100-F | CTTCCATACCCAGATTCTACCGCGCTACCTGTCTGC |
| | swap94-100-R | TAGAATCTGGGTATGGAAGCCAGGGATGGGAGCAGATC |
| pUGT73AM30$^{\text{swap101-108}}$ | swap101-108-F | CAGCAAGTGATCTCCTTTACGAACCGTCGGAGAAGTTGTTC |
| | swap101-108-R | AGGAGATCACTTGCTGCTCTGAAAAAGGTGCTAGCGCT |
| pUGT73AM19$^{\text{swapVR1}}$ | swap92-107-F | GACAGGTAGCGCGGAAAAAGGTGCTAGCGCTACCAAGTGAAGGTAGCAAGTCCAC |
| | swap92-107-R | TTCCGCGCTACCTGTCTGCTATACGACCCATCTGAAAAACTGTTTGAGGA |

Note: homologous arms are underlined.

**Table S9. Statistical parameters for structural analysis of SgUGT73AM30.**

| Data ID: (UGT73AM30_Mogrol) | Overall (outer shell) |
|---|---|
| Wavelength | 0.97918 |
| Resolution range | 26.48-2.6 (2.693-2.6) |
| Space group | P 32 |
| Unit cell | 105 105 122.661 90 90 120 |
| Total reflections | 265750 (26875) |
| Unique reflections | 46443 (4639) |
| Multiplicity | 5.7 (5.8) |
| Completeness (%) | 99.84 (99.89) |
| Mean I/sigma(I) | 9.31 (2.08) |
| Wilson B-factor | 48.02 |
| R-merge | 0.1632 (1.528) |
| R-meas | 0.1798 (1.681) |
| R-pim | 0.07489 (0.6951) |
| CC1/2 | 0.994 (0.475) |
| CC* | 0.999 (0.802) |
| Reflections used in refinement | 46442 (4635) |
| Reflections used for R-free | 2474 (203) |
| R-work | 0.1884 (0.2756) |
| R-free | 0.2448 (0.3338) |
| CC(work) | 0.966 (0.728) |
| CC(free) | 0.945 (0.543) |
| Number of non-hydrogen atoms | 11586 |
| macromolecules | 11331 |
| ligands | 177 |
| solvent | 78 |
| Protein residues | 1437 |
| RMS(bonds) | 0.003 |
| RMS(angles) | 0.60 |
| Ramachandran favored (%) | 97.19 |
| Ramachandran allowed (%) | 2.60 |
| Ramachandran outliers (%) | 0.21 |
| Rotamer outliers (%) | 2.42 |
| Clashscore | 5.14 |
| Average B-factor | 58.39 |
| macromolecules | 58.46 |
| ligands | 59.68 |
| solvent | 44.90 |
| Number of TLS groups | 14 |

Note: Statistics for the highest-resolution shell are shown in parentheses.

**Table S10. Compound information and corresponding identifiers used in this study.**

| Compound# | Name | CAS# | Formula | Supplier | Ref. |
|---|---|---|---|---|---|
| **1** | Mogrol | 88930-15-8 | $C_{30}H_{52}O_4$ | ChemFaces | / |
| **2** | Mogroside IA1 | 88901-46-6 | $C_{36}H_{62}O_9$ | Yuanye | / |
| **3** | Mogrol 25-*O*-glucoside | / | $C_{36}H_{62}O_9$ | / | / |
| **4** | Mogroside IIE | 88901-38-6 | $C_{42}H_{72}O_{14}$ | Yuanye | / |
| **5** | Mogroside IIE 4′-*O*-glucoside | / | $C_{48}H_{82}O_{19}$ | / | / |
| **6** | Mogroside III | 130567-83-8 | $C_{48}H_{82}O_{19}$ | Yuanye | / |
| **7** | iso-Mogroside IVA* | 2146088-08-4 | $C_{54}H_{92}O_{24}$ | / | / |
| **8** | Mogroside IIIA2 | 88901-43-3 | $C_{48}H_{82}O_{19}$ | ChemFaces | / |
| **9** | Mogroside IIIE | 88901-37-5 | $C_{48}H_{82}O_{19}$ | ChemFaces | / |
| **10** | iso-Mogroside IVE* | 2146088-06-2 | $C_{54}H_{92}O_{24}$ | / | / |
| **11** | Siamenoside I | 126105-12-2 | $C_{54}H_{92}O_{24}$ | Yuanye | / |
| **12** | Mogroside IV | 89590-95-4 | $C_{54}H_{92}O_{24}$ | Yuanye | / |
| **13** | Mogroside IVA | 88901-41-1 | $C_{54}H_{92}O_{24}$ | ChemFaces | / |
| **14** | Hederagenin | 465-99-6 | $C_{30}H_{48}O_4$ | ChemFaces | / |
| **15** | Hederagenin 28-*O*-glucoside | 53931-25-2 | $C_{36}H_{58}O_9$ | / | [1] |
| **16** | Oleanolic acid | 508-02-1 | $C_{30}H_{48}O_3$ | ChemFaces | / |
| **17** | Ursolic Acid | 77-52-1 | $C_{30}H_{48}O_3$ | ChemFaces | / |
| **18** | Protopanaxadiol | 30636-90-9 | $C_{30}H_{52}O_3$ | ChemFaces | / |
| **19** | Protopanaxatriol | 34080-08-5 | $C_{30}H_{52}O_4$ | ChemFaces | / |
| **20** | Protopanaxatriol 3-*O*-glucoside | / | $C_{36}H_{62}O_9$ | / | / |
| **21** | Ginsenoside F1 | 53963-43-2 | $C_{36}H_{62}O_9$ | ChemFaces | / |
| **22** | Ginsenoside Rh1 | 63223-86-9 | $C_{36}H_{62}O_9$ | ChemFaces | / |
| **23** | Ginsenoside CK | 39262-14-1 | $C_{36}H_{62}O_8$ | ChemFaces | / |
| **24** | Ginsenoside F2 | 62025-49-4 | $C_{42}H_{72}O_{13}$ | ChemFaces | / |
| **25** | Ginsenoside F1 3-*O*-glucoside | / | $C_{42}H_{72}O_{14}$ | / | / |
| **26** | Ginsenoside Rh1 3-*O*-glucoside* | / | $C_{42}H_{72}O_{14}$ | / | / |
| **27** | Ginsenoside Rg1 | 22427-39-0 | $C_{42}H_{72}O_{14}$ | ChemFaces | / |
| **28** | Ginsenoside Rf | 52286-58-5 | $C_{42}H_{72}O_{14}$ | ChemFaces | / |
| **29** | Ginsenoside Rh2 | 78214-33-2 | $C_{36}H_{62}O_8$ | ChemFaces | / |
| **30** | Ginsenoside Rh1 monoglucoside* | / | $C_{42}H_{72}O_{14}$ | / | / |
| **31** | Quillaic acid | 631-01-6 | $C_{30}H_{46}O_5$ | ChemFaces | / |
| **32** | Quillaic acid monoglucoside* | / | $C_{36}H_{56}O_{10}$ | / | / |
| **33** | Momordicine I | 91590-76-0 | $C_{30}H_{48}O_4$ | ChemFaces | / |
| **34** | Momordicine IV | 894412-35-2 | $C_{36}H_{58}O_9$ | / | / |
| **35** | Momordicine II | 91590-75-9 | $C_{36}H_{58}O_9$ | / | / |
| **36** | Cucurbitacin IIb | 50298-90-3 | $C_{30}H_{48}O_7$ | ChemFaces | / |
| **37** | Cucurbitacin IIb 25-*O*-glucoside | / | $C_{36}H_{58}O_{12}$ | / | / |

| Compound# | Name | CAS# | Formula | Supplier | Ref. |
|---|---|---|---|---|---|
| **38** | Mogroside IE1 | 88901-39-7 | $C_{36}H_{62}O_9$ | Yuanye | / |
| **39** | Mogroside IIA2 | 88901-45-5 | $C_{42}H_{72}O_{14}$ | ChemFaces | / |
| **40** | Mogroside IIA1 | 88901-44-4 | $C_{42}H_{72}O_{14}$ | ChemFaces | / |
| **41** | Cucurbitacin IIa | 58546-34-2 | $C_{32}H_{50}O_8$ | ChemFaces | / |
| **42** | Cucurbitacin B | 6199-67-3 | $C_{32}H_{46}O_8$ | ChemFaces | / |
| **43** | Cycloastragenol | 78574-94-4 | $C_{30}H_{50}O_5$ | ChemFaces | / |
| **44** | Mogroside IIB* | 942615-25-0 | $C_{42}H_{72}O_{14}$ | / | / |
| **45** | Mogroside IIA2 25-*O*-glucoside | / | $C_{48}H_{82}O_{19}$ | / | / |
| **46** | Cycloastragenol 25-*O*-glucoside | 86500-65-4 | $C_{36}H_{60}O_{10}$ | / | [2] |

Note: The "Ref." column denotes the source references of previously published spectroscopic data used for structural identification. An asterisk indicates that NMR data for the product is lacking.

**Table S11. The amino acid sequences of UGT73s.**

| UGT73s | Sequences |
|---|---|
| BmUGT73GF5 | MAVSANLHFVLFPLMAQGHMIPMVDIARLLAQRGVKITIFTTPHNASRFEGHLA RLIGSGLHIKVIKLDFPCEEAGLPVGCENVDLLPSPDYALPFFTAANMLQKPAEV LFQQLSPRPSCIISDMCLPYTMDIACKFDIPRLSFIGFGCFALFILHKLKLFPEILES VSSEHEYFVIPGLPDKIEITKAQIPLQWKESFEEFGERINAAEMASHGVVVNSCEE LEGKYAEEYKREKENKVWCIGPVSLCNKNILDMAERGRPGSIDGQYCLDWLDK HESKSVIYVCLGSICNLTALQMMELGSGLEASNRPFIWVIREGKSSHELKILMEG EKFEEGIEGKGLLIWGWAPQLLILSHPAVGGFLNHCGWNSTTEGIAAGVPMITW PLFADQFLNEKVVEQILKIGVKVVSEKPTNPAFGIHTEMQVKREDVKKAVETLM EEGEDGEGRRRRARELGEMVKMAGEEGGSSHLNLTSLIQEIMNHAGSKEHS* |
| BmUGT73GF6 | MAVSANLHFVLFPLMAQGHMIPMVDIARLLAQRGVKITIFTTPHNASRFEGHLA RLIGSGLHIKVIKLDFPCEEAGLPVGCENVDLLPSSDYALPFFTAANMLQKPAEV LFQQLSPRPSCIISDMCLPYTMDIASKFDIPRLSFIGFGCFALFILHKLKFFQDVVE SVSSEHEYFVIPGLPDKIEMTKAQIPGPWKEGFKEFRERINAAEMASHGVVVNS CEELEGKYAEEYKREKENKVWCIGPVSHCNKNILDMAERGRPGSIDGQYCLDW LDKHESKSVIYVCLGSLCNHNALQMLELGLGLEASNRPFVWVIRDGENSDELK VLMESEKFEERIEGKGLLIWGWAPQLLILSHPAVGGFLTHCGWNSTTEGIAAGV PMITWPLFAEQFLNEKVVEQILKIGVKVGAEKPTNPIFGIHAEMQVKREDVKKA VETLMEEGGDGEGRRRRARELGEMVKMAGEEGGSSHLNLTSLIQEIMNHAGSK EHS* |
| CsUGT73AM3 | MDSHTHGTPHFLLFPFMAQGHMIPMIDLAKFLARRGAIVTIVTTPLNSARFHSVL TRAIDSGHQIHVRELQFPSHQETGLPEGCENVDLLPSLASISQFYRAISLLHQPSE KLFEQLTPRPNCIISDMCIPWTFDISQKFHVPRLVFYSLSCFFLLCMRSLTTNYEF LNSNPDSEFLTLPGLPSQVEFRRSQIFTSTDDYLIQYSFRMWEVDRQSYGVIVNV FEEMEPEHVTEYIKGRESPEKVWCVGPLSLSNDNELDKAERGNKAIIDGHECIK WMDEQKPSSVVYVSLGSLCNLCTEQIKELGLGLVASNKPFIWVIRKANLTEALV KWMDEYEFEEKTKGRGLVIRGWAPQVLILSHSAIGCFLTHCGWNSSVEGISAGV PMITWPLFADQLYNHKFIVEILKVGVSVGEGTVGDLGGVQKVVVKREKVKEAI EMVMDGDGSEERRKRCKEYGEKAKKAAEEGGSSHRNLNRLVEDITAHAFACG NGNGSC* |
| CsUGT73AM7 | MASTPHFLLFPFLAQGHIIPTLDLAKLLARRGAIVTILTTPHNATRNHSVLARAID SGLQIHVVQIPFPCNKAGLPEGCENMDLLPSFRSVPTFFRSTFLLYDSSDELLQQL CPPPTAIISDICLPWTLTLAQKYNIPRLVFYNLSCLYFLCLKDLEMKGPLIQSISDS DTVTLVDGFKFRKAQLPKSVNEDMIAFIEEINKADRMSHGVIFNSFEELEPKNLA EYKKIGELPDRVWCVGPVWLCNDDKLDRAYRGDRASIDENECSKWLDEQGPC SVVYVALGSLCNLVTGQLIELGLGLEASNKPFIWVIRKGNLTEELLKWVEEYDF EGKIKGRGVLIRGWAPQVLILSHPSIGCFLTHCGWNSSMEGITVGVPMITWPLFA DQVFNQTLIVEILRIGVSLGVEEGVPWGEEEEKGIVVRKEKVKEAIEMVMEGEN REELKKRCRELGEKAKMAVEEGGSSHRNLTLLIQDAQKNFEL* |
| CsUGT73AM8 | MASTHILLFPFMAQGHMIPMFDLAKLLAHHGFIITIVTTPHNAHRYHSVLARAT HSGLQIHVALLPFPSTQVGLPEGCENLDSLPPPPSSVSAFCRATYLLYEPSEKLFH QLSPRPSCIISDMCLPWTLRLAQNHQIPRLVFYSLSCFFLLCMRSLKTNHSLVTSI SDSEFLTLPDLPHPVEIRKSRLPTMKNEEMGKLSYDMAEADRVSHGVILNVFEE MEAEYVAEYRKSRDSPQKVWCVGPVSLCNDNKLDKAERGEKSSIHEDECMKW LNGQQPSSVVYVSMGSLCNLSTPQLIELGLGLEASKKPFIWAIRKGNLTDELQS WIMEYNFEGKIEGWGLVIRGWAPQVAILSHSAIGSFLTHCGWNSSIEGISAGVP MITWPLFADQVFNAKLIVEVLKVGVNVGEETALYWGEEKDKEVMVKREEVRE AIEMVMNGENREEMKERAEKLAEMAKRAVEEGGSSHQNLKELVEELFKI* |

| UGT73s | Sequences |
| --- | --- |
| CsUGT73AM9 | MASPPHFLLFPFMAQGHVIPMIDLAKLLAHRGVIITIVVTPTNAARNHSVLDRAI<br>RSGLQIRMIQLPFPSKEGGLPEGCDNLDLLPSFKFASKFFRATSFLYQPSEDLFHQ<br>LKPRPICIISDTYLPWTFQLSQKFQVPRLVYSTFSCFCFLCIHCLMTNPALSISDSD<br>SVIFSDFTDPVEFRKSELPKSTDEDILKFTSEIIQTDAQSYGVIFNTFVEMEYNYIT<br>DYRKTRQKSPEKVWCVGPVSLYNDDKLDLLERGGKASINQQECINWLDEQQPS<br>SVIYVSLGSLCNLVTAQLIELGLGLEASNKPFIWSIREANLTEELMKWLEEYDLE<br>GKTKGKGLVICGWAPQVLILTHSAIGCFLTHCGWNSSIEGISAGVPMITWPLFGD<br>QIFNYKLIVDVLKVGVSVGVETLVNWGEEDEKGVYVKREMVREAIEMVLEGE<br>KREEMRERSKKLAEIAKRGMEEGGSSYKDITMVIEDIIGNGGC* |
| CsUGT73AT1 | MASSKSKQSLLLPHFLLFPVMAQGHQIPMAELAKLLSQSGVKTTLITTPQNATRI<br>QSLLSQSPLTQIIQLPFPSHQQHLLQNCENFDSLPSLHLLPQFLTATSFLYSEIEHL<br>FPQLSPKPCCIVSDMALPWTIQIAHKFNVPRLVFYSLSAFYLLFMATLRATDFGE<br>KIMAASDYELISIPNFPDSIQVTKSQLVFTLDPVFLEWGNQMAKADRASYGFIM<br>NSFNGLEPKYLEEFKKTIGSDKVWCIGPVSLCNKDTKDKAKRGNKAAIDEQEC<br>MKWLDKQESESVIYAALGSICNVIAPQIIELGLALEASNKPFIWVIRQTKSTKKE<br>VENWLAESEFEQRIKDRGLVIRGWAPQVLILSHPAVGGFVTHCGWNSTIEGISM<br>GVPMVTWPLFSDQTFNEKLIVEVLRIGVSVGVEKCLRWGVEEEIGVQVKKEAIR<br>GAIEKVMSGEGEEMRKRVRELAAIAKATMEEGGSSHLNLKRLIEEIMHQADSQ<br>QSQN* |
| GpUGT73AM10 | MASQCLEPHFVLFPLMAQGHMIPMVDLAKLLAQRGAIVTLVTTPHNAARYDG<br>DLSRATDSGLRIRVAQLQFPCEEAGLPKGFENLDLFPPTSDAMIRFFKATYLLCD<br>ASEKLLEDLSPRPSCIVSDMCLPWTMQVAKKHHIPRIAFSTFSFFFFSCMRSLVIN<br>RDHINSLSDSEYVTFSDLPDPIELQKSQIPQTLDEEMLKFSHEMREVDRLSYGVII<br>NTFEEMEPKYAEEYKKVRESDKVWCIGPVSLCNKEKLDKAQRGNNASIDEYEC<br>INWLDKQQPCSVIHVSLGSLCNLLTPQLIELGLGLEASNKPFIWVIRQGNMTREL<br>EKWLLEYGFEERTKGRGLVIRGWAPQVLILSHRSIGGFLTHCGWNSTIEGISAGV<br>PMITWPLFADQIFNEKLITQILRIGISVGVDTIMAWGEEEKKGVLVKKENVKEAI<br>DSLMDGDDRNERRERCKKLAEAANRAVEEGGSSHHNLTLLIEDVIHHQAGCVN<br>GTC* |
| GpUGT73AM11 | MASQNHKPHFVLFPFMAQGHLIPMVDLAKLLAQQGAIVTIVNTPHNAARYDAV<br>LARAVVSGLQIRVALLEFPCEEAGLPKGSENMDLLPTLDSMLKLFKATFLLCDA<br>SDKLIEDLSPRPSCIVSDMCLPWTMQVDKKHHIPRIVFYSFSFFFLLCMRSLVVN<br>SDHIKSIPDSEYVTLSDVPDQVKFRKSQLPQTPNEEMEKLSYEMGEADRLSYGVI<br>INTFEEMEPRYVEEYKKVRESEKVWSIGPVSLCNKENLDKAQRGNKASIDEHEC<br>INWLDKQQPCSVIHVSFGSLCNLLTPQLIELGLGLEASNKPFIWVIRQGNLKEELE<br>SWLLEYGFEEKTKGRGLVIRGWAPQVLILSHPSIGGFLTHCGWNSTIEGITAGVP<br>MITWPLFADQIFNEKLITQILRIGISVGVDTVLPFGEEEKRGVLVKKENVKEAIDS<br>LMDGDDREERRERCKKLAEVANRAVEEGGSSHHNLTLLIEDFIHQTSCDNGTF* |
| GpUGT73AM12 | MASQHYKPHFVLFPYMAPGHMIPMIDLAKLLAQQGAIVTIVNTPHNAARFDDV<br>LARAVDSGLQIQVALLQFPCEEAGLPKGSENVDLLPSLDSQIKLFKATILLSDAS<br>EKLLEDLSPKPSCIVSDFCLRWTMQVAKKHHIPRIVFYCLSFFSLLSLRSLVINRD<br>HIKSLSDSEYVTLSDMPDQVKFQKSQLRQPVDEEMGKLAYDMGEADRLSYGVI<br>VNTFEEMEPRYVEEYKKVRESDKVWCIGPVSLCNKEKLDKAQRGNKASIDEHE<br>CINWLDKQQPCSVIHVSLGSLCNLQTPQLIELGLGLEASNKPFIWVIRQGNLKEE<br>LESWLLEYGFEEKTKGRGLVIRGWAPQLLILPHPSIGGFLTHCGWNSTLEGITAC<br>VPMITWPLFADQIFNEKLITQILRIGISIGVDTFVAWGGEEEKGDFVKKENVKEAI<br>DSLMDGDDREERRGRCKKLAEVANRAVEEGGSSLQNLTLLIEDFTHQASCD* |

| UGT73s | Sequences |
|---|---|
| GpUGT73AM13 | MASQHYKPHFVLFPYMAPGHMIPMIDLAKLLAQQGAIVTIVNTPHNAARFDDV<br>LARAVDSGLQIQVALLQFPCEEAGLPKGSENLDLLPSLDSHIKLFKATILLSDASE<br>KLLEDLSPKPSCIVSDFCLQWTMQVAKKHHIPRIVFYSLSFFYLLSIRSLVINCDHI<br>KSLSDSEYVTLSDMPDQVEFQKSQLPQPVDEEIGKLTYDMGEADRLSYGVIVNT<br>FEEMEPRYVEEYKKVRESDKVWCIGPVSLCNKEKLDKAQRGNKASIDEHECIN<br>WLDKQQPCSVIHVSLGSICNLQTPQLIELGLGLEASNKPFIWVIRQGNLKEDLES<br>WLLEYGFEEKTKGRGLVIRGWAPQVLILPHPSIGGFLTHCGWNSTIEGISAGVPM<br>ITWPLFADQIFNEKLITQILRIGISIGVDTFVAWGGEEEKGDFVKKENVKEAIDSL<br>MDGDDREERRGRCKKLAEVANRAVEEGGSSLQNLTLLIEDFTHQASCD* |
| GpUGT73AM14 | MASLHHKPHFVLFPFMAPGHIIPMVDLSKLLAQRGAIVTIVDTPNNAARYDAVL<br>ARAIDSGLQIRVALLQFPCGEAGLPKGSENLDLLPSLDSMQKLFKATVLLCDAS<br>EKLLEDLRPRPSCIVSDMCLPWTIQVAKKHHIPRIGFYSLSFFFLMCMQSLAKNR<br>DHIKSIPDSEYVTLSDVPDQVEFRKSQLPQTPYEEMEKLSYEMGEADRLSYGVIV<br>NTFEEMEPRYVEEYKKVRESDKVWSIGPVSLCNKENLDKAQRGNKASIDEHECI<br>NWLDKQQPCSVIHVSFGSLCNLLTPQLIELGLGLEASNKPFIWVIRQGNLKEELE<br>SWLLEYGFEEKTKGRGLVIRGWAPQVLILSHPSIGGFLTHCGWNSTIEGITAGVP<br>MITWPLFADQIFNEKLITQILRIGISVGVDTVLPLGEEEKRGVLVKKENVKEAIDS<br>LMDGDDRKERRERCKKLAEVANRVVEEGGSSHHNLTLLIEDFSHQTSCDNGTC<br>* |
| GpUGT73FX1 | MASISHSPKPHFILFPLMAQGHLIPMLDLAKLLAHQGVIITFVTTPHNAARFEQV<br>LKRAIDSGLHIQIVKLPFRCEEVGLPKGCENFDLLPSLGLAPNFVQATLSMRQAT<br>DKLFEELSPRPTCLISDMGFPWTVEISLKFHIPRFVFCSMSCFYLLSLLNLRENRA<br>LLDSIASESEYFLIPNLPDRIRITKSQINYVPNVLFSKVRNEVAKADLASYGVIVN<br>TFEDLDTEYVEEYKRAVGPERVWCVGPVSLCNKDILDKFQRGNNASIDGFECLE<br>WLDKQQPCSVVYAALGSFCNVILPQFVELGLALEESNKPFIWVIRQGKVLRELE<br>KWISEYGFEEKTKGRGLVIHGWAPQILILSHPAIGGFITHCGWNSTIEAISAGVPLI<br>TWPLFGDQFFNEKLIVQVLKIGIRIGVEKCVMWGDEEKIGVMVKKENIRDALHS<br>LMNGEERDEIRERTKKLSEKAKRAVEEGGSSHLNMKQLIADIVHIGAGWSRPNF<br>* |
| McUGT73AM15 | MAMASPHFVLFPFMAQGHMIPMIDLAKLLARRGAIISFITTPRNAARNYSVLAR<br>AINSGLRIHVHQLDFPSNQVGLPEGCENLDLLPSYDDHLTSKFFQATFLLREPWE<br>KLVEQLIPRPTCIITDTGFPWTLPLSLRLHIPRLVYYTHSCLYLLIMRSIQSRQPLL<br>ASISDDSEYVTLSDLPHAVQFLKSQLSSVADGDAAKFLEAMEEADAHSHGVVIN<br>IFEEMEAENVAAYRKHRKSPDRVWCVGPVSLCSEDKLDMAQRGNRACIDEAQ<br>CMNWLDAQRPSSVVYVSLGSLCNLSTAQLIEMGSGLAESKKPFIWVVRKEYAT<br>EEFQKWATEYDLEGKTEGRGLVIQGWAPQVLILTHSAIGCFLTHCGWNSCTEAI<br>SAGVPMITWPIFGDQNFNKKLIVEILRIGVGVGVETAVDWAGKAEKRALVKRD<br>KVREAIEMAMDGDGSEEMRERCKQLAEKAKRALQEGGSSHLNLKLLIQDIVAY<br>GTGCQNGNCS* |
| McUGT73AM16 | MATAMASPHFLIFPFMAQGHMIPMMDLANLLAHQGAIVTIVTTPHNAARYHSV<br>LTRAIGSGSQIRVVQLEFPCHEAGLPHGCENLDLLPSLSSMSTFMKATYLLHDPS<br>EKLLPQLSPRPTCIISDMCLPWTLTLAHKFRVPRLVLYSLSCFFLLCIHSVKYHIPS<br>FSSISDSELVDFSGLPHPVQFRKSQLPKATDEAMSKFGYEMGEADRQSHGVIINT<br>FEEMEPEYLAEYRKLRELPEKVWCVGPLLLYNDNKLDVAQRGNTAAIDENECI<br>NWLDGQRPCSVVYVSLGSLCNLTTPQLIELGLGLEASNKPFIWVIRKLNLTKELL<br>DWMEEYDFEGRTKGRGLVIRGWAPQVMILSHSSTGCFLTHCGWNSSIEGMSAG<br>VPMITWPLFADQVFNEKLLVEIVRIGVSVGAETAVPWGEEEKIGVLVKRESVRE<br>AIEMAMDGDGSEEMRQRCKQLAEKAKRAVQEGGSSHRNLKLLIEEIVDDGRSC<br>ENGSC* |

| UGT73s | Sequences |
| --- | --- |
| McUGT73AM17 | MASPHFLLFPFMAQGHMIPMIDLAKLLAHQGAIVTIVTTPHNAARCHSVLTRAI<br>HSGLQIRVLQLEFPCHEVGLPHGCENLDLLPSLSSSSAFHKATCLLHDQSEKLFP<br>QLSPRPTCIVSDMCLPWTLTLAHKFRLPRLVLYSLSCFSLLCMHTLITQLPLLSSF<br>SDSELVTFSDLPHPVQFRKSQLPIATDEEMAKLGYDMMEANRQSHGVIINTFEE<br>MEHEHLAEYRKLREFPERVWCVGPVSLCNNDKLDVAQRGNTAAIDVHECVNW<br>LDGQRPCSVVYVSLGSLCNLTTPQLIELGLGLEASNKPFIWVIRKLNLTKEMLD<br>WMEEYDFEGRTKRRGLVIRGWAPQVMILSHSSTGCFLTHCGWNSSIEGMSAGV<br>PMITWPLFADQVFNERLLVEIIRIGVSVGAETAVPWGEEEKIGVLVKRESVREAI<br>EMAMDGDGSEEMRQRCKQLAEKAKRTVKEGGSSHRNLNLLIQDTVAQGRSCD<br>NGNC* |
| McUGT73AM18 | MASPHFVLFPYMAQGHMIPMIDLAKLLAQRGAIVTLVTTPKNAARNDSVLARA<br>IESGLQIKVVQLEFPWNQAGLPEGCENLDLLPSLASISKFNAAASLLYEPSVQLL<br>GNLSPRPACVVSDMNLPWTLRLARHHRLPRLVFYTFSCFFLLSLRSFAKTHTLLS<br>STSDEELVKFGVEMMVADQHSDGVIFSGFEEMEPEQIAEYRNERKSPEKVWCV<br>GPVSLCNDDKLDVAQRGNRASIDVHECVNWMDGQHQSSVVYISLGSLCNVDA<br>SQLIELGLGLEASNKPFIWVIRELNLTEELLNWLEEYDLVGRTKGRGLVIRGWA<br>PQMMILSHSAIGCFLTHCGWNSSMEGVSAGVPMITWPLFADQMSNGKLIVEILK<br>IGVSVGVDTSVPWGEEEKRGVLVRREKVREAIEMAMDGEGSEEMRQRCKQLA<br>EKAKRAVQEGGSSNRNLTLLMDHINGGSS* |
| McUGT73AT6 | MAPHFLLVPIMAQGHLIPMADFAKLLAEQGVKVTLVTTPQNVPRFQSLFSKPAL<br>QIQIQIINFPSQNGIEYENFDSLPSLGLSAKLLAETSLLRGPTEELFERLSPRPSCLV<br>SDMALPWTGHVARKFNVPRLVFYSLSCFYLRVMITLRTSGVVRKILAAPESEPI<br>ALPNLPDPVQVTRSKSIFSLDPLFVDWTGQLAKADHESHGFALNSFHDLEPKYL<br>QEFKNAIGSDKVWYIGPVNLCNKDIMDKVQRGNKSAIGERECLEWLDKQDPGS<br>VIYAALGSQCNVVTTQIVELGLALQASNRPFIWVIRQTNATHELEKWLSEHNFE<br>GKTNGRGLVVRGWAPQVLILSHRATGAFLTHCGWNSTVEAISAGIPMVAWPLF<br>ADQVFNEMLIVQILKIGASVGVEKCYTWGVEEEIGVQVKKEVVIDALEKVMDG<br>EDKKGMRRRAKEFAERAKAAMEDGGSSQLNIKLLIEDIMHQQHFVPA* |
| McUGT73AT7 | MASENAAAAAPHFLLLPIMAQGHLIPMADFAKLLAEQGVKVTLVTTPQNAPRF<br>QSLLSKPALQIQIQTISFPPENGIQCENFDSLPSLELGAKFFADTSLLRGPTEELFQ<br>RLSPRPSCLVSDMALPWTGGIARKFNVPRLVFYSLSCFYLRVITTLRTTTDVVYK<br>IMAAADLEPVTIPDLPDPVQITKAKLIFSLDPIFMDWSAQMAKADHESYGFVLNS<br>FQDLEPKYLSEFKKVTGSKKVWCVGPVSLCNKDTMDKTHRGDKSAIGERECLE<br>WLDKQDPSSVIYAALGSTCNVATSQMIELGLALQASNRPFIWVIRQTNATQGLE<br>KWLSEHNFEDKANGRGLVVRGWAPQVLILSHPAIGAFVTHCGWNSTIEAIATG<br>VPMVAWPLFADQMFNEMLIVQILKIGASVGVEKCYAWGAEEEIGVQVKKEAVI<br>DAIEKVMDGEDKEGMRRRVREFAERAKAATEDGGSSHLNVKLLIKDIMDQGH<br>SGPNKI* |
| SgUGT73AM1 | MLMHALTPTAPFFSIKPNTEPPSATTRQPPMDSPPQKPHFLLFPFMAQGHMIPMI<br>DLAKLLAQRGAIITVVTTPHNAARYHSVLARAIDSGLHIHVLQLQFPCNEGGLPE<br>GCENFDLLPSLGSASTFFRATFLLYEPSEKVFEELIPRPTCIISDMCLPWTVRLAQ<br>KYHVPRLVFYSLSCFFLLCMRSLKNNQALISSKSDSELVTFSDLPDPVEFLKSQL<br>PKSNDEEMAKFGYEIGEADRQSHGVIVNVFEEMEPKYLAEYRKERESPEKVWC<br>VGPVSLCNDNKLDKAQRGNKASIDERECIEWLDGQQPSSVVYVSLGSLCNLVT<br>AQLIELGLGLEASNKPFIWVIRKGNITEELQKWLVEYDFEEKTKGRGLVILGWA<br>PQVLILSHPAIGCFLTHCGWNSSIEGISAGMPMITWPLFADQVFNEKLIVEILRIG<br>VSVGMETAMHWGEEEEKGVVVKREKVREAIERAMDGDEREERRERCKELAE<br>MAKRAVEEGGSSHRNLTLLTEDILVNGGGQERMDDADDFPTIVN* |

| UGT73s | Sequences |
|---|---|
| SgUGT73AM2 | MDSPPHRPHFLLFPFMAQGHMIPMIDLAKLLAQRGAIVTILTTPHNAARTHSVL<br>ARAIDSGLQIRVRPLQFPCKEAGLPEGCENLDLLPSLGSASTFFRATCLLYDPSEK<br>LFEELSPRPTCIISDMCLPWTIRLAQKYHVPRLVFYSLSCFFLLCMRSLKNNPALI<br>SSKSDSEFVTFSDLPDPVEFLKSELPKSTDEDLVKFSYEMGEADRKSYGVILNIFE<br>EMEPKYLAEYGNERESPEKVWCVGPVSLCNDNKLDKAQRGNKASIDERECIK<br>WLGGQQPSSVVYASLGSLCNLVTAQFIELGLGLEASNKPFIWVIRKGNITEELQK<br>WLVEYDFEEKTKGRGLVILGWAPQVLILSHPSIGCFLTHCGWNSSIEGISAGVPM<br>VTWPLFSDQVFNEKLIVQILRIGVSVGAETAMNWGEEEEKGVVVKREKVREAIE<br>RMMDGDEREERRERCKELAETAKRAIEEGGSSHRNLTLLIEDIGTSLRRL* |
| SgUGT73AM19 | MASPRHTPHFLLFPFMAQGHMIPMIDLARLLAQRGVIITIITTPHNAARYHSVLA<br>RAIDSGLHIHVLQLQFPCKEGGLPEGCENVDLLPSLASIPRFYRAASDLLYEPSEK<br>LFEELIPRPTCIISDMCLPWTMRIALKYHVPRLVFYSLSCFFLLCMRSLKNNLALI<br>SSKSDSEFVTFSDLPDPVEFLKSELPKSTDEDLVKFSYEMGEADRQSYGVILNLF<br>EEMEPKYLAEYEKERESPERVWCVGPVSLCNDNKLDKAERGNKASIDEYKCIR<br>WLDGQQPSSVVYVSLGSLCNLVTAQIIELGLGLEASKKPFIWVIRRGNITEELQK<br>WLVEYDFEEKIKGRGLVILGWAPQVLILSHPAIGCFLTHCGWNSSIEGISAGVPM<br>VTWPLFADQVFNEKLIVQILRIGVSVGTETTMNWGEEEEKGVVVKREKVREAIE<br>IVMDGDEREERRERCKELAETAKRAIEEGGSSHRNLTMLIEDIIHGGGLSYEKGS<br>CR* |
| SgUGT73AM20 | MDSPRHKPHFLLFPFMAQGHMIPMIDLAKLLAQRGAIITILTTPRNAARYHSVLA<br>RAIDSGLHIHVLQLQFPCNEGGLPEGCENFDLLPSLGSAPTFFRATFLLYDPSEKL<br>FEELIPRPTCIISDMCLPWTIRLAQKYHVPRLVFYSLSCFFLLCLGSLGSKNALVS<br>FMSDSEFVTLPDLPDQFEFRKALLPKSTDEDMAMFNYELMEADRMSHGVILNT<br>FEEMEPKYLAEYKKIRESPEKVWCVGPVSLCNDNKLDKAQRGDKASIDEHECI<br>KWLDGQQPSSVVYVSMGSLCKLVTAQLIELGLGLEASKKPFIWVIRKGNITEEL<br>QKWVVEYDFEEKTRGRGLLILGWAPQVLILPHPAIGCFLTHCGWNSCIEGISAG<br>VPMVTWPLFADQVFSEKLIVQILRLDVSVGVETAMHWGEEEEKEVVVRREKVR<br>EAIEIVMDGDEREERRERCKELAETTKRAAEEGGLVSSKPHTAD* |
| SgUGT73AM21 | MDSSPQKPHFLLFPFMAQGHMIPMIDLAKLLAQRGAIITIVTTPDNASRTHSVLA<br>RAIDSGLQIRVRPLQFPCKEAGLPEGCENLDLLPSLGSASTFFRATCLLYDPSEKL<br>FEELIPRPTCIISDMCLPWTIRLAQKYHVPRLVFYSLSCFFLLCMRSLKNNQALIS<br>SKSDSELVTFSDLPDPVEFLKSQLPKSIDEEMAKFGYEMGEADRQSHGVIVNAF<br>EEMEPKYLAEYRKERESPEKVWCVGPVSFCNDNKLDKAQRGNKASIDERECIE<br>WLDGQQPSSVVYVSLGSLCNLVTAQLIELGLGLEASNKLFIWVIRKGNITEELQ<br>KWLAEYDFEEKTKGRGLVIRGWAPQLLILSHPAIGCFLTHCGWNSSIEGISAGVP<br>MITWPLFTDQVFNEKLIVEILRIGVSVGMETAMHWGEEEEKGVVVKREKVREAI<br>ERLMDGDARDERRERCKNFGEMAKRAVKEEGSSHRNLTLLIEDIVRGGGCDC* |
| SgUGT73AM22 | MDSPPQKPHFLLFPFMAQGHMIPMIDLAKLLAQRGAIITVSTTPHNAARYHSVL<br>ARAIDSGLHIHVLQLRFPCKEGGLPEGCENIDLLPSHYSPFTFFSATFLLYEPSEK<br>VFEELIPRPTCIISDISLPWTIRLAHKYHVPRLVFYSLSCFFLLCMRSLRNNQALIS<br>SKSDSELVTFSDLPDPVEFLKSQLPKSNDEEMAKFGYEIGEADRQSHGVIVNVFE<br>EMEPKHLAEYRKERESPEKVWCVGPVSLCYDNKLDKAQRGNKASIDERECIEW<br>LDGQQPSSVVYVSLGSQCNLVTAQLIELGLGLEASKKPFIWVIRKAIITEELQKW<br>LVEYDFEEKTKGRSLLIRGWAPQVLILSHPALGCFLTHCGWNSSIEGISAGVPMV<br>TLPLFGDQIFNETLIVQILRIGVRVGVEAAMQCGEEEEIDRGVVVRREKVREAIGI<br>VMDGDEREGRRERCKELAETAKRAVEEGGSSHQNLTRLIEDIASSLRRLSEWKF<br>YLIIIS* |

| UGT73s | Sequences |
| --- | --- |
| SgUGT73AM30 | MASLSPPQKPHFLLFPFMAQGHMIPMIDLAKLLAQRGAIITVATTPHNAARTHS<br>VLARAIDSGLQIRVRPLQFPCKEAGLPEGCENLDLLPSLGSASTFFRATCLLYDPS<br>EKLFEELIPRPTYIISDMCLPWTIRLAQKYHVPRLVFYSLSCFFLLCMRSLKNNQA<br>LISSKSDSELVTFSDLPDPVEFLKSQLPKSIDEEMAKFGYEMGEADRQSHGVIVN<br>AFEEMEPKYLAEYRKERESPEKVWCVGPVSLCNDNKLDKAQRGNKASIDEREC<br>IKWLDGQQPSSVAYVSLGSLCNLVAAQLIELGLGLEASNKPFIWVIRKGNITEEL<br>QKWLVEYDFEEKTKGRGLVIRGWAPQVLILSHPAIGCFLTHCGWNSSIEGISAG<br>VPMITWPLFADQVFNEKLIVEILRIGVSVGMETAMHWGEEEEKGVVVKREKVR<br>EAIERVMDGDEREERRERCKELAEKAKRAVEEGGSSHRNLTLLTEDIIVNGGGC<br>ENGRCH* |
| SgUGT73AT4 | MASESKPPHFLLFPVMAQGHLIPMVDLAKLLAQRGVKITLITTPQNASRIDSFLA<br>RATQSGLQIQTLQLQFPSERAGLPEGCENFDSLPSLDLIPKFLTLTTLLCRATEEL<br>FEQLSPRPSCIVSDMALPWTIQLADKFKVPRLVLYSLSCLYLLVMANLQAFNGE<br>KMLPTSESEKFVVPNLPDRVEVTKSQLVFTLDPDFLQWGAEMVKADHASYGFI<br>FNTFDGLEPKYLEGYKKAIGSDKVWCVGPVSLCHKDTADMAVRGNKAAIDQH<br>ECLQWLDRQETGSVVYAALGSICNPIAAQIMELGLALEASKRPFIWVIRQAKAS<br>RELEKWLSEYGFEEKTKGRGVVIHGWAPQVLILSHPAIGAFVTHCGWNSTIEGIS<br>AGVPMVAWPLFADQILNEKVIVQVLRVGVSVGVEKCFRWGAEEEVGVQVKKE<br>AIGDAIEKLMGGEGKEEMRRRVRDLAEKAKAAMEEGGSSHLNVKMLIDDIMH<br>HSSSRF* |
| SgUGT73GB1 | MGAAQPHFVLMPLMSPGHLIPMIDMAKLLAQHGVAVSIVTSPLNALRFKSTVR<br>RSAESGLCINLLTIPFPWQEAGVPEGCENVDSLPCRSLIRNFYLASTLMQRPFEQL<br>LEALHPPPSCLVSGKNLAWTVETARKFRIPRIFFDGMGCFSFACSHKIEASKVHK<br>AIAKSSDSFLVPDIPHRIELTQSKLPENLDGASDMKDVLDSYKAGDVVADGILV<br>NTFEELEGEYIGEFKKVKGDKVWCVGPVSACNTLHSDKFERGKTASINETECVG<br>WLDSWDRQSVVYACLGSICGLKPWQLMELGLGLEASNRPFIWVIGGGEKTQDL<br>QKWVDEGGFTERTKERGLLVWGWAPQVSILSHPAIGAFLTHCGWNSVLEGVT<br>AGVPMITCPLFAEQFLNEQLIERVLGIGVSLGVETCVKWGMEEKAGTVIKREDV<br>KKAIEKIMDQTEEAENRGKKARELGEMAKKSIVKGGSSYRGIDKFIEYVVETKQ<br>RRSKLVHQPPPPPPTTTTTGSNILRT* |
| TpUGT73AM28 | MAMASPRHQPHFLLFPFMAQGHMIPMIDLAKLLAQRGVLCTIVTTPQNAARFD<br>SVLARATHSGLQIRVSQLQFPWEKAGLPQGCENLDLLPSLGYGTTFFQATYLLY<br>DQSEKLFEELSPRPTCIVSDMCLPWTIRLSQKYNVPRLVLYSLSCFFLLCLRSMK<br>TNRDLISSMSDSEFVTFSDLPDRVEFQKSELPKATDEGMAKFAYDMVDADRDSF<br>GVILNIFEEMEPKYLAEYRKERKSPEKVWCVGPVSLCNEQRLDKAQRGNKASID<br>ENECTKWLDGQQPSSVVYVSLGSLCNLLPAQLIQLGLGLEASKKPFIWVIRKGN<br>LTEELQKWLVEYDFEEKTKGRGLVIRGWAPQVLILSHPASGCFLTHCGWNSSV<br>EGLSAGVPMITWPLFADQVFNEKLIVQILRIGITVSAETSLQWGEEENRGVVVKS<br>EEVREGIEMVMDGEESEERGGRCKQLAEMAKRAVEEGGSSHRNLTLLIEDIVN<br>GSGCENGSSC* |
| TpUGT73AM29 | MPSPRSKPHFLLFPFMAQGHMIPMIDLAKLLDQRGAICTIVTTPLNAARFDSALA<br>RAIHSGLQIHVSQLQFPWEEAGLPDGCENLDLLPSMDYNISFFRATNLLYNPSEK<br>LFGELSPRPTCIISDMCLPWTLRLSQKYHIPRLVFYSLSFFFLLCMRSMKTNRDLI<br>NSMSDSEFVTFSDLPDQVEFRKSELPKFTDEEMVKFVADMREADMESLGVILNI<br>FEEMEPKYLAQYRKERKFPDKVWCVGPVSICNENRLDKAQRGNKAFIDEHECT<br>EWLDGQQPSSVVYVSLGSLCNLLPAQLIELGLGLEASKKPFIWVIRKGNLTEELQ<br>KWLVEYGFEEKTKGRGLVIRGWAPQVLILSHPAIGCFLTHCGWNSSIEGLSAGV<br>PMITWPLFADQVFNEKLIVQILRVGISVSVETEEKEGVVEKREKVREAIEMVMD<br>GDEREERRERCKQFAEMAKRAVEEGGSSHRNLTLLIEDIVNGSGFENGSC* |

| UGT73s | Sequences |
|---|---|
| TpUGT73AT5 | MASQPEAPHFLLFPVMAQGHMIPMVDLAKLLAQSGVKVTLVTTPQNASRFHTV<br>LARATQSGLQIQTLQLQFPAQQAGLPEGCENFDSLPSLGMIPKFLTVTSLLGRAA<br>EELFEDLIPRPSCIVSDMALPWTIQIAHKFNVPRIVFYSLSYLYLLVMANLRALD<br>VGTMLASSGSEPIIMPNLPDRVEVTKSQLIFTLDPDFLEWGAEMAKADHASYGFI<br>VNSYDELEPKYLKAYKKAIGSDKVWCVGPVCLCNKETVDMAERGNKAAIDEY<br>ECTEWLDRQEPGSVVYAALGSICNAVAAQIIELGLGLEASNRPFIWVIRQANASE<br>EIENWISEYGFEEKTKGRGVVIRGWSPQVLILSHPAIGGFITHCGWNSTIEGISAG<br>VPMVAWPLFADQIFNEKLIVQVLKVGVSVGVEKSFIWGGEEEIGVQVKKEAIRD<br>AVERLMEGEGSEEMRRRVKDLAKRAKTAMEEGGSSHLNIKLLIEDVMHLTDSG<br>KAPSSSGFTFAPV* |
| TpUGT73GB2 | MDAARPHFVLFPLMSPGHLIPMIDMAKLLAQHGVAVSIVTSPLNALRFESTVYR<br>AAESGLCIDLLTIPFPWLEAGVPEGCENIDSLPSRSLIRNFFLASTLMQRPFEQLLE<br>VLHPPPSCIVSGKNLAWTAETASKFQIPRFFFDGMGCFASACSHKIEVSGVHKTV<br>ANSSDTFLVPGLPHKIELTKSKLPENLDGASDMKDVLNSSLVGDFGADGILVNT<br>FEELESEYIREYKKDKGDKVWCVGPVSACNTLHADKFERGKTASINETECLGW<br>LDPWERQSVVYACLGSICGLKSWQLIELGLGLEASNKPFIWVIGGGEKTEDLRK<br>WVDENGFTERTRERGLLVWGWAPQVSILSHPAVGAFLTHCGWNSALEGITAG<br>VPTLTCPLFSEQFLNEQLIEQVLGIGVRLGVESCVKWGMEEKAGTIIKREKVKN<br>AIEKILDHTDEAGTRRKKAREVGEMARRAIAKGGSSYDDMDKFIAYVVKRNRH<br>RL* |
| VvUGT73CK9 | MASQLLQLHFVLIPFMAPGHLIPMVDMARLLAQHGVIVTVVTTPLNATRFKSMI<br>DRAVESGLQIHLLELQFPAVEAGLPEGCENVDLLPSRSLIRNFFVAASMLQQPLE<br>QLFQELQPRPSCIISGKNLAWTADTARKFQIPRLYFDAMSCFAFSCSHNLEASKV<br>HESISKLETFLVPGLPDQIELTKAQLPESLNPDSSDLTGILNQMRASESIADGIVV<br>NTYEELEPRYVKEYKRIKGDKVWCIGPVSACNKLNLDKAERGKKALVDENQC<br>LRWLDSWEPNSVVYACLGSISGLTALQLIELGLGLEASNRPFIWVIRGGEKSKEL<br>ERWILEEGFEERTEGRGLLIRGWAPQMLILSHPSIGVFLTHCGWNSTLEGVCTGV<br>PILTCPLFAEQFINEKLVVQILGIGVSVGVESAVTWGMEEKFGVVMKREDVMK<br>AIDEVMDKGEGGEKRRKRARELGEMAKKAIEEGGSSYLNMKRLIHYILQQTIG<br>NPSTQLS* |
| PsUGT73FA6 | MALQEHNQLHFVLFPPMAQGHLIPTIDMARLFAERGITVTIITTPLNTFRFKTIID<br>RAVDSGFSIRLLQLRFPSVEAGLPEGCENVDSLPSREMVSNFFEATSVLQEPLEQ<br>LLGVLKPVPSCIISAIAFPWTAETARKLQIPRLVFHGTCCFSLLCCHNILEYKVAD<br>SITSESESFVIPGLPDRIEIIKAMLPGNLPQSSGDLKNFRDQINAAELTAHGVVVNS<br>FNELEPMYAEEYLKAKFGKVWCTGPVSLCNKETLDKAERGNKASVDESQCLK<br>WLDLKDPGSVLYVCLGSLCRLTTSEMIELGLGLEASKHPFIWVIRGRDKYLDLE<br>KWLLEEKFEERNKDRGLVIKGWAPQILILSHKAIGGFLTHCGWNSVLEGVCAG<br>VPMITWPMGEEQFFNEKLVVQVLGIGVKIRGDDIHASDATKPMELMEEHRKFG<br>SDENKREEVEKAVNRVMDGGGEGEERRRKVRELGERAKKAMEGGGSSQLNM<br>TLFIQDIQQQGPKQG* |
| BvUGT73BG6 | MASTDQIHVIVIPLMSQSHLIPMIDLSKILAKRGLIITIITTPLNAARYQATVDRAIR<br>SGRLRIKLVPLPFPCNEAGLPEGCENLDSLPSPDLQTKFLVSSSLLQAPLESHLEK<br>VDPRPSCIISSLPWTKDVAYKFNIPRFAYQGISCFTLLCSHKMMLHKVHENVKY<br>DNEPFLVPDVPDKIEFTYAQLPKMMRTSSSHGDDGIDLKDIRHQFKQAEISAEGL<br>LVNTFEELELDYVKSYQKVAMGNVLCVGALSLCNRMVKDYEEMTSIDEHYCL<br>KWLDTMKPKSVIYVCFGSLCHLLPKQLIEIGKGLEASNSPFIWIIREVDYNEEIKK<br>WVKNENFEDRLKGKGLIIKGWAPQVLILSHPATGGFMTHCGWNSTLEGVSAGV<br>PMITWPMFAEQFYNEKLIVQVLKIGIRIGVEVTMQWGEEANDGIYVKRESVKD<br>VVDQLMDEGEKGQERRRKAQELGERANQAVEEGGSSYMNITSFIEHVKQWCN<br>LN* |

| UGT73s | Sequences |
|---|---|
| JrUGT73AB29 | MASMITSQPHFVLIPLMAQGHMIPMIDMARLFAEHGVIVSLVTTPYNASRFETTI<br>HRATGSGLPILLVQLEFPCKQVGLPAGYENLDILPSRDLLTKFYEGLSMLQQPLE<br>KHLQNQRHPPTCIISDKCLSWTSNTAQKFNIPRLVFHGMGCFSLLSSHNIKFYNA<br>HRSISSDSEPFVIPGLPQRILITRAQLPGSFVTLPGLDDVRDKMQEAESTAYGVVV<br>NTFNELEQGIVEEYGKAIKKKVWCVGPVSLYNKESLDKFERGDKTLIDEQQCLE<br>WLNSMEPSSVIYTCLGSLCRLVPSQIIELGLGLEASKQPFIWVIKTGERYLELEEW<br>LENERFEERNKGRGLLIKGWAPQDFILSHPAIGGFITHCGWNSTIESVCHGVPMI<br>TWPLFSEQFFNEKLVVEILRIGIRVGVEIPVRWGEEEKVGVLVKKEKVEKAIALL<br>MDGGDEGVRRRKKARELGEMARRAMEKGGSSQLNMSSLIEDITKLQSIRSRQG<br>GVVTSNCI* |
| PpUGT73BT3 | MASESHDDQLHFVLIPLMSPGHLIPMADNAKLLAERGVVVTIVTTPLNAIRIKPII<br>DRSIDSGLPIQLVQFSLPLQEFGLPEGCENMDSVPSRKLFWNFFAAVEKLQEPVE<br>KFLETMKPNPSCIIADKYMAWTANIARKFRIPRLLFDGTSCFALLCSHNIQEHKV<br>LESVSGSEPFLVPGLPDEIELTKFQLPGNMNPGSKDFSSLHDKVKESEEGAYGIV<br>VNSFEELEFEYVKEFKKVNQGRVWSIGPVSLSNKTALDKAQRGNMASIDENKC<br>LNWLGSWPQSSVVYACLGSLSQVTTLQLVELGLGLEASNRPFVWVISRNKIDE<br>WEKWLLEDGFEERINGRGLLIHGWAPQVLILSHPAVGGFLTHCGWNSTLEGICA<br>GIPMITWPLFAEQFYNEKFIVQVLKIGESVGAKVAIPLGEQEISKVLVKRVEFKE<br>AIDKVMIKEGKEGEDRRKRARELAVLAKKATEGRGSSYLNMTLLIEDIRSCKAN<br>N* |

**Table S12. Summary of *S. grosvenorii* datasets used for transcriptome analysis**

| Sample tissues | Groups | Descriptions | BioProjects |
|---|---|---|---|
| fruit-1 | a | 3 days after anthesis | PRJEB23466 |
| fruit-2 | a | 3 days after anthesis | PRJEB23466 |
| fruit-3 | a | 5 days after anthesis | PRJNA773651 |
| fruit-4 | a | 5 days after anthesis | PRJNA773651 |
| fruit-5 | a | 5 days after anthesis | PRJNA773651 |
| fruit-6 | a | 10 days after anthesis | PRJNA773651 |
| fruit-7 | a | 10 days after anthesis | PRJNA773651 |
| fruit-8 | a | 10 days after anthesis | PRJNA773651 |
| fruit-9 | a | 15 days after anthesis | PRJNA773651 |
| fruit-10 | a | 15 days after anthesis | PRJNA773651 |
| fruit-11 | a | 15 days after anthesis | PRJNA773651 |
| fruit-12 | a | 20 days after anthesis | PRJNA773651 |
| fruit-13 | a | 20 days after anthesis | PRJNA773651 |
| fruit-14 | a | 20 days after anthesis | PRJNA773651 |
| fruit-15 | a | 20 days after anthesis | PRJEB23466 |
| fruit-16 | a | 20 days after anthesis | PRJEB23466 |
| fruit-17 | a | 25 days after anthesis | PRJNA773651 |
| fruit-18 | a | 25 days after anthesis | PRJNA773651 |
| fruit-19 | a | 25 days after anthesis | PRJNA773651 |
| fruit-20 | a | 30 days after anthesis | PRJNA773651 |
| fruit-21 | a | 30 days after anthesis | PRJNA773651 |
| fruit-22 | a | 30 days after anthesis | PRJNA773651 |
| fruit-23 | a | 35 days after anthesis | this study |
| fruit-24 | a | 35 days after anthesis | this study |
| root-1 | b | roots | PRJEB23466 |
| root-2 | b | roots | PRJEB23466 |
| root-3 | b | roots | PRJEB23466 |
| root-4 | b | young roots | this study |
| root-5 | b | young roots | this study |
| root-6 | b | young roots | this study |
| stem-1 | c | mature stems | this study |
| stem-2 | c | mature stems | this study |
| stem-3 | c | mature stems | this study |
| leaves-1 | d | leaves beside fruits | PRJEB23466 |
| leaves-2 | d | leaves beside fruits | PRJEB23466 |
| leaves-3 | d | leaves of female plants | PRJEB23466 |
| leaves-4 | d | leaves of female plants | PRJEB23466 |
| leaves-5 | d | leaves of female plants | PRJEB23466 |
| leaves-6 | d | leaves of male plants | PRJEB23466 |
| leaves-7 | d | leaves of male plants | PRJEB23466 |
| leaves-8 | d | leaves of male plants | PRJEB23466 |
| bud-1 | e | bud | PRJNA1209606 |
| bud-2 | e | bud | PRJNA1209606 |
| bud-3 | e | bud | PRJNA1209606 |
| bud-4 | e | bud | PRJNA1209606 |
| bud-5 | e | bud | PRJNA1209606 |
| bud-6 | e | bud | PRJNA1209606 |

**Table S13. Summary of species included in the comparative analysis, with notes on genome quality and manual curation of UGT73 annotations**

| Clade | Family | Species | Genome quality | Manual curation | UGT73 tandem array | UGT73 copy number |
|---|---|---|---|---|---|---|
| Core eudicots | Brassicaceae | *Arabidopsis thaliana* | chromosome | No | √ | 6 |
| | Datiscaceae | *Datisca glomerata* | scaffold | Yes | × | 0 (approx.) |
| | Begoniaceae | *Begonia loranthoides* | chromosome | Yes | × | 0 (approx.) |
| | Cucurbitaceae | *Begonia darthvaderiana* | chromosome (contaminated) | No | √ | 4 |
| | | *Begonia peltatifolia* | chromosome | No | √ | 2 (approx.) |
| | | *Begonia masoniana* | chromosome | No | √ | 2 |
| | | *Citrullus lanatus* | chromosome | No | √ | 7 |
| | | *Cucumis sativus* | chromosome | Yes | √ | 5 |
| | | *Cucurbita pepo* | chromosome | Yes | √ | 3 (approx.) |
| | | *Cucurbita moschata* | chromosome | No | √ | 4 |
| | | *Herpetospermum pedunculosum* | chromosome | Yes | √ | 6 |
| | | *Trichosanthes anguina* | chromosome | No | √ | 7 |
| | | *Sicyos edulis* | chromosome | No | √ | 7 |
| | | *Momordica charantia* | chromosome | Yes | √ | 6 |
| | | *Siraitia grosvenorii* | chromosome | No | √ | 9 |
| | | *Thladiantha pustulata* | chromosome | No | √ | 4 |
| | | *Gynostemma pentaphyllum* | chromosome | No | √ | 6 |
| | Coriariaceae | *Coriaria nepalensis* | chromosome | No | √ | 3 |
| | Juglandaceae | *Cyclocarya paliurus* | chromosome | Yes | √ | 6 |
| | Juglandaceae | *Juglans regia* | chromosome | Yes | √ | 4 |
| | Betulaceae | *Corylus avellana* | chromosome | Yes | √ | 10 |
| | Rosaceae | *Prunus persica* | chromosome | No | √ | 7 |
| | Cannabaceae | *Cannabis sativa* | chromosome | No | √ | 11 |
| | Fabaceae | *Glycine max* | chromosome | No | √ | 6 |
| | | *Astragalus membranaceus* | chromosome | No | √ | 9 |
| | Vitaceae | *Vitis vinifera* | chromosome | No | √ | 4 |
| | Rubiaceae | *Coffea arabica* | chromosome | No | √ | 4 |
| | Lamiaceae | *Prunella vulgaris* | chromosome | Yes | √ | 3 |
| | Asteraceae | *Stevia rebaudiana* | chromosome | Yes | √ | 3 |
| | Araliaceae | *Aralia elata* | chromosome | No | √ | 6 |
| | Campanulaceae | *Platycodon grandiflorus* | chromosome | Yes | √ | 11 |
| | Apiaceae | *Centella asiatica* | chromosome | No | √ | 9 |
| | | *Daucus carota* | chromosome | No | √ | 3 |

| | | | | | | |
|---|---|---|---|---|---|---|
| | Caryophyllaceae | *Saponaria officinalis* | chromosome | No | × | 1 |
| | Chenopodiaceae | *Beta vulgaris* | chromosome | No | × | 1 |
| Basal eudicots | Ranunculaceae | *Coptis chinensis* | chromosome | No | × | 0 |
| | | *Aquilegia coerulea* | scaffold | No | × | 0 |
| | Papaveraceae | *Papaver somniferum* | chromosome | No | × | 1 |
| Monocots | Poaceae | *Oryza sativa* | chromosome | No | × | 0 |
| Magnoliids | Aristolochiaceae | *Aristolochia fimbriata* | chromosome | No | × | 0 |
| | Lauraceae | *Persea americana* | chromosome | No | × | 0 |
| ANA-grade | Nymphaeaceae | *Nymphaea colorata* | chromosome | No | × | 0 |

Note: In this table, copy numbers are marked as "approx." for species with highly fragmented assemblies or incomplete gene models. To assess the robustness of our findings to genome data quality, we conducted a sensitivity analysis using a subset of nine species with chromosome-level assemblies and high BUSCO scores (>91%), as summarized in Figure 6B. Manual curation of the syntenic block "Oxred-PE-UGT73-TLP" confirmed that the key observations—retention of this region and consistency in tandem duplication patterns—remained stable across variations in genome data quality.

**References**

[1] a) J. M. Augustin, S. Drok, T. Shinoda, K. Sanmiya, J. K. Nielsen, B. Khakimov, C. E. Olsen, E. H. Hansen, V. Kuzina, C. T. Ekstrøm, T. Hauser, S. Bak, *Plant Physiol.* **2012**, *160* (4), 1881, https://doi.org/10.1104/pp.112.202747; b) B. Petit, A.-C. Mitaine-Offer, C. Delaude, T. Miyamoto, C. Tanaka, M.-A. Lacaille-Dubois, *Phytochemistry* **2019**, *162*, 260, https://doi.org/10.1016/j.phytochem.2019.03.020.

[2] Y. Duan, W. Du, Z. Song, R. Chen, K. Xie, J. Liu, D. Chen, J. Dai, *Acta Pharmaceutica Sinica B* **2023**, *13* (1), 271, https://doi.org/10.1016/j.apsb.2022.05.015.
