# Supplementary material for: Tandem Duplication‐Driven Neofunctionalization of UDP‐Glycosyltransferases Shapes the Diversification of Triterpenoid Saponins in the Cucurbitaceae
Source: Adv Sci (Weinh). 2026 Jun 18:e76159. Online ahead of print. doi: 10.1002/advs.76159 (PMC13336362; doi:10.1002/advs.76159)
Supplement: Supplementary file 1 — Supporting File: advs76159‐sup‐0001‐SuppMat.pdf. [file ADVS-9999-e76159-s001.pdf]

Supporting Information

Tandem duplication-driven neo-functionalization of UDP-glycosyltransferases shapes the diversification of triterpenoid saponins in the Cucurbitaceae

Guangyi Wang, Mengmeng Li, Xuehui Dai, Yanchen Zhang, Yuhan Wu, Zhaotao Yan, Chenfei Tian, Hongkai Fan, Haili Liu, Xiaowei Zhang, Yiming Yang, Jiayu Lu, Yuwei Sun, Yong Wang**

1. Supporting Figures S1-S69
2. Supporting Tables S1-S13

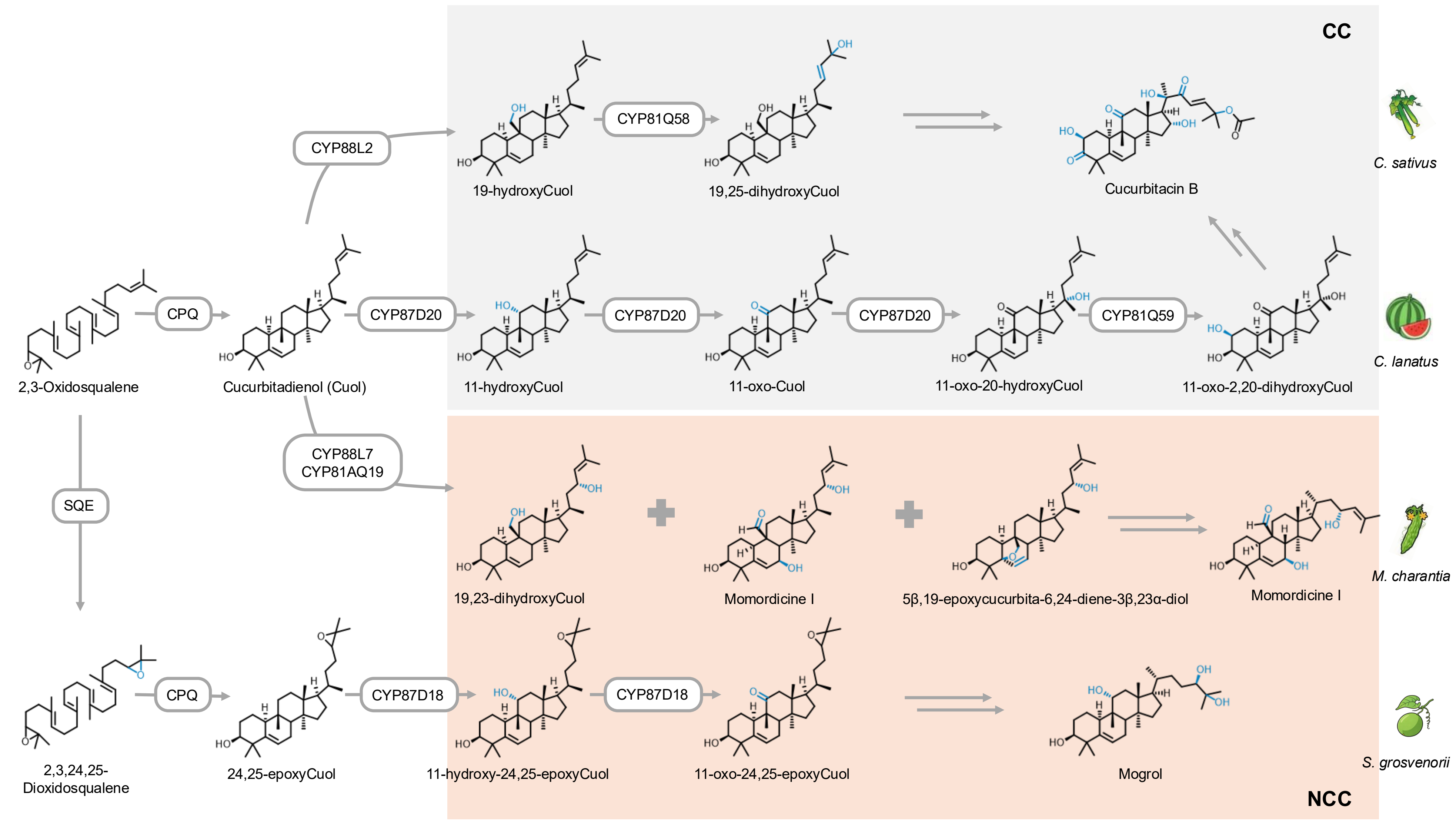

Figure S1. Previously characterized oxidation steps in the Cucurbitaceae family.

CC, Classical cucurbitacins; NCC, non-canonical cucurbitacins

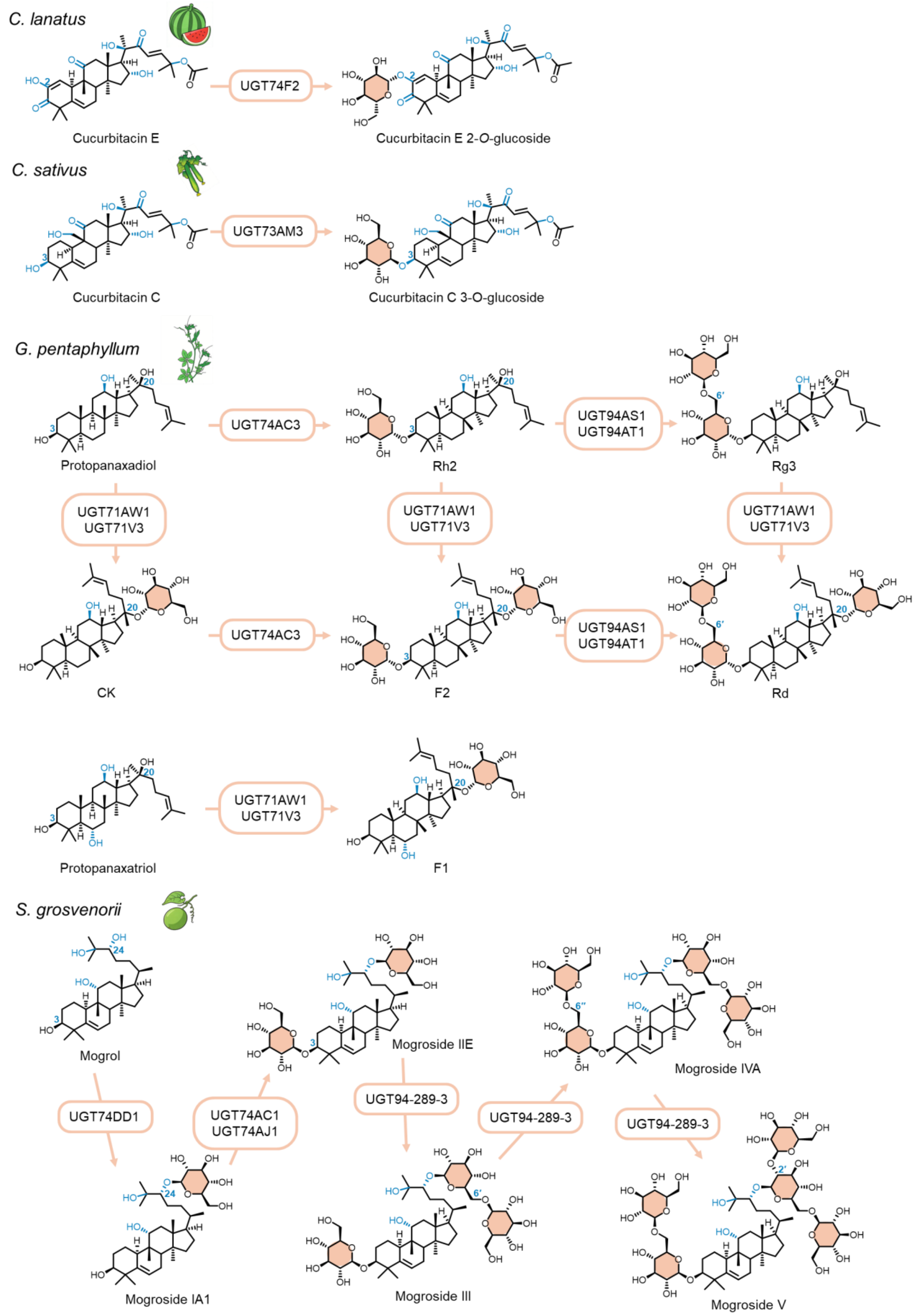

Figure S2. Reported Cucurbitaceae UGTs participating in saponins biosynthesis.

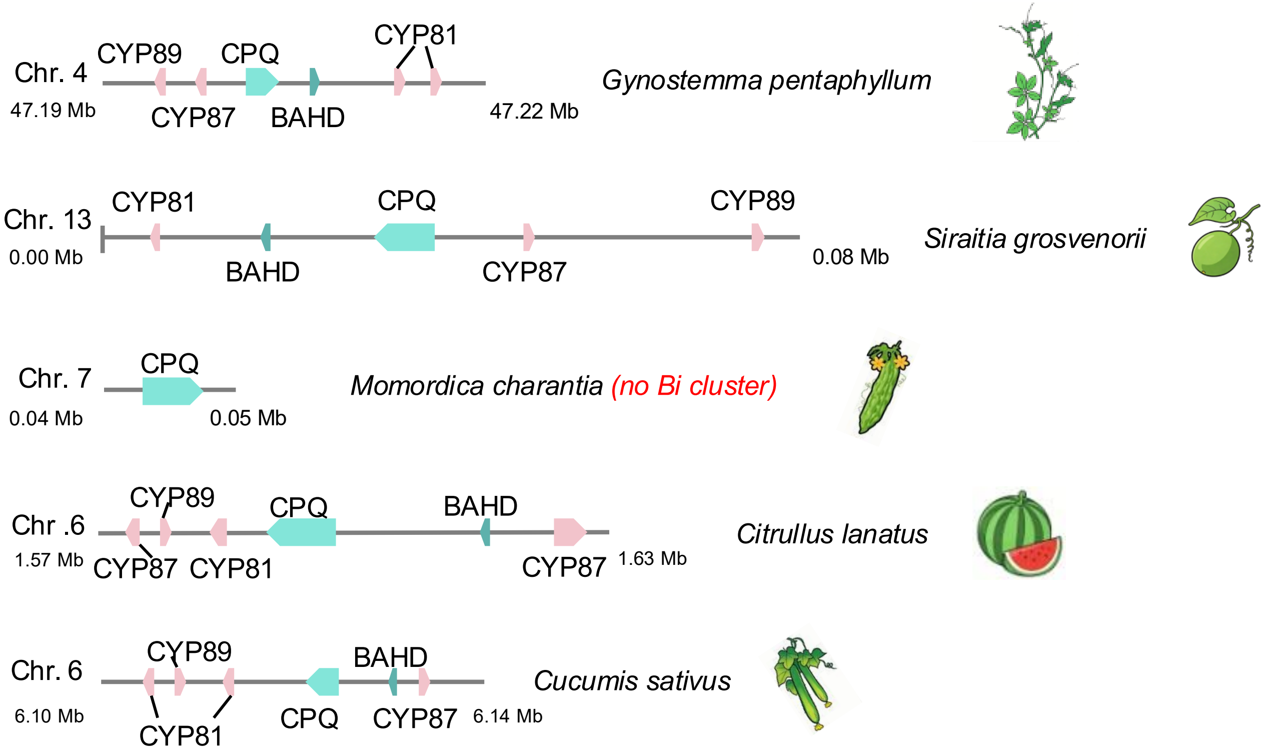

Figure S3. The cucurbitacin biosynthetic gene clusters (BGCs) in different Cucurbitaceae species. The cucurbitacin BGCs are present in most of the cucurbits, except for bitter melon

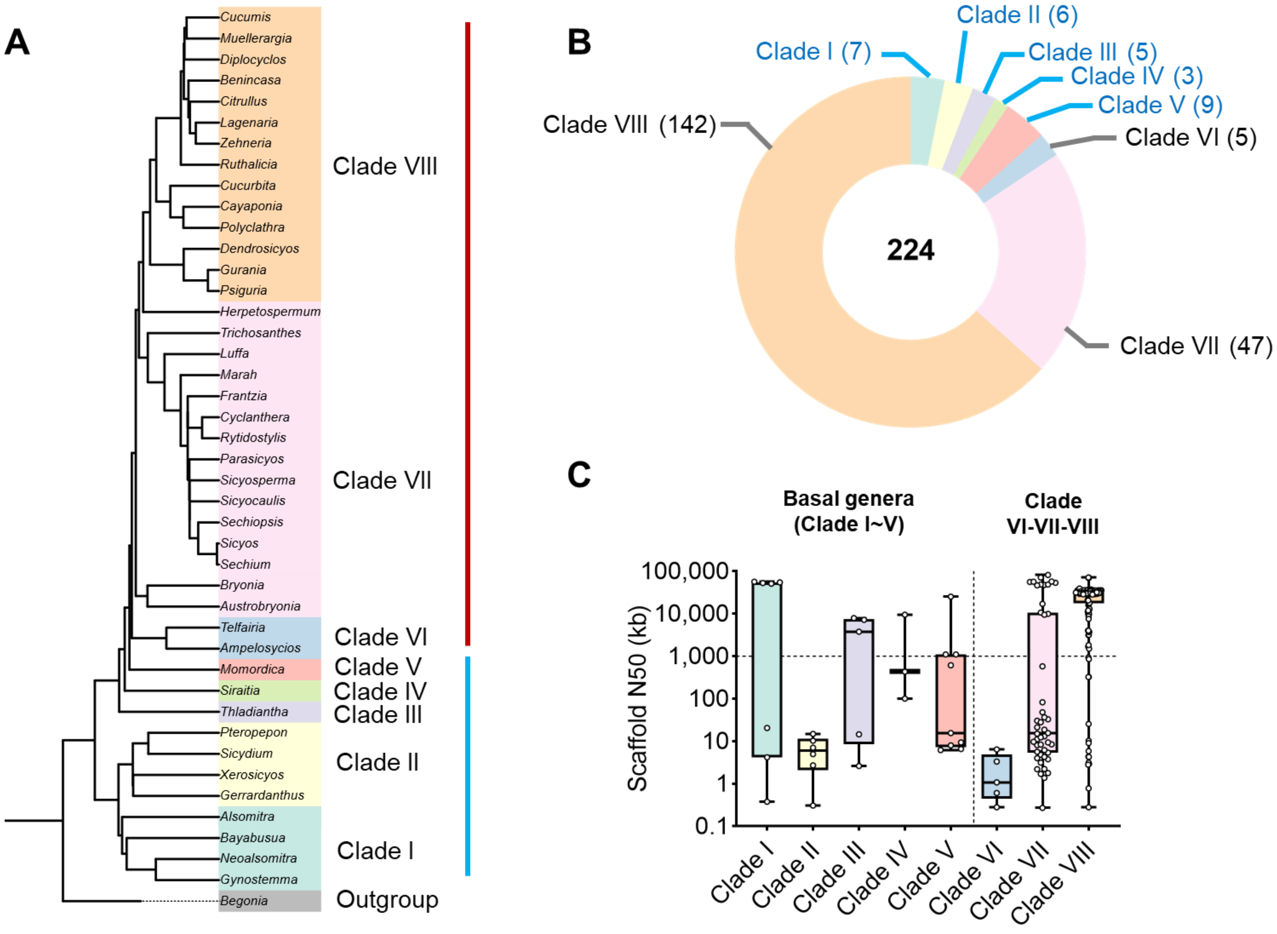

Figure S4. Sequenced genomes of Cucurbitaceae family plants. (**A**) Phylogenetic tree of Cucurbitaceae genera having their genomes sequenced. Blue line, basal genera (Clade I~V); Red line, late-diverged clades (Clade VI-VIII). (**B**) The pie chart shows the number of whole genome sequencing projects in different clades. The basal genera clades are indicated in blue. (**C**) Scaffold N50 of the assembly represents the quality of Cucurbitaceae genomes. Those with scaffold N50>1000 kb (1 Mb) is considered well assembled. Box plots show the median (center line), interquartile range (box), and full data range (whiskers). Sample sizes (n) for each clade are: I (7), II (6), III (5), IV (3), V (9), VI (5), VII (47), VIII (142).

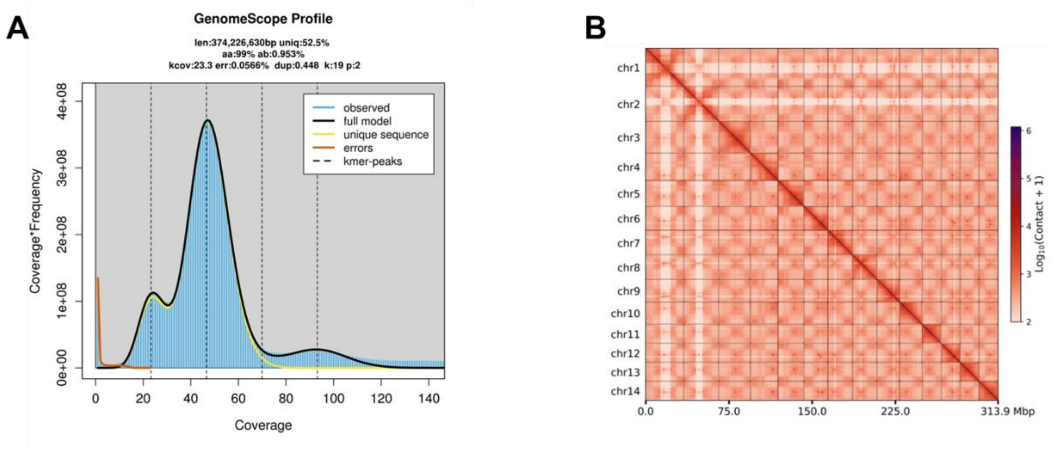

Figure S5. Estimation of genome size and chromatin interactions in *S. grosvenorii*. (**A**) Genome size estimation of *S. grosvenorii* based on 19-mer frequency analysis. (**B**) Hi-C interaction heatmap of the *S. grosvenorii* chromosomes.

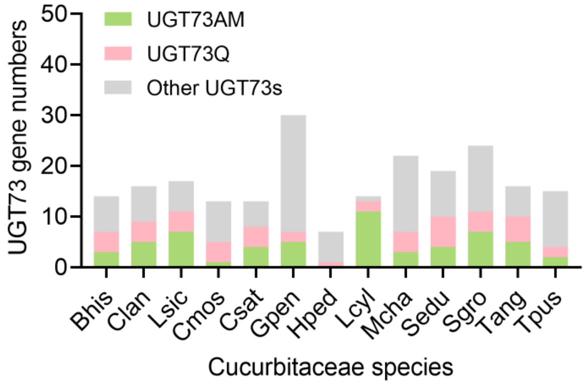

	Bhis	Clan	Lsic	Cmos	Csat	Gpen	Hped	Lcyl	Mcha	Sedu	Sgro	Tang	Tpus
UGT73	14	16	17	13	13	30	7	14	22	19	24	16	15
UGT73AM	**3**	**5**	**7**	**1**	**4**	**5**	**0**	**11**	**3**	**4**	**7**	**5**	**2**
UGT73Q	**4**	**4**	**4**	**4**	**4**	**2**	**1**	**2**	**4**	**6**	**4**	**5**	**2**
Others	7	7	6	8	5	23	6	1	15	9	13	6	11

Figure S6. Distribution of *UGT73AM* and *UGT73Q* subfamilies genes across Cucurbitaceae species. The table below the column graph lists the gene numbers of the UGT73 family for each analyzed species. Abbreviations: *Benincasa hispida* (Bhis), *C. lanatus* (Clan), *Lagenaria siceraria* (Lsic), *Cucurbita. moschata* (Cmos), *C. sativus* (Csat), *G. pentaphyllum* (Gpen), *H. pedunculosum* (Hped), *Luffa aegyptiaca* (Lcyl), *M. charantia* (Mcha), *Sechium edule* (Sedu), *S. grosvenorii* (Sgro), *Trichosanthes anguina* (Tang) and *T. pustulata* (Tpus).

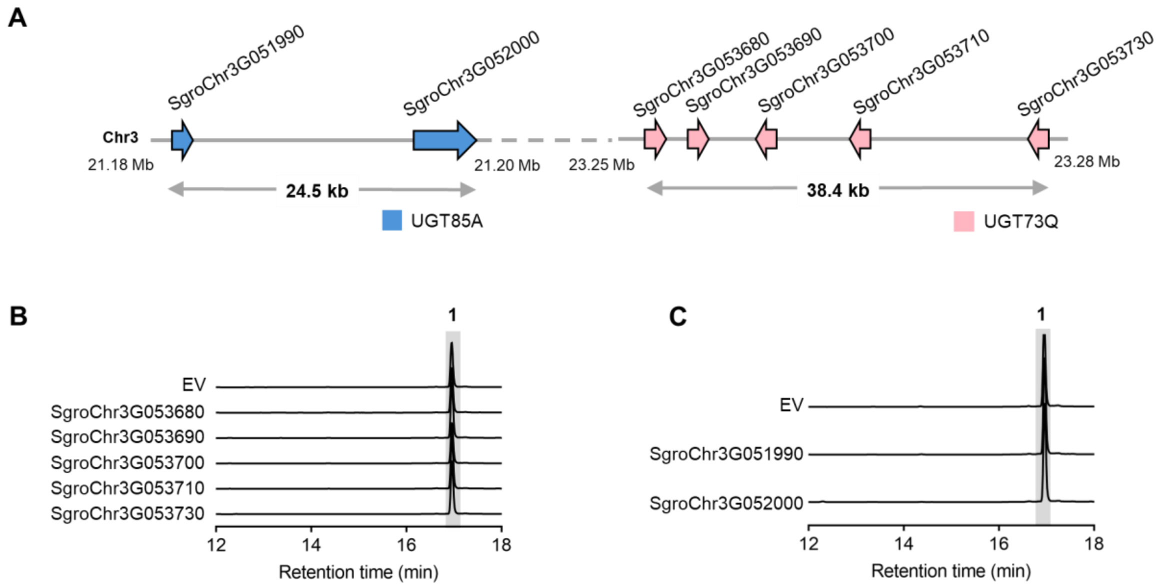

Figure S7. Screening of UGT85A and UGT73Q candidates. (**A**) Tandem array of *UGT73Q* and *UGT85A* genes on chromosome 3. (**B**) HPLC chromatograms of the in vitro assay of SgUGT73Q toward mogrol (**1**) (C) HPLC chromatograms of the in vitro assay of SgUGT85A toward mogrol (**1**). EV, empty vector (pET28a).

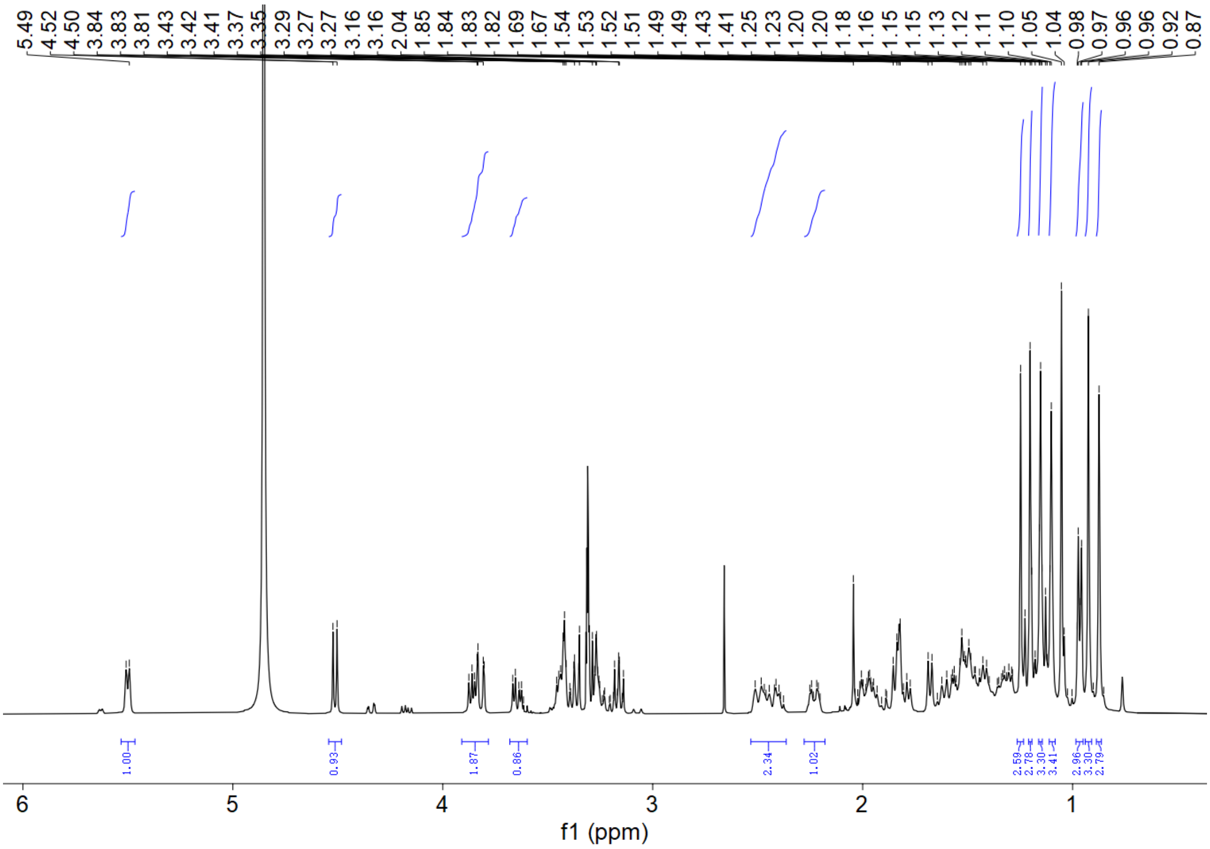

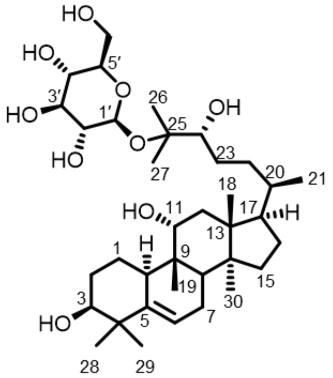

Figure S8. ^{1}H (400 MHz, methanol-d_4) NMR spectrum of mogrol 25-*O*-glucoside (3).

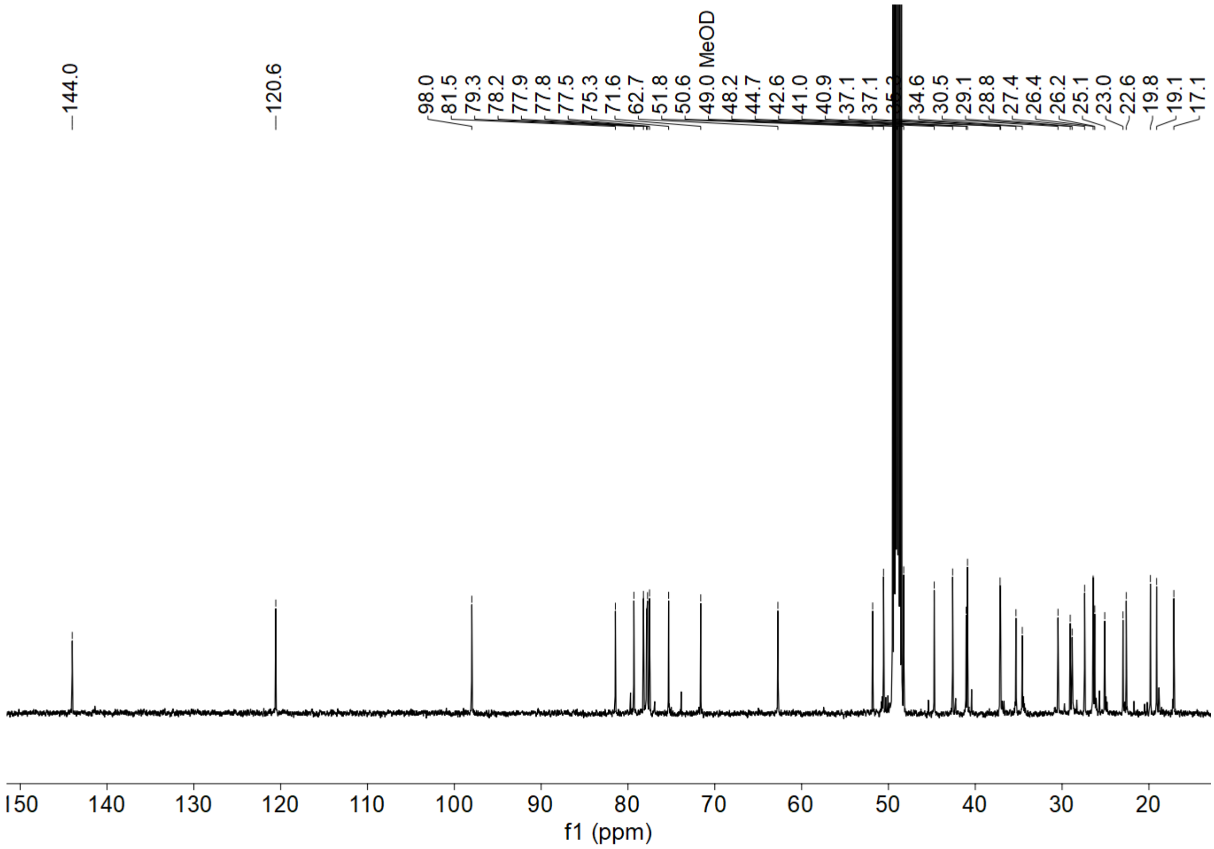

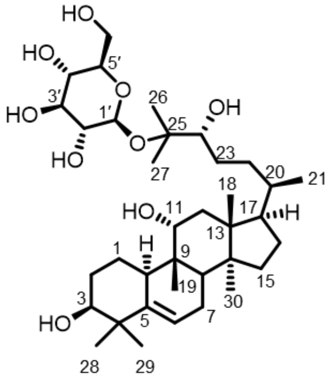

Figure S9. ^{13}C (125 MHz, methanol-*d*$_4$) NMR spectrum of mogrol 25-*O*-glucoside (3). δ_C (ppm): 26.4 (C-1); 30.5 (C-2); 77.5 (C-3); 42.6 (C-4); 144.0 (C-5); 120.6 (C-6); 25.1 (C-7); 44.7 (C-8); 40.9 (C-9); 37.1 (C-10); 79.3 (C-11); 41.1 (C-12); 48.2 (C-13); 50.6 (C-14); 35.3 (C-15); 28.8 (C-16); 51.8 (C-17); 17.1 (C-18); 26.2 (C-19); 37.1 (C-20); 19.1 (C-21); 34.6 (C-22); 29.1 (C-23); 77.9 (C-24); 81.5 (C-25); 22.6 (C-26); 23.0 (C-27); 26.4 (C-28); 27.4 (C-29); 19.8 (C-30); 98.0 (C-1′); 75.3 (C-2′); 78.2 (C-3′); 71.6 (C-4′); 77.8 (C-5′); 62.7 (C-6′).

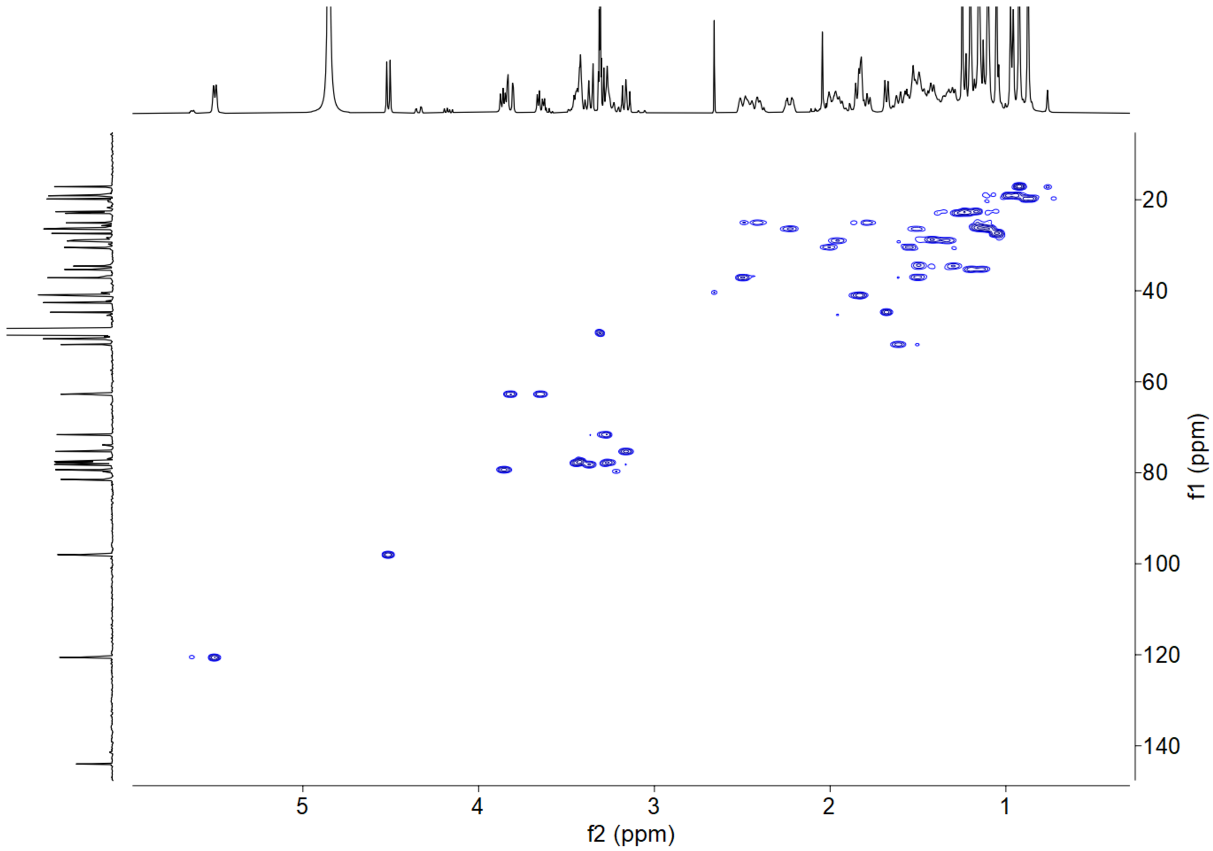

Figure S10. HSQC spectrum of mogrol 25-*O*-glucoside (3).

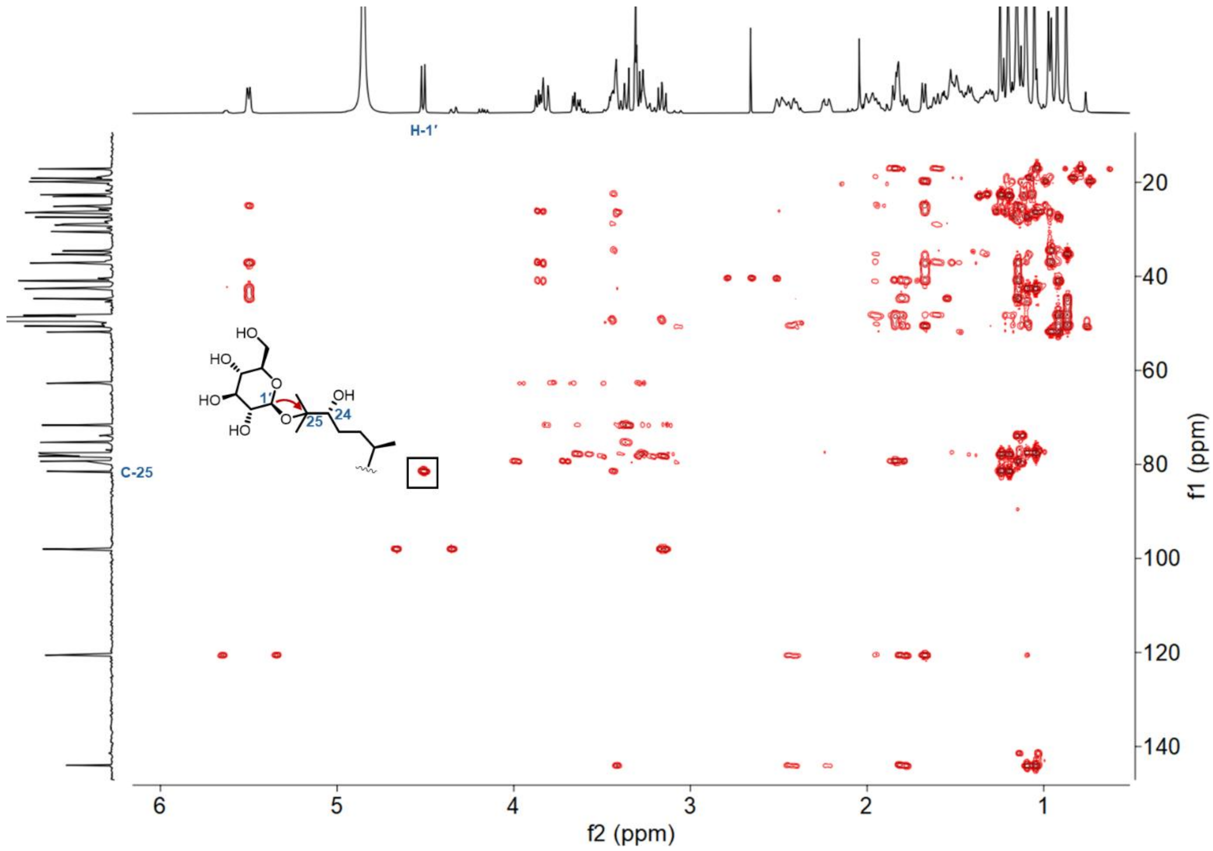

Figure S11. HMBC spectrum of mogrol 25-*O*-glucoside (3).

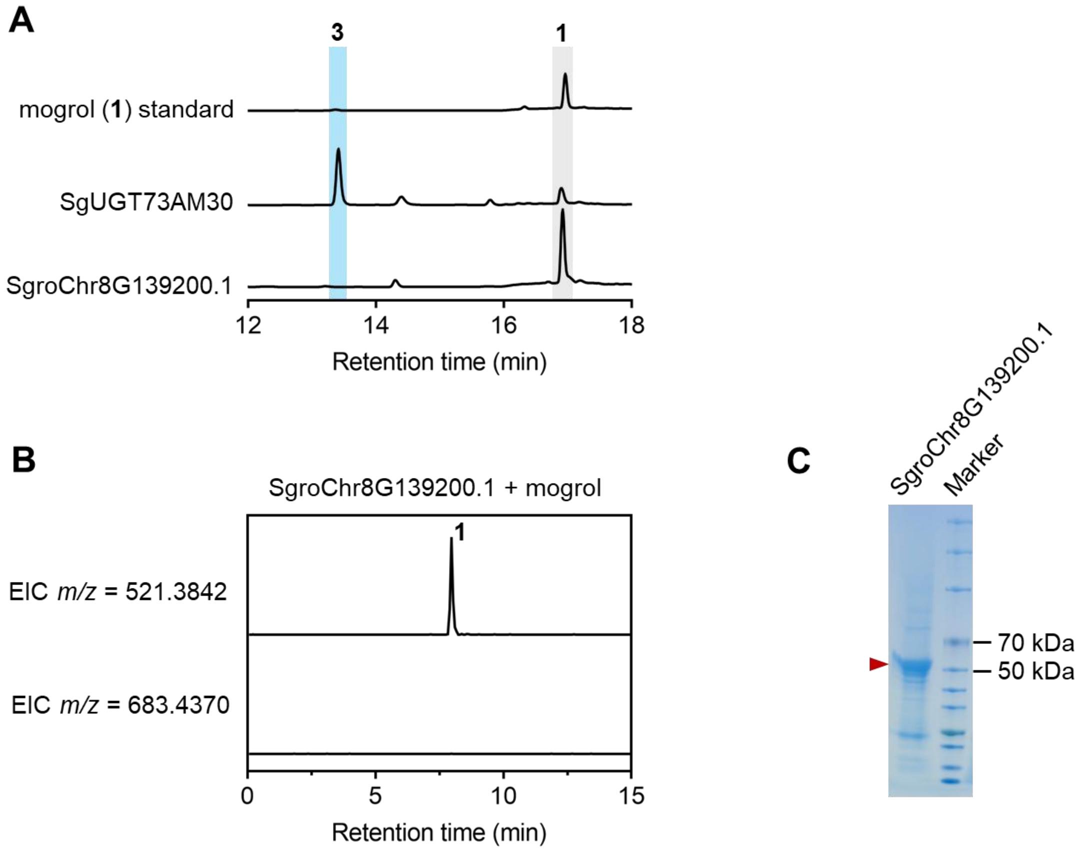

Figure S12. Functional characterization of the previously reported SgUGT73D5 (annotated as SgroChr8G139200.1). (**A**) HPLC-UV chromatogram of SgUGT73D5, showing no detectable glycosylation activity toward substrate **1**. (**B**) LC-MS analysis of SgUGT73D5, confirming the absence of product formation from substrate **1**. (**C**) SDS-PAGE analysis of purified SgUGT73D5 protein.

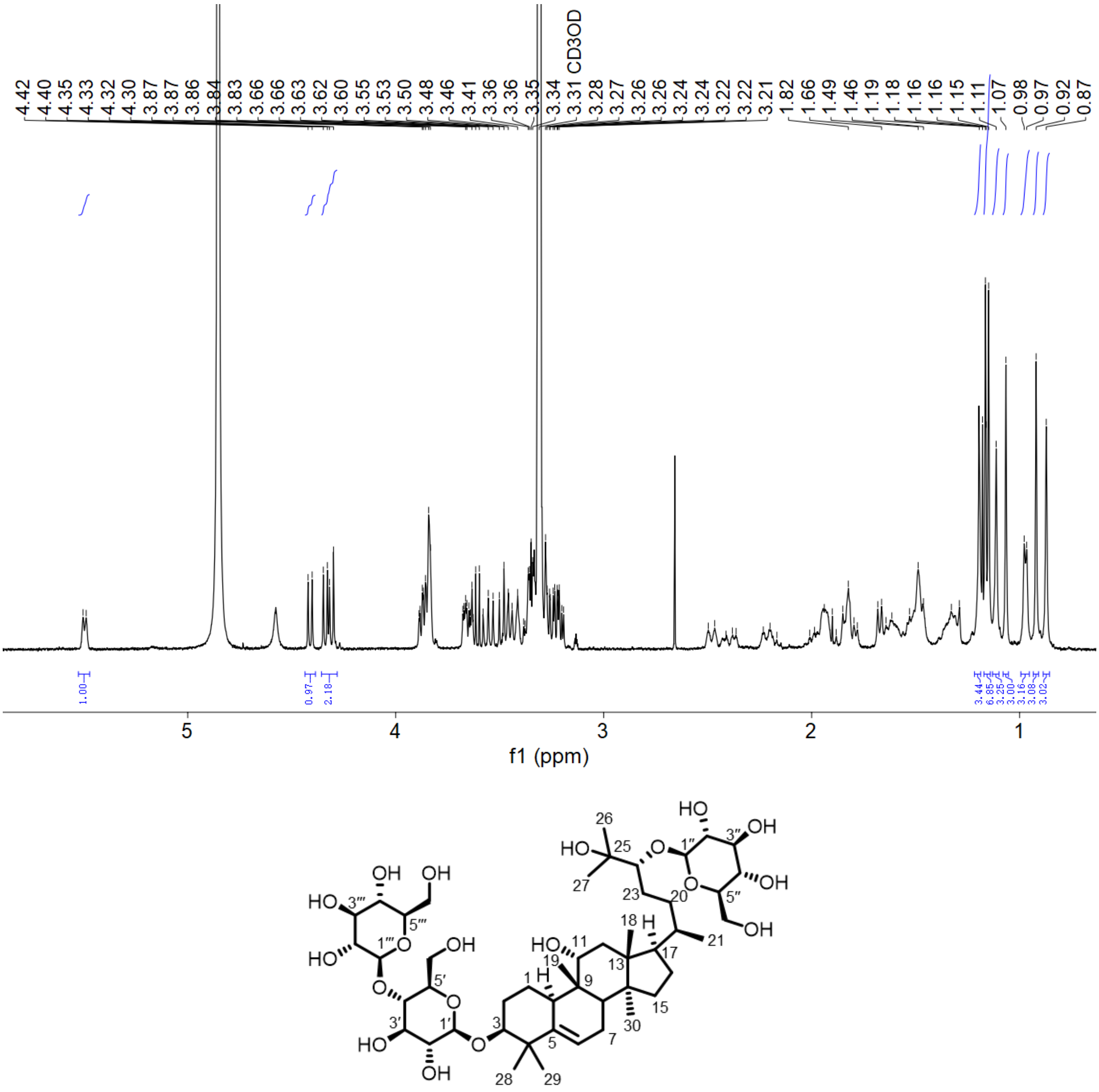

Figure S13. ^{1}H (400 MHz, methanol-d_4) NMR spectrum of mogroside IIE 4′-*O*-glucoside (5).

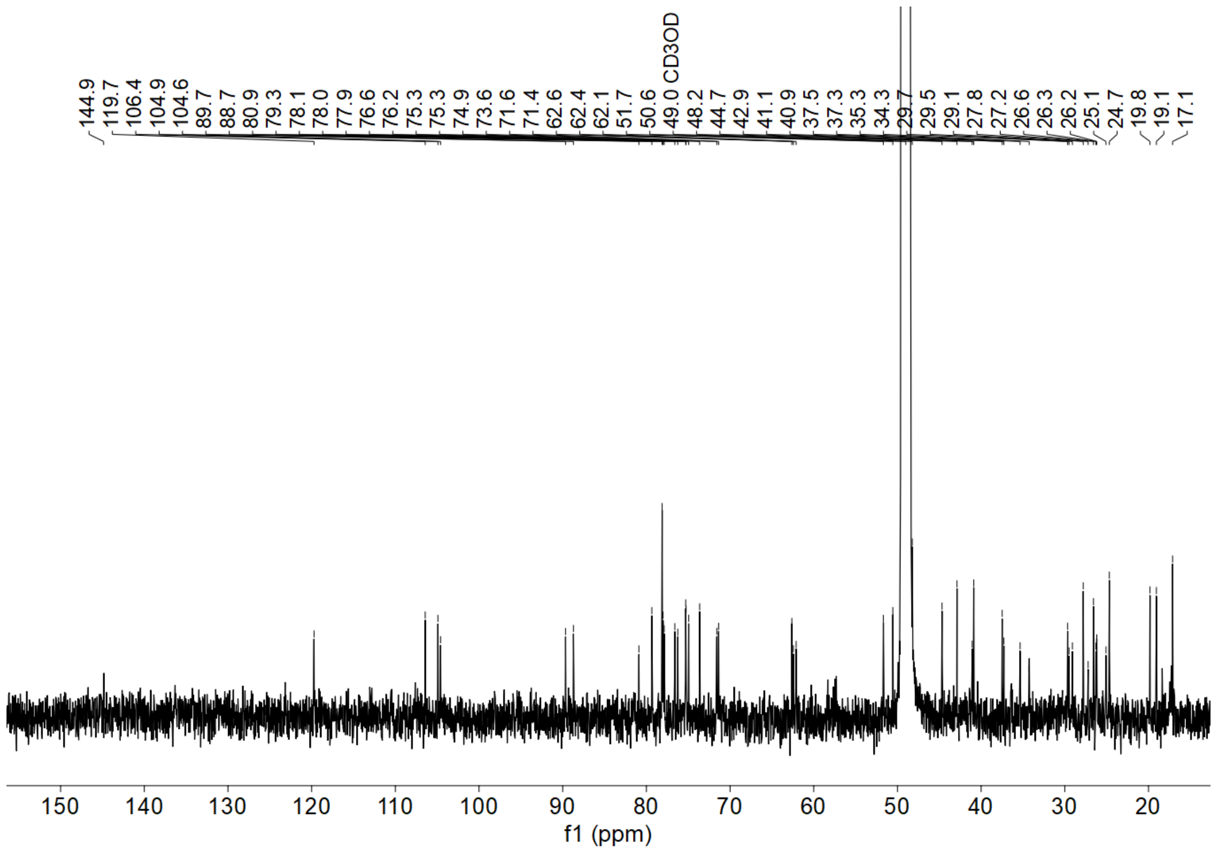

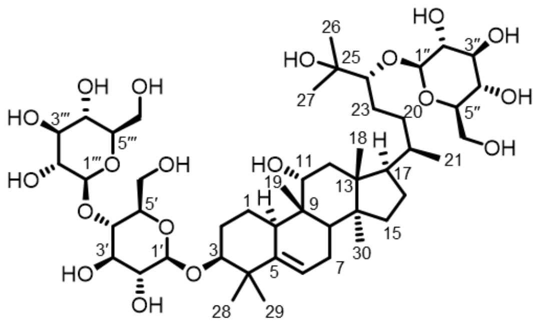

Figure S14. ^{13}C (125 MHz, methanol-d_4) NMR spectrum of mogroside IIE 4′-*O*-glucoside (5). δ_C (ppm): 27.2 (C-1); 29.5 (C-2); 88.7 (C-3); 42.9 (C-4); 144.9 (C-5); 119.7 (C-6); 25.1 (C-7); 44.7 (C-8); 40.9 (C-9); 37.5 (C-10); 79.4 (C-11); 41.1 (C-12); 48.2 (C-13); 50.6 (C-14); 35.4 (C-15); 29.1 (C-16); 51.7 (C-17); 17.2 (C-18); 26.2 (C-19); 37.3 (C-20); 19.1 (C-21); 34.3 (C-22); 29.7 (C-23); 89.7 (C-24); 73.6 (C-25); 26.6 (C-26); 24.7 (C-27); 27.8 (C-28); 26.3 (C-29); 19.8 (C-30); 106.4 (C-1′); 75.3 (C-2′); 76.6(C-3′); 80.9 (C-4′); 78.1 (C-5′); 62.6 (C-6′); 104.9 (C-1″); 75.3 (C-2″); 78.0 (C-3″); 71.6 (C-4″); 78.1 (C-5″); 62.4 (C-6″); 104.6 (C-1‴); 75.0 (C-2‴); 77.9 (C-3‴); 71.4 (C-4‴); 76.3 (C-5‴); 62.1 (C-6‴).

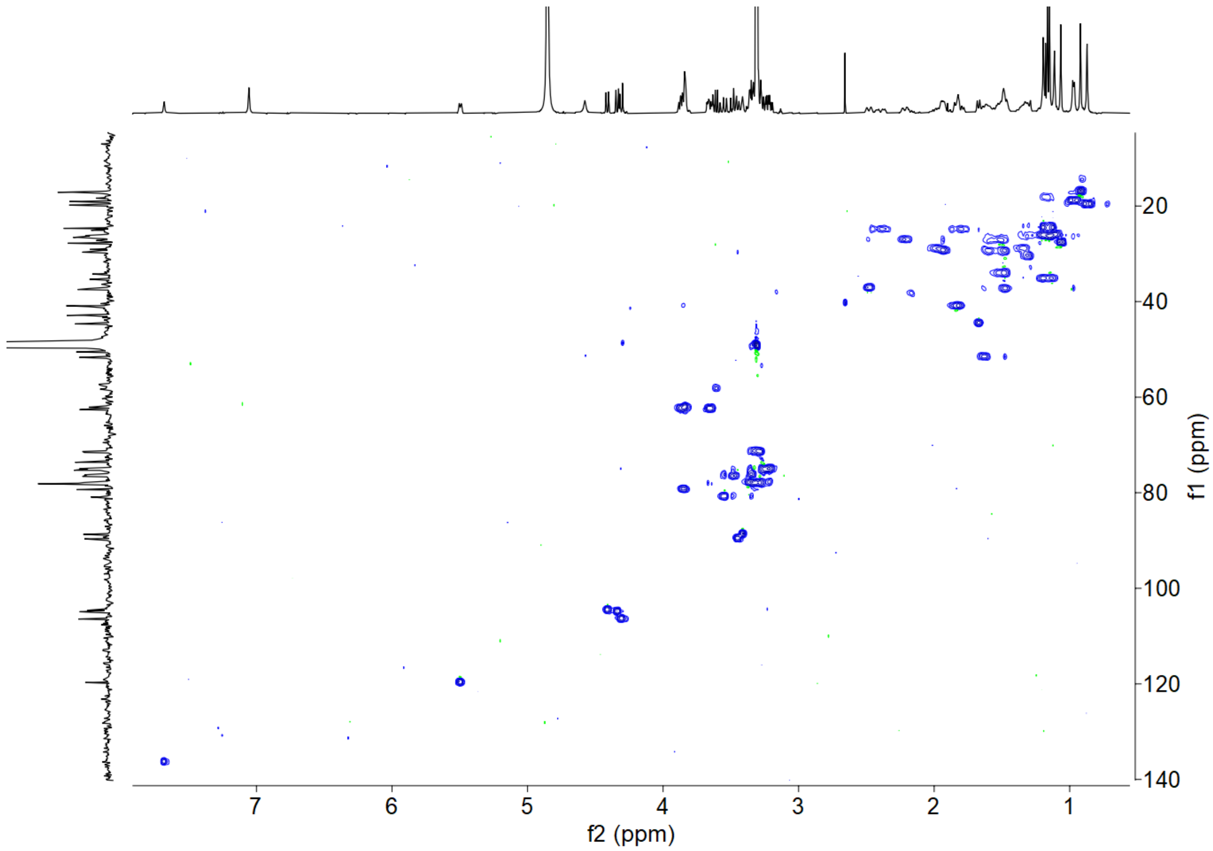

Figure S15. HSQC spectrum of mogroside IIE 4′-*O*-glucoside (5).

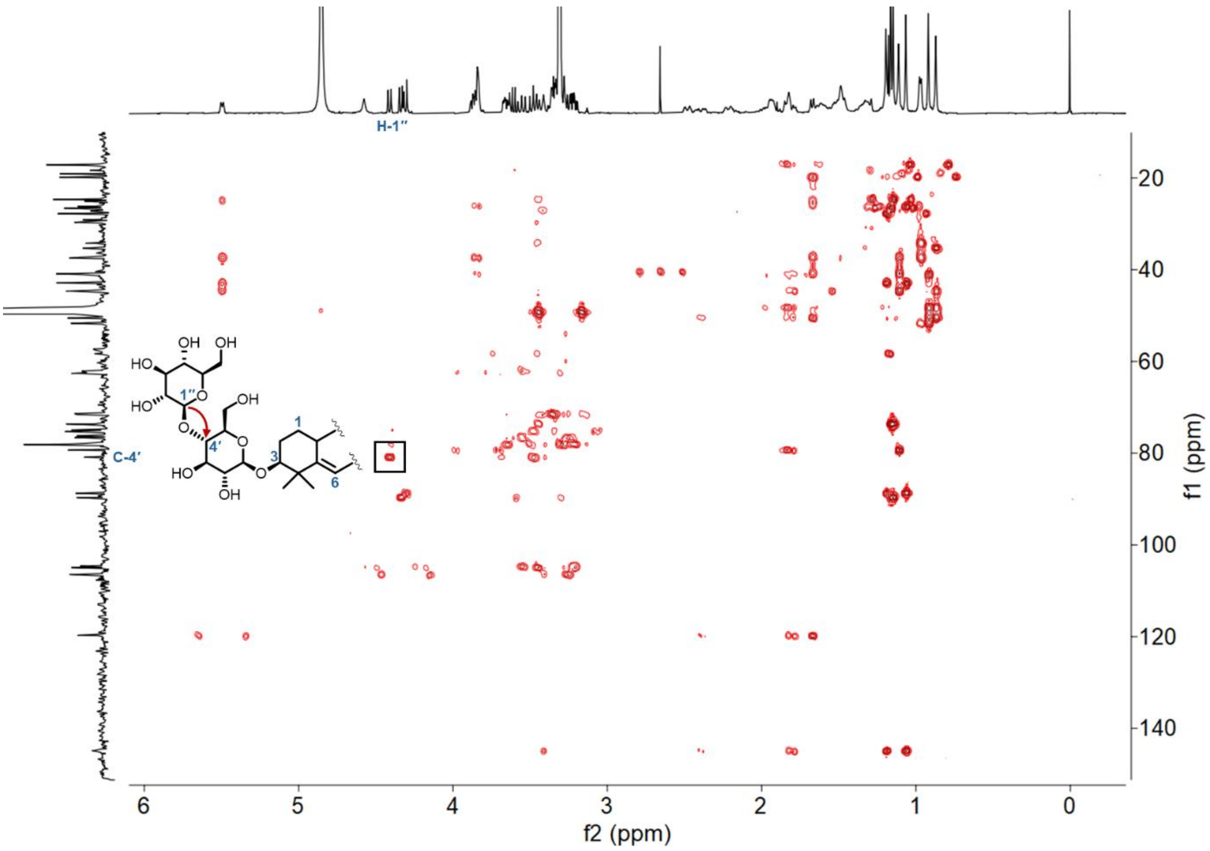

Figure S16. HMBC spectrum of mogroside IIE 4′-*O*-glucoside (5).

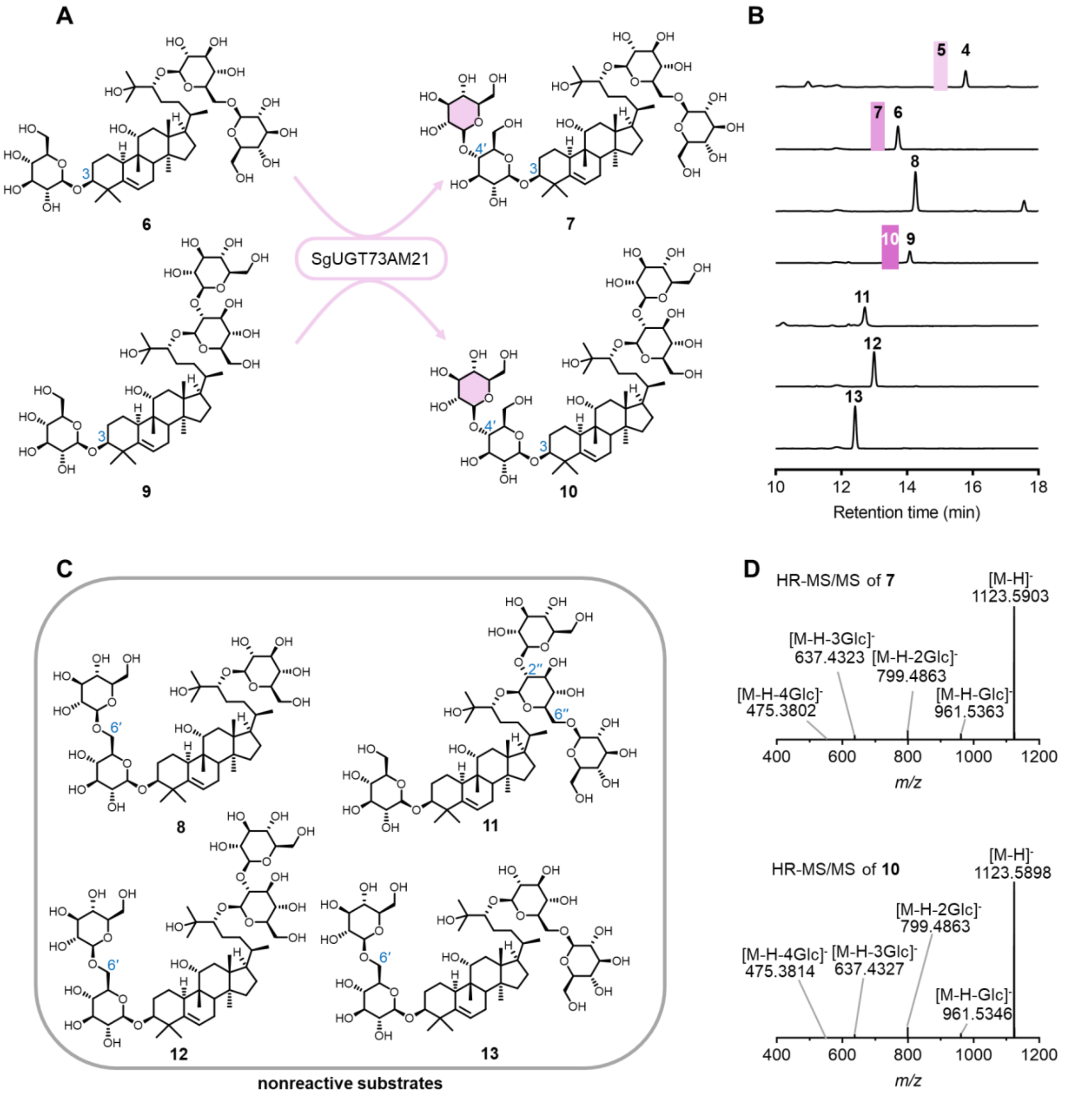

Figure S17. Substrate spectrum of SgUGT73AM21. (**A**) Proposed glycosylation reactions of SgUGT73AM21 towards **6** and **9**. (**B**) HPLC chromatograms showing the *in vitro* assay of SgUGT73AM21 toward **4**, **6**, **8**, **9**, **11**-**13**. (C) Chemical structures of the substrates **8**, **11~13** not recognized by SgUGT73AM21. (**D**) HR-MS/MS spectra of glycosylated products **7** and **10**.

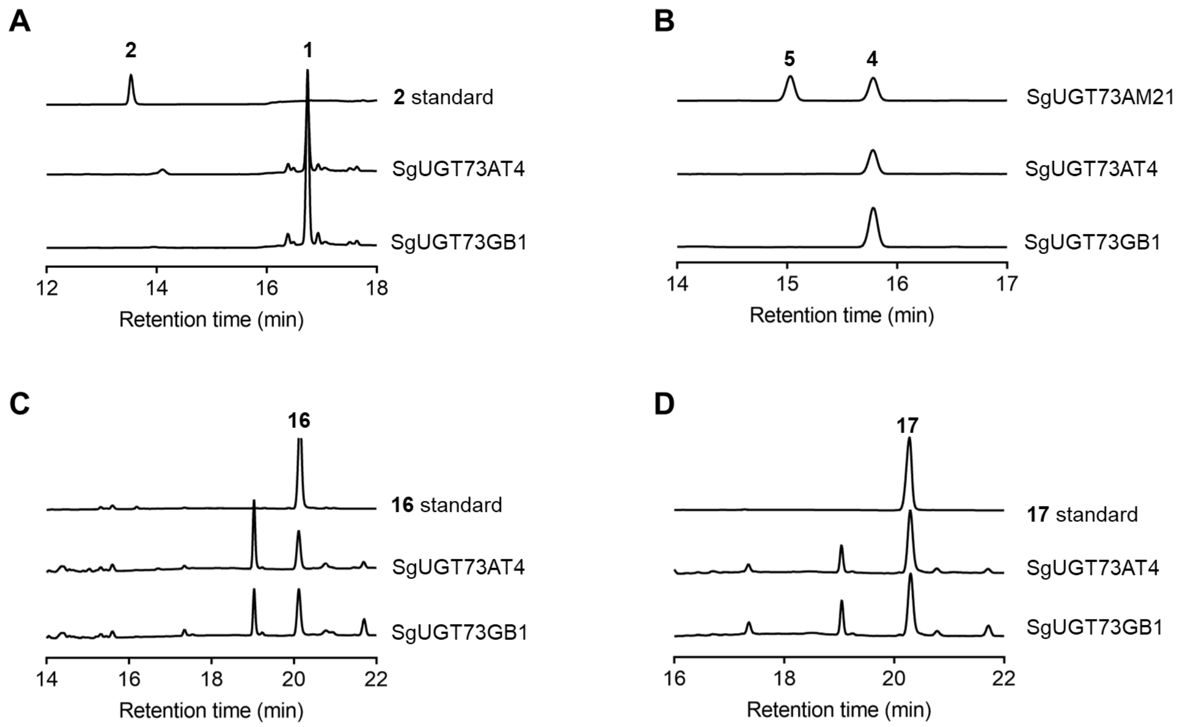

Figure S18. Functional characterization of SgUGT73AT4 and SgUGT73GB1 towards substrates 1 (A), 4 (B), 16 (C) and 17 (D). Neither of the enzymes mentioned above exhibit any activity toward these substrates.

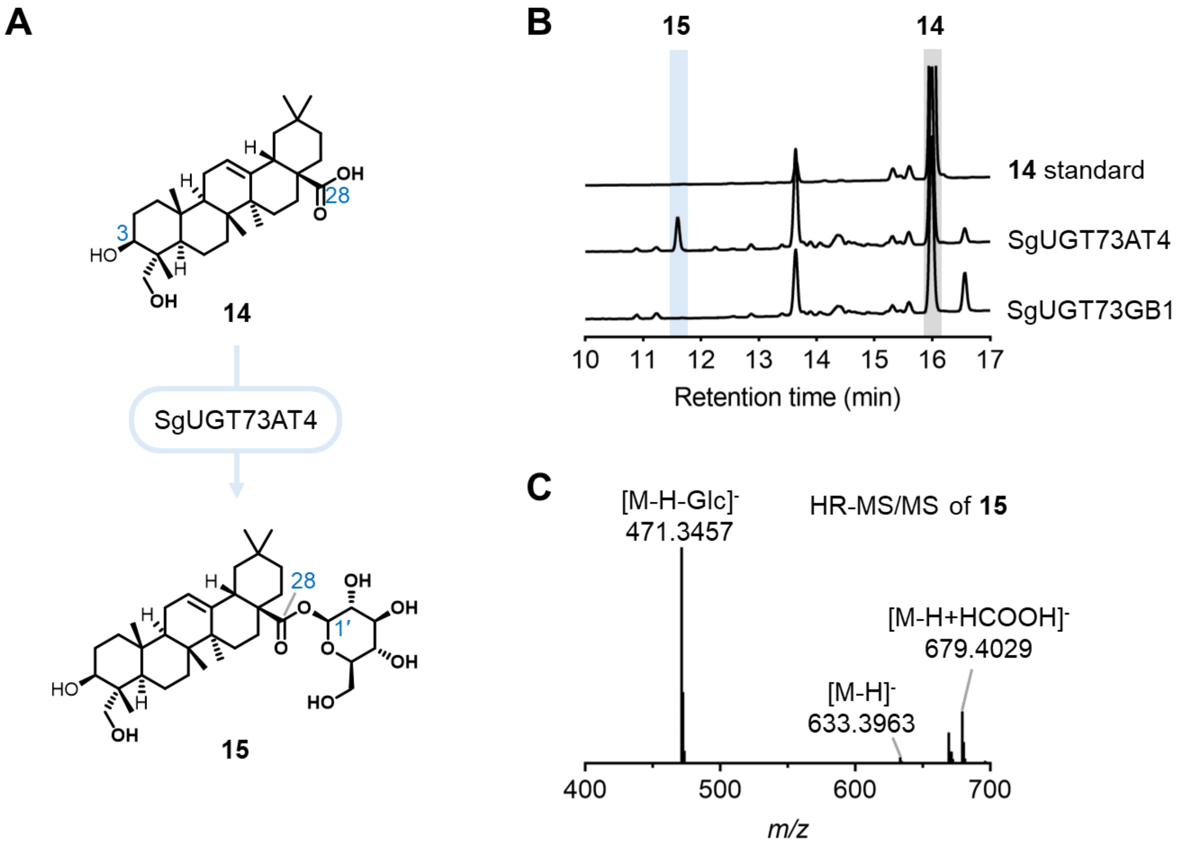

Figure S19. Function of SgUGT73AT4. (**A**) Characterized glycosylation reactions of SgUGT73AT4 towards **14**. (**B**) HPLC chromatograms showing the *in vitro* assay of SgUGT73AT4 and SgUGT73GB1 toward **14**. (C) HR-MS/MS spectra of the glycosylated products **15**.

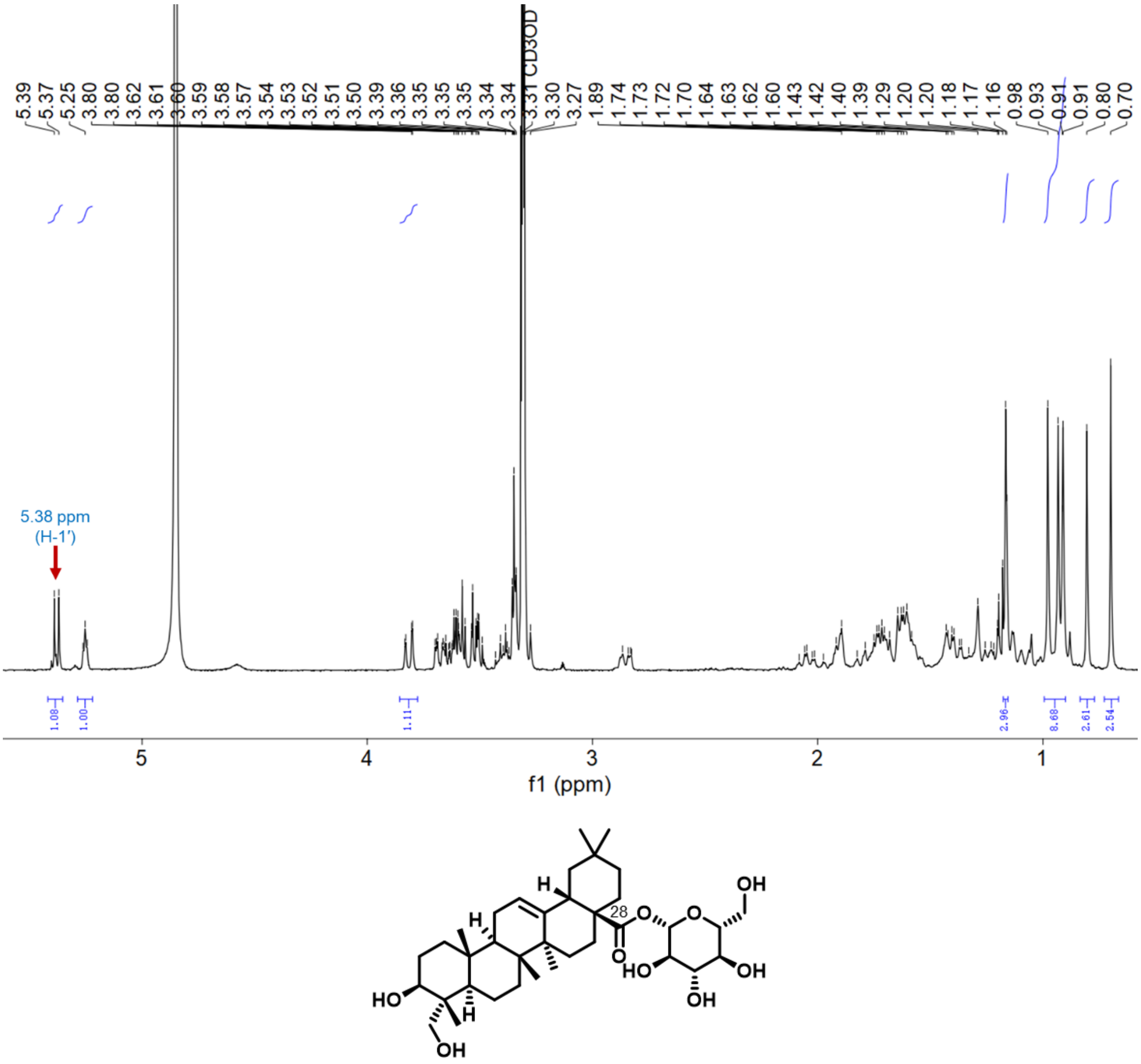

Figure S20. **^{1}H (400 MHz, methanol-*d*4) NMR spectrum of hederagenin 28-*O*-glucoside (15).** The chemical shift of anomeric proton of C28-*O*-β-D-glucose and C3-*O*-β-D-glucose is 5.34 and 4.39 ppm in methanol-*d*4, respectively.

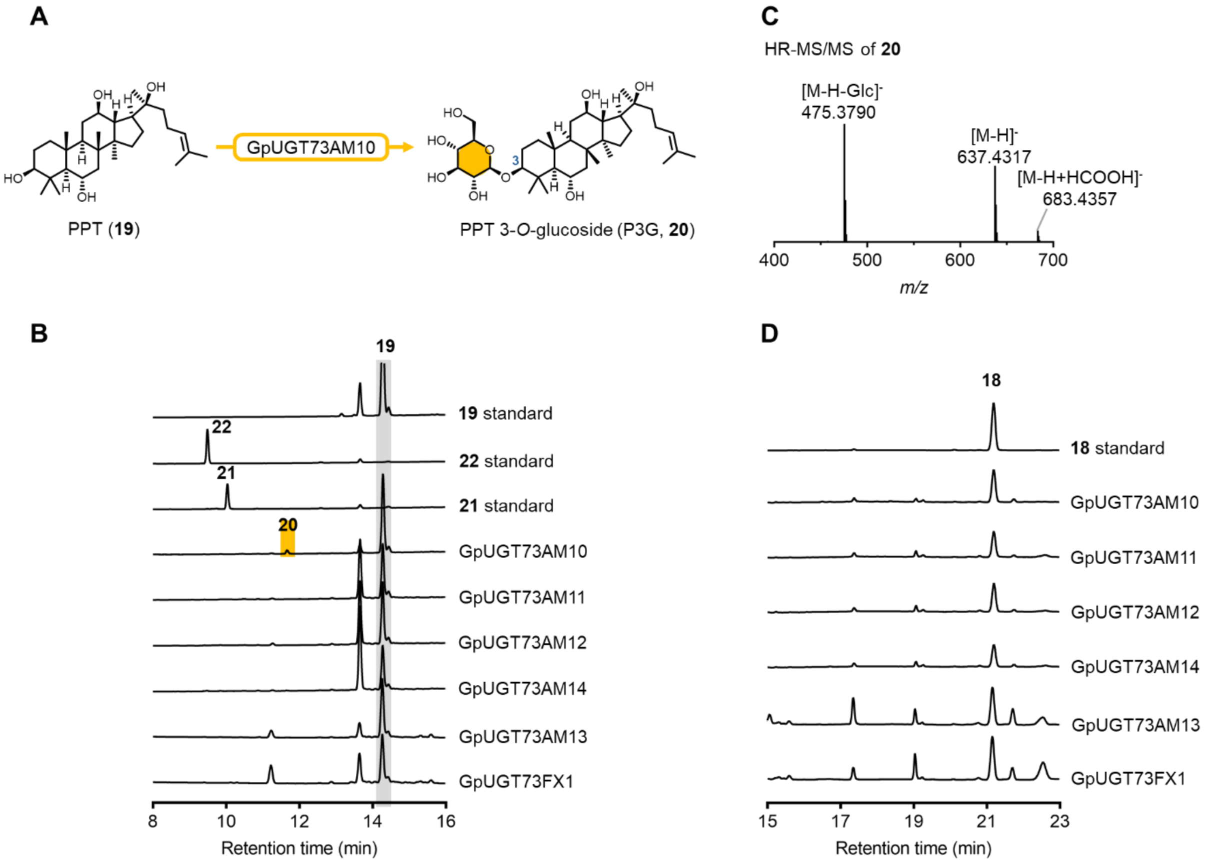

Figure S21. Function of GpUGT73AM10. (**A**) Identified glycosylation reaction of GpUGT73AM10 towards PPT (**19**). (**B**) HPLC chromatograms depicting the *in vitro* assay of GpUGT73AMs and GpUGT73FX1 toward **19**. (C) HR-MS/MS spectra of the glycosylated product **20**. (**D**) HPLC chromatograms revealed that GpUGT73s exhibited no activity against PPD (**18**) *in vitro*.

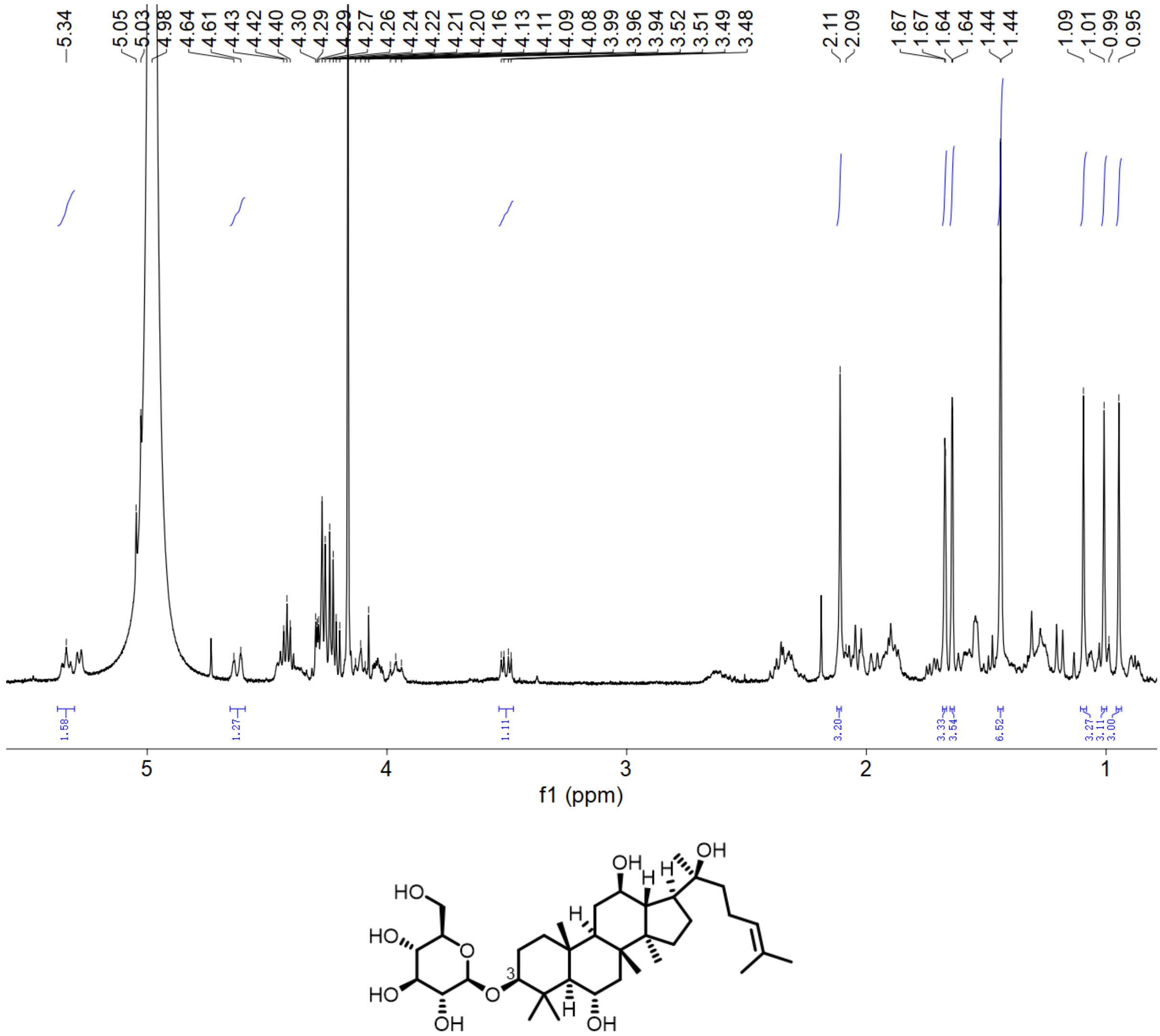

Figure S22. ^{1}H (400 MHz, pyridine-*d*$_5$) NMR spectrum of protopanaxatriol 3-*O*-glucoside (20).

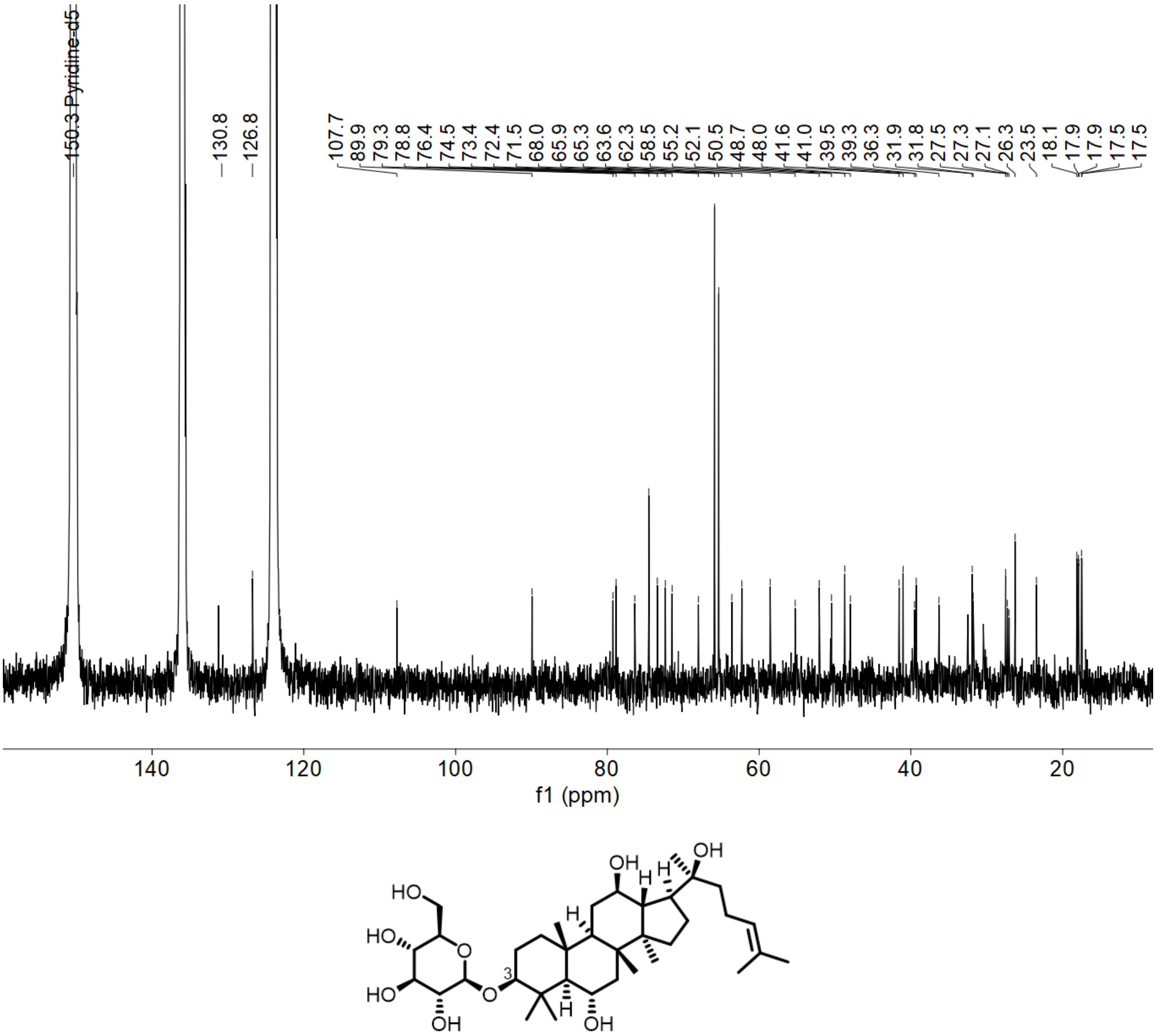

Figure S23. ^{13}C (125 MHz, pyridine-d_5) NMR spectrum of protopanaxatriol 3-*O*-glucoside (20).

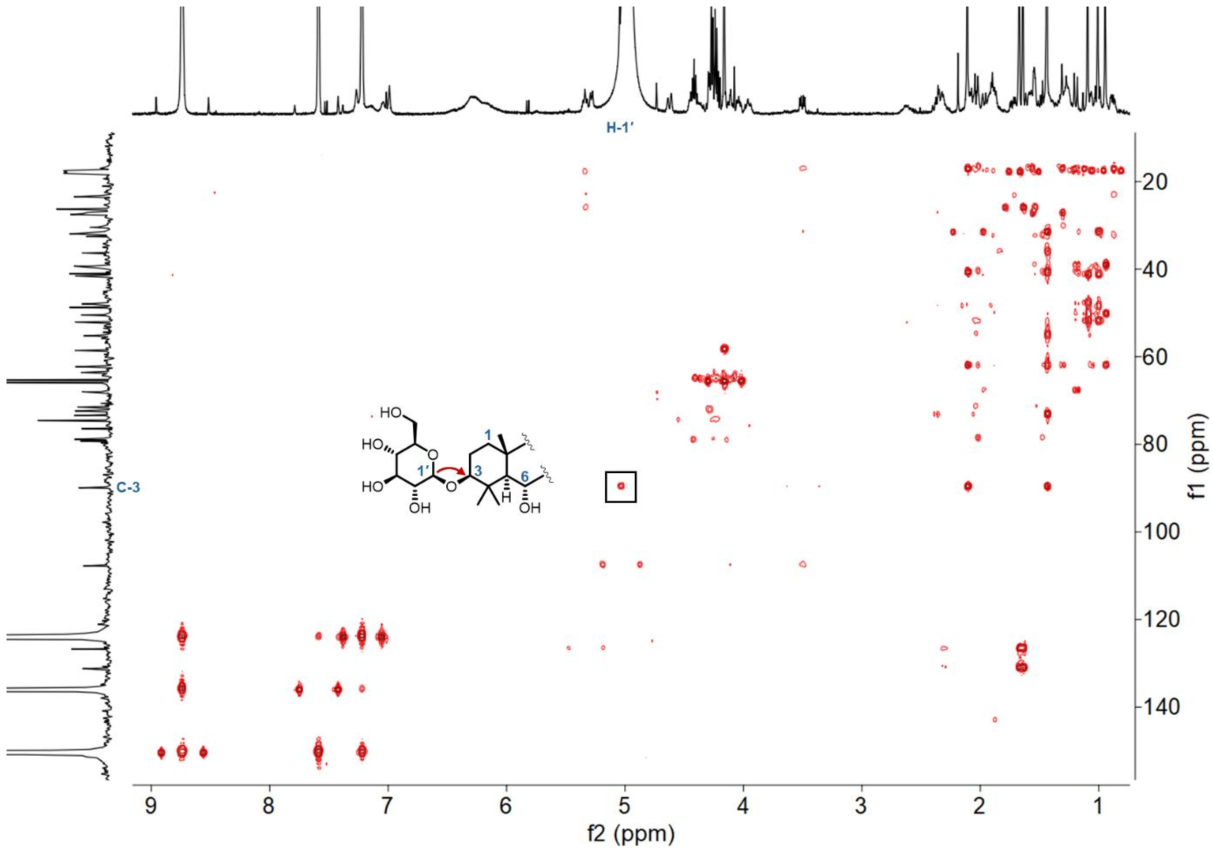

Figure S24. HMBC spectrum of protopanaxatriol 3-*O*-glucoside (20).

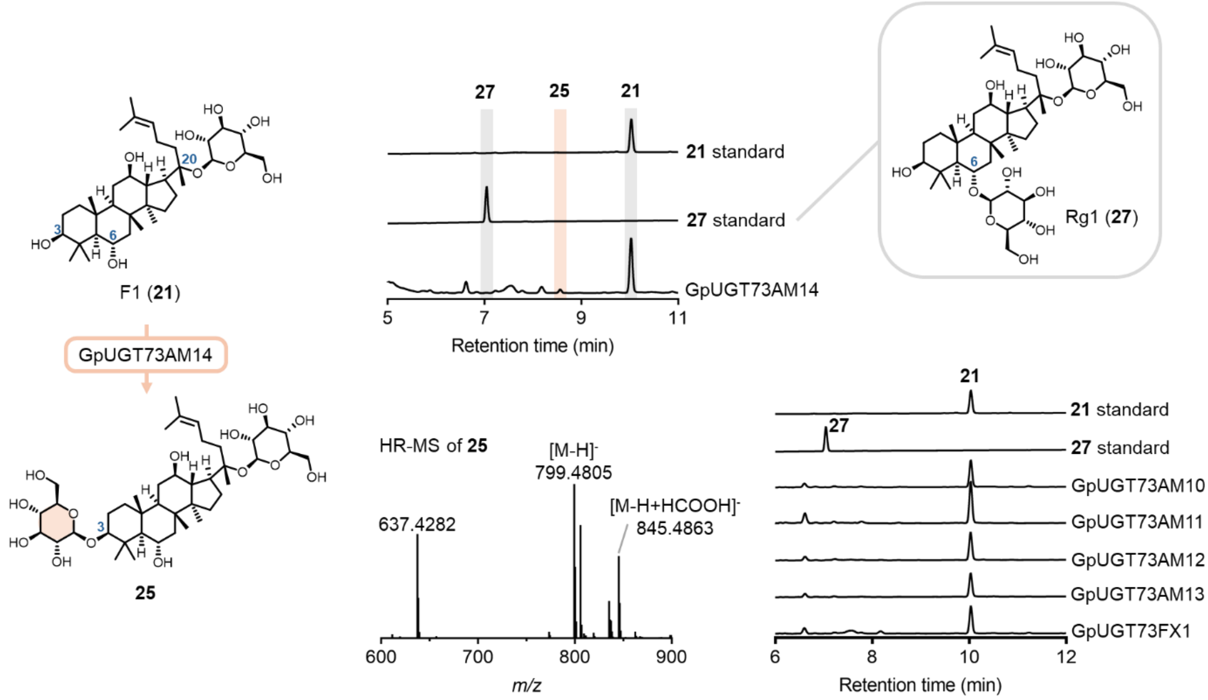

Figure S25. GpUGT73AM14 as ginsenoside F1 3-*O*-glycosyltransferase. The versatile GpUGT73AM14 possesses ginsenoside F1 (**21**) 3-*O*-glycosyltransferase activity, whereas the remaining GpUGT73s were not active.

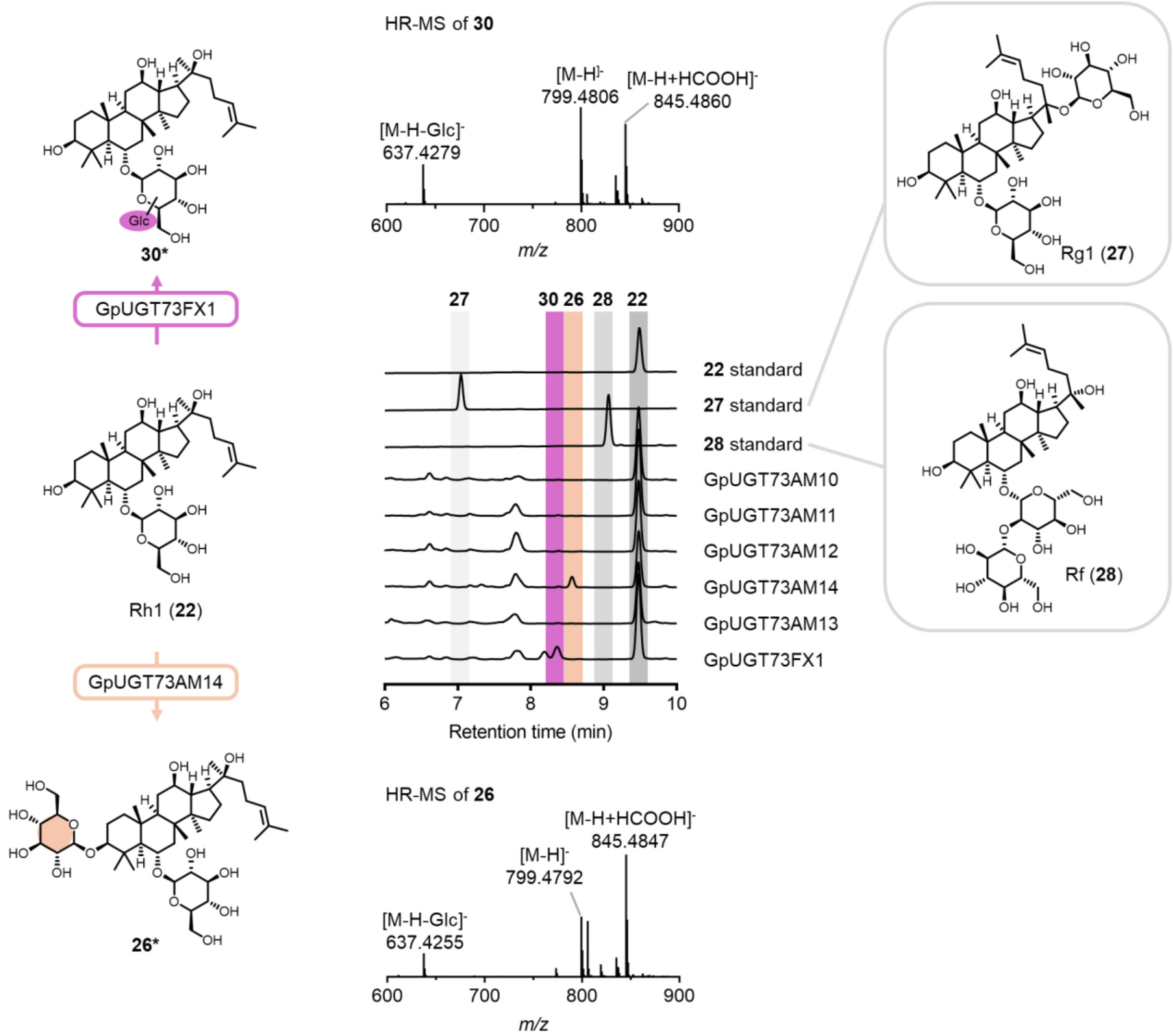

Figure S26. ***In vitro*** **biochemical characterization of GpUGT73FX1 and GpUGT73AM14 towards ginsenoside Rh1.** Unfortunately, neither product could be purified enough for NMR confirmation. The activity of GpUGT73AM14 as ginsenoside F1 3-*O*-glycosyltransferase suggested product **26** might be ginsenoside Rh1 3-*O*-glucoside, while product **30**, based on comparison with standards, was putatively identified as di-glucoside with an extended sugar chain at C-6 position.

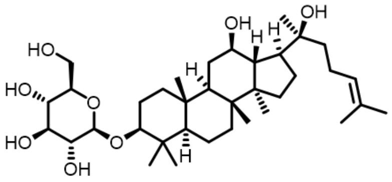

Rh2 (**29**)

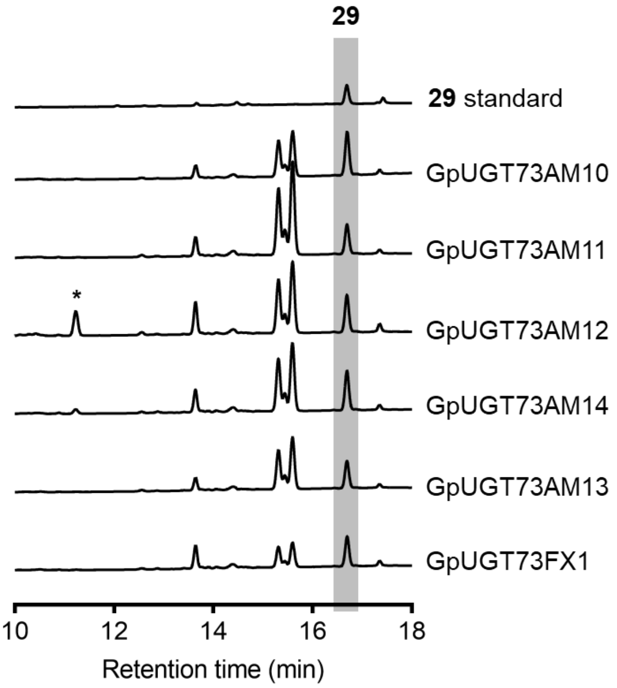

Figure S27. *In vitro* assay of GpUGT73AMs and GpUGT73FX1 toward ginsenoside Rh2. None of the UGT73s exhibit glycosylation activity against the ginsenoside Rh2 (**29**). The asterisk (*) indicates impurity.

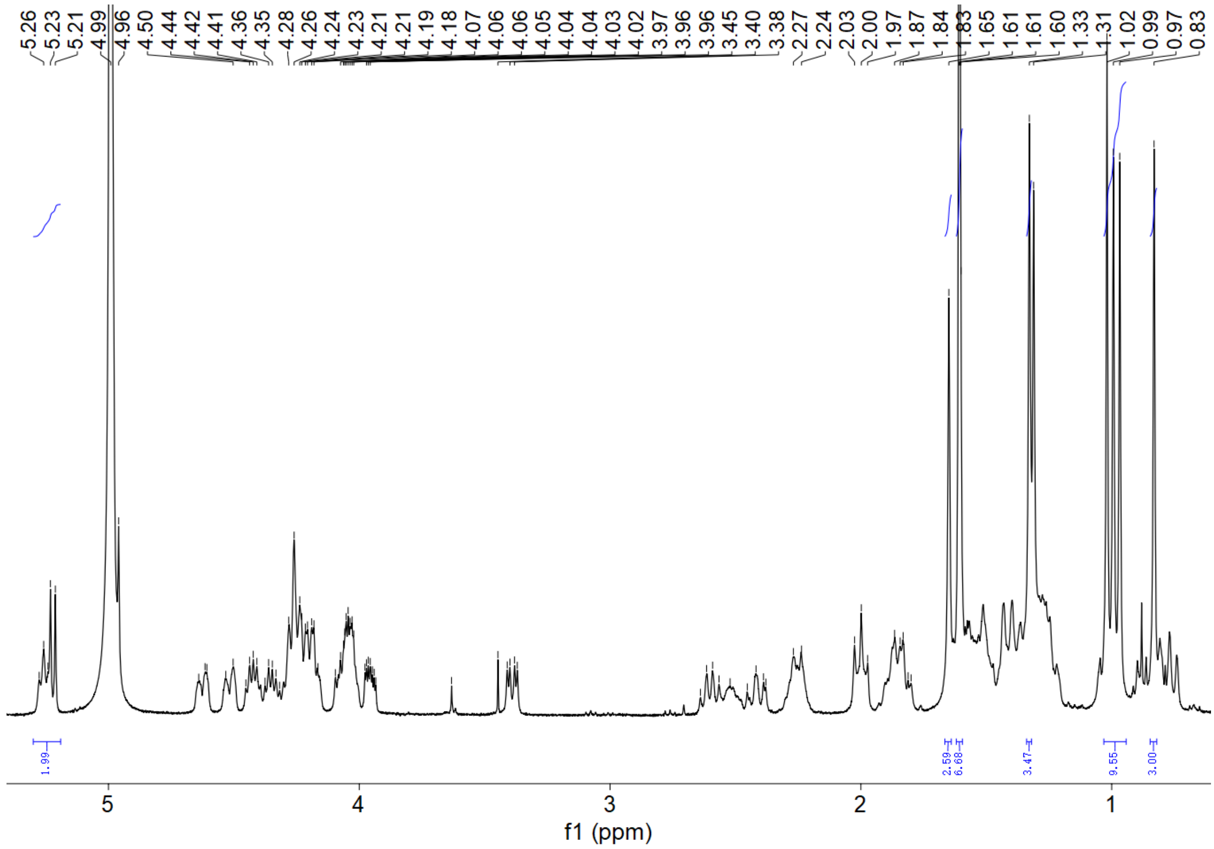

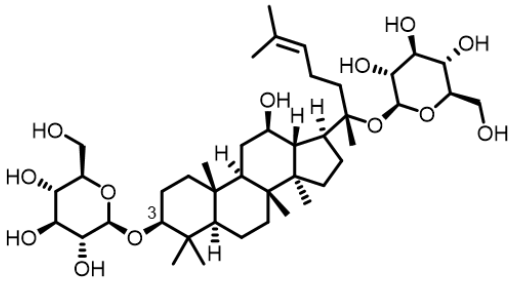

Figure S28. ^{1}H (400 MHz, pyridine-*d*5) NMR spectrum of ginsenoside F2 (24).

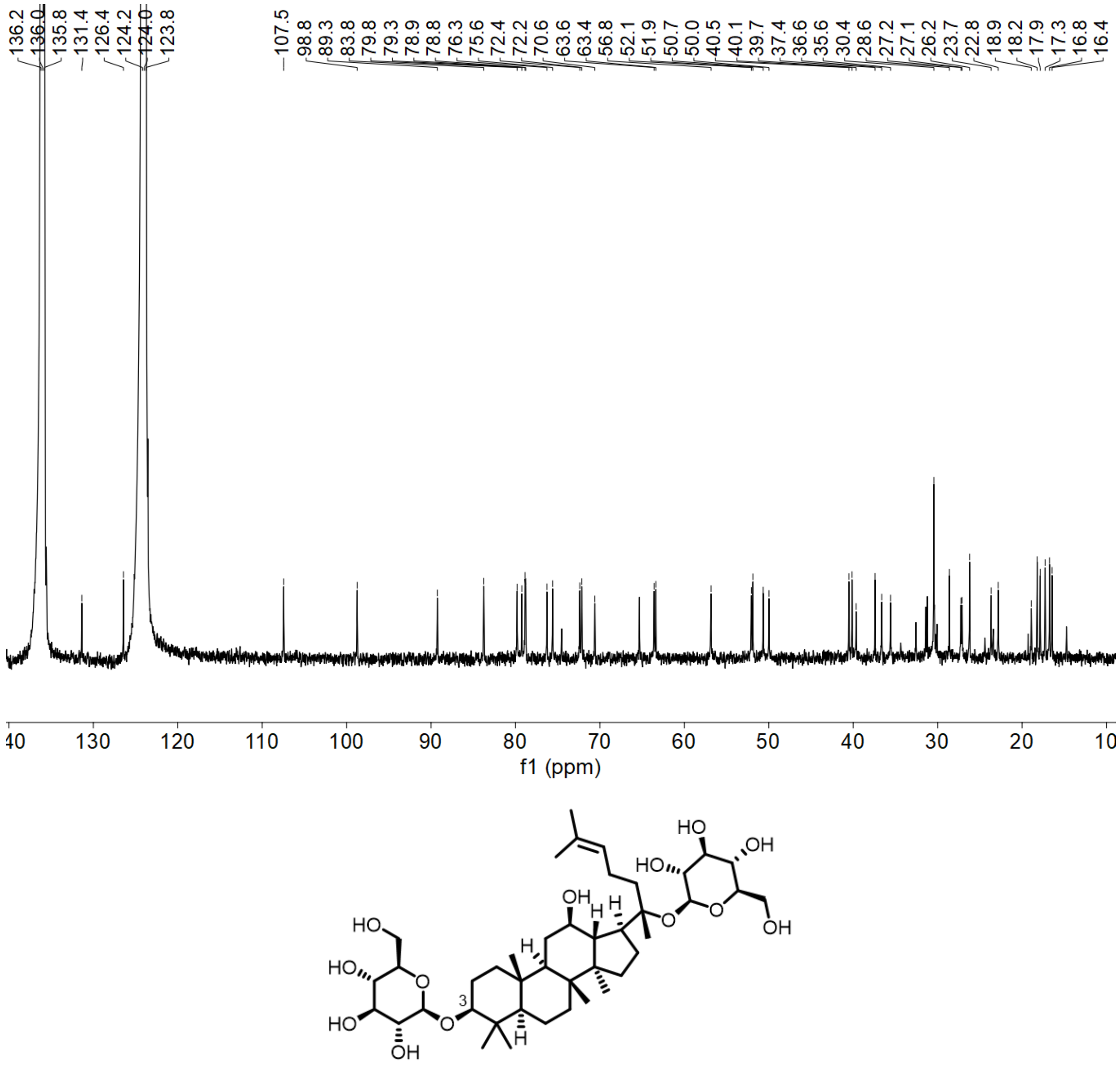

Figure S29. ^{13}C (125 MHz, pyridine-d_5) NMR spectrum of ginsenoside F2 (24).

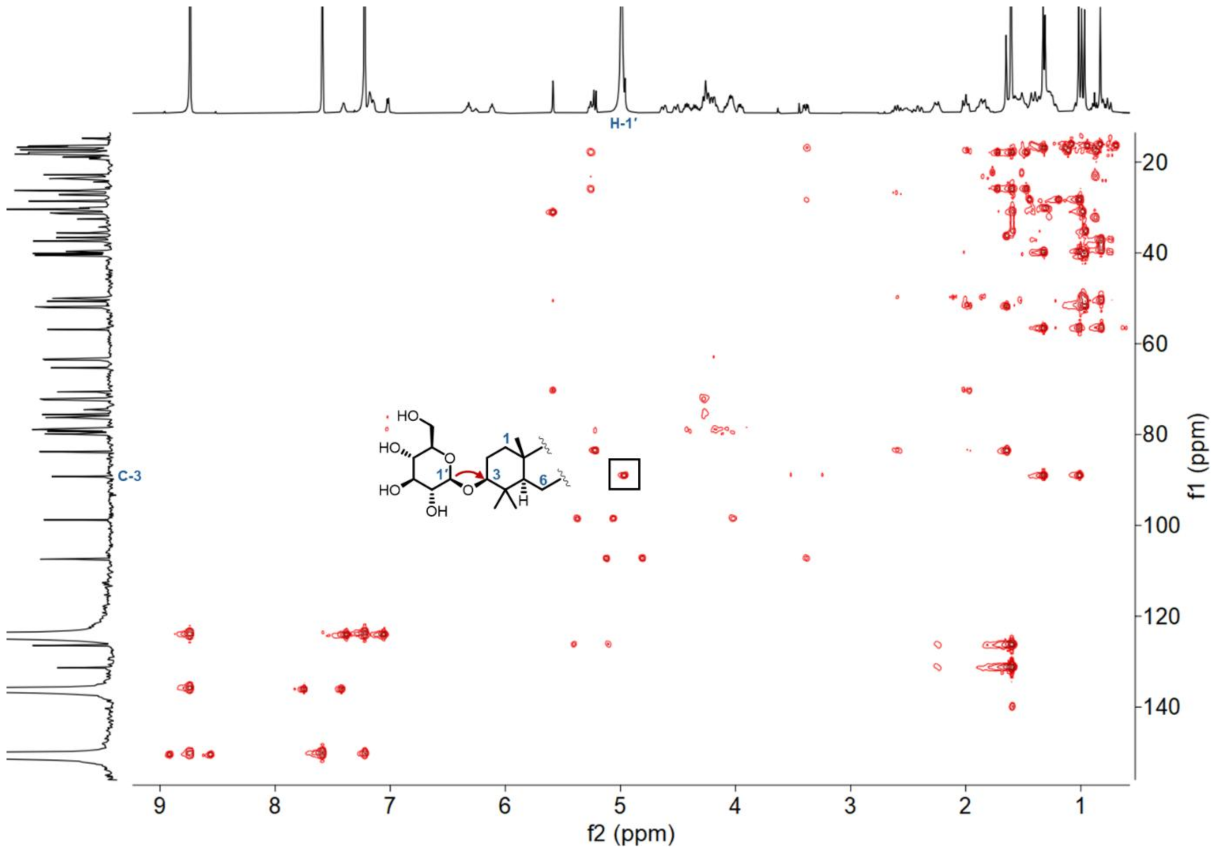

Figure S30. HMBC spectrum of ginsenoside F2 (24).

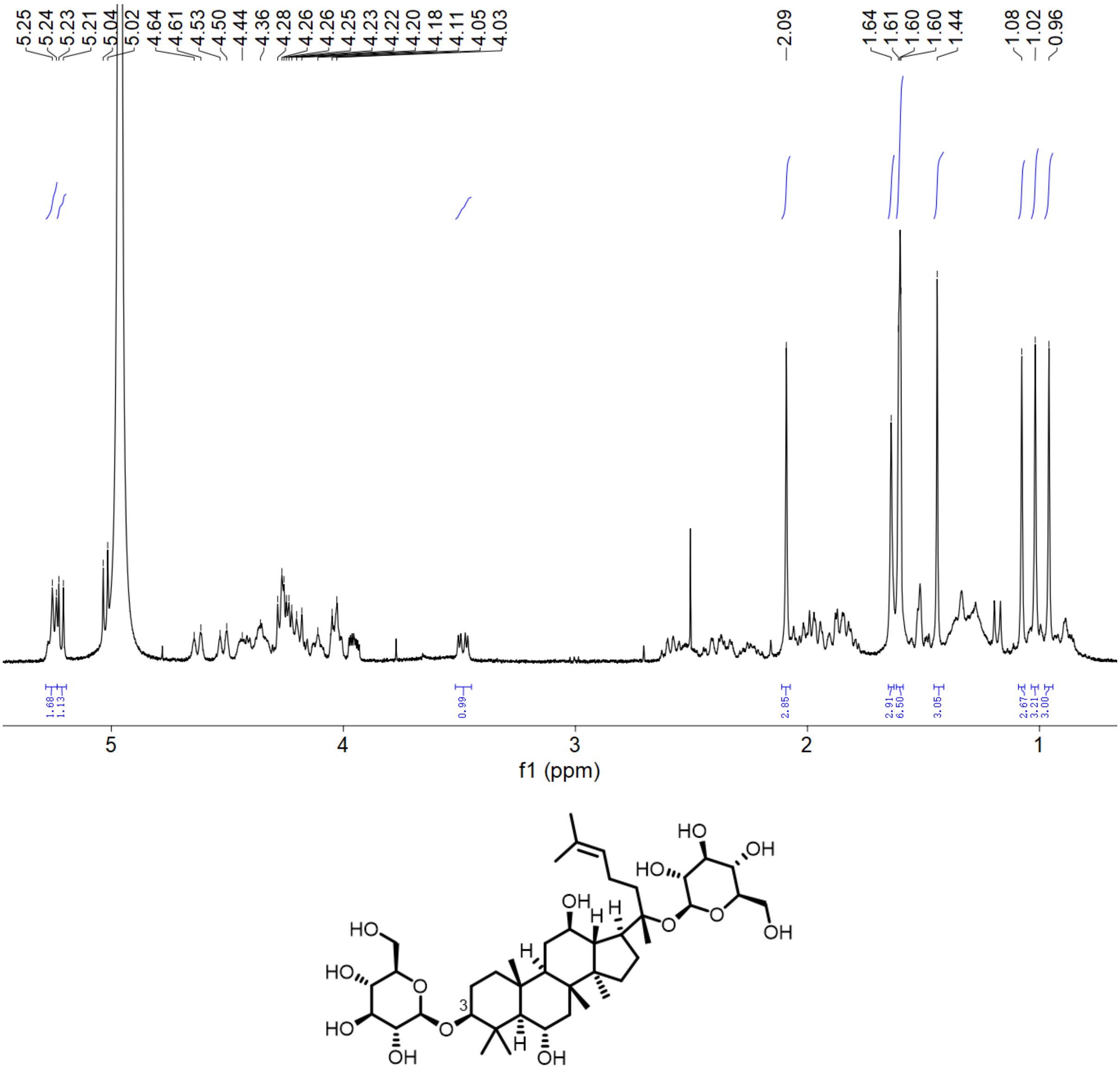

Figure S31. **^{1}H (400 MHz, pyridine-*d*5) NMR spectrum of ginsenoside F1 3-*O*-glucoside (25).**

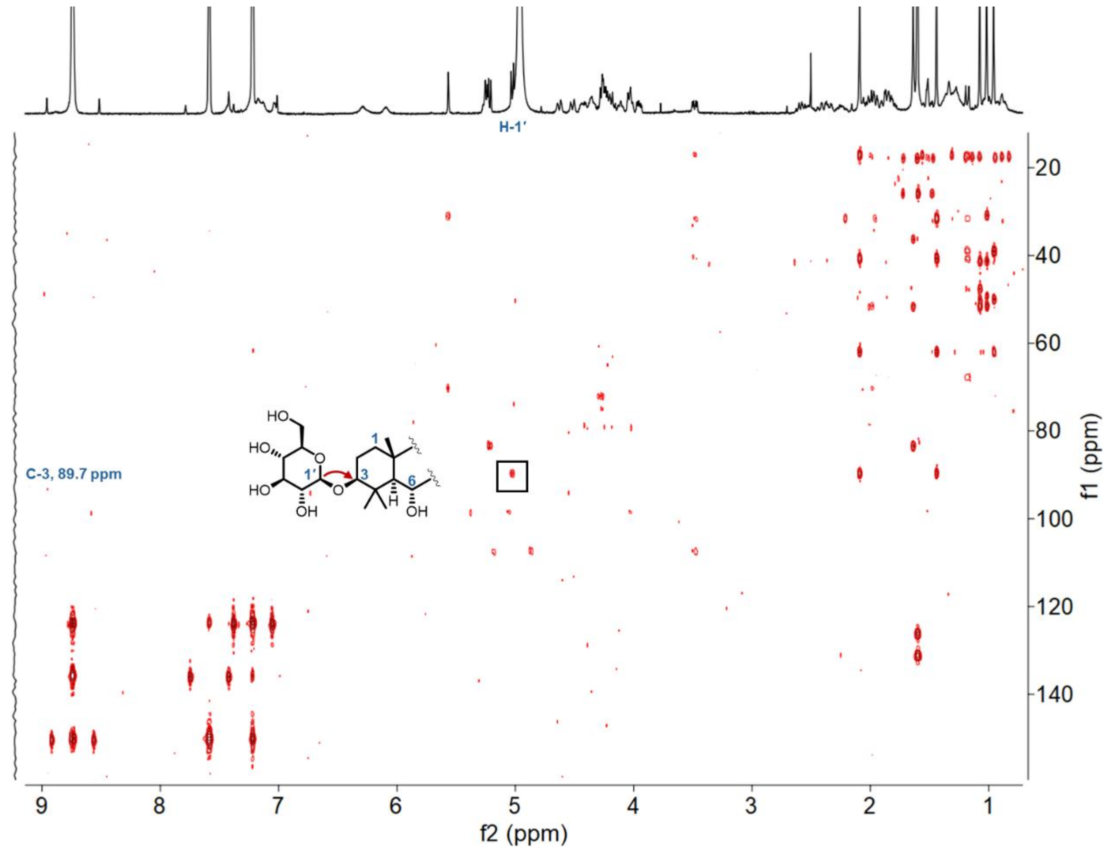

Figure S32. HMBC spectrum of ginsenoside F1 3-*O*-glucoside (25).

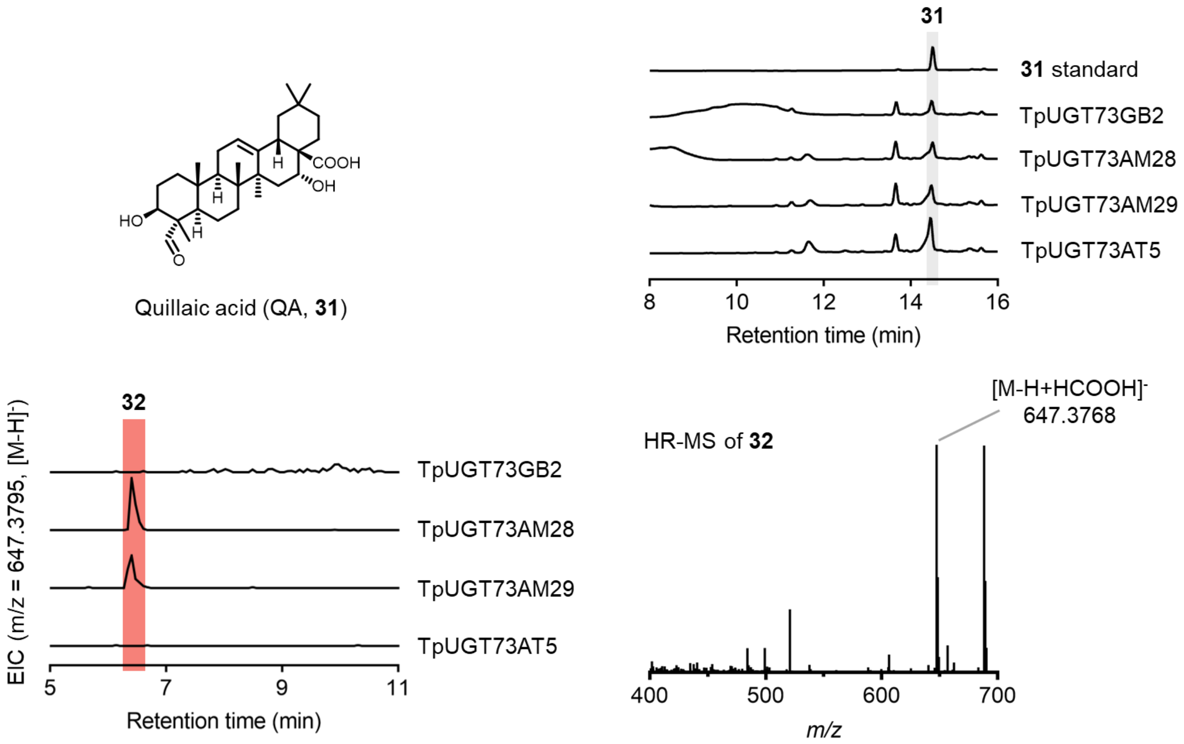

Figure S33. *In vitro* biochemical assay of TpUGT73s. TpUGT73AM28 and TpUGT73AM29 exhibited slight glycosylation towards QA (**31**), which could only be detected by LC-MS.

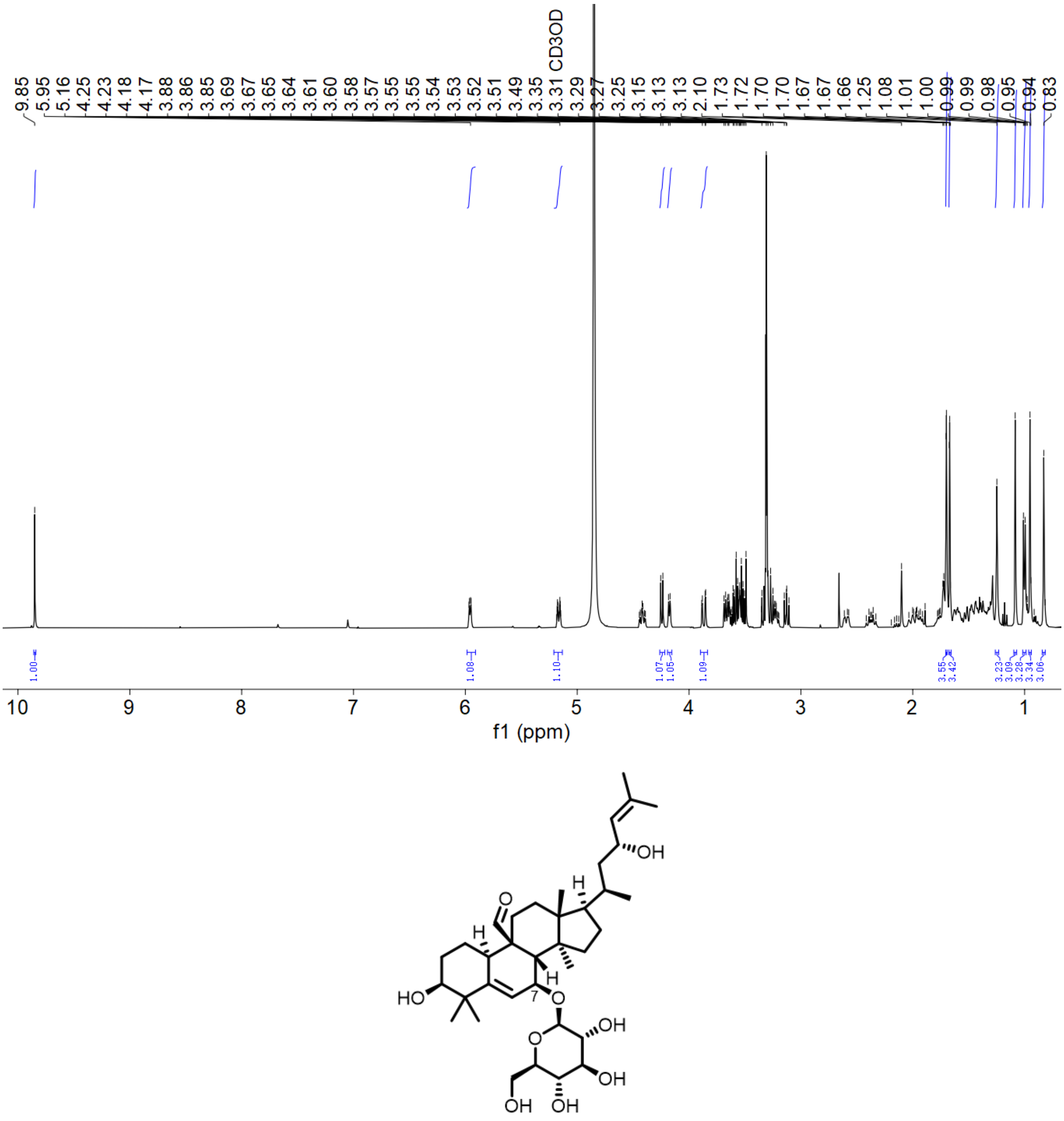

Figure S34. **^{1}H (400 MHz, methanol-d_4) NMR spectrum of momordicine IV (34).**

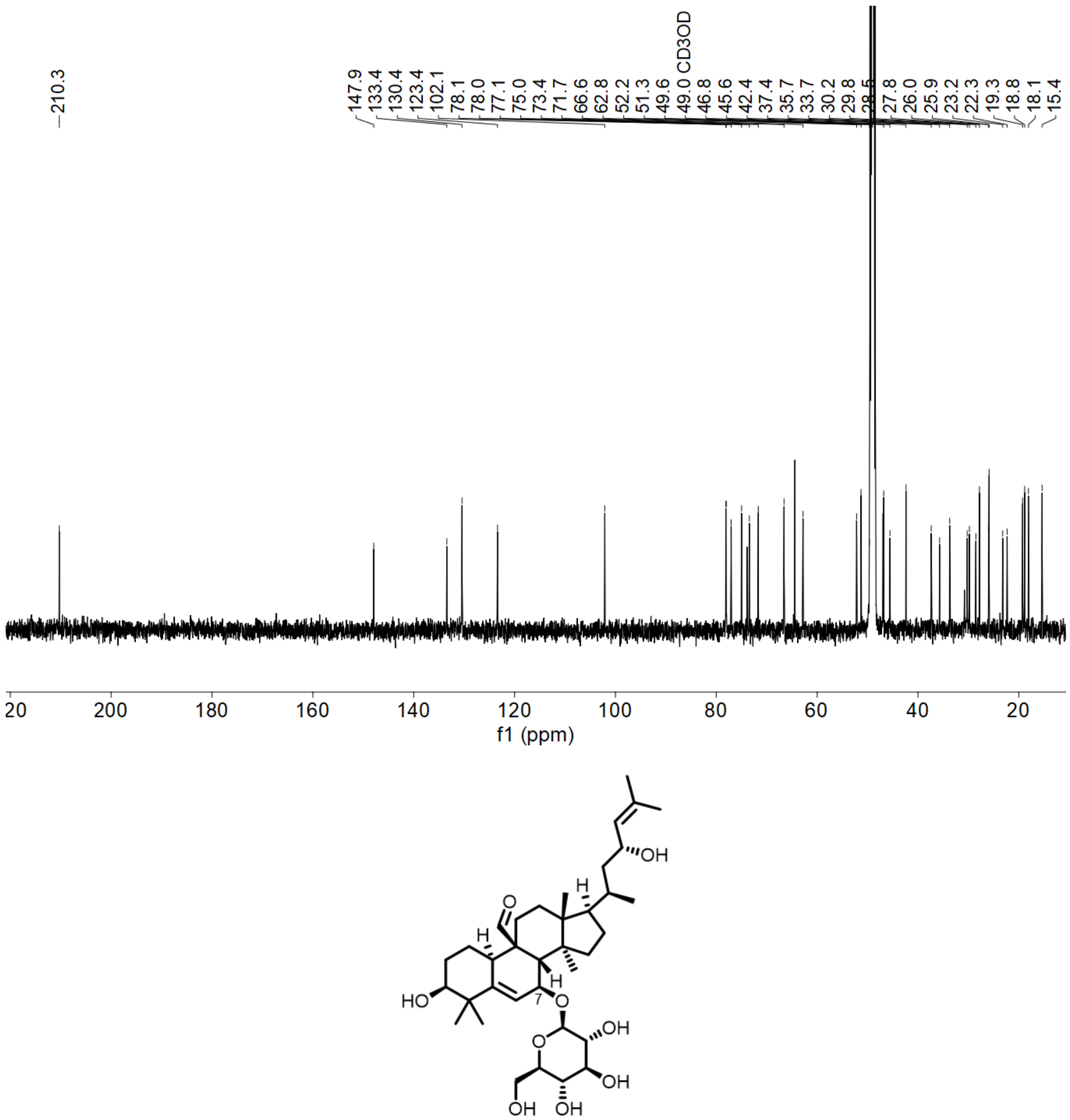

Figure S35. **^{13}C (125 MHz, methanol-*d*$_4$) NMR spectrum of momordicine IV (34).**

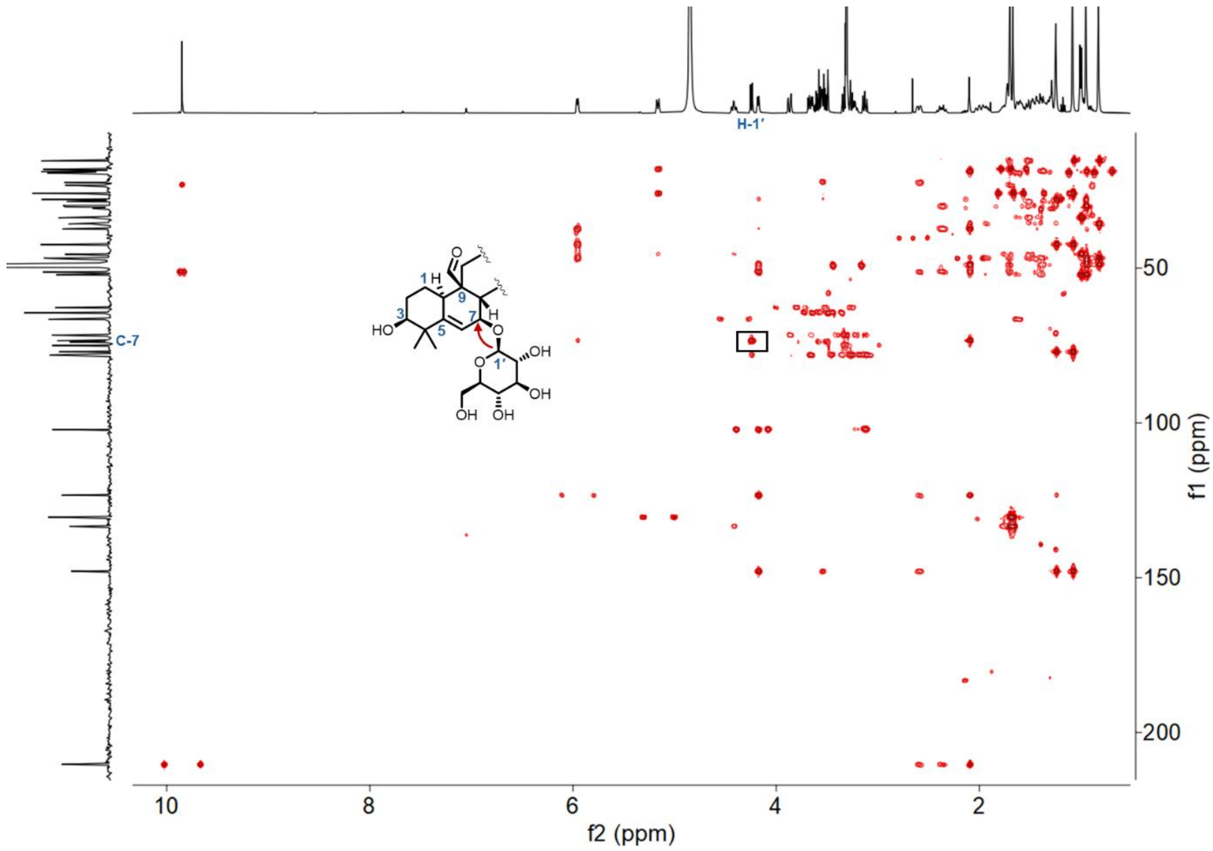

Figure S36. HMBC spectrum of momordicine IV (34).

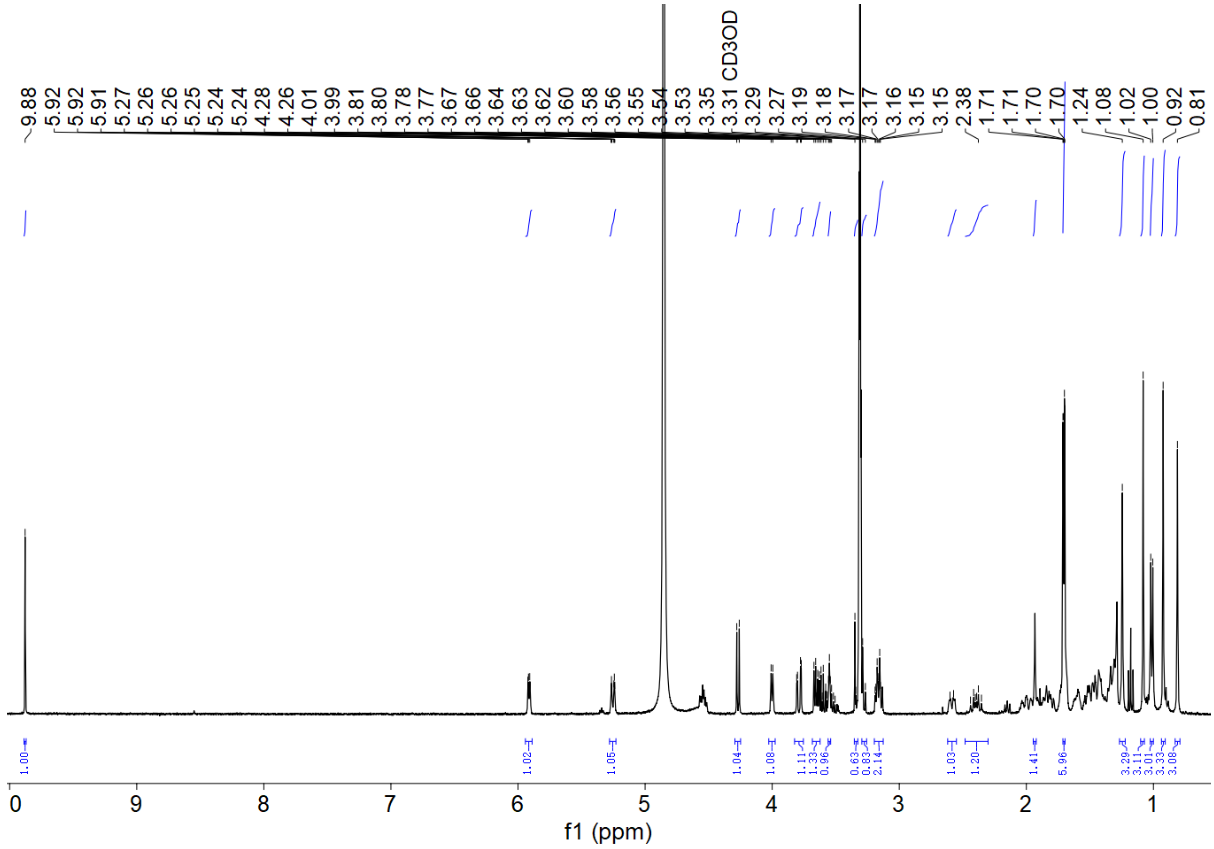

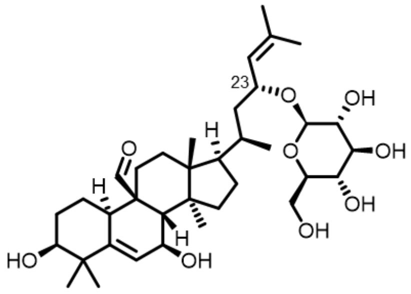

Figure S37. ^{1}H (400 MHz, methanol-*d*4) NMR spectrum of momordicine II (35).

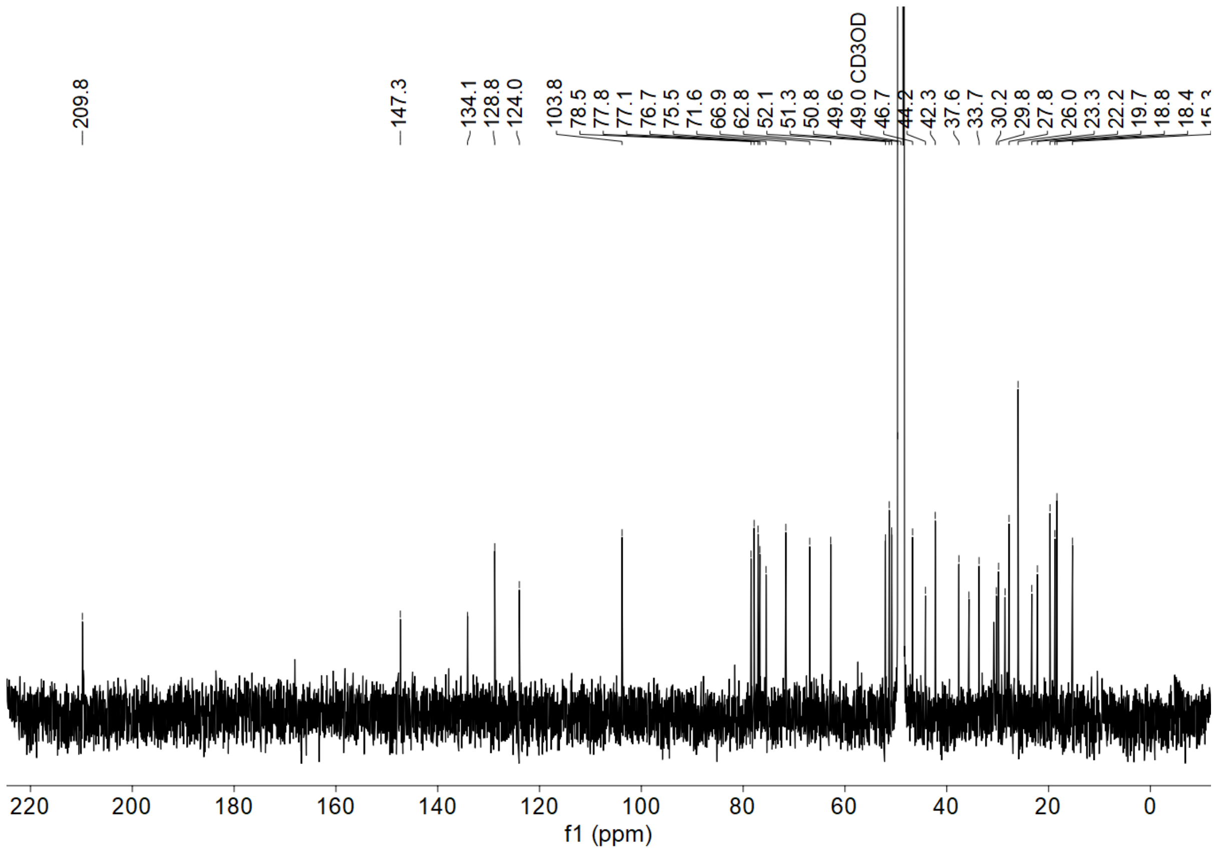

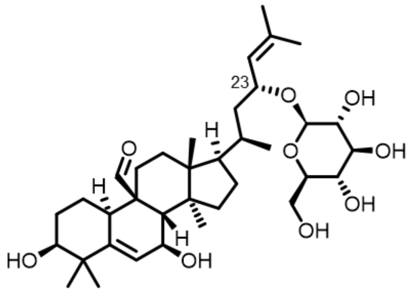

Figure S38. ^{13}**C (125 MHz, methanol-*d*$_4$) NMR spectrum of momordicine II (35).**

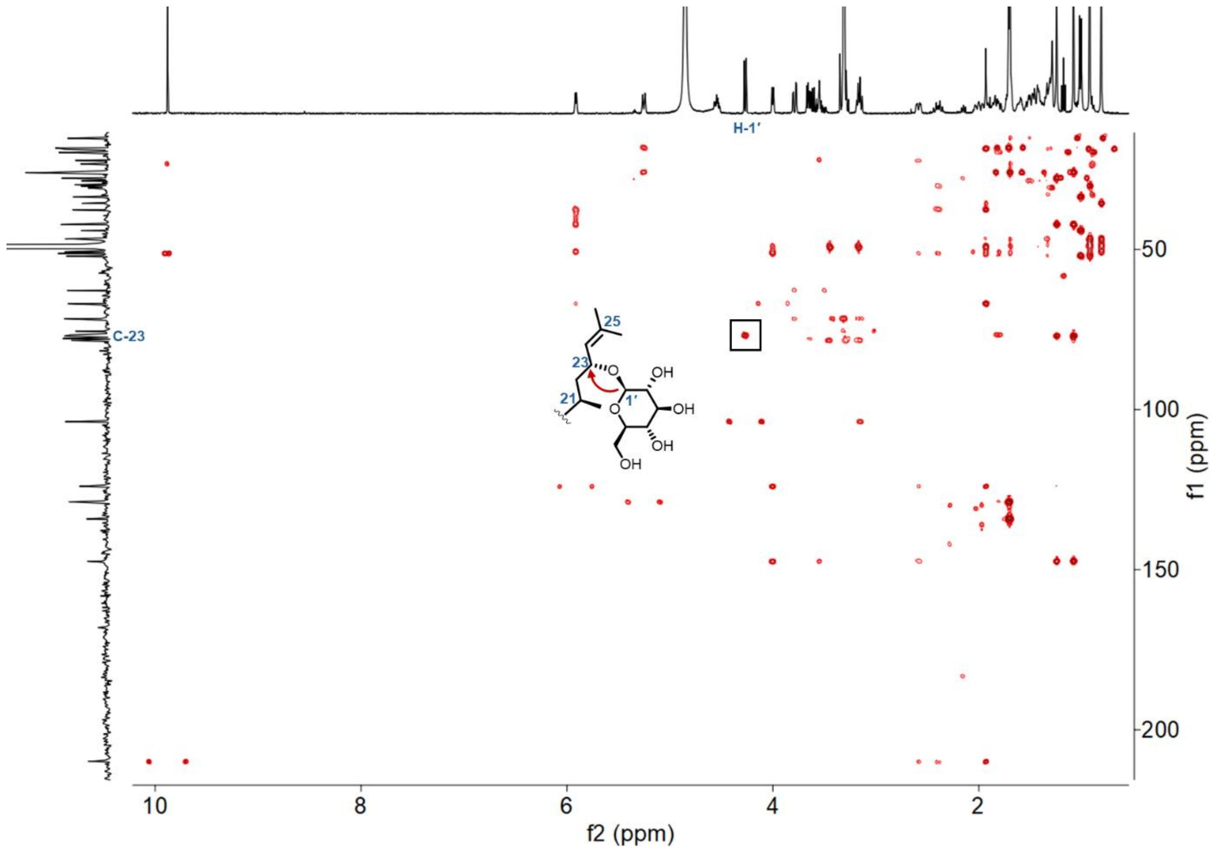

Figure S39. HMBC spectrum of momordicine II (35).

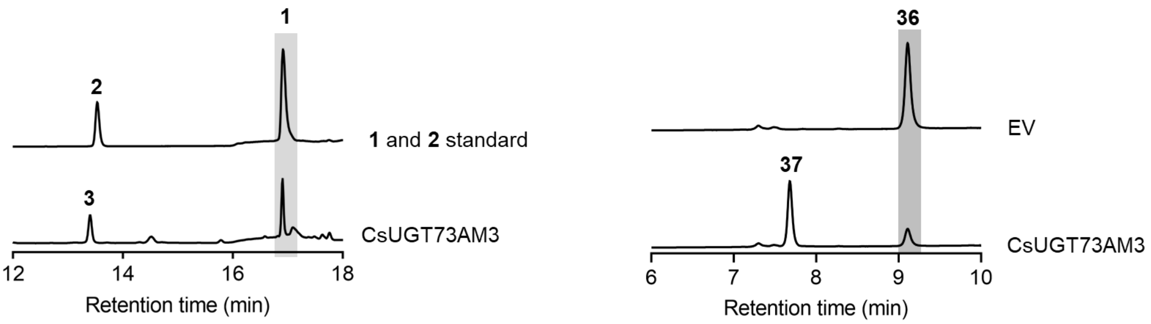

Figure S40. ***In vitro*** **assay of CsUGT73AM3 with mogrol (1) and cucurbitacin IIb (36).**

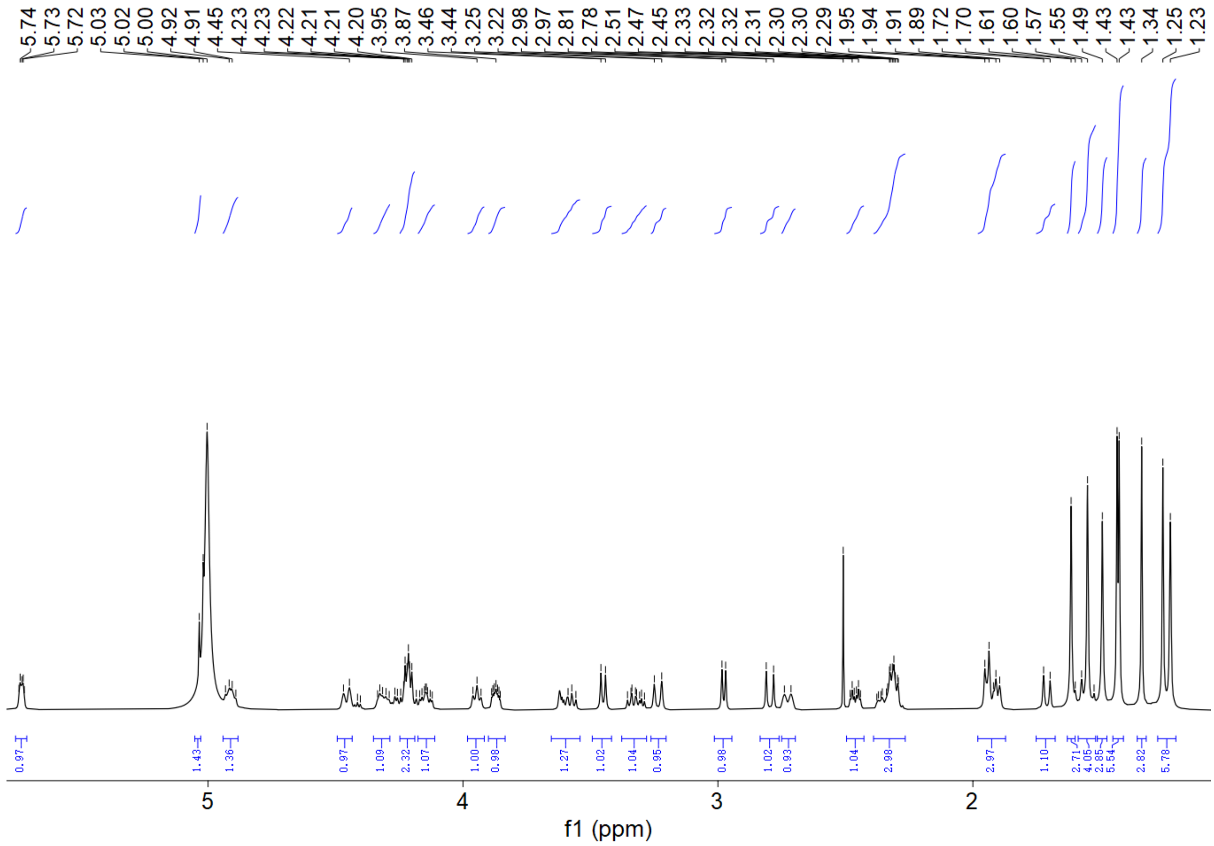

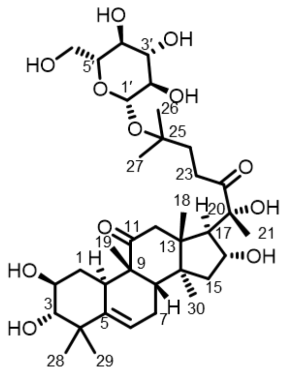

Figure S41. ^{1}H (400 MHz, pyridine-*d*$_5$) NMR spectrum of cucurbitacin IIb 25-*O*-glucoside (37).

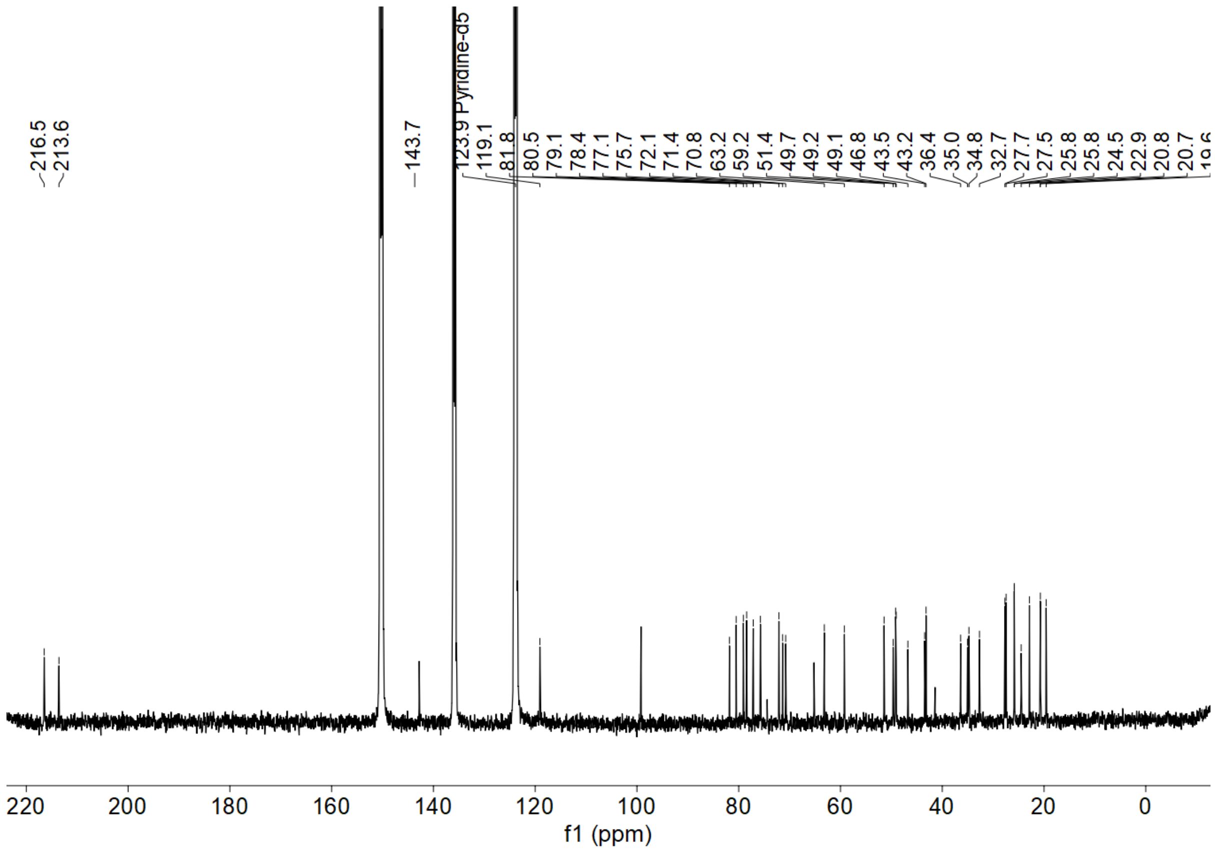

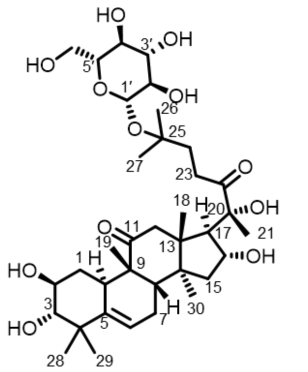

Figure S42. ^{13}C (125 MHz, pyridine-d_5) NMR spectrum of cucurbitacin IIb 25-*O*-glucoside (37). δ_C (ppm): 35.0 (C-1); 71.4 (C-2); 81.8 (C-3); 43.5 (C-4); 143.7 (C-5); 119.1 (C-6); 22.9 (C-7); 43.2 (C-8); 49.1 (C-9); 34.8 (C-10); 213.6 (C-11); 49.2 (C-12); 49.7 (C-13); 51.4 (C-14); 46.8 (C-15); 70.8 (C-16); 59.2 (C-17); 20.7 (C-18); 20.8 (C-19); 80.5 (C-20); 24.5 (C-21); 216.5 (C-22); 32.7 (C-23); 36.4 (C-24); 77.1 (C-25); 27.7 (C-26); 27.5 (C-27); 19.6 (C-28); 25.9 (C-29); 25.8 (C-30); 99.6 (C-1′); 75.7 (C-2′); 79.1 (C-3′); 72.1 (C-4′); 78.4 (C-5′); 63.2 (C-6′).

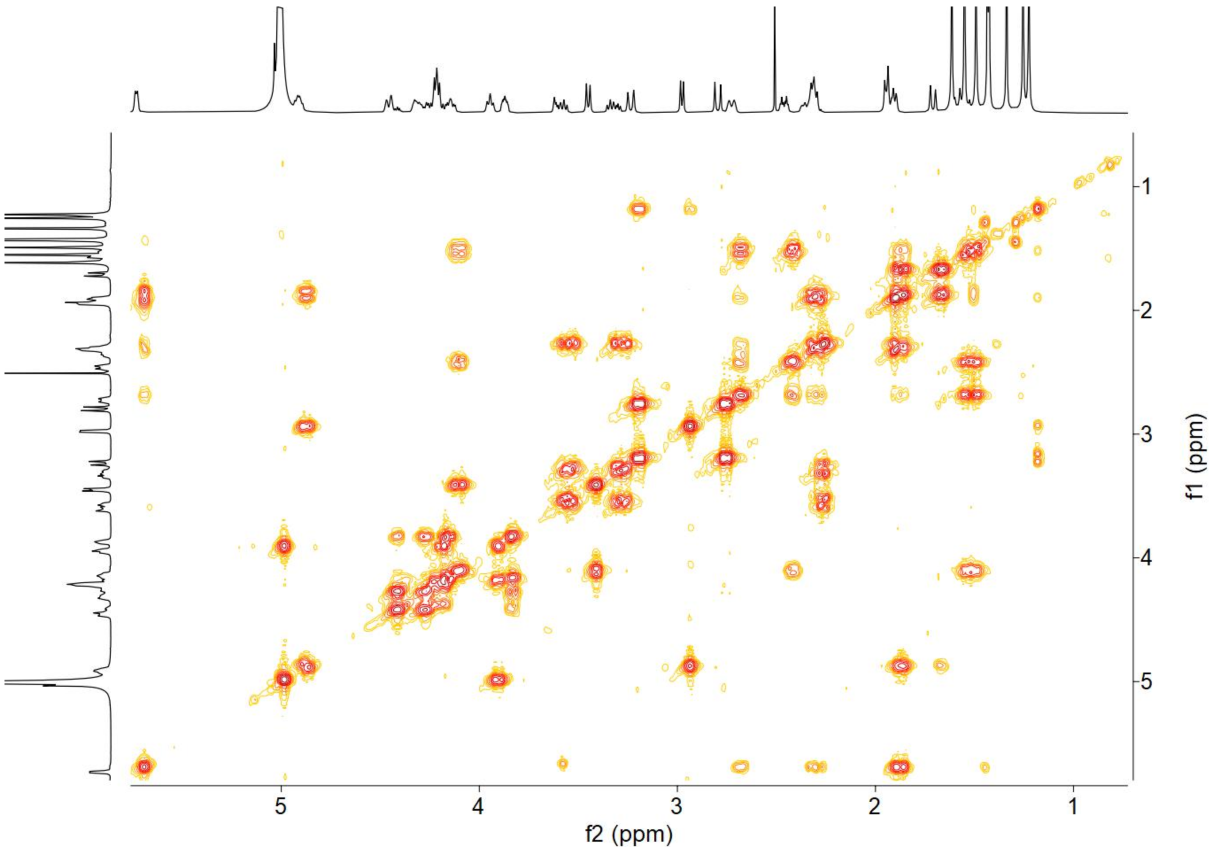

Figure S43. H-H COSY spectrum of cucurbitacin IIb 25-*O*-glucoside (37).

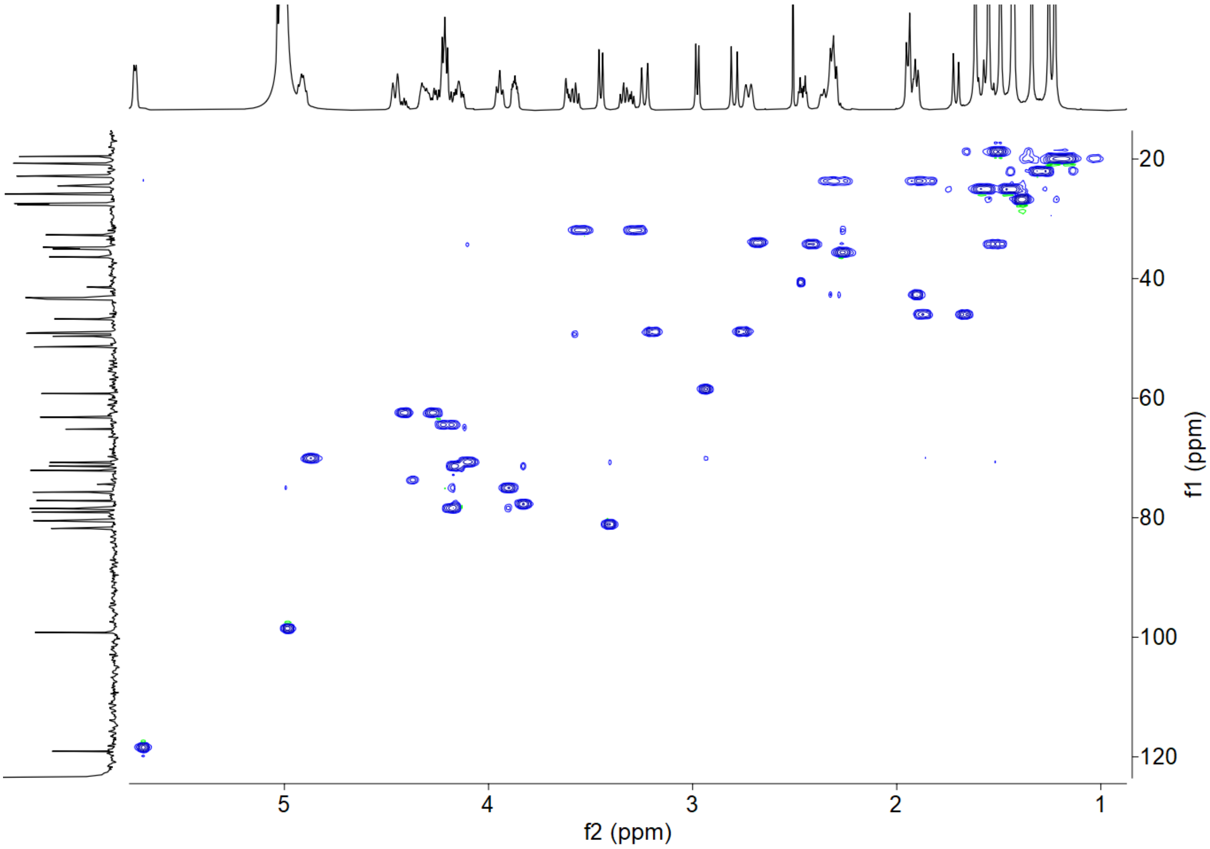

Figure S44. HSQC spectrum of cucurbitacin IIb 25-*O*-glucoside (37).

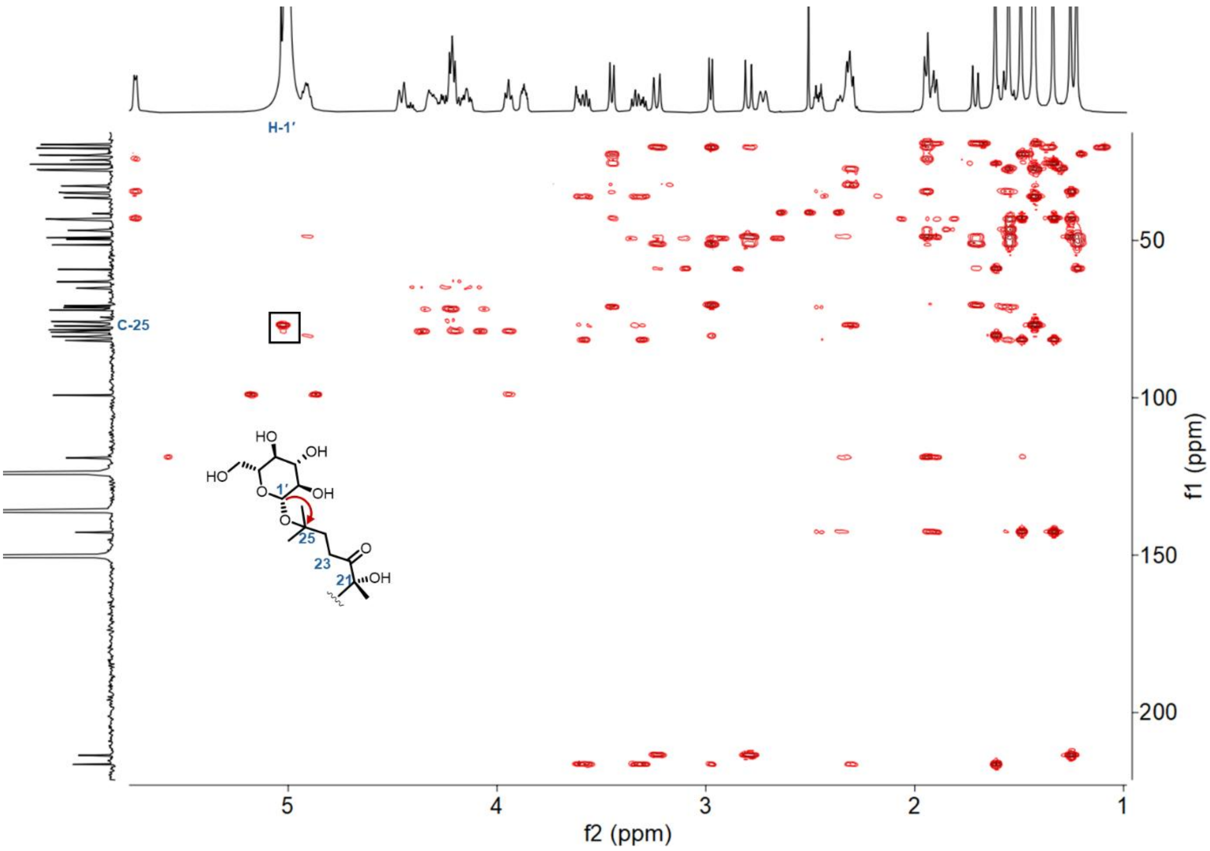

Figure S45. HMBC spectrum of cucurbitacin IIb 25-*O*-glucoside (37).

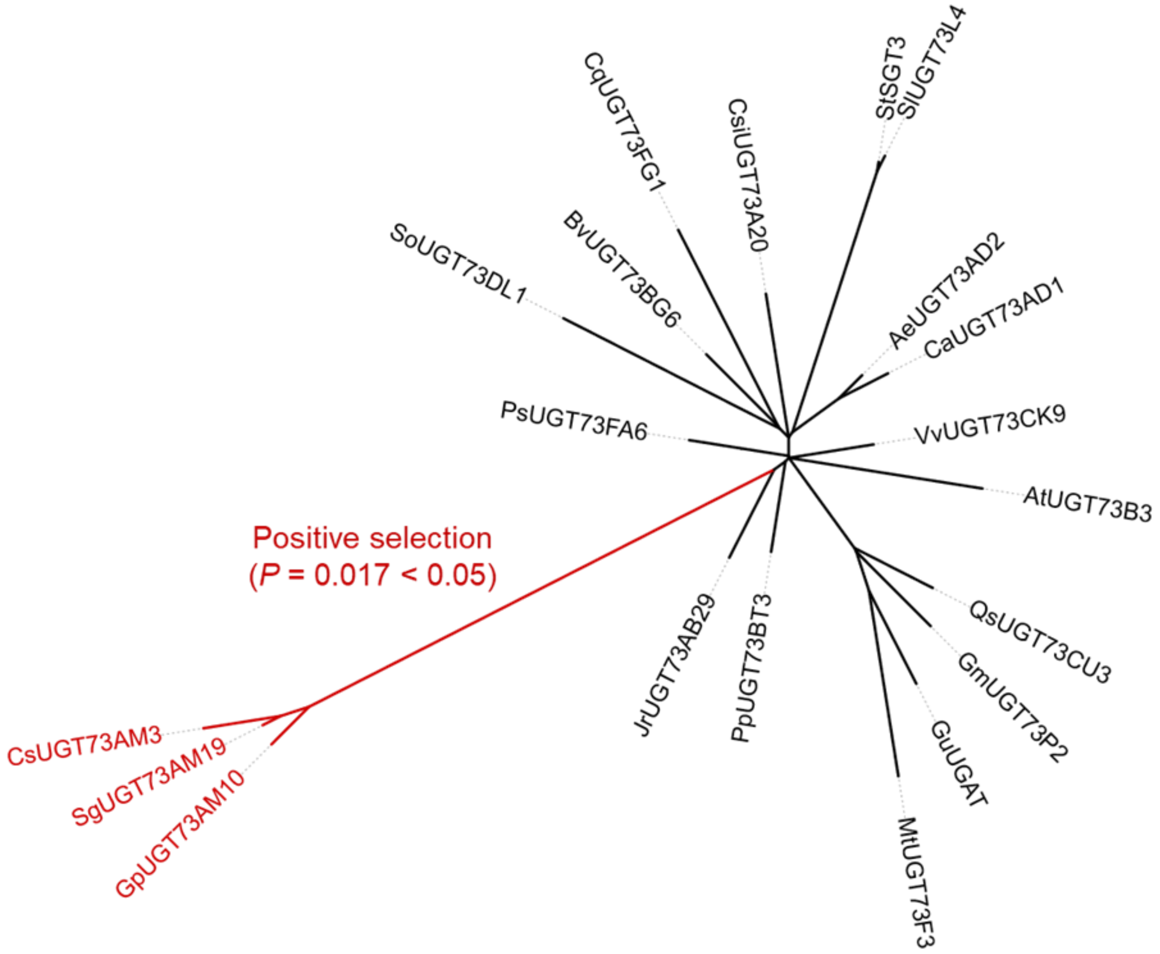

Figure S46. Maximum likelihood tree of functional characterized UGT73. Positive selection of Cucurbitaceae UGT73AMs were detected (p = 0.017). Leaf labels correspond to the following NCBI accession numbers: StSGT3 (ABB84472), SlUGT73L4 (ADQ37966), CsiUGT73A20 (ALO19886), GmUGT73P2 (BAI99584), GuUGAT (ANJ03631), MtUGT73F3 (ACT34898), AtUGT73B3 (AAM47999), CqUGT73FG1 (XM_021858666.1), SoUGT73DL1 (OR426405), CaUGT73AD1 (ALD84259), AeUGT73AD2 (UMX47351) and QsUGT73CU3 (WEU75099). The other UGT73s amino acid sequences are listed in Table S10.

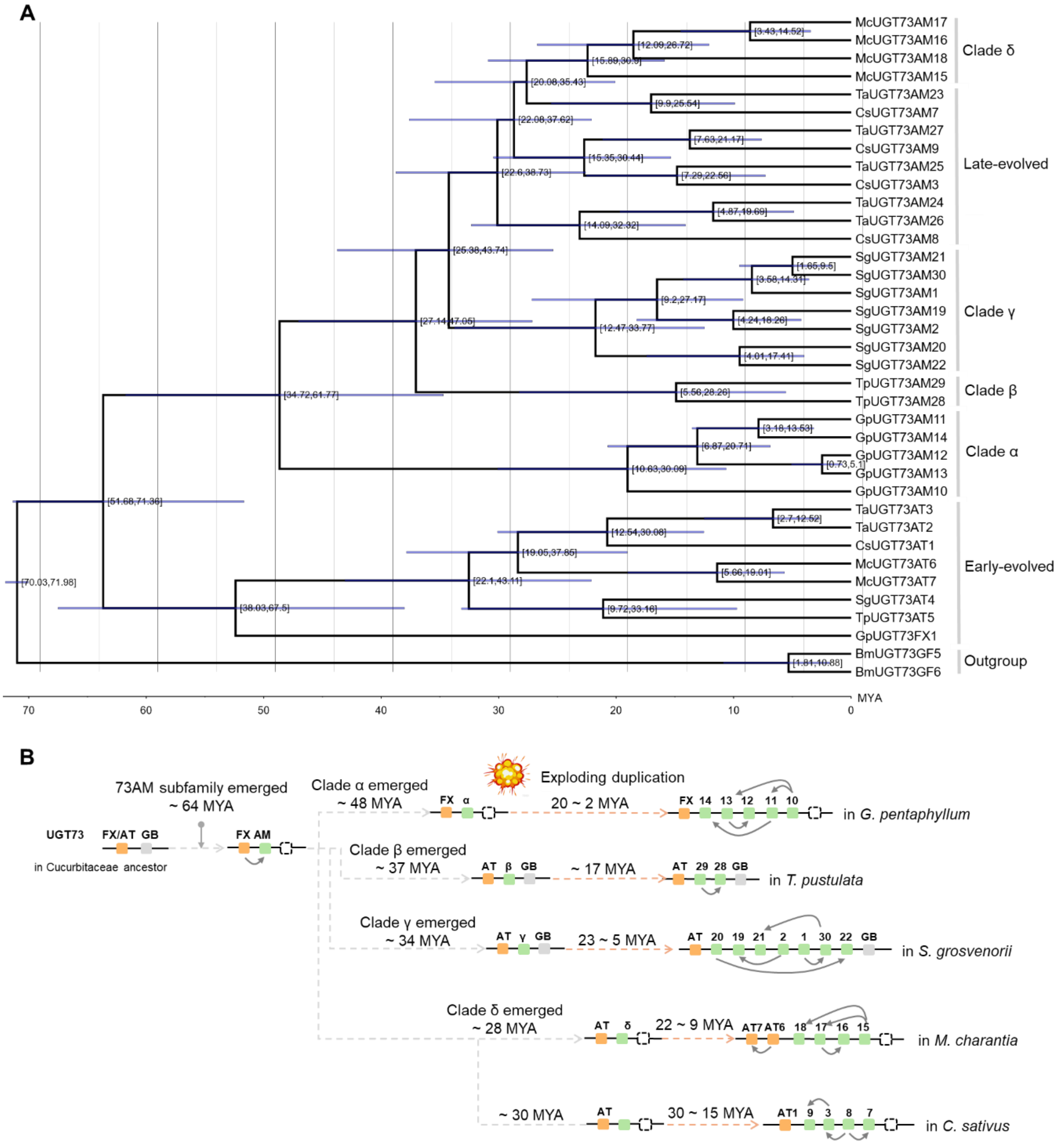

Figure S47. Origin and evolution of UGT73AMs in Cucurbitaceae. (**A**) Bayesian phylogeny of Cucurbitaceae UGT73AMs. The divergence time between BmUGT73GF and Cucurbitaceae UGT73AMs was calibrated to 70.5 million years ago (MYA), based on the Begoniaceae-Cucurbitaceae divergence time obtained from TimeTree (https://timetree.org). (**B**) Proposed evolutionary pathway of UGT73AMs in Cucurbitaceae plants.

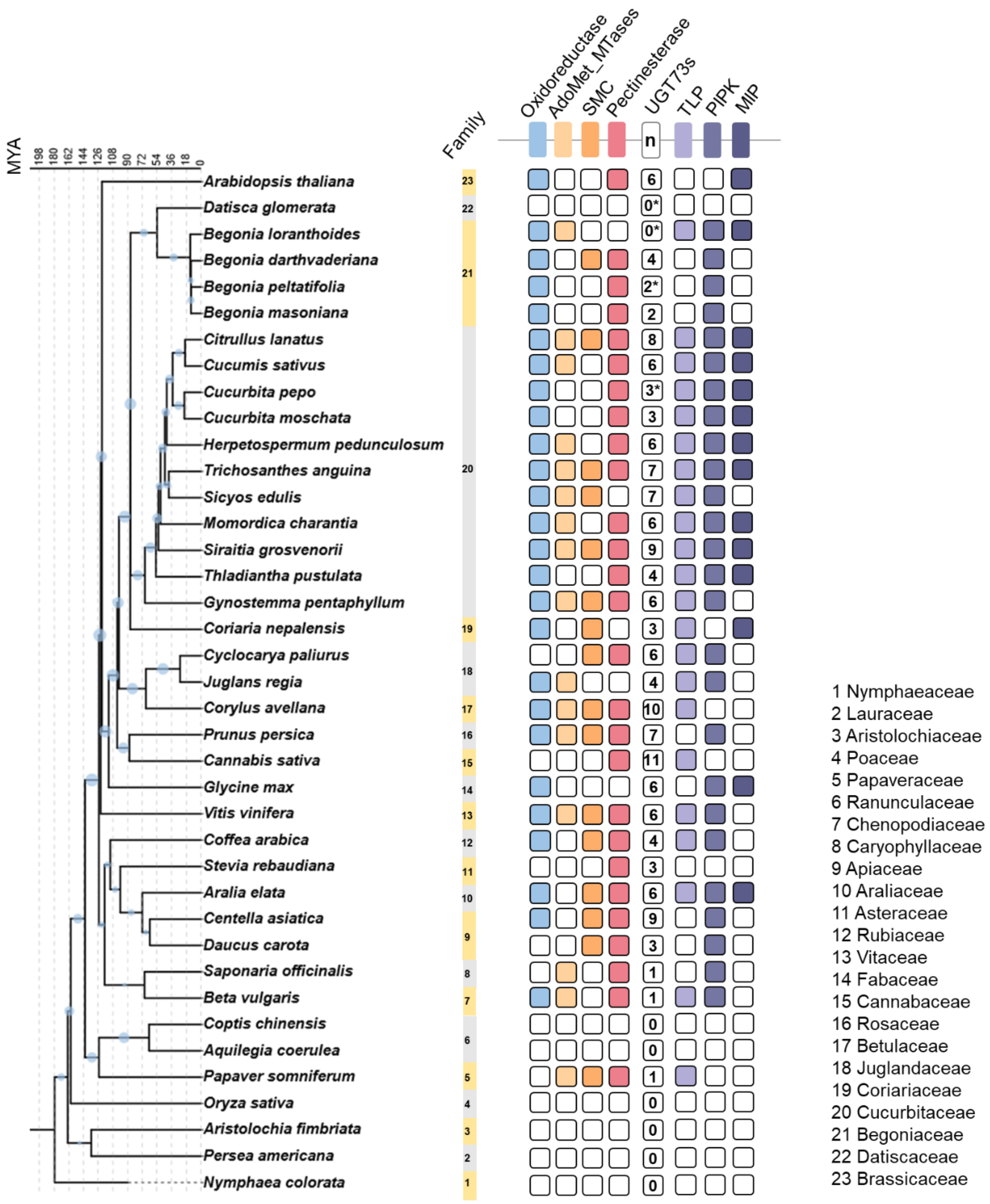

Figure S48. Distribution of 'UGT73 tandem array' across angiosperms species. The asterisk (*) indicated that the number of tandem duplicates of UGT73 is uncertain due to gaps in the genomic region or low quality of genome assemblies. Blue circles at nodes indicate bootstrap support values, with circle size proportional to the value ranging from 13 to 98. SMC, chromosome segregation protein; TLP, thaumatin-like proteins; PIPK, phosphatidylinositol phosphate kinase; MIP, major intrinsic protein.

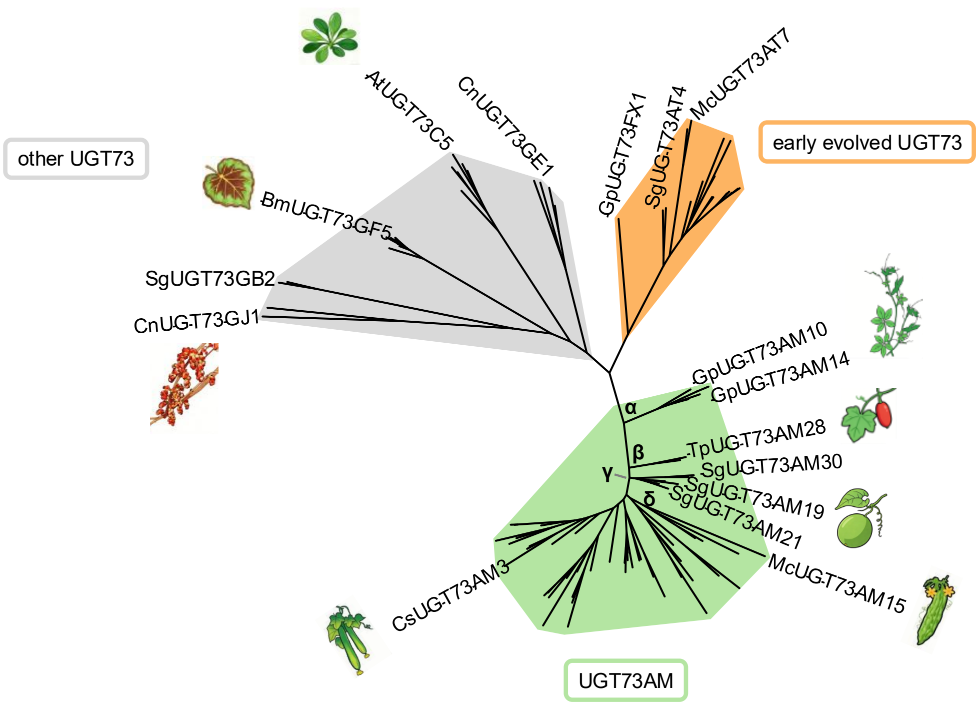

	Cucurbitales			Brassicales
	Begoniaceae	Coriariaceae	Cucurbitaceae	Brassicaceae
UGT73AM	×	×	○	×
UGT73AT	×	×	○	×
UGT73GB	×	×	○	×
UGT73FX	×	×	○	×
UGT73GE	×	○	×	×
UGT73GF	○	×	×	×
UGT73GJ	×	○	×	×
UGT73C	×	×	×	○

Figure S49. Phylogeny of UGT73s in Cucurbitales (AtUGT73Cs were used as outgroup). Generally, the UGT73 subfamilies listed here were found specific to each family.

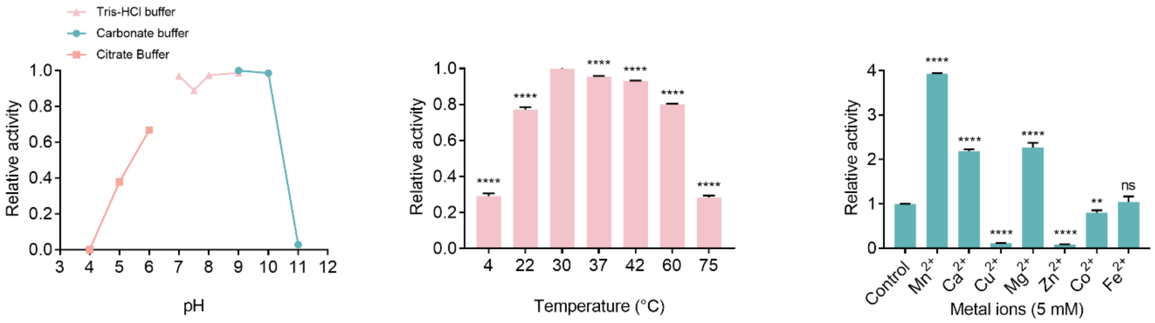

Figure S50. Optimization of SgUGT73AM30 activity. (**A**) The optimal pH for enzymatic reaction was 8~10. pH 8 was used in subsequent experiments. Tris-HCl buffer, 20 mM Tris base; Carbonate buffer, 50 mM Na_2HPO_4-NaH_2PO_4; Citrate buffer, 50 mM Citric Acid- Sodium Citrate. (**B**) The optimal temperature was determined as 30°C. (**C**) Influence of different divalent metal ions on the reactions. Copper and zinc ions significantly inhibited enzymatic activity; on the contrary, manganese, calcium and magnesium ions improved the activity. UDPG and mogrol (**1**) were used as the sugar donor and acceptor, respectively. Data are presented as the mean ± SD of three independent biological replicates (n=3). Statistical significance versus the control group (relative activity defined as 1.0) was determined by unpaired two-tailed Student's t-test (**, P < 0.01, ****, P < 0.0001; ns, not significant difference).

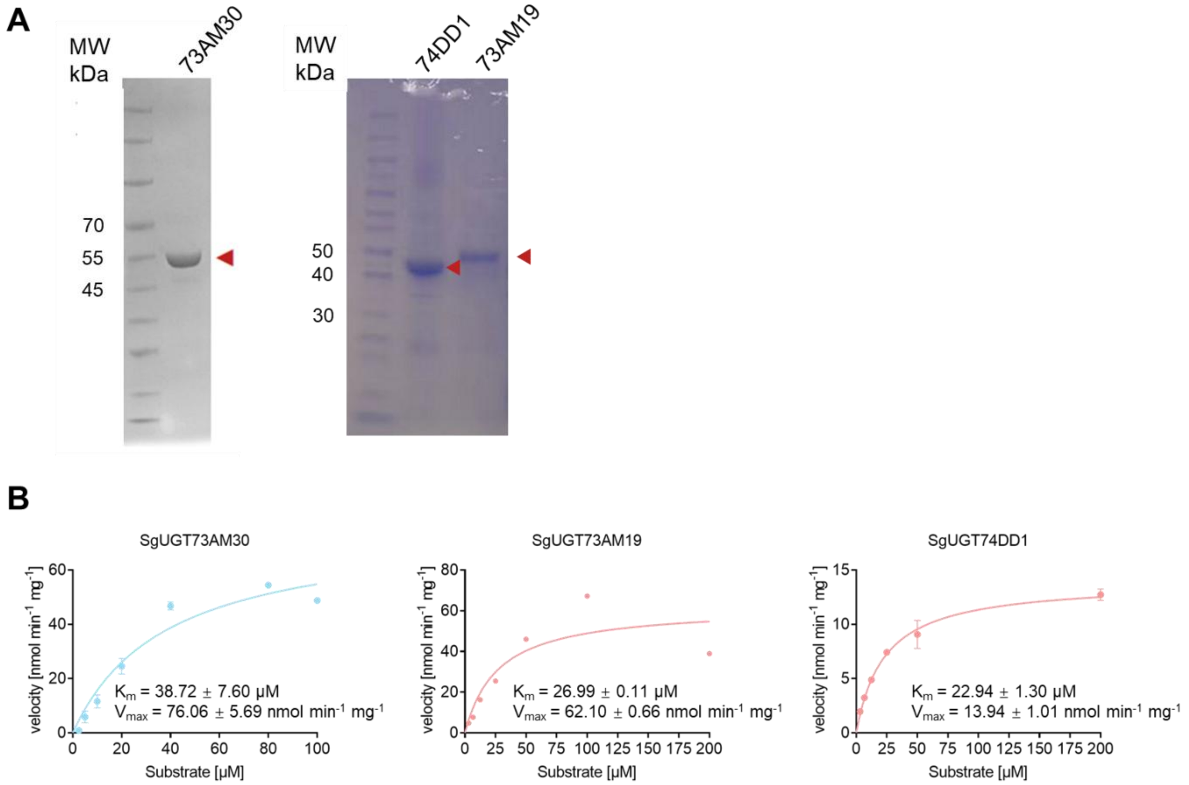

Figure S51. Enzyme expression and kinetics of SgUGT73AM30, SgUGT73AM19 and SgUGT74DD1. (**A**) SDS-PAGE analysis of the His_6-tagged proteins purified by nickel magnetic beads. **(B)** Determination of kinetic parameters for SgUGT73AM30, SgUGT73AM19 and SgUGT74DD1. The kinetic curves (initial velocity vs. substrate concentration) were fitted to the Michaelis-Menten equation. The derived parameters (Km and Vmax) are presented as mean ±SD from three independent experiments.

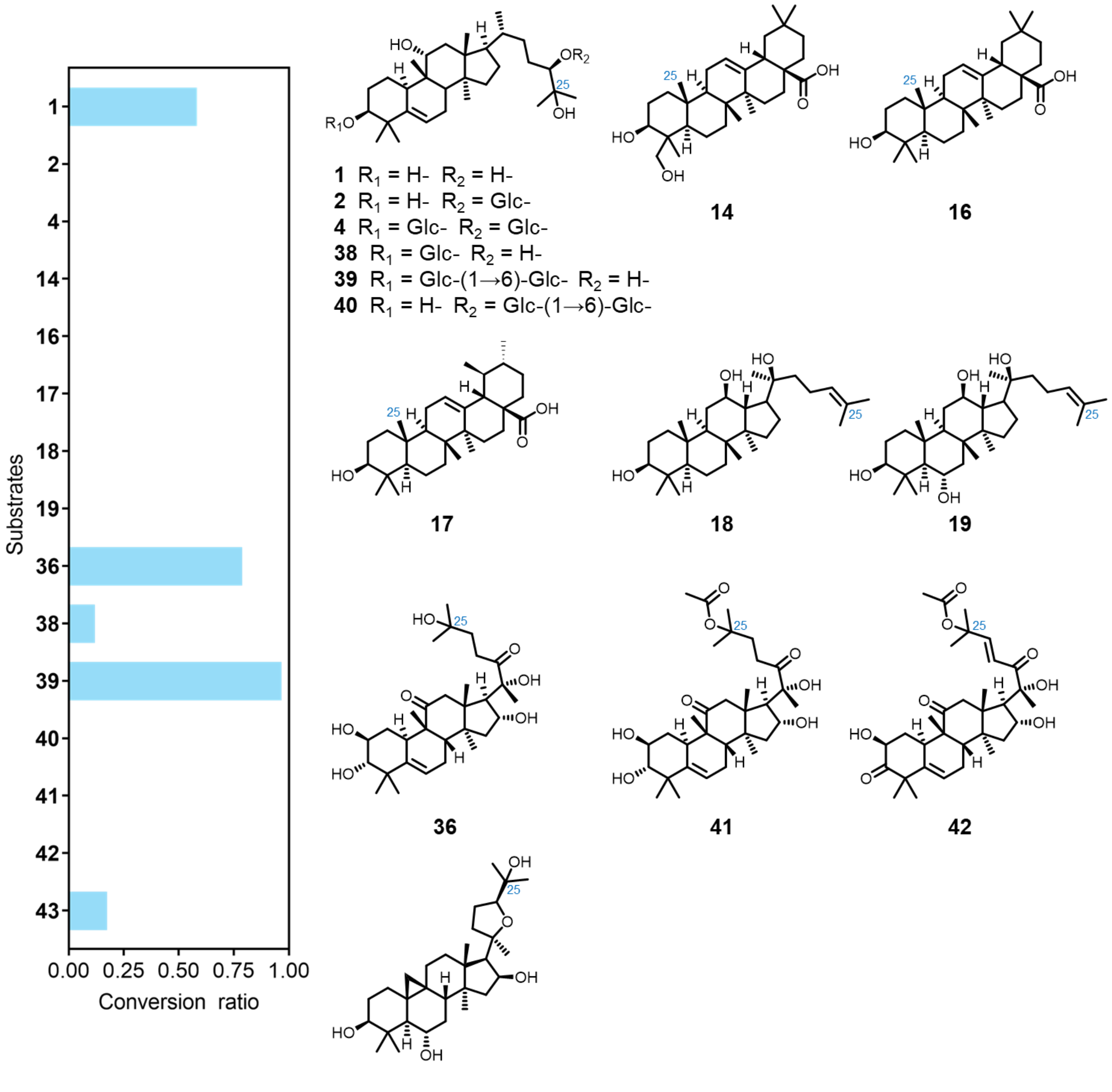

Figure S52. Screening the substrates of SgUGT73AM30. The substrate library consists of 15 compounds including mogrol (**1**), mogroside IA1 (**2**), mogroside IIE (**4**), hederagenin (**14**), oleanolic acid (**16**), ursolic acid (**17**), (20*S*)-protopanaxadiol (**18**), (20*S*)-protopanaxatriol (**19**), cucurbitacin IIb (**36**), mogroside IE1 (**38**), mogroside IIA2 (**39**), mogroside IIA1 (**40**), cucurbitacin IIa (**41**), cucurbitacin B (**42**), cycloastragenol (**43**).

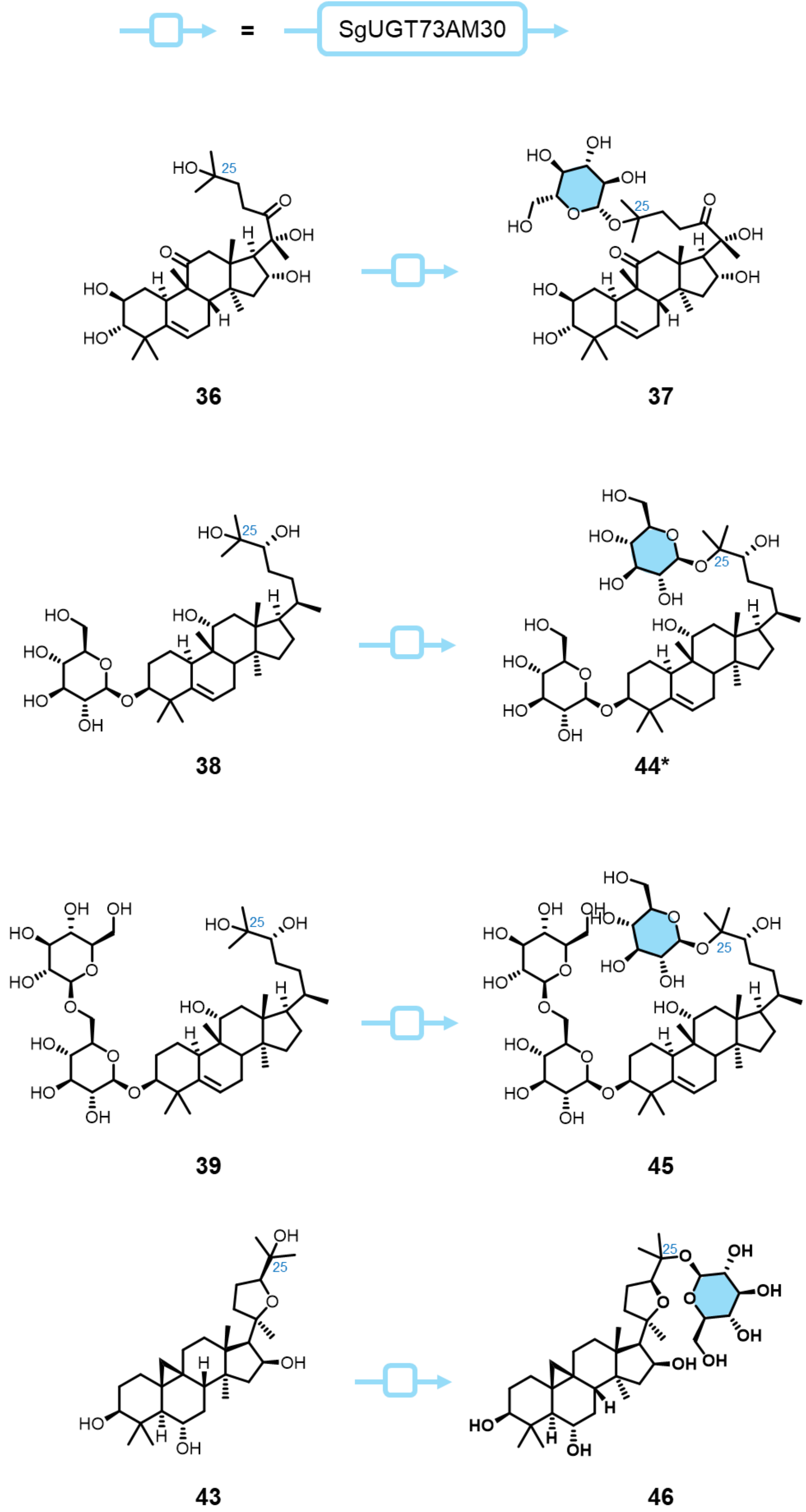

Figure S53. Characterized glycosylation activity of SgUGT73AM30. An asterisk denotes that NMR data are not available for this product.

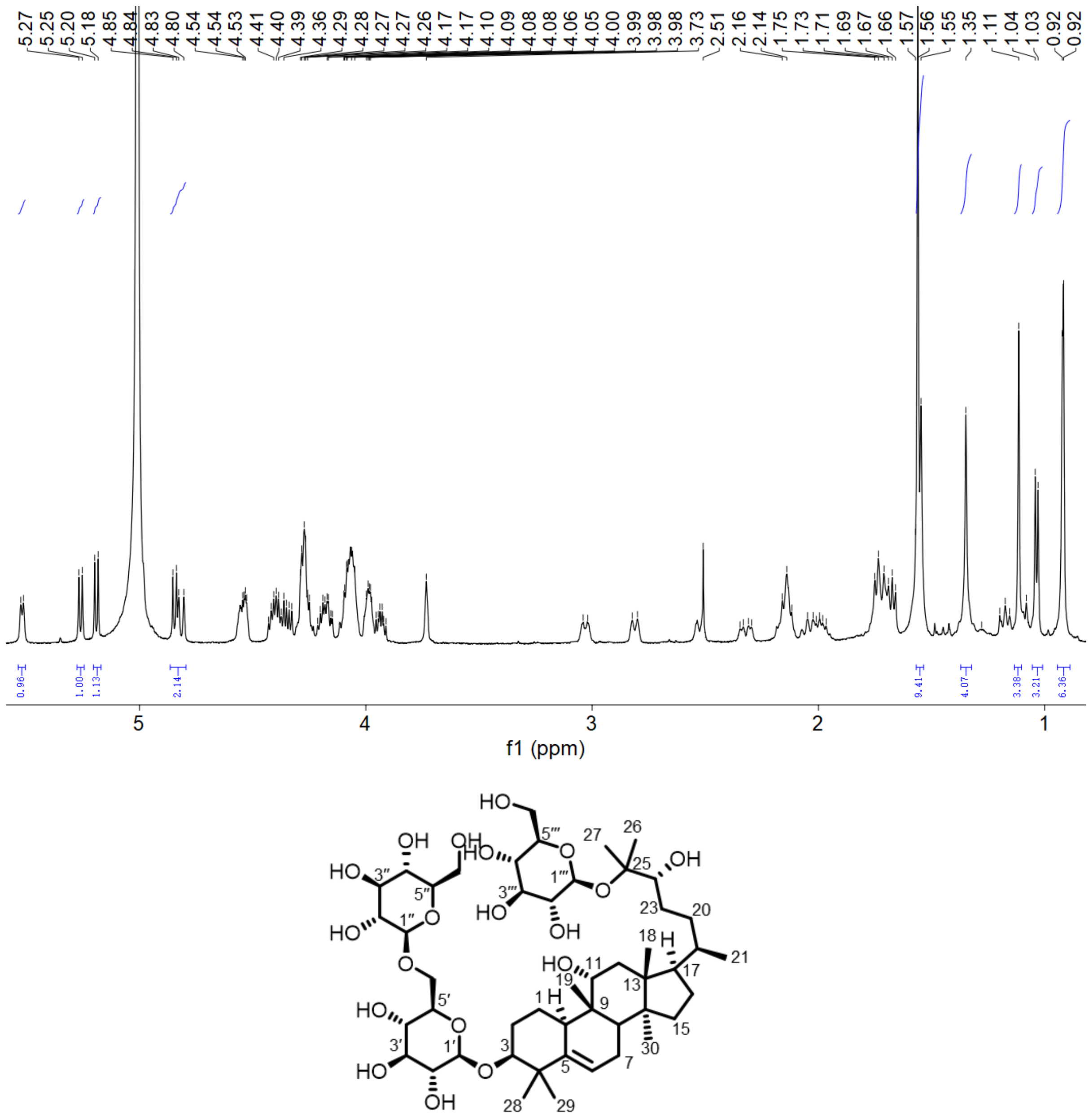

Figure S54. ^{1}H (400 MHz, pyridine-*d*5) NMR spectrum of mogroside IIA2 25-*O*-glucoside (45).

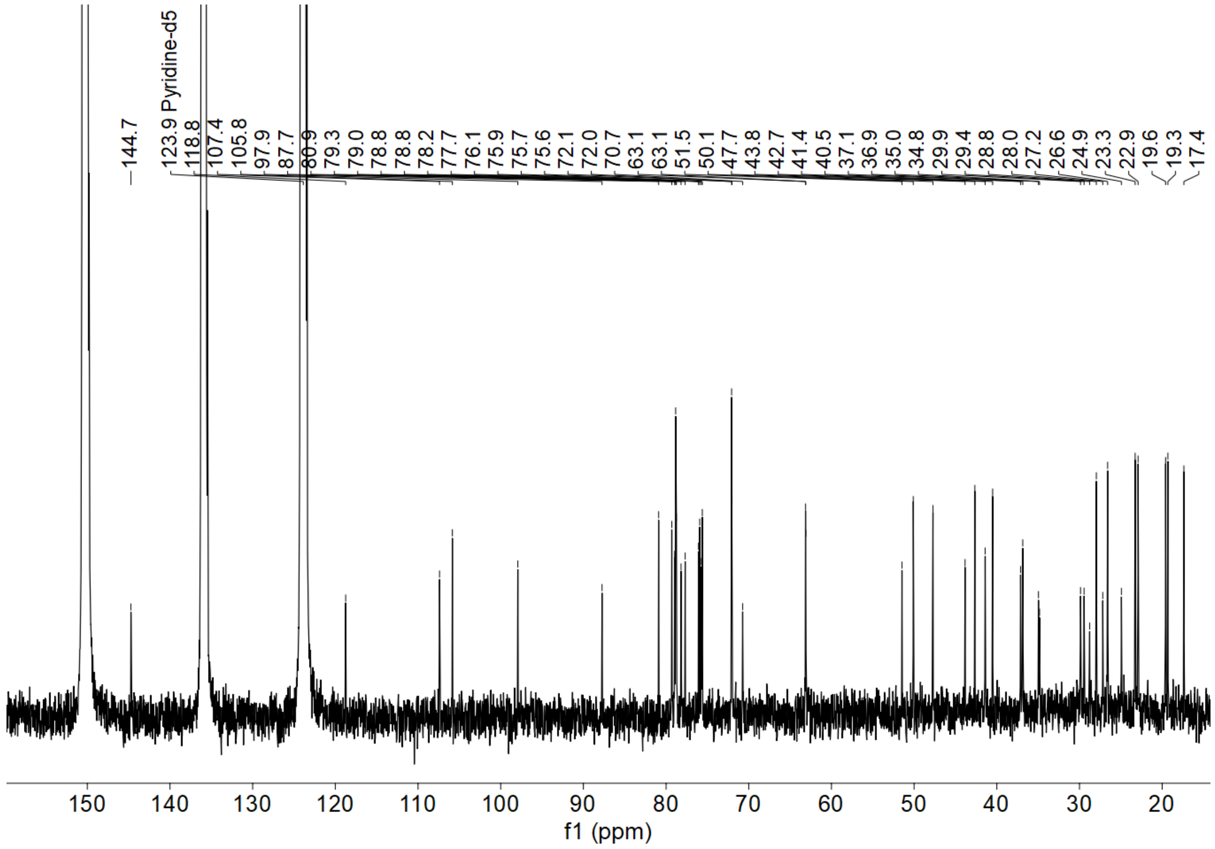

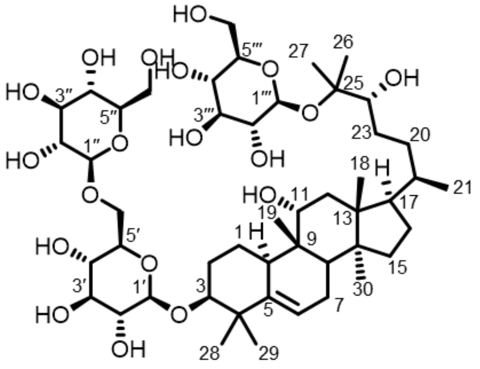

Figure S55. ^{13}C (125 MHz, pyridine-d_5) NMR spectrum of mogroside IIA2 25-*O*-glucoside (45). δ_C (ppm): 27.2 (C-1); 29.9 (C-2); 87.7 (C-3); 42.7 (C-4); 144.7 (C-5); 118.8 (C-6); 24.9 (C-7); 43.8 (C-8); 40.5 (C-9); 37.1 (C-10); 77.7 (C-11); 41.4 (C-12); 47.7 (C-13); 50.1 (C-14); 35.0 (C-15); 28.8 (C-16); 51.5 (C-17); 17.4 (C-18); 26.6 (C-19); 36.9 (C-20); 19.3 (C-21); 34.8 (C-22); 29.4 (C-23); 75.7 (C-24); 80.9 (C-25); 22.9 (C-26); 23.3 (C-27); 28.0 (C-28); 26.6 (C-29); 19.6 (C-30); 107.4(C-1′); 75.9 (C-2′); 78.8 (C-3′); 72.0 (C-4′); 78.2 (C-5′); 70.7 (C-6′); 105.8 (C-1″); 75.6 (C-2″); 78.8 (C-3″); 72.1 (C-4″); 78.8 (C-5″); 63.1 (C-6″); 97.9 (C-1‴); 76.1 (C-2‴);79.0 (C-3‴); 72.1 (C-4‴); 79.3 (C-5‴); 63.1 (C-6‴).

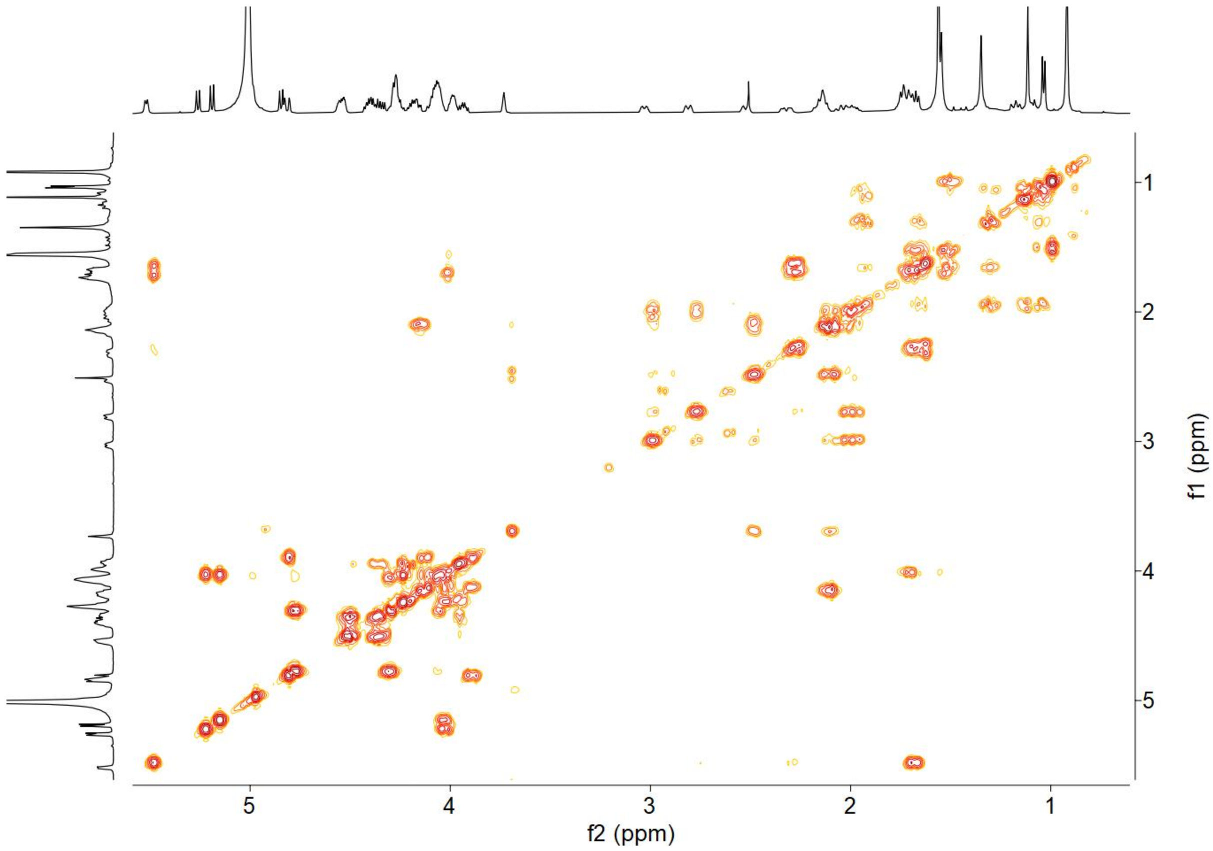

Figure S56. H-H COSY spectrum of mogroside IIA2 25-*O*-glucoside (45).

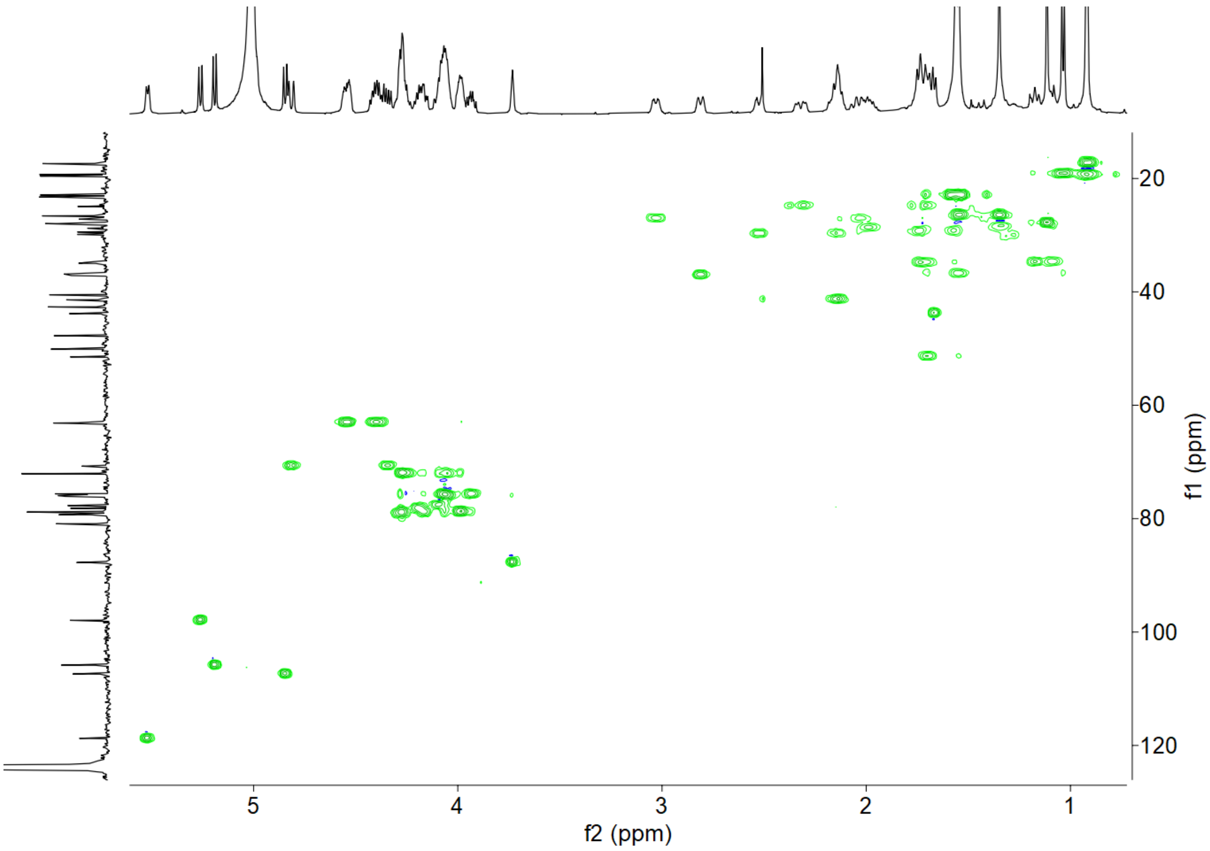

Figure S57. HSQC spectrum of mogroside IIA2 25-*O*-glucoside (45).

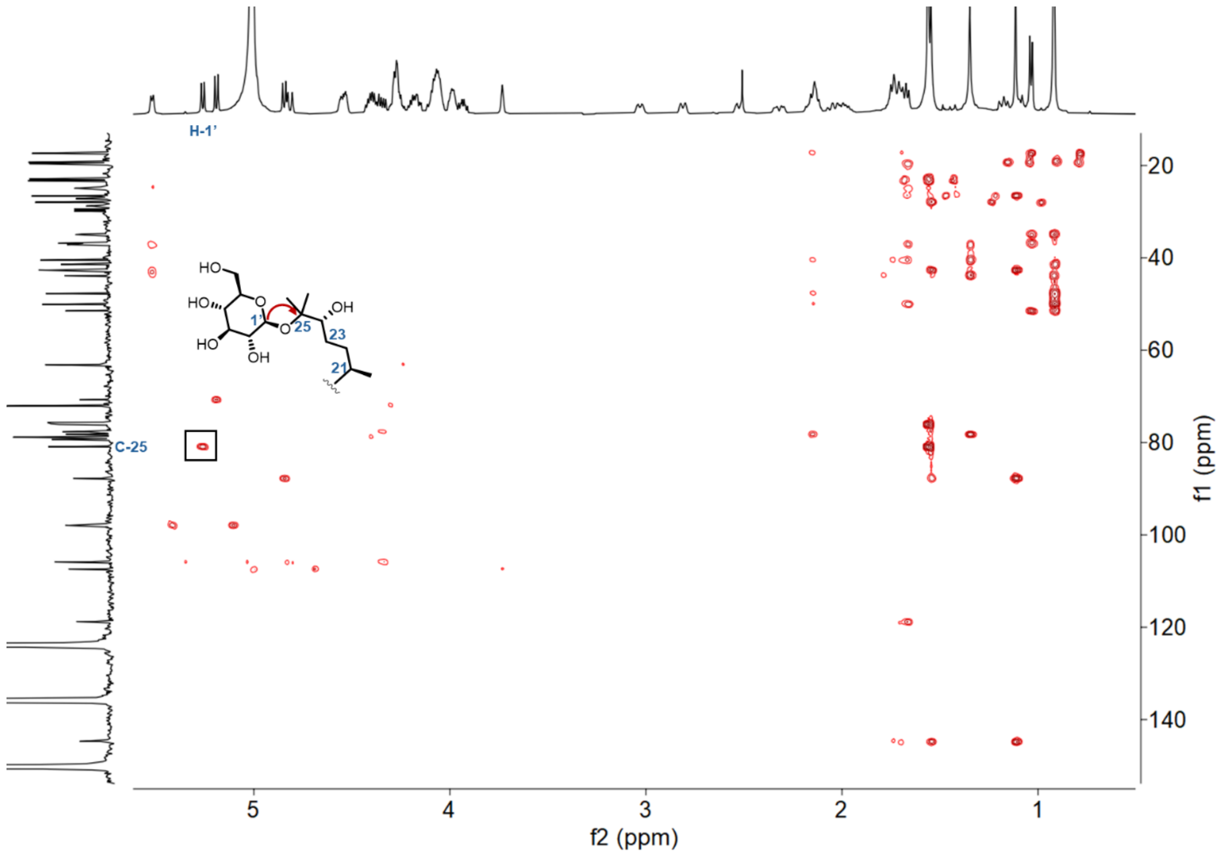

Figure S58. HMBC spectrum of mogroside IIA2 25-*O*-glucoside (45).

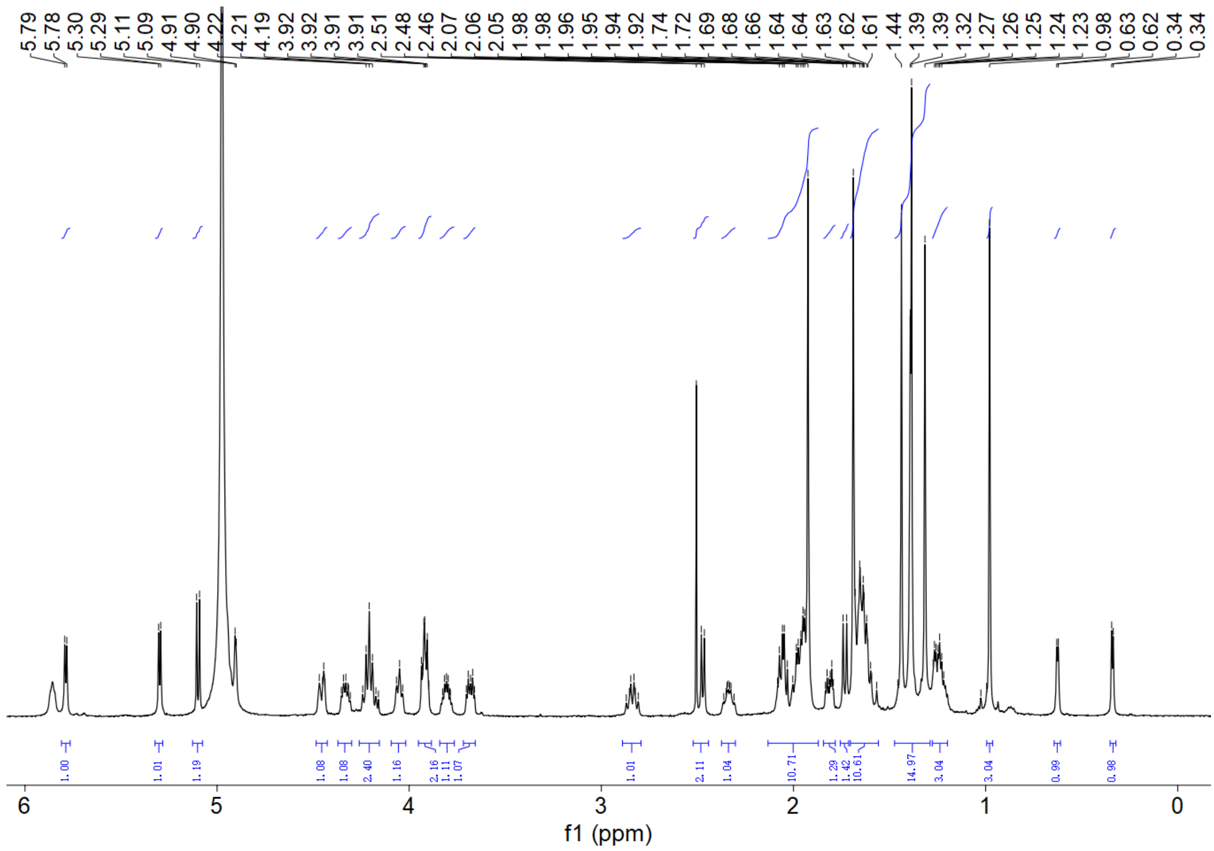

Figure S59. ^{1}H (400 MHz, pyridine-d_5) NMR spectrum of cycloastragenol 25-*O*-glucoside (46).

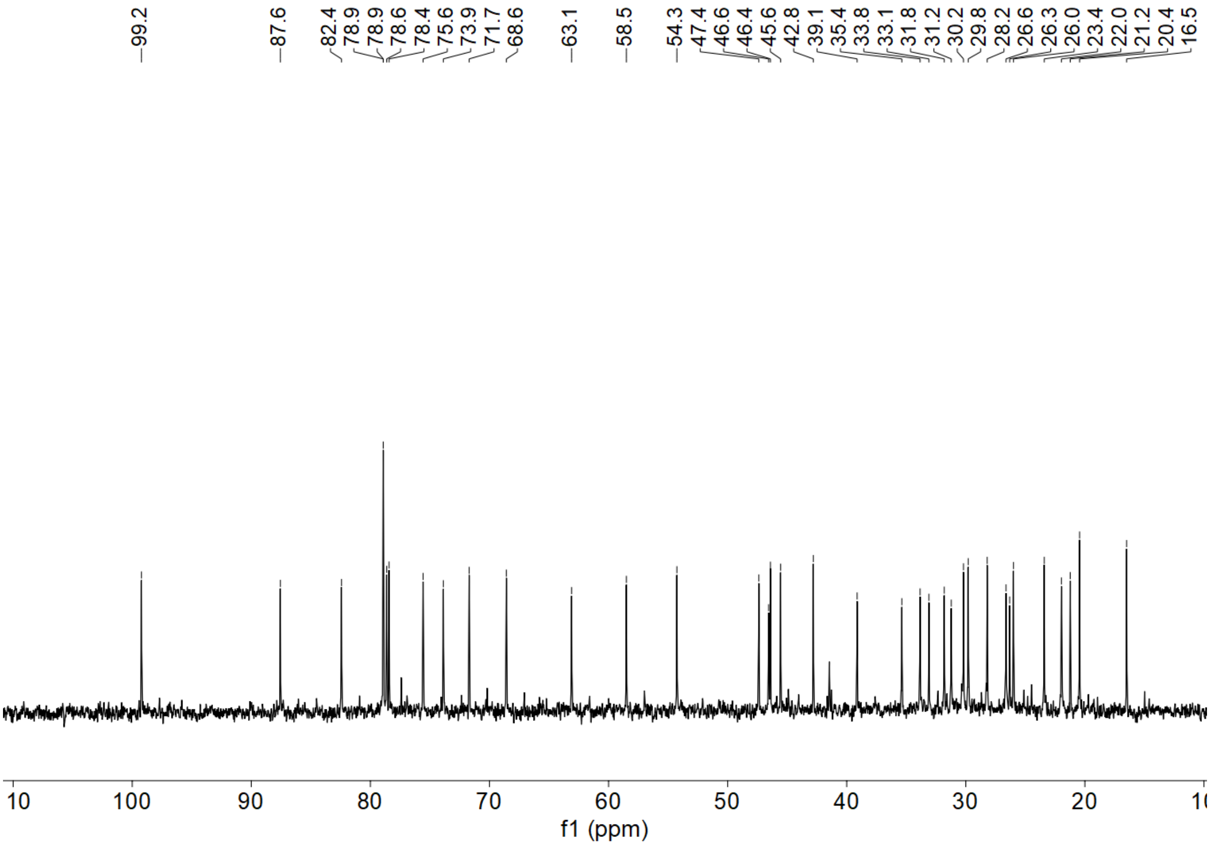

Figure S60. ^{13}**C (125 MHz, pyridine-*d*$_5$) NMR spectrum of cycloastragenol 25-*O*-glucoside (46).**

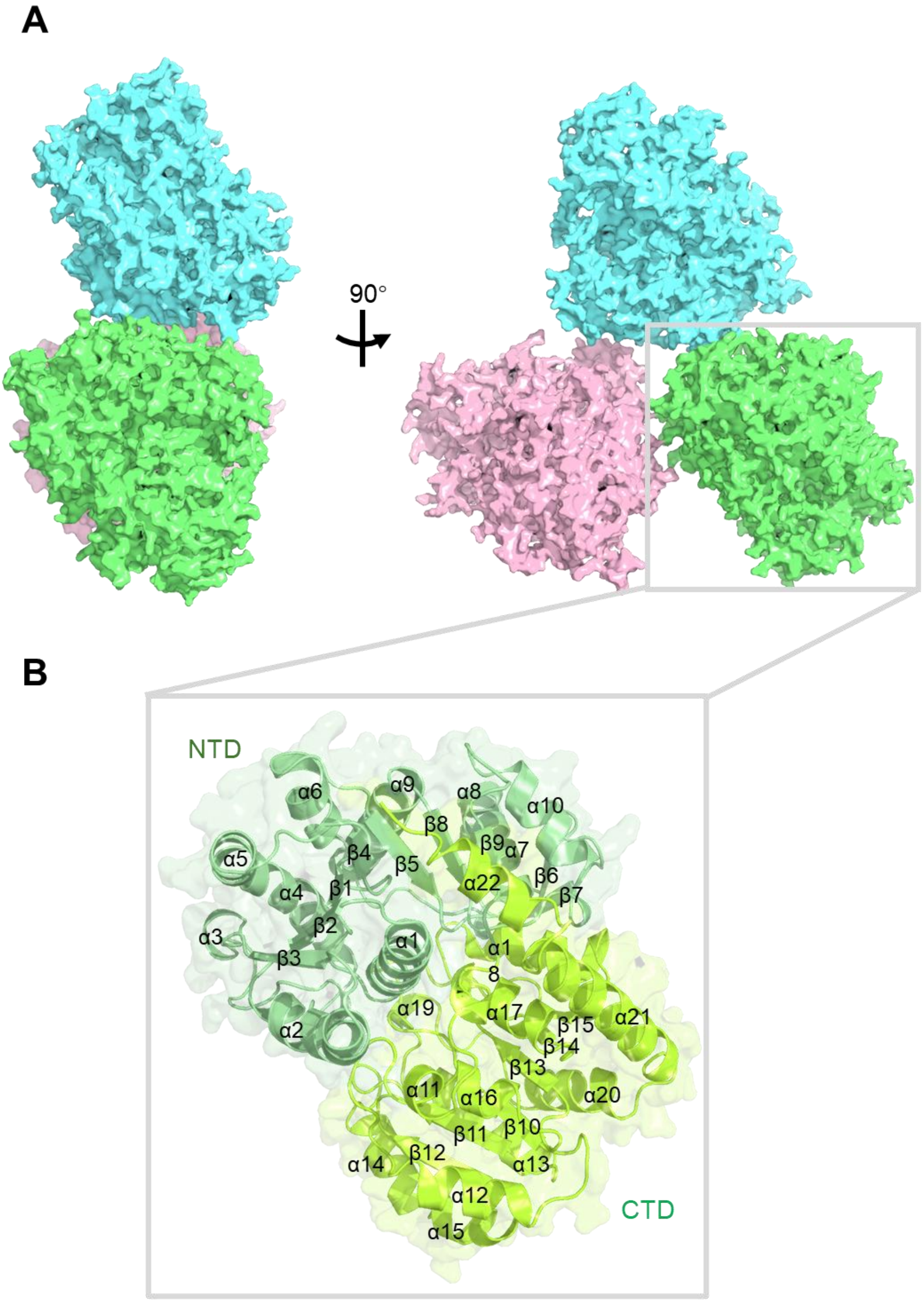

Figure S61. Overview of SgUGT73AM30 crystal structure. (**A**) A homotrimer crystal structure of SgUGT73AM30. (**B**) The monomeric protein structure consists of NTD domain and CTD domain, containing 22 α-helices and 15 β-sheets.

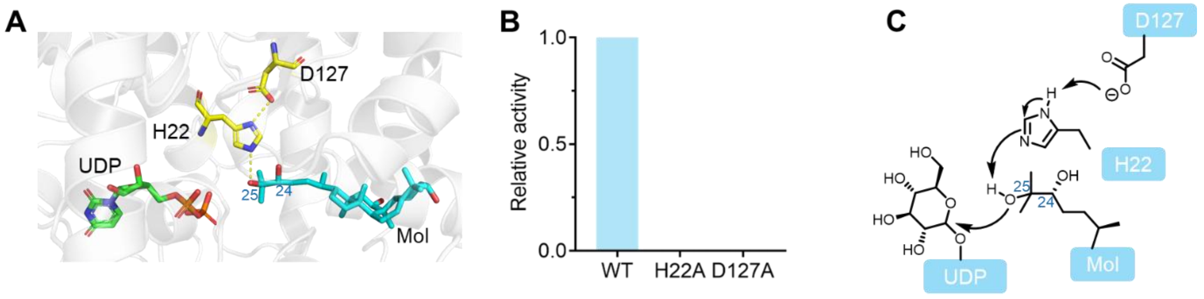

Figure S62. H22-D126-aided deprotonation of C25-hydroxyl in mogrol (Mol, 1).

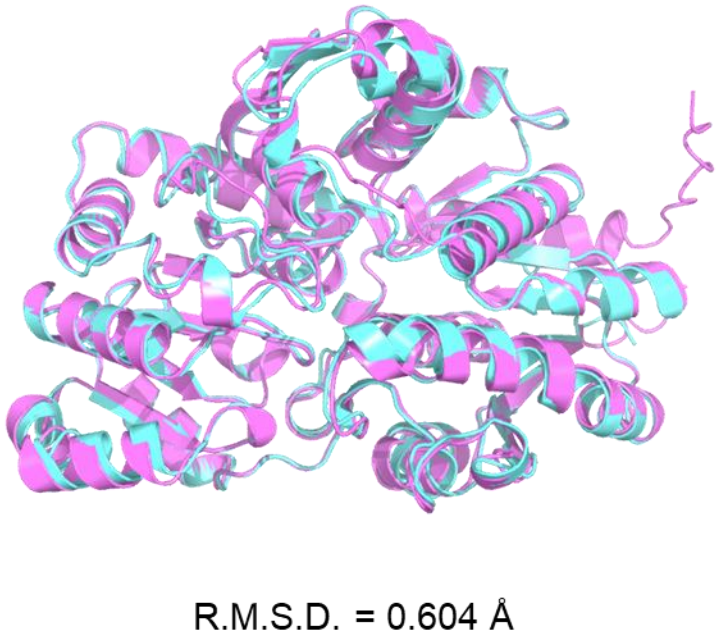

Figure S63. Comparison of the AlphaFold-predicted SgUGT73AM19 structure (purple) with the crystal structure of SgUGT73AM30 (cyan).

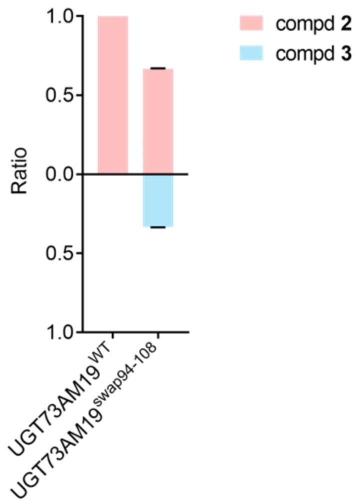

Protein	Sequences
UGT73AM19$^{swap94-108}$	MASPRHTPHFLLFPFMAQGHMIPMIDLARLLAQRGVIITIITTPHN<br>AARYHSVLARAIDSGLHIHVLQLQFPCKEGGLPEGCENVDLLPSL<br>**GSASTFFRATCLLYD**PSEKLFEELIPRPTCIISDMCLPWTMRIALK<br>YHVPRLVFYSLSCFFLLCMRSLKNNLALISSKSDSEFVTFSDLPD<br>PVEFLKSELPKSTDEDLVKFSYEMGEADRQSYGVILNLFEEMEP<br>KYLAEYEKERESPERVWCVGPVSLCNDNKLDKAERGNKASIDE<br>YKCIRWLDGQQPSSVVYVSLGSLCNLVTAQIIELGLGLEASKKPFI<br>WVIRRGNITEELQKWLVEYDFEEKIKGRGLVILGWAPQVLILSHP<br>AIGCFLTHCGWNSSIEGISAGVPMVTWPLFADQVFNEKLIVQILRI<br>GVSVGTETTMNWGEEEEKGVVVKREKVREAIEIVMDGDEREER<br>RERCKELAETAKRAIEEGGSSHRNLTMLIEDIIHGGGLSYEKGSC<br>R*

Figure S64. *In vitro* assay of SgUGT73AM19$^{swap94-108}$. The resulting chimeric sequence is shown above, with the swapped segment highlighted in bold. Data are presented as the mean ± SD (n = 3 biological replicates).

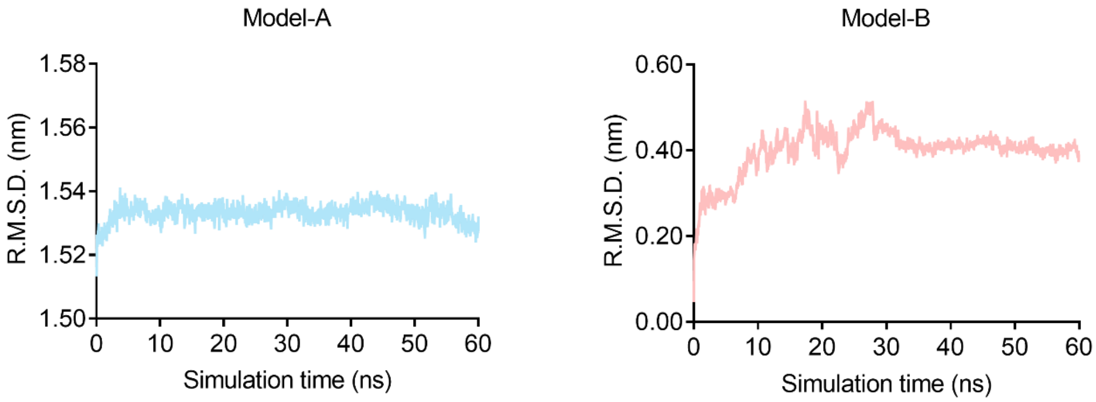

Figure S65. The plot of R.M.S.D values over 60 ns for Model-A and Model-B.

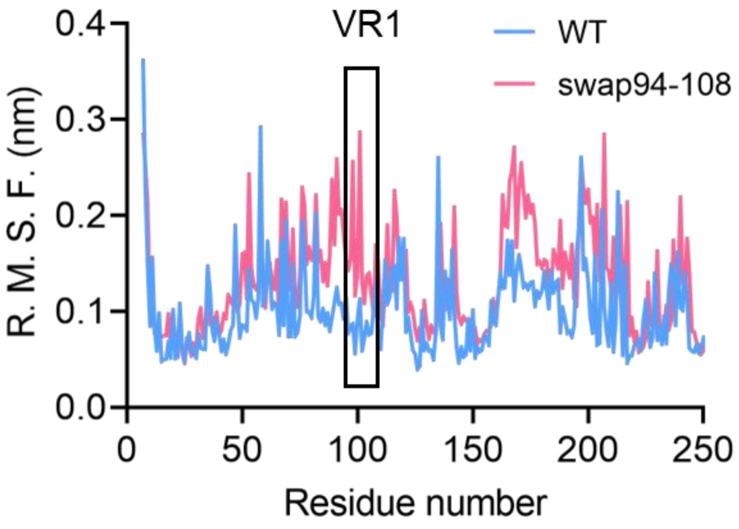

Figure S66. The plot of R.S.M.F. values of NTD domain in Model-A and Model-B.

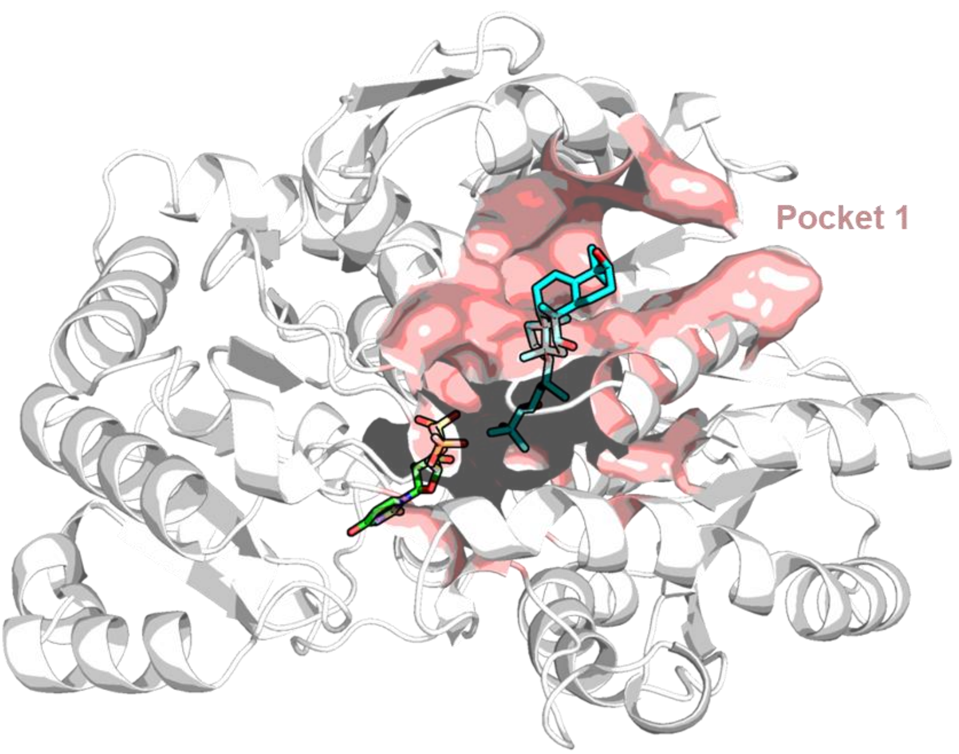

SgUGT73AM30-mogrol-UDP complex

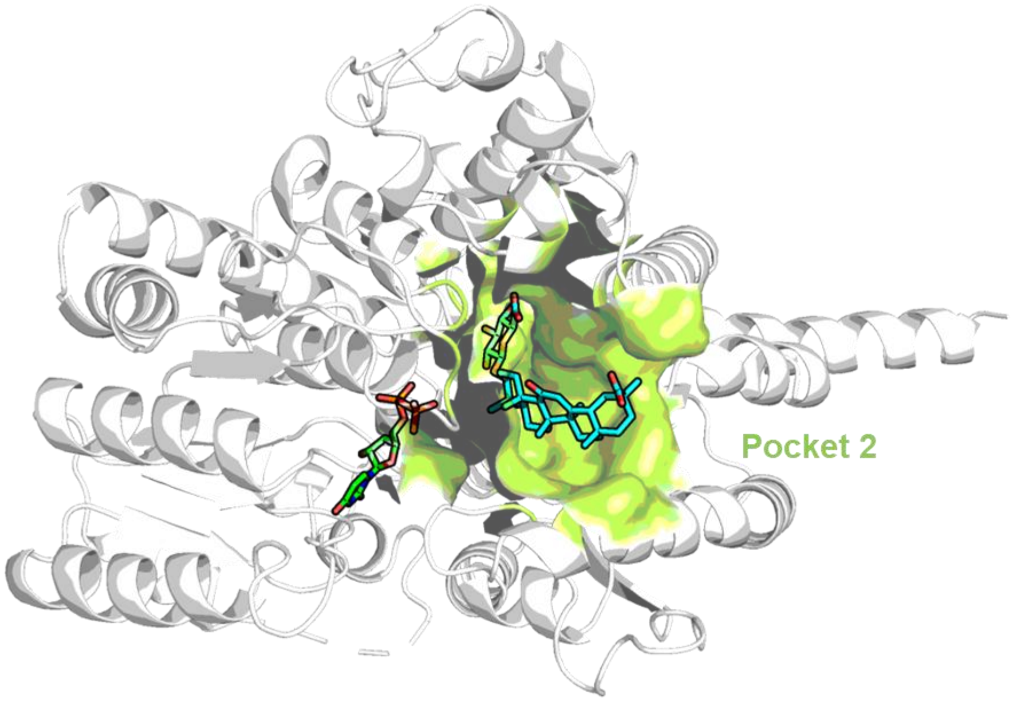

GuUGT73P12-glycyrrhetinic acid 3-*O*-monoglucuronide-UDP complex
(PDB ID: 7C2X)

Figure S67. Structural divergence of UGT73 substrate-binding pockets.

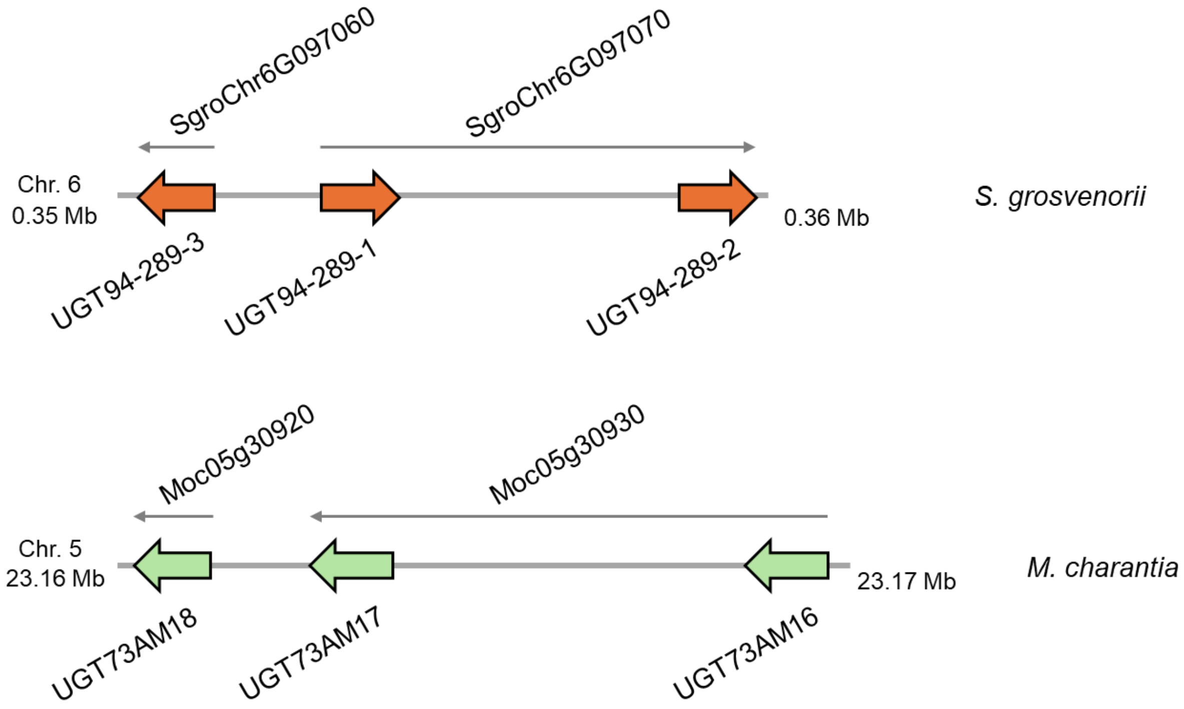

Figure S68. Representative cases of genomic annotation discrepancy. Gray arrows denote the originally annotated gene models, while colored arrows indicate the actual gene arrangements as revealed by manual curation.

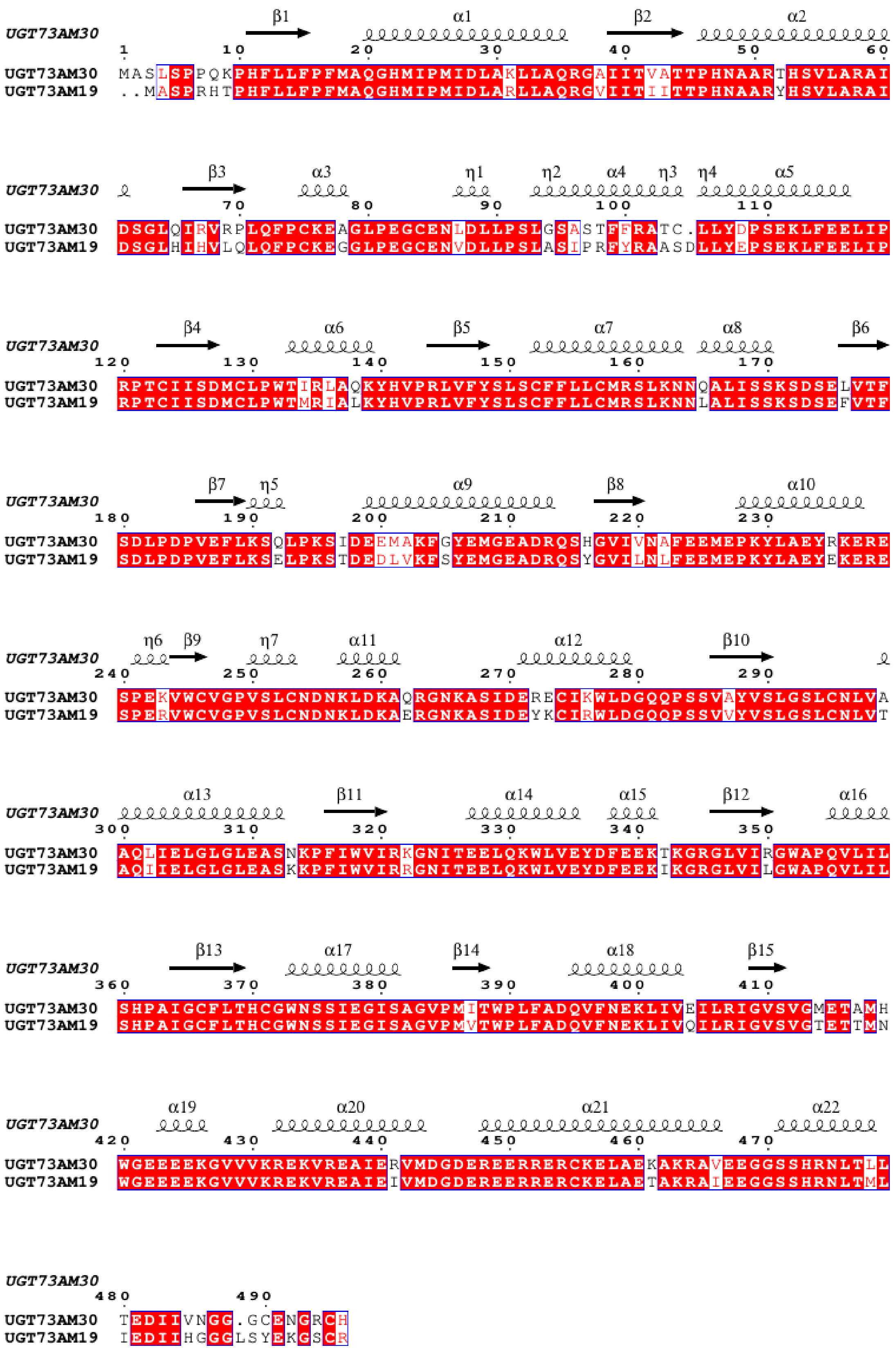

Figure S69. Pairwise sequence alignment of SgUGT73AM30 and SgUGT73AM19.

Table S1. Statistics of different versions of *S. grosvenorii* genome

	S. grosvenorii genome assembly		
	Version 1	Version 2	Version 3
Pseudochromosomes	/	/	14
Total scaffolds	12,772	4,128	42
Assembly size (Mb)	420.15	469.52	315.62
Scaffold N50 (Mb)	0.10	0.43	21.88
Longest scaffold (Mb)	0.80	7.68	34.40
Contig N50 (Mb)	0.03	/	/
GC content (%)	33.39	33.49	33.64
Protein	Unpublished	30,565	21,096
BUSCO (%)	Unpublished	89.2	98.7
Reference	(*22*)	(*28*)	This study

Table S2. BUSCO evaluation o *S. grosvenorii* and *T. pustulata* gene annotation.

	Annotation BUSCO quality (%)	
	S. grosvenorii	*T. pustulata*
Complete BUSCOs	98.9	91.6
Complete Single-Copy BUSCOs	97.6	90.6
Complete Duplicated BUSCOs	1.3	1.0
Fragmented BUSCOs	0.4	4.6
Missing BUSCOs	0.7	3.8
Total BUSCO groups searched	100.0	100.0

Table S3. Distribution of UGT family genes in Cucurbitaceae species identified by HMM-based search.

	Bhis	Clan	Lsic	Cmos	Csat	Gpen	Hped	Lcyl	Mcha	Sedu	Sgro	Tang	Tpus
UGT94	4	6	9	5	6	13	4	8	8	12	7	5	6
UGT93	3	3	3	5	3	2	0	4	3	2	2	3	3
UGT92	1	1	2	2	2	2	1	2	2	2	2	2	1
UGT91	6	5	6	7	8	11	5	9	4	10	7	6	7
UGT90	1	2	2	2	1	1	1	3	1	1	2	1	3
UGT89	3	3	3	1	1	1	3	1	2	1	4	2	1
UGT87	2	3	4	3	2	4	3	3	2	3	2	4	2
UGT86	0	1	1	2	1	1	1	1	1	1	1	1	1
UGT85	6	11	11	13	13	6	5	6	7	11	8	11	7
UGT83	2	3	3	2	0	1	1	5	2	3	5	1	2
UGT82	1	1	1	1	1	1	1	1	1	2	1	1	1
UGT79	3	2	3	3	5	2	5	3	4	4	4	4	5
UGT78	1	1	2	2	2	1	2	2	2	1	3	3	1
UGT76	2	3	3	4	4	9	4	6	6	7	4	6	2
UGT74	19	21	20	30	23	30	24	30	28	26	27	32	20
UGT73	14	16	17	13	13	30	7	14	22	19	24	16	15
UGT72	5	5	4	11	4	5	4	5	5	7	8	5	4
UGT71	8	5	7	5	8	20	8	2	7	8	6	8	5
UGT709	5	5	7	6	4	10	6	9	6	8	7	7	3
UGT708	1	1	1	1	1	1	1	1	1	1	1	1	1

Table S4. Distribution of UGT-related orthogroups in Cucurbitaceae species identified by OrthoFinder analysis.

Orthogroup	Bhis	Clan	Lsic	Cmos	Csat	Gpen	Hped	Lcyl	Mcha	Sedu	Sgro	Tang	Tpus
OG00	2	5	6	6	8	2	2	2	2	1	2	4	4
OG01	4	4	4	4	4	2	0	2	4	6	4	5	2
OG02	3	5	7	3	4	0	1	0	3	4	7	3	0
OG03	3	2	5	2	6	5	1	1	3	4	1	1	1
OG04	1	2	2	2	1	3	1	3	1	5	2	2	1
OG05	3	3	3	1	1	1	3	1	2	1	4	2	1
OG06	2	2	0	2	2	2	2	4	1	2	1	4	1
OG07	2	2	3	2	2	2	2	2	1	1	1	3	1
OG08	1	1	2	5	1	0	3	4	2	1	1	1	1
OG09	2	4	3	2	2	1	1	1	1	2	1	1	1
OG10	2	1	2	4	1	2	1	1	1	1	3	1	1
OG11	1	2	2	3	1	2	1	1	1	1	1	1	4
OG12	2	1	2	2	2	0	1	2	4	1	2	1	0
OG13	1	2	2	2	1	1	1	2	1	3	1	2	0
OG14	1	1	1	1	1	1	2	3	2	0	2	2	2
OG15	2	2	1	2	1	1	1	2	1	2	1	2	1
OG16	2	1	2	2	1	0	2	2	0	2	2	2	1
OG17	1	1	1	2	1	5	1	1	0	2	1	1	1
OG18	1	1	1	1	1	2	1	2	3	2	1	2	0
OG19	0	1	1	0	0	7	1	2	3	0	1	2	0
OG20	2	2	2	2	2	0	0	2	1	1	1	2	1
OG21	1	2	1	2	2	2	1	1	1	1	1	1	2
OG22	0	1	1	1	0	0	1	2	0	5	2	3	1
OG23	0	0	0	1	1	6	1	2	1	2	1	1	1
OG24	2	2	1	1	1	1	2	1	2	1	1	1	1
OG25	1	1	1	3	1	2	0	0	2	1	1	2	1
OG26	1	0	2	0	1	8	0	0	0	0	0	2	2
OG27	2	1	1	0	2	0	2	0	3	2	1	2	0
OG28	1	1	1	1	1	2	2	1	1	1	1	2	1

Orthogroup	Bhis	Clan	Lsic	Cmos	Csat	Gpen	Hped	Lcyl	Mcha	Sedu	Sgro	Tang	Tpus
OG29	1	1	1	3	1	0	0	2	2	1	1	1	2
OG30	1	1	1	1	0	1	0	3	1	2	4	0	1
OG31	1	1	1	2	0	3	1	1	1	2	1	1	1
OG32	1	1	1	1	1	2	1	1	1	2	1	1	1
OG33	1	1	1	0	1	2	2	1	1	1	1	1	1
OG34	1	1	2	1	2	0	1	0	1	2	1	1	1
OG35	0	1	1	1	0	3	1	1	2	2	1	0	1
OG36	1	1	0	1	1	1	1	3	1	1	1	1	1
OG37	1	1	1	1	1	1	1	1	1	1	1	2	1
OG38	2	1	1	2	1	0	1	0	1	2	1	1	1
OG39	0	0	0	2	0	0	2	2	0	5	1	2	0
OG40	3	2	3	0	6	0	0	0	0	0	0	0	0
OG41	1	2	2	1	0	0	1	2	1	1	1	1	1
OG42	1	1	1	1	1	2	1	1	1	1	1	1	1
OG43	1	1	1	1	1	1	1	1	1	2	1	1	1
OG44	1	1	1	0	0	2	0	1	1	4	1	0	1
OG45	1	1	1	1	1	1	0	0	1	2	1	2	1
OG46	1	1	1	1	0	2	1	1	1	1	1	1	1
OG47	1	1	1	1	1	1	2	1	0	1	1	1	1
OG48	1	1	1	1	1	0	1	1	1	1	3	1	0
OG49	0	0	0	1	1	2	1	1	1	2	1	2	1
OG50	1	1	1	1	1	1	1	1	1	1	1	1	1
OG51	1	2	2	0	1	0	0	1	1	1	2	1	1
OG52	1	1	1	1	1	1	1	1	1	1	1	1	1
OG53	1	1	1	1	1	1	1	1	1	1	1	1	1
OG54	0	1	1	1	0	2	1	1	1	2	1	1	1
OG55	0	1	1	2	1	1	1	1	1	1	1	1	1
OG56	1	1	0	1	1	2	1	1	1	1	1	1	0
OG57	0	0	2	1	1	0	0	0	1	2	0	2	3
OG58	1	1	1	0	0	2	0	3	1	0	1	0	2
OG59	1	1	1	2	1	1	0	0	1	1	1	1	1

Orthogroup	Bhis	Clan	Lsic	Cmos	Csat	Gpen	Hped	Lcyl	Mcha	Sedu	Sgro	Tang	Tpus
OG60	1	1	0	0	0	1	0	4	1	2	1	0	1
OG61	1	0	1	2	1	0	1	1	2	1	1	0	1
OG62	0	0	0	1	1	2	1	1	1	2	0	2	1
OG63	2	1	0	1	2	2	0	0	1	0	1	0	1
OG64	0	2	0	2	1	0	1	0	1	1	1	1	1
OG65	1	0	1	1	1	0	1	0	1	1	1	1	2
OG66	0	1	1	1	1	0	1	1	1	2	1	1	0
OG67	1	1	0	0	1	2	1	1	1	0	1	1	1
OG68	0	0	0	1	1	4	1	1	1	0	1	1	0
OG69	0	1	1	2	1	0	1	2	1	0	1	1	0
OG70	0	1	1	1	0	1	1	1	1	1	1	1	1
OG71	0	0	0	1	0	0	0	1	0	5	1	2	0
OG72	0	0	1	0	2	0	1	1	1	1	1	1	1
OG73	1	1	0	1	1	0	1	1	1	1	1	0	1
OG74	1	1	1	1	1	1	1	1	1	0	0	1	0
OG75	0	0	0	0	0	7	0	1	1	0	0	0	1
OG76	1	0	0	0	0	2	1	1	1	1	1	1	1
OG77	1	1	1	0	0	1	0	1	0	1	1	0	3
OG78	0	0	1	1	1	0	1	1	1	0	2	2	0
OG79	0	0	1	2	1	0	1	1	1	0	1	1	0
OG80	0	0	0	0	0	3	0	2	2	0	1	0	1
OG81	0	0	0	0	1	1	1	1	1	1	1	2	0
OG82	0	1	1	1	1	0	1	0	1	1	0	1	0
OG83	1	1	1	0	1	0	1	1	0	0	1	1	0
OG84	0	0	0	3	0	0	0	2	0	1	1	1	0
OG85	0	1	1	0	1	0	0	0	1	0	1	2	0
OG86	0	0	1	1	1	0	0	1	1	0	1	1	0
OG87	0	0	0	0	0	0	0	2	0	2	2	1	0
OG88	0	0	0	0	0	1	0	1	1	0	1	0	2
OG89	1	0	1	0	1	0	1	0	0	1	1	0	0
OG90	0	0	0	0	0	0	2	0	1	1	1	1	0

Orthogroup	Bhis	Clan	Lsic	Cmos	Csat	Gpen	Hped	Lcyl	Mcha	Sedu	Sgro	Tang	Tpus
OG91	0	0	0	0	0	1	0	1	2	0	1	0	1
OG92	0	0	0	0	0	6	0	0	0	0	0	0	0
OG93	0	0	0	1	0	0	1	1	1	1	0	0	1
OG94	0	0	0	0	0	5	0	0	0	0	0	0	0
OG95	0	0	0	0	0	1	0	0	1	0	1	0	1
OG96	0	0	0	0	0	4	0	0	0	0	0	0	0
OG97	0	0	0	0	0	2	0	0	1	0	1	0	0
OG98	0	0	0	0	0	1	0	0	1	0	1	0	1
OG99	0	0	0	0	0	0	0	1	1	0	1	0	1
OG100	0	0	0	0	0	0	0	1	0	0	2	1	0
OG101	0	0	0	0	0	1	0	0	0	0	1	0	1
OG102	0	0	0	0	0	0	1	0	1	0	0	0	1
OG103	1	0	0	1	0	0	1	0	0	0	0	0	0
OG104	0	0	0	0	0	0	0	0	1	0	1	0	1
OG105	0	0	0	0	0	3	0	0	0	0	0	0	0
OG106	0	0	0	0	0	1	0	1	0	0	1	0	0
OG107	0	0	0	1	0	0	0	0	1	0	1	0	0
OG108	0	0	0	0	0	0	0	0	1	0	1	0	1
OG109	0	0	0	0	0	1	0	0	0	0	1	1	0
OG110	0	0	0	0	0	0	1	0	0	1	1	0	0
OG111	0	0	0	0	0	2	0	0	0	0	0	0	0
OG112	0	0	0	0	0	0	0	0	0	0	0	0	2
OG113	0	0	1	1	0	0	0	0	0	0	0	0	0
OG114	0	0	0	0	0	0	0	0	1	0	0	1	0

Table S5. Genomic resources used in this study.

Species	Genome assembly	ID	Source
Aquilegia coerulea	Aquilegia_coerulea_v1	GCA_002738505.1	NCBI
Arabidopsis thaliana	TAIR10	GCF_000001735.3	NCBI
Aralia elata	Aralia elata genome and annotation	10.5061/dryad.69p8cz937	Dryad Digital Repository
Aristolochia fimbriata	ASM1984555v1	GCA_019845555.1	NCBI
Begonia darthvaderiana	ASM2243294v1	GCA_022432945.1	NCBI
Begonia loranthoides	ASM2243306v1	GCA_022433065.1	NCBI
Begonia masoniana	ASM2243297v1	GCA_022432975.1	NCBI
Begonia peltatifolia	ASM2243305v1	GCA_022433055.1	NCBI
Beta vulgaris	EL10.2	GCF_026745355.1	NCBI
Cannabis sativa	ASM2916894v1	GCF_029168945.1	NCBI
Centella asiatica	/	GWHFIFH00000000.1	CNCB
Citrullus lanatus	PI_254622	PI_254622_v1	Watermelon Genome Database (http://www.watermelondb.cn)
Coffea arabica	Coffea_Arabica_ET-39_HiFi	GCF_036785885.1	NCBI
Coptis chinensis	ASM1568090v1	GCA_015680905.1	NCBI
Coriaria nepalensis	/	10.6084/m9.figshare.22263274	figshare
Corylus avellana	CavTom2PMs-1.0	GCF_901000735.1	NCBI
Cucumis sativus	Chinese Long_v3	/	CuGenDBv2
Cucurbita moschata	Rifu_v1	/	CuGenDBv2
Cucurbita pepo	MU-CU-16_v4.1	/	CuGenDBv2
Cyclocarya paliurus	ASM2985693v1	GCA_029856935.1	NCBI
Datisca glomerata	ASM325502v1	GCA_003255025.1	NCBI
Daucus carota	DH1_v3.0	GCF_001625215.2	NCBI

Species	Genome assembly	ID	Source
Glycine max	Glycine_max_v4.0	GCF_000004515.6	NCBI
Gynostemma pentaphyllum	JGL_1.0	GCA_020536105.1	Dr. Ding Huang
Herpetospermum pedunculosum	/	10.6084/m9.figshare.21626153.v2	figshare
Juglans regia	Walnut_2.0	GCF_001411555.2	NCBI
Momordica charantia	OHB3-1_v2	/	CuGenDBv2
Nymphaea colorata	ASM883128v2	GCF_008831285.2	NCBI
Oryza sativa	IRGSP-1.0	GCA_001433935.1	Ensembl Plants
Papaver somniferum	ASM357369v1	GCF_003573695.1	NCBI
Persea americana	XTBG_Pame_1.0	GCA_051132755.1	NCBI
Prunus persica	Prunus_persica_NCBIv2	GCF_000346465.2	NCBI
Saponaria officinalis	Saponaria_officinalis_var_Norfolk2_v1.0	GCA_040167595.1	NCBI
Sicyos edulis	Chayote	/	CuGenDBv2
Siraitia grosvenorii	/	/	this study
Stevia rebaudiana	/	10.6084/m9.figshare.14169491	figshare
Thladiantha pustulata	ASM4400753v1	GCA_044007535.1	NCBI
Trichosanthes anguina	Snakegourd	/	CuGenDBv2
Vitis vinifera	ASM3070453v1	GCF_030704535.1	NCBI

Table S6. UGT73 subfamily proteins in syntenic UGT73 tandem array.

Species	Locations	UGT73AT/FXs	UGT73AMs	UGT73GBs	Other UGT73s
Begonia masoniana	Chr. 4<br>(52.09-52.11 Mb)	×	×	×	BmUGT73GF5<br>BmUGT73GF6
Gynostemma pentaphyllum	CM100508.1 (28.11-28.23 Mb)	GpUGT73FX1	GpUGT73AM10<br>GpUGT73AM11<br>GpUGT73AM12<br>GpUGT73AM13<br>GpUGT73AM14	×	×
Thladiantha pustulata	JBINJJ010000016 (9.83-9.86 Mb)	TpUGT73AT5	TpUGT73AM28<br>TpUGT73AM29	TpUGT73GB2	×
Siraitia grosvenorii	Chr. 2<br>(2.09-2.15 Mb)	SgUGT73AT4	SgUGT73AM1<br>SgUGT73AM2<br>SgUGT73AM19<br>SgUGT73AM20<br>SgUGT73AM21<br>SgUGT73AM22<br>SgUGT73AM30	SgUGT73GB1	×
Momordica charantia	Chr. 5<br>(23.14-23.22 Mb)	McUGT73AT6<br>McUGT73AT7	McUGT73AM15<br>McUGT73AM16<br>McUGT73AM17<br>McUGT73AM18	×	×
Cucumis sativus	Chr. 3<br>(28.11-28.23 Mb)	CsUGT73AT1	CsUGT73AM3<br>CsUGT73AM7<br>CsUGT73AM8<br>CsUGT73AM9	×	×

Table S7. Plasmids used in this study.

Plasmids	Resistance	Description	Usage	Source
pET-28a(+)	Kan	Protein expression vector	Plasmid construction	Novagen (U.S.A.)
pUGT73AM1	Kan	pET-28a-His-SgUGT73AM1	Candidates validation	this study
pUGT73AM2	Kan	pET-28a-His-SgUGT73AM2	Candidates validation	this study
pUGT73AM19	Kan	pET-28a-His-SgUGT73AM19	Candidates validation	this study
pUGT73AM20	Kan	pET-28a-His-SgUGT73AM20	Candidates validation	this study
pUGT73AM21	Kan	pET-28a-His-SgUGT73AM21	Candidates validation	this study
pUGT73AM22	Kan	pET-28a-His-SgUGT73AM22	Candidates validation	this study
pUGT73AM30	Kan	pET-28a-His-SgUGT73AM30	Candidates validation	this study
pUGT73AT4	Kan	pET-28a-His-SgUGT73AT4	Candidates validation	this study
pUGT73GB1	Kan	pET-28a-His-SgUGT73GB1	Candidates validation	this study
pUGT73AM10	Kan	pET-28a-His-GpUGT73AM10	Candidates validation	this study
pUGT73AM11	Kan	pET-28a-His-GpUGT73AM11	Candidates validation	this study
pUGT73AM12	Kan	pET-28a-His-GpUGT73AM12	Candidates validation	this study
pUGT73AM13	Kan	pET-28a-His-GpUGT73AM13	Candidates validation	this study
pUGT73AM14	Kan	pET-28a-His-GpUGT73AM14	Candidates validation	this study
pUGT73FX1	Kan	pET-28a-His-GpUGT73FX1	Candidates validation	this study
pUGT73AM15	Kan	pET-28a-His-McUGT73AM15	Candidates validation	this study
pUGT73AM16	Kan	pET-28a-His-McUGT73AM16	Candidates validation	this study
pUGT73AM17	Kan	pET-28a-His-McUGT73AM17	Candidates validation	this study
pUGT73AM18	Kan	pET-28a-His-McUGT73AM18	Candidates validation	this study
pUGT73AT6	Kan	pET-28a-His-McUGT73AT6	Candidates validation	this study
pUGT73AT7	Kan	pET-28a-His-McUGT73AT7	Candidates validation	this study

Plasmids	Resistance	Description	Usage	Source
pUGT73AM28	Kan	pET-28a-His-TpUGT73AM28	Candidates validation	this study
pUGT73AM29	Kan	pET-28a-His-TpUGT73AM29	Candidates validation	this study
pUGT73AT5	Kan	pET-28a-His-TpUGT73AT5	Candidates validation	this study
pUGT73GB2	Kan	pET-28a-His-TpUGT73GB2	Candidates validation	this study
pUGT73AM30opt	Kan	pET-28a-His-SgUGT73AM30opt	Large-scale protein purification	this study
pUGT73AM30^{L87V}	Kan	pET-28a-His-SgUGT73AM30opt-L87V	Mutants assessment	this study
pUGT73AM30^{F100Y}	Kan	pET-28a-His-SgUGT73AM30opt-F100Y	Mutants assessment	this study
pUGT73AM30^{M201L}	Kan	pET-28a-His-SgUGT73AM30opt-M201L	Mutants assessment	this study
pUGT73AM30^{A202V}	Kan	pET-28a-His-SgUGT73AM30opt-A202V	Mutants assessment	this study
pUGT73AM30^{G205S}	Kan	pET-28a-His-SgUGT73AM30opt-G205S	Mutants assessment	this study
pUGT73AM30^{quard}	Kan	pET-28a-His-SgUGT73AM30opt-L87V/F100Y/M201L/A202V	Mutants assessment	this study
pUGT73AM30^{swapVR1}	Kan	pET-28a-His-SgUGT73AM30opt-swap94-108	Mutants assessment	this study
pUGT73AM30^{swapVR2}	Kan	pET-28a-His-SgUGT73AM30opt-swap192-205	Mutants assessment	this study
pUGT73AM30$^{swapVR1/VR2}$	Kan	pET-28a-His-SgUGT73AM30opt-swap192-205/swap192-205	Mutants assessment	this study
pUGT73AM30$^{swap94-100}$	Kan	pET-28a-His-SgUGT73AM30opt-swap94-100	Mutants assessment	this study
pUGT73AM30$^{swap101-108}$	Kan	pET-28a-His-SgUGT73AM30opt-swap101-108	Mutants assessment	this study
pUGT73AM30$^{swapVR1/mono}$	Kan	pET-28a-His-SgUGT73AM30opt-swap94-108-L87V	Mutants assessment	this study
pUGT73AM30$^{swapVR1/di}$	Kan	pET-28a-His-SgUGT73AM30opt-swap94-108-L87V/M201L	Mutants assessment	this study
pUGT73AM30$^{swapVR1/tri}$	Kan	pET-28a-His-SgUGT73AM30opt-swap94-108-L87V/M201L/A202V	Mutants assessment	this study
pUGT73AM19^{swapVR1}	Kan	pET-28a-His-SgUGT73AM19-swap92-107	Mutants assessment	this study

Table S8. Primers used in this study.

UGT constructs	Primers	Sequences (5' to 3')
pUGT73AM1	AM1-F	ATCATCATCACAGCAGCGGCATGCTGATGCACGCTCTCAC
	AM1-R	CGGAGCTCGAATTCGGATCCTCAATTAACTATAGTAGGAAAATCATCAGCATCATCC
pUGT73AM2	AM2-F	ATCATCATCACAGCAGCGGCATGGATTCCCCACCCCACA
	AM2-R	CGGAGCTCGAATTCGGATCCTCACAACCTCCTCAATGAAGTACCTATATC
pUGT73AM19	AM19-F	ATCATCATCACAGCAGCGGCATGGCTTCTCCTCGCCACAC
	AM19-R	CGGAGCTCGAATTCGGATCCTCAGCGACAACTTCCTTTCTCATAA
pUGT73AM20	AM20-F	ATCATCATCACAGCAGCGGCATGGATTCCCCACGCCAC
	AM20-R	CGGAGCTCGAATTCGGATCCTCAATCAGCAGTGTGAGGTTTCGA
pUGT73AM21	AM21-F	ATCATCATCACAGCAGCGGCATGGATTCCTCACCCCAAAAACC
	AM21-R	CGGAGCTCGAATTCGGATCCCTAACAGTCACAACCTCCTCCTCG
pUGT73AM22	AM22-F	ATCATCATCACAGCAGCGGCATGGATTCCCCACCCCAAAAAC
	AM22-R	CGGAGCTCGAATTCGGATCCTTAACTAATAATAATCAGATAAAACTTCCATTCTGACAA
pUGT73AM30	AM30-F	ATCATCATCACAGCAGCGGCATGGCTTCCCTTTCCCCAC
	AM30-R	CGGAGCTCGAATTCGGATCCTTAATGGCATCTTCCATTCTCACAACC
pUGT73AT4	AT4-F	ATCATCATCACAGCAGCGGCATGGCTTCCGAATCGAAACCAC
	AT4-R	CGGAGCTCGAATTCGGATCCCTAGAATCTGCTTGATGAATGATGCATGATG
pUGT73GB1	GB1-F	ATCATCATCACAGCAGCGGCATGGGCGCTGCTCAGC
	GB1-R	CGGAGCTCGAATTCGGATCCCTAAGTTCGCAGGATGTTTGATCC
pUGT73AM10	AM10-F	ATCATCATCACAGCAGCGGCATGGCTTCCCAATGCCTTG
	AM10-R	CGGAGCTCGAATTCGGATCCTCAGCAGGTTCCATTAACACAGCC
pUGT73AM11	AM11-F	ATCATCATCACAGCAGCGGCATGGCTTCCCAAAACCACAAG
	AM11-R	CGGAGCTCGAATTCGGATCCTCAAAAGGTTCCATTATCACAGCTTG
pUGT73AM12	AM12-F	ATCATCATCACAGCAGCGGCATGGCTTCCCAACACTACAAGC
	AM12-R	CGGAGCTCGAATTCGGATCCCTAATCACAGCTTGCTTGATGAGTAAAATC
pUGT73AM13	AM13-F	ATCATCATCACAGCAGCGGCATGGCTTCCCAACACTACAAGC
	AM13-R	CGGAGCTCGAATTCGGATCCCTAATCACAGCTTGCTTGATGAGTAAAATC
pUGT73AM14	AM14-F	ATCATCATCACAGCAGCGGCATGGCTTCCCTACACCACAAG
	AM14-R	CGGAGCTCGAATTCGGATCCTCAGCAGGTTCCATTATCACAGCTTG
pUGT73FX1	FX1-F	ATCATCATCACAGCAGCGGCATGGCTTCCATTTCCCATTCTC
	FX1-R	CGGAGCTCGAATTCGGATCCTCAGAAGTTAGGCCGACTCCAG
pUGT73AM15	AM15-F	ATCATCATCACAGCAGCGGCATGGCCATGGCTTCCCC
	AM15-R	CGGAGCTCGAATTCGGATCCTCATGAACAGTTCCCATTCTGACAAC
pUGT73AM16	AM16-F	ATCATCATCACAGCAGCGGCATGGCCACGGCAATGGC
	AM16-R	CGGAGCTCGAATTCGGATCCTCAACAGCTTCCATTCTCGCAAC
pUGT73AM17	AM17-F	ATCATCATCACAGCAGCGGCATGGCATCCCCTCACTTTCTTC

UGT constructs	Primers	Sequences (5’ to 3’)
	AM17-R	CGGAGCTCGAATTCGGATCCTCAGCAGTTTCCATTGTCACAAC
pUGT73AM18	AM18-F	ATCATCATCACAGCAGCGGCATGGCTTCCCCTCACTTTGTG
	AM18-R	CGGAGCTCGAATTCGGATCCTCATGATGATCCTCCATTGATGTGG
pUGT73AT6	AT6-F	ATCATCATCACAGCAGCGGCATGGCGCCCCACTTCC
	AT6-R	CGGAGCTCGAATTCGGATCCCTAAGCCGGAACAAAATGTTGTTG
pUGT73AT7	AT7-F	ATCATCATCACAGCAGCGGCATGGCTTCGGAAAACGCAGC
	AT7-R	CGGAGCTCGAATTCGGATCCCTAAATTTTATTCGGGCCAGAATGTCC
pUGT73AM28	AM28-F	ATCATCATCACAGCAGCGGCATGGCCATGGCTTCTCC
	AM28-R	CGGAGCTCGAATTCGGATCCTCAGCAGGAGCTTCCATTTTCAC
pUGT73AM29	AM29-F	ATCATCATCACAGCAGCGGCATGCCTTCCCCCCGAAG
	AM29-R	CGGAGCTCGAATTCGGATCCTCAGCAGCTTCCATTCTCAAATCC
pUGT73AT5	AT5-F	ATCATCATCACAGCAGCGGCATGGCTTCCCAACCCGAAG
	AT5-R	CGGAGCTCGAATTCGGATCCTCAAACAGGAGCAAAAGTGAATCCAG
pUGT73GB2	GB2-F	ATCATCATCACAGCAGCGGCATGGACGCTGCTCGGC
	GB2-R	CGGAGCTCGAATTCGGATCCTTACAATCGATGCCGATTTCTTTTCAC
pUGT73AM30^{L87V}	L87V-F	GCGAAAACGTGGATCTGCTCCCATCCCTG
	L87V-R	GAGCAGATCCACGTTTTCGCAGCCCTCCG
pUGT73AM30^{F100Y}	F100Y-F	AGCGCTAGCACCTTTTATCGCGCTACCTGTCTGC
	F100Y-R	AAAGGTGCTAGCGCTACCCA
pUGT73AM30^{M201L}	M201L-F	CGAGGAACTGGCGAAATTCGGTTATGAGATGG
	M201L-R	TTTCGCCAGTTCCTCGTCGATGCTTTTGG
pUGT73AM30^{A202V}	A202V-F	GGAAATGGTGAAATTCGGTTATGAGATGGGTG
	A202V-R	GAATTTCACCATTTCCTCGTCGATGCT
pUGT73AM30^{G205S}	G205S-F	GAAATTCTCCTATGAGATGGGTGAGGCAGA
	G205S-R	CTCATAGGAGAATTTCGCCATTTCCTCGT
pUGT73AM30^{swapVR1}	swapVR1-F	ATCACTTGCTGCTCTGTAGAATCTGGGTATGGAAGCCAGGGATGGGAGCAGATCC
	swapVR1-R	CAGAGCAGCAAGTGATCTCCTTTACGAACCGTCGGAGAAGTTGTTCG
pUGT73AM30^{swapVR2}	swapVR2-F	GTCTTCATCGGTGGATTTAGGTAGCTCAGATTTCAGGAATTCTACCGGAT
	swapVR2-R	ATCCACCGATGAAGACTTGGTGAAGTTTAGTTATGAGATGGGTGAGGCAG
pUGT73AM30$^{swap94-100}$	swap94-100-F	CTTCCATACCCAGATTCTACCGCGCTACCTGTCTGC
	swap94-100-R	TAGAATCTGGGTATGGAAGCCAGGGATGGGAGCAGATC
pUGT73AM30$^{swap101-108}$	swap101-108-F	CAGCAAGTGATCTCCTTTACGAACCGTCGGAGAAGTTGTTC
	swap101-108-R	AGGAGATCACTTGCTGCTCTGAAAAAGGTGCTAGCGCT
pUGT73AM19^{swapVR1}	swap92-107-F	GACAGGTAGCGCGGAAAAAGGTGCTAGCGCTACCAAGTGAAGGTAGCAAGTCCAC
	swap92-107-R	TTCCGCGCTACCTGTCTGCTATACGACCCATCTGAAAAACTGTTTGAGGA

Note: homologous arms are underlined.

Table S9. Statistical parameters for structural analysis of SgUGT73AM30.

Data ID: (UGT73AM30_Mogrol)	Overall (outer shell)
Wavelength	0.97918
Resolution range	26.48-2.6 (2.693-2.6)
Space group	P 32
Unit cell	105 105 122.661 90 90 120
Total reflections	265750 (26875)
Unique reflections	46443 (4639)
Multiplicity	5.7 (5.8)
Completeness (%)	99.84 (99.89)
Mean I/sigma(I)	9.31 (2.08)
Wilson B-factor	48.02
R-merge	0.1632 (1.528)
R-meas	0.1798 (1.681)
R-pim	0.07489 (0.6951)
CC1/2	0.994 (0.475)
CC*	0.999 (0.802)
Reflections used in refinement	46442 (4635)
Reflections used for R-free	2474 (203)
R-work	0.1884 (0.2756)
R-free	0.2448 (0.3338)
CC(work)	0.966 (0.728)
CC(free)	0.945 (0.543)
Number of non-hydrogen atoms	11586
macromolecules	11331
ligands	177
solvent	78
Protein residues	1437
RMS(bonds)	0.003
RMS(angles)	0.60
Ramachandran favored (%)	97.19
Ramachandran allowed (%)	2.60
Ramachandran outliers (%)	0.21
Rotamer outliers (%)	2.42
Clashscore	5.14
Average B-factor	58.39
macromolecules	58.46
ligands	59.68
solvent	44.90
Number of TLS groups	14

Note: Statistics for the highest-resolution shell are shown in parentheses.

Table S10. Compound information and corresponding identifiers used in this study.

Compound#	Name	CAS#	Formula	Supplier	Ref.
**1**	Mogrol	88930-15-8	$C_{30}H_{52}O_4$	ChemFaces	/
**2**	Mogroside IA1	88901-46-6	$C_{36}H_{62}O_9$	Yuanye	/
**3**	Mogrol 25-*O*-glucoside	/	$C_{36}H_{62}O_9$	/	/
**4**	Mogroside IIE	88901-38-6	$C_{42}H_{72}O_{14}$	Yuanye	/
**5**	Mogroside IIE 4′-*O*-glucoside	/	$C_{48}H_{82}O_{19}$	/	/
**6**	Mogroside III	130567-83-8	$C_{48}H_{82}O_{19}$	Yuanye	/
**7**	iso-Mogroside IVA*	2146088-08-4	$C_{54}H_{92}O_{24}$	/	/
**8**	Mogroside IIIA2	88901-43-3	$C_{48}H_{82}O_{19}$	ChemFaces	/
**9**	Mogroside IIIE	88901-37-5	$C_{48}H_{82}O_{19}$	ChemFaces	/
**10**	iso-Mogroside IVE*	2146088-06-2	$C_{54}H_{92}O_{24}$	/	/
**11**	Siamenoside I	126105-12-2	$C_{54}H_{92}O_{24}$	Yuanye	/
**12**	Mogroside IV	89590-95-4	$C_{54}H_{92}O_{24}$	Yuanye	/
**13**	Mogroside IVA	88901-41-1	$C_{54}H_{92}O_{24}$	ChemFaces	/
**14**	Hederagenin	465-99-6	$C_{30}H_{48}O_4$	ChemFaces	/
**15**	Hederagenin 28-*O*-glucoside	53931-25-2	$C_{36}H_{58}O_9$	/	[1]
**16**	Oleanolic acid	508-02-1	$C_{30}H_{48}O_3$	ChemFaces	/
**17**	Ursolic Acid	77-52-1	$C_{30}H_{48}O_3$	ChemFaces	/
**18**	Protopanaxadiol	30636-90-9	$C_{30}H_{52}O_3$	ChemFaces	/
**19**	Protopanaxatriol	34080-08-5	$C_{30}H_{52}O_4$	ChemFaces	/
**20**	Protopanaxatriol 3-*O*-glucoside	/	$C_{36}H_{62}O_9$	/	/
**21**	Ginsenoside F1	53963-43-2	$C_{36}H_{62}O_9$	ChemFaces	/
**22**	Ginsenoside Rh1	63223-86-9	$C_{36}H_{62}O_9$	ChemFaces	/
**23**	Ginsenoside CK	39262-14-1	$C_{36}H_{62}O_8$	ChemFaces	/
**24**	Ginsenoside F2	62025-49-4	$C_{42}H_{72}O_{13}$	ChemFaces	/
**25**	Ginsenoside F1 3-*O*-glucoside	/	$C_{42}H_{72}O_{14}$	/	/
**26**	Ginsenoside Rh1 3-*O*-glucoside*	/	$C_{42}H_{72}O_{14}$	/	/
**27**	Ginsenoside Rg1	22427-39-0	$C_{42}H_{72}O_{14}$	ChemFaces	/
**28**	Ginsenoside Rf	52286-58-5	$C_{42}H_{72}O_{14}$	ChemFaces	/
**29**	Ginsenoside Rh2	78214-33-2	$C_{36}H_{62}O_8$	ChemFaces	/
**30**	Ginsenoside Rh1 monoglucoside*	/	$C_{42}H_{72}O_{14}$	/	/
**31**	Quillaic acid	631-01-6	$C_{30}H_{46}O_5$	ChemFaces	/
**32**	Quillaic acid monoglucoside*	/	$C_{36}H_{56}O_{10}$	/	/
**33**	Momordicine I	91590-76-0	$C_{30}H_{48}O_4$	ChemFaces	/
**34**	Momordicine IV	894412-35-2	$C_{36}H_{58}O_9$	/	/
**35**	Momordicine II	91590-75-9	$C_{36}H_{58}O_9$	/	/
**36**	Cucurbitacin IIb	50298-90-3	$C_{30}H_{48}O_7$	ChemFaces	/
**37**	Cucurbitacin IIb 25-*O*-glucoside	/	$C_{36}H_{58}O_{12}$	/	/

**Compound#**	**Name**	**CAS#**	**Formula**	**Supplier**	**Ref.**
**38**	Mogroside IE1	88901-39-7	$C_{36}H_{62}O_9$	Yuanye	/
**39**	Mogroside IIA2	88901-45-5	$C_{42}H_{72}O_{14}$	ChemFaces	/
**40**	Mogroside IIA1	88901-44-4	$C_{42}H_{72}O_{14}$	ChemFaces	/
**41**	Cucurbitacin IIa	58546-34-2	$C_{32}H_{50}O_8$	ChemFaces	/
**42**	Cucurbitacin B	6199-67-3	$C_{32}H_{46}O_8$	ChemFaces	/
**43**	Cycloastragenol	78574-94-4	$C_{30}H_{50}O_5$	ChemFaces	/
**44**	Mogroside IIB*	942615-25-0	$C_{42}H_{72}O_{14}$	/	/
**45**	Mogroside IIA2 25-*O*-glucoside	/	$C_{48}H_{82}O_{19}$	/	/
**46**	Cycloastragenol 25-*O*-glucoside	86500-65-4	$C_{36}H_{60}O_{10}$	/	[2]

Note: The “Ref.” column denotes the source references of previously published spectroscopic data used for structural identification. An asterisk indicates that NMR data for the product is lacking.

Table S11. The amino acid sequences of UGT73s.

UGT73s	Sequences
BmUGT73GF5	MAVSANLHFVLFPLMAQGHMIPMVDIARLLAQRGVKITIFTTPHNASRFEGHLA<br>RLIGSGLHIKVIKLDFPCEEAGLPVGCENVDLLPSPDYALPFFTAANMLQKPAEV<br>LFQQLSPRPSCIISDMCLPYTMDIACKFDIPRLSFIGFGCFALFILHKLKLFPEILES<br>VSSEHEYFVIPGLPDKIEITKAQIPLQWKESFEEFGERINAAEMASHGVVVNSCEE<br>LEGKYAEEYKREKENKVWCIGPVSLCNKNILDMAERGRPGSIDGQYCLDWLDK<br>HESKSVIYVCLGSICNLTALQMMELGSGLEASNRPFIWVIREGKSSHELKILMEG<br>EKFEEGIEGKGLLIWGWAPQLLILSHPAVGGFLNHCGWNSTTEGIAAGVPMITW<br>PLFADQFLNEKVVEQILKIGVKVVSEKPTNPAFGIHTEMQVKREDVKKAVETLM<br>EEGEDGEGRRRRARELGEMVKMAGEEGGSSHLNLTSLIQEIMNHAGSKEHS*
BmUGT73GF6	MAVSANLHFVLFPLMAQGHMIPMVDIARLLAQRGVKITIFTTPHNASRFEGHLA<br>RLIGSGLHIKVIKLDFPCEEAGLPVGCENVDLLPSSDYALPFFTAANMLQKPAEV<br>LFQQLSPRPSCIISDMCLPYTMDIASKFDIPRLSFIGFGCFALFILHKLKFFQDVVE<br>SVSSEHEYFVIPGLPDKIEMTKAQIPGPWKEGFKEFRERINAAEMASHGVVVNS<br>CEELEGKYAEEYKREKENKVWCIGPVSHCNKNILDMAERGRPGSIDGQYCLDW<br>LDKHESKSVIYVCLGSLCNHNALQMLELGLGLEASNRPFVWVIRDGENSDELK<br>VLMESEKFEERIEGKGLLIWGWAPQLLILSHPAVGGFLTHCGWNSTTEGIAAGV<br>PMITWPLFAEQFLNEKVVEQILKIGVKVGAEKPTNPIFGIHAEMQVKREDVKKA<br>VETLMEEGGDGEGRRRRARELGEMVKMAGEEGGSSHLNLTSLIQEIMNHAGSK<br>EHS*
CsUGT73AM3	MDSHTHGTPHFLLFPFMAQGHMIPMIDLAKFLARRGAIVTIVTTPLNSARFHSVL<br>TRAIDSGHQIHVRELQFPSHQETGLPEGCENVDLLPSLASISQFYRAISLLHQPSE<br>KLFEQLTPRPNCIISDMCIPWTFDISQKFHVPRLVFYSLSCFFLLCMRSLTTNYEF<br>LNSNPDSEFLTLPGLPSQVEFRRSQIFTSTDDYLIQYSFRMWEVDRQSYGVIVNV<br>FEEMEPEHVTEYIKGRESPEKVWCVGPLSLSNDNELDKAERGNKAIIDGHECIK<br>WMDEQKPSSVVYVSLGSLCNLCTEQIKELGLGLVASNKPFIWVIRKANLTEALV<br>KWMDEYEFEEKTKGRGLVIRGWAPQVLILSHSAIGCFLTHCGWNSSVEGISAGV<br>PMITWPLFADQLYNHKFIVEILKVGVSVGEGTVGDLGGVQKVVVKREKVKEAI<br>EMVMDGDGSEERRKRCKEYGEKAKKAAEEGGSSHRNLNRLVEDITAHAFACG<br>NGNGSC*
CsUGT73AM7	MASTPHFLLFPFLAQGHIIPTLDLAKLLARRGAIVTILTTPHNATRNHSVLARAID<br>SGLQIHVVQIPFPCNKAGLPEGCENMDLLPSFRSVPTFFRSTFLLYDSSDELLQQL<br>CPPPTAIISDICLPWTLTLAQKYNIPRLVFYNLSCLYFLCLKDLEMKGPLIQSISDS<br>DTVTLVDGFKFRKAQLPKSVNEDMIAFIEEINKADRMSHGVIFNSFEELEPKNLA<br>EYKKIGELPDRVWCVGPVWLCNDDKLDRAYRGDRASIDENECSKWLDEQGPC<br>SVVYVALGSLCNLVTGQLIELGLGLEASNKPFIWVIRKGNLTEELLKWVEEYDF<br>EGKIKGRGVLIRGWAPQVLILSHPSIGCFLTHCGWNSSMEGITVGVPMITWPLFA<br>DQVFNQTLIVEILRIGVSLGVEEGVPWGEEEEKGIVVRKEKVKEAIEMVMEGEN<br>REELKKRCRELGEKAKMAVEEGGSSHRNLTLLIQDAQKNFEL*
CsUGT73AM8	MASTHILLFPFMAQGHMIPMFDLAKLLAHHGFIITIVTTPHNAHRYHSVLARAT<br>HSGLQIHVALLPFPSTQVGLPEGCENLDSLPPPPSSVSAFCRATYLLYEPSEKLFH<br>QLSPRPSCIISDMCLPWTLRLAQNHQIPRLVFYSLSCFFLLCMRSLKTNHSLVTSI<br>SDSEFLTLPDLPHPVEIRKSRLPTMKNEEMGKLSYDMAEADRVSHGVILNVFEE<br>MEAEYVAEYRKSRDSPQKVWCVGPVSLCNDNKLDKAERGEKSSIHEDECMKW<br>LNGQQPSSVVYVSMGSLCNLSTPQLIELGLGLEASKKPFIWAIRKGNLTDELQS<br>WIMEYNFEGKIEGWGLVIRGWAPQVAILSHSAIGSFLTHCGWNSSIEGISAGVP<br>MITWPLFADQVFNAKLIVEVLKVGVNVGEETALYWGEEKDKEVMVKREEVRE<br>AIEMVMNGENREEMKERAEKLAEMAKRAVEEGGSSHQNLKELVEELFKI*

UGT73s	Sequences
CsUGT73AM9	MASPPHFLLFPFMAQGHVIPMIDLAKLLAHRGVIITIVVTPTNAARNHSVLDRAI RSGLQIRMIQLPFPSKEGGLPEGCDNLDLLPSFKFASKFFRATSFLYQPSEDLFHQ LKPRPICIISDTYLPWTFQLSQKFQVPRLVYSTFSCFCFLCIHCLMTNPALSISDSD SVIFSDFTDPVEFRKSELPKSTDEDILKFTSEIIQTDAQSYGVIFNTFVEMEYNYIT DYRKTRQKSPEKVWCVGPVSLYNDDKLDLLERGGKASINQQECINWLDEQQPS SVIYVSLGSLCNLVTAQLIELGLGLEASNKPFIWSIREANLTEELMKWLEEYDLE GKTKGKGLVICGWAPQVLILTHSAIGCFLTHCGWNSSIEGISAGVPMITWPLFGD QIFNYKLIVDVLKVGVSVGVETLVNWGEEDEKGVYVKREMVREAIEMVLEGE KREEMRERSKKLAEIAKRGMEEGGSSYKDITMVIEDIIGNGGC*
CsUGT73AT1	MASSKSKQSLLLPHFLLFPVMAQGHQIPMAELAKLLSQSGVKTTLITTPQNATRI QSLLSQSPLTQIIQLPFPSHQQHLLQNCENFDSLPSLHLLPQFLTATSFLYSEIEHL FPQLSPKPCCIVSDMALPWTIQIAHKFNVPRLVFYSLSAFYLLFMATLRATDFGE KIMAASDYELISIPNFPDSIQVTKSQLVFTLDPVFLEWGNQMAKADRASYGFIM NSFNGLEPKYLEEFKKTIGSDKVWCIGPVSLCNKDTKDKAKRGNKAAIDEQEC MKWLDKQESESVIYAALGSICNVIAPQIIELGLALEASNKPFIWVIRQTKSTKKE VENWLAESEFEQRIKDRGLVIRGWAPQVLILSHPAVGGFVTHCGWNSTIEGISM GVPMVTWPLFSDQTFNEKLIVEVLRIGVSVGVEKCLRWGVEEEIGVQVKKEAIR GAIEKVMSGEGEEMRKRVRELAAIAKATMEEGGSSHLNLKRLIEEIMHQADSQ QSQN*
GpUGT73AM10	MASQCLEPHFVLFPLMAQGHMIPMVDLAKLLAQRGAIVTLVTTPHNAARYDG DLSRATDSGLRIRVAQLQFPCEEAGLPKGFENLDLFPPTSDAMIRFFKATYLLCD ASEKLLEDLSPRPSCIVSDMCLPWTMQVAKKHHIPRIAFSTFSFFFFSCMRSLVIN RDHINSLSDSEYVTFSDLPDPIELQKSQIPQTLDEEMLKFSHEMREVDRLSYGVII NTFEEMEPKYAEEYKKVRESDKVWCIGPVSLCNKEKLDKAQRGNNASIDEYEC INWLDKQQPCSVIHVSLGSLCNLLTPQLIELGLGLEASNKPFIWVIRQGNMTREL EKWLLEYGFEERTKGRGLVIRGWAPQVLILSHRSIGGFLTHCGWNSTIEGISAGV PMITWPLFADQIFNEKLITQILRIGISVGVDTIMAWGEEEKKGVLVKKENVKEAI DSLMDGDDRNERRERCKKLAEAANRAVEEGGSSHHNLTLLIEDVIHHQAGCVN GTC*
GpUGT73AM11	MASQNHKPHFVLFPFMAQGHLIPMVDLAKLLAQQGAIVTIVNTPHNAARYDAV LARAVVSGLQIRVALLEFPCEEAGLPKGSENMDLLPTLDSMLKLFKATFLLCDA SDKLIEDLSPRPSCIVSDMCLPWTMQVDKKHHIPRIVFYSFSFFFLLCMRSLVVN SDHIKSIPDSEYVTLSDVPDQVKFRKSQLPQTPNEEMEKLSYEMGEADRLSYGVI INTFEEMEPRYVEEYKKVRESEKVWSIGPVSLCNKENLDKAQRGNKASIDEHEC INWLDKQQPCSVIHVSFGSLCNLLTPQLIELGLGLEASNKPFIWVIRQGNLKEELE SWLLEYGFEEKTKGRGLVIRGWAPQVLILSHPSIGGFLTHCGWNSTIEGITAGVP MITWPLFADQIFNEKLITQILRIGISVGVDTVLPFGEEEKRGVLVKKENVKEAIDS LMDGDDREERRERCKKLAEVANRAVEEGGSSHHNLTLLIEDFIHQTSCDNGTF*
GpUGT73AM12	MASQHYKPHFVLFPYMAPGHMIPMIDLAKLLAQQGAIVTIVNTPHNAARFDDV LARAVDSGLQIQVALLQFPCEEAGLPKGSENVDLLPSLDSQIKLFKATILLSDAS EKLLEDLSPKPSCIVSDFCLRWTMQVAKKHHIPRIVFYCLSFFSLLSLRSLVINRD HIKSLSDSEYVTLSDMPDQVKFQKSQLRQPVDEEMGKLAYDMGEADRLSYGVI VNTFEEMEPRYVEEYKKVRESDKVWCIGPVSLCNKEKLDKAQRGNKASIDEHE CINWLDKQQPCSVIHVSLGSLCNLQTPQLIELGLGLEASNKPFIWVIRQGNLKEE LESWLLEYGFEEKTKGRGLVIRGWAPQLLILPHPSIGGFLTHCGWNSTLEGITAC VPMITWPLFADQIFNEKLITQILRIGISIGVDTFVAWGGEEEKGDFVKKENVKEAI DSLMDGDDREERRGRCKKLAEVANRAVEEGGSSLQNLTLLIEDFTHQASCD*

UGT73s	Sequences
GpUGT73AM13	MASQHYKPHFVLFPYMAPGHMIPMIDLAKLLAQQGAIVTIVNTPHNAARFDDV<br>LARAVDSGLQIQVALLQFPCEEAGLPKGSENLDLLPSLDSHIKLFKATILLSDASE<br>KLLEDLSPKPSCIVSDFCLQWTMQVAKKHHIPRIVFYSLSFFYLLSIRSLVINCDHI<br>KSLSDSEYVTLSDMPDQVEFQKSQLPQPVDEEIGKLTYDMGEADRLSYGVIVNT<br>FEEMEPRYVEEYKKVRESDKVWCIGPVSLCNKEKLDKAQRGNKASIDEHECIN<br>WLDKQQPCSVIHVSLGSICNLQTPQLIELGLGLEASNKPFIWVIRQGNLKEDLES<br>WLLEYGFEEKTKGRGLVIRGWAPQVLILPHPSIGGFLTHCGWNSTIEGISAGVPM<br>ITWPLFADQIFNEKLITQILRIGISIGVDTFVAWGGEEEKGDFVKKENVKEAIDSL<br>MDGDDREERRGRCKKLAEVANRAVEEGGSSLQNLTLLIEDFTHQASCD*
GpUGT73AM14	MASLHHKPHFVLFPFMAPGHIIPMVDLSKLLAQRGAIVTIVDTPNNAARYDAVL<br>ARAIDSGLQIRVALLQFPCGEAGLPKGSENLDLLPSLDSMQKLFKATVLLCDAS<br>EKLLEDLRPRPSCIVSDMCLPWTIQVAKKHHIPRIGFYSLSFFFLMCMQSLAKNR<br>DHIKSIPDSEYVTLSDVPDQVEFRKSQLPQTPYEEMEKLSYEMGEADRLSYGVIV<br>NTFEEMEPRYVEEYKKVRESDKVWSIGPVSLCNKENLDKAQRGNKASIDEHECI<br>NWLDKQQPCSVIHVSFGSLCNLLTPQLIELGLGLEASNKPFIWVIRQGNLKEELE<br>SWLLEYGFEEKTKGRGLVIRGWAPQVLILSHPSIGGFLTHCGWNSTIEGITAGVP<br>MITWPLFADQIFNEKLITQILRIGISVGVDTVLPLGEEEKRGVLVKKENVKEAIDS<br>LMDGDDRKERRERCKKLAEVANRVVEEGGSSHHNLTLLIEDFSHQTSCDNGTC<br>*
GpUGT73FX1	MASISHSPKPHFILFPLMAQGHLIPMLDLAKLLAHQGVIITFVTTPHNAARFEQV<br>LKRAIDSGLHIQIVKLPFRCEEVGLPKGCENFDLLPSLGLAPNFVQATLSMRQAT<br>DKLFEELSPRPTCLISDMGFPWTVEISLKFHIPRFVFCSMSCFYLLSLLNLRENRA<br>LLDSIASESEYFLIPNLPDRIRITKSQINYVPNVLFSKVRNEVAKADLASYGVIVN<br>TFEDLDTEYVEEYKRAVGPERVWCVGPVSLCNKDILDKFQRGNNASIDGFECLE<br>WLDKQQPCSVVYAALGSFCNVILPQFVELGLALEESNKPFIWVIRQGKVLRELE<br>KWISEYGFEEKTKGRGLVIHGWAPQILILSHPAIGGFITHCGWNSTIEAISAGVPLI<br>TWPLFGDQFFNEKLIVQVLKIGIRIGVEKCVMWGDEEKIGVMVKKENIRDALHS<br>LMNGEERDEIRERTKKLSEKAKRAVEEGGSSHLNMKQLIADIVHIGAGWSRPNF<br>*
McUGT73AM15	MAMASPHFVLFPFMAQGHMIPMIDLAKLLARRGAIISFITTPRNAARNYSVLAR<br>AINSGLRIHVHQLDFPSNQVGLPEGCENLDLLPSYDDHLTSKFFQATFLLREPWE<br>KLVEQLIPRPTCIITDTGFPWTLPLSLRLHIPRLVYYTHSCLYLLIMRSIQSRQPLL<br>ASISDDSEYVTLSDLPHAVQFLKSQLSSVADGDAAKFLEAMEEADAHSHGVVIN<br>IFEEMEAENVAAYRKHRKSPDRVWCVGPVSLCSEDKLDMAQRGNRACIDEAQ<br>CMNWLDAQRPSSVVYVSLGSLCNLSTAQLIEMGSGLAESKKPFIWVVRKEYAT<br>EEFQKWATEYDLEGKTEGRGLVIQGWAPQVLILTHSAIGCFLTHCGWNSCTEAI<br>SAGVPMITWPIFGDQNFNKKLIVEILRIGVGVGVETAVDWAGKAEKRALVKRD<br>KVREAIEMAMDGDGSEEMRERCKQLAEKAKRALQEGGSSHLNLKLLIQDIVAY<br>GTGCQNGNCS*
McUGT73AM16	MATAMASPHFLIFPFMAQGHMIPMMDLANLLAHQGAIVTIVTTPHNAARYHSV<br>LTRAIGSGSQIRVVQLEFPCHEAGLPHGCENLDLLPSLSSMSTFMKATYLLHDPS<br>EKLLPQLSPRPTCIISDMCLPWTLTLAHKFRVPRLVLYSLSCFFLLCIHSVKYHIPS<br>FSSISDSELVDFSGLPHPVQFRKSQLPKATDEAMSKFGYEMGEADRQSHGVIINT<br>FEEMEPEYLAEYRKLRELPEKVWCVGPLLLYNDNKLDVAQRGNTAAIDENECI<br>NWLDGQRPCSVVYVSLGSLCNLTTPQLIELGLGLEASNKPFIWVIRKLNLTKELL<br>DWMEEYDFEGRTKGRGLVIRGWAPQVMILSHSSTGCFLTHCGWNSSIEGMSAG<br>VPMITWPLFADQVFNEKLLVEIVRIGVSVGAETAVPWGEEEKIGVLVKRESVRE<br>AIEMAMDGDGSEEMRQRCKQLAEKAKRAVQEGGSSHRNLKLLIEEIVDDGRSC<br>ENGSC*

UGT73s	Sequences
McUGT73AM17	MASPHFLLFPFMAQGHMIPMIDLAKLLAHQGAIVTIVTTPHNAARCHSVLTRAI<br>HSGLQIRVLQLEFPCHEVGLPHGCENLDLLPSLSSSSAFHKATCLLHDQSEKLFP<br>QLSPRPTCIVSDMCLPWTLTLAHKFRLPRLVLYSLSCFSLLCMHTLITQLPLLSSF<br>SDSELVTFSDLPHPVQFRKSQLPIATDEEMAKLGYDMMEANRQSHGVIINTFEE<br>MEHEHLAEYRKLREFPERVWCVGPVSLCNNDKLDVAQRGNTAAIDVHECVNW<br>LDGQRPCSVVYVSLGSLCNLTTPQLIELGLGLEASNKPFIWVIRKLNLTKEMLD<br>WMEEYDFEGRTKRRGLVIRGWAPQVMILSHSSTGCFLTHCGWNSSIEGMSAGV<br>PMITWPLFADQVFNERLLVEIIRIGVSVGAETAVPWGEEEKIGVLVKRESVREAI<br>EMAMDGDGSEEMRQRCKQLAEKAKRTVKEGGSSHRNLNLLIQDTVAQGRSCD<br>NGNC*
McUGT73AM18	MASPHFVLFPYMAQGHMIPMIDLAKLLAQRGAIVTLVTTPKNAARNDSVLARA<br>IESGLQIKVVQLEFPWNQAGLPEGCENLDLLPSLASISKFNAAASLLYEPSVQLL<br>GNLSPRPACVVSDMNLPWTLRLARHHRLPRLVFYTFSCFFLLSLRSFAKTHTLLS<br>STSDEELVKFGVEMMVADQHSDGVIFSGFEEMEPEQIAEYRNERKSPEKVWCV<br>GPVSLCNDDKLDVAQRGNRASIDVHECVNWMDGQHQSSVVYISLGSLCNVDA<br>SQLIELGLGLEASNKPFIWVIRELNLTEELLNWLEEYDLVGRTKGRGLVIRGWA<br>PQMMILSHSAIGCFLTHCGWNSSMEGVSAGVPMITWPLFADQMSNGKLIVEILK<br>IGVSVGVDTSVPWGEEEKRGVLVRREKVREAIEMAMDGEGSEEMRQRCKQLA<br>EKAKRAVQEGGSSNRNLTLLMDHINGGSS*
McUGT73AT6	MAPHFLLVPIMAQGHLIPMADFAKLLAEQGVKVTLVTTPQNVPRFQSLFSKPAL<br>QIQIQIINFPSQNGIEYENFDSLPSLGLSAKLLAETSLLRGPTEELFERLSPRPSCLV<br>SDMALPWTGHVARKFNVPRLVFYSLSCFYLRVMITLRTSGVVRKILAAPESEPI<br>ALPNLPDPVQVTRSKSIFSLDPLFVDWTGQLAKADHESHGFALNSFHDLEPKYL<br>QEFKNAIGSDKVWYIGPVNLCNKDIMDKVQRGNKSAIGERECLEWLDKQDPGS<br>VIYAALGSQCNVVTTQIVELGLALQASNRPFIWVIRQTNATHELEKWLSEHNFE<br>GKTNGRGLVVRGWAPQVLILSHRATGAFLTHCGWNSTVEAISAGIPMVAWPLF<br>ADQVFNEMLIVQILKIGASVGVEKCYTWGVEEEIGVQVKKEVVIDALEKVMDG<br>EDKKGMRRRAKEFAERAKAAMEDGGSSQLNIKLLIEDIMHQQHFVPA*
McUGT73AT7	MASENAAAAAPHFLLLPIMAQGHLIPMADFAKLLAEQGVKVTLVTTPQNAPRF<br>QSLLSKPALQIQIQTISFPPENGIQCENFDSLPSLELGAKFFADTSLLRGPTEELFQ<br>RLSPRPSCLVSDMALPWTGGIARKFNVPRLVFYSLSCFYLRVITTLRTTTDVVYK<br>IMAAADLEPVTIPDLPDPVQITKAKLIFSLDPIFMDWSAQMAKADHESYGFVLNS<br>FQDLEPKYLSEFKKVTGSKKVWCVGPVSLCNKDTMDKTHRGDKSAIGERECLE<br>WLDKQDPSSVIYAALGSTCNVATSQMIELGLALQASNRPFIWVIRQTNATQGLE<br>KWLSEHNFEDKANGRGLVVRGWAPQVLILSHPAIGAFVTHCGWNSTIEAIATG<br>VPMVAWPLFADQMFNEMLIVQILKIGASVGVEKCYAWGAEEEIGVQVKKEAVI<br>DAIEKVMDGEDKEGMRRRVREFAERAKAATEDGGSSHLNVKLLIKDIMDQGH<br>SGPNKI*
SgUGT73AM1	MLMHALTPTAPFFSIKPNTEPPSATTRQPPMDSPPQKPHFLLFPFMAQGHMIPMI<br>DLAKLLAQRGAIITVVTTPHNAARYHSVLARAIDSGLHIHVLQLQFPCNEGGLPE<br>GCENFDLLPSLGSASTFFRATFLLYEPSEKVFEELIPRPTCIISDMCLPWTVRLAQ<br>KYHVPRLVFYSLSCFFLLCMRSLKNNQALISSKSDSELVTFSDLPDPVEFLKSQL<br>PKSNDEEMAKFGYEIGEADRQSHGVIVNVFEEMEPKYLAEYRKERESPEKVWC<br>VGPVSLCNDNKLDKAQRGNKASIDERECIEWLDGQQPSSVVYVSLGSLCNLVT<br>AQLIELGLGLEASNKPFIWVIRKGNITEELQKWLVEYDFEEKTKGRGLVILGWA<br>PQVLILSHPAIGCFLTHCGWNSSIEGISAGMPMITWPLFADQVFNEKLIVEILRIG<br>VSVGMETAMHWGEEEEKGVVVKREKVREAIERAMDGDEREERRERCKELAE<br>MAKRAVEEGGSSHRNLTLLTEDILVNGGGQERMDDADDFPTIVN*

UGT73s	Sequences
SgUGT73AM2	MDSPPHRPHFLLFPFMAQGHMIPMIDLAKLLAQRGAIVTILTTPHNAARTHSVL<br>ARAIDSGLQIRVRPLQFPCKEAGLPEGCENLDLLPSLGSASTFFRATCLLYDPSEK<br>LFEELSPRPTCIISDMCLPWTIRLAQKYHVPRLVFYSLSCFFLLCMRSLKNNPALI<br>SSKSDSEFVTFSDLPDPVEFLKSELPKSTDEDLVKFSYEMGEADRKSYGVILNIFE<br>EMEPKYLAEYGNERESPEKVWCVGPVSLCNDNKLDKAQRGNKASIDERECIK<br>WLGGQQPSSVVYASLGSLCNLVTAQFIELGLGLEASNKPFIWVIRKGNITEELQK<br>WLVEYDFEEKTKGRGLVILGWAPQVLILSHPSIGCFLTHCGWNSSIEGISAGVPM<br>VTWPLFSDQVFNEKLIVQILRIGVSVGAETAMNWGEEEEKGVVVKREKVREAIE<br>RMMDGDEREERRERCKELAETAKRAIEEGGSSHRNLTLLIEDIGTSLRRL*
SgUGT73AM19	MASPRHTPHFLLFPFMAQGHMIPMIDLARLLAQRGVIITIITTPHNAARYHSVLA<br>RAIDSGLHIHVLQLQFPCKEGGLPEGCENVDLLPSLASIPRFYRAASDLLYEPSEK<br>LFEELIPRPTCIISDMCLPWTMRIALKYHVPRLVFYSLSCFFLLCMRSLKNNLALI<br>SSKSDSEFVTFSDLPDPVEFLKSELPKSTDEDLVKFSYEMGEADRQSYGVILNLF<br>EEMEPKYLAEYEKERESPERVWCVGPVSLCNDNKLDKAERGNKASIDEYKCIR<br>WLDGQQPSSVVYVSLGSLCNLVTAQIIELGLGLEASKKPFIWVIRRGNITEELQK<br>WLVEYDFEEKIKGRGLVILGWAPQVLILSHPAIGCFLTHCGWNSSIEGISAGVPM<br>VTWPLFADQVFNEKLIVQILRIGVSVGTETTMNWGEEEEKGVVVKREKVREAIE<br>IVMDGDEREERRERCKELAETAKRAIEEGGSSHRNLTMLIEDIIHGGGLSYEKGS<br>CR*
SgUGT73AM20	MDSPRHKPHFLLFPFMAQGHMIPMIDLAKLLAQRGAIITILTTPRNAARYHSVLA<br>RAIDSGLHIHVLQLQFPCNEGGLPEGCENFDLLPSLGSAPTFFRATFLLYDPSEKL<br>FEELIPRPTCIISDMCLPWTIRLAQKYHVPRLVFYSLSCFFLLCLGSLGSKNALVS<br>FMSDSEFVTLPDLPDQFEFRKALLPKSTDEDMAMFNYELMEADRMSHGVILNT<br>FEEMEPKYLAEYKKIRESPEKVWCVGPVSLCNDNKLDKAQRGDKASIDEHECI<br>KWLDGQQPSSVVYVSMGSLCKLVTAQLIELGLGLEASKKPFIWVIRKGNITEEL<br>QKWVVEYDFEEKTRGRGLLILGWAPQVLILPHPAIGCFLTHCGWNSCIEGISAG<br>VPMVTWPLFADQVFSEKLIVQILRLDVSVGVETAMHWGEEEEKEVVVRREKVR<br>EAIEIVMDGDEREERRERCKELAETTKRAAEEGGLVSSKPHTAD*
SgUGT73AM21	MDSSPQKPHFLLFPFMAQGHMIPMIDLAKLLAQRGAIITIVTTPDNASRTHSVLA<br>RAIDSGLQIRVRPLQFPCKEAGLPEGCENLDLLPSLGSASTFFRATCLLYDPSEKL<br>FEELIPRPTCIISDMCLPWTIRLAQKYHVPRLVFYSLSCFFLLCMRSLKNNQALIS<br>SKSDSELVTFSDLPDPVEFLKSQLPKSIDEEMAKFGYEMGEADRQSHGVIVNAF<br>EEMEPKYLAEYRKERESPEKVWCVGPVSFCNDNKLDKAQRGNKASIDERECIE<br>WLDGQQPSSVVYVSLGSLCNLVTAQLIELGLGLEASNKLFIWVIRKGNITEELQ<br>KWLAEYDFEEKTKGRGLVIRGWAPQLLILSHPAIGCFLTHCGWNSSIEGISAGVP<br>MITWPLFTDQVFNEKLIVEILRIGVSVGMETAMHWGEEEEKGVVVKREKVREAI<br>ERLMDGDARDERRERCKNFGEMAKRAVKEEGSSHRNLTLLIEDIVRGGGCDC*
SgUGT73AM22	MDSPPQKPHFLLFPFMAQGHMIPMIDLAKLLAQRGAIITVSTTPHNAARYHSVL<br>ARAIDSGLHIHVLQLRFPCKEGGLPEGCENIDLLPSHYSPFTFFSATFLLYEPSEK<br>VFEELIPRPTCIISDISLPWTIRLAHKYHVPRLVFYSLSCFFLLCMRSLRNNQALIS<br>SKSDSELVTFSDLPDPVEFLKSQLPKSNDEEMAKFGYEIGEADRQSHGVIVNVFE<br>EMEPKHLAEYRKERESPEKVWCVGPVSLCYDNKLDKAQRGNKASIDERECIEW<br>LDGQQPSSVVYVSLGSQCNLVTAQLIELGLGLEASKKPFIWVIRKAIITEELQKW<br>LVEYDFEEKTKGRSLLIRGWAPQVLILSHPALGCFLTHCGWNSSIEGISAGVPMV<br>TLPLFGDQIFNETLIVQILRIGVRVGVEAAMQCGEEEEIDRGVVVRREKVREAIGI<br>VMDGDEREGRRERCKELAETAKRAVEEGGSSHQNLTRLIEDIASSLRRLSEWKF<br>YLIIIS*

UGT73s	Sequences
SgUGT73AM30	MASLSPPQKPHFLLFPFMAQGHMIPMIDLAKLLAQRGAIITVATTPHNAARTHS<br>VLARAIDSGLQIRVRPLQFPCKEAGLPEGCENLDLLPSLGSASTFFRATCLLYDPS<br>EKLFEELIPRPTYIISDMCLPWTIRLAQKYHVPRLVFYSLSCFFLLCMRSLKNNQA<br>LISSKSDSELVTFSDLPDPVEFLKSQLPKSIDEEMAKFGYEMGEADRQSHGVIVN<br>AFEEMEPKYLAEYRKERESPEKVWCVGPVSLCNDNKLDKAQRGNKASIDEREC<br>IKWLDGQQPSSVAYVSLGSLCNLVAAQLIELGLGLEASNKPFIWVIRKGNITEEL<br>QKWLVEYDFEEKTKGRGLVIRGWAPQVLILSHPAIGCFLTHCGWNSSIEGISAG<br>VPMITWPLFADQVFNEKLIVEILRIGVSVGMETAMHWGEEEEKGVVVKREKVR<br>EAIERVMDGDEREERRERCKELAEKAKRAVEEGGSSHRNLTLLTEDIIVNGGGC<br>ENGRCH*
SgUGT73AT4	MASESKPPHFLLFPVMAQGHLIPMVDLAKLLAQRGVKITLITTPQNASRIDSFLA<br>RATQSGLQIQTLQLQFPSERAGLPEGCENFDSLPSLDLIPKFLTLTTLLCRATEEL<br>FEQLSPRPSCIVSDMALPWTIQLADKFKVPRLVLYSLSCLYLLVMANLQAFNGE<br>KMLPTSESEKFVVPNLPDRVEVTKSQLVFTLDPDFLQWGAEMVKADHASYGFI<br>FNTFDGLEPKYLEGYKKAIGSDKVWCVGPVSLCHKDTADMAVRGNKAAIDQH<br>ECLQWLDRQETGSVVYAALGSICNPIAAQIMELGLALEASKRPFIWVIRQAKAS<br>RELEKWLSEYGFEEKTKGRGVVIHGWAPQVLILSHPAIGAFVTHCGWNSTIEGIS<br>AGVPMVAWPLFADQILNEKVIVQVLRVGVSVGVEKCFRWGAEEEVGVQVKKE<br>AIGDAIEKLMGGEGKEEMRRRVRDLAEKAKAAMEEGGSSHLNVKMLIDDIMH<br>HSSSRF*
SgUGT73GB1	MGAAQPHFVLMPLMSPGHLIPMIDMAKLLAQHGVAVSIVTSPLNALRFKSTVR<br>RSAESGLCINLLTIPFPWQEAGVPEGCENVDSLPCRSLIRNFYLASTLMQRPFEQL<br>LEALHPPPSCLVSGKNLAWTVETARKFRIPRIFFDGMGCFSFACSHKIEASKVHK<br>AIAKSSDSFLVPDIPHRIELTQSKLPENLDGASDMKDVLDSYKAGDVVADGILV<br>NTFEELEGEYIGEFKKVKGDKVWCVGPVSACNTLHSDKFERGKTASINETECVG<br>WLDSWDRQSVVYACLGSICGLKPWQLMELGLGLEASNRPFIWVIGGGEKTQDL<br>QKWVDEGGFTERTKERGLLVWGWAPQVSILSHPAIGAFLTHCGWNSVLEGVT<br>AGVPMITCPLFAEQFLNEQLIERVLGIGVSLGVETCVKWGMEEKAGTVIKREDV<br>KKAIEKIMDQTEEAENRGKKARELGEMAKKSIVKGGSSYRGIDKFIEYVVETKQ<br>RRSKLVHQPPPPPPTTTTTGSNILRT*
TpUGT73AM28	MAMASPRHQPHFLLFPFMAQGHMIPMIDLAKLLAQRGVLCTIVTTPQNAARFD<br>SVLARATHSGLQIRVSQLQFPWEKAGLPQGCENLDLLPSLGYGTTFFQATYLLY<br>DQSEKLFEELSPRPTCIVSDMCLPWTIRLSQKYNVPRLVLYSLSCFFLLCLRSMK<br>TNRDLISSMSDSEFVTFSDLPDRVEFQKSELPKATDEGMAKFAYDMVDADRDSF<br>GVILNIFEEMEPKYLAEYRKERKSPEKVWCVGPVSLCNEQRLDKAQRGNKASID<br>ENECTKWLDGQQPSSVVYVSLGSLCNLLPAQLIQLGLGLEASKKPFIWVIRKGN<br>LTEELQKWLVEYDFEEKTKGRGLVIRGWAPQVLILSHPASGCFLTHCGWNSSV<br>EGLSAGVPMITWPLFADQVFNEKLIVQILRIGITVSAETSLQWGEEENRGVVVKS<br>EEVREGIEMVMDGEESEERGGRCKQLAEMAKRAVEEGGSSHRNLTLLIEDIVN<br>GSGCENGSSC*
TpUGT73AM29	MPSPRSKPHFLLFPFMAQGHMIPMIDLAKLLDQRGAICTIVTTPLNAARFDSALA<br>RAIHSGLQIHVSQLQFPWEEAGLPDGCENLDLLPSMDYNISFFRATNLLYNPSEK<br>LFGELSPRPTCIISDMCLPWTLRLSQKYHIPRLVFYSLSFFFLLCMRSMKTNRDLI<br>NSMSDSEFVTFSDLPDQVEFRKSELPKFTDEEMVKFVADMREADMESLGVILNI<br>FEEMEPKYLAQYRKERKFPDKVWCVGPVSICNENRLDKAQRGNKAFIDEHECT<br>EWLDGQQPSSVVYVSLGSLCNLLPAQLIELGLGLEASKKPFIWVIRKGNLTEELQ<br>KWLVEYGFEEKTKGRGLVIRGWAPQVLILSHPAIGCFLTHCGWNSSIEGLSAGV<br>PMITWPLFADQVFNEKLIVQILRVGISVSVETEEKEGVVEKREKVREAIEMVMD<br>GDEREERRERCKQFAEMAKRAVEEGGSSHRNLTLLIEDIVNGSGFENGSC*

UGT73s	Sequences
TpUGT73AT5	MASQPEAPHFLLFPVMAQGHMIPMVDLAKLLAQSGVKVTLVTTPQNASRFHTV<br>LARATQSGLQIQTLQLQFPAQQAGLPEGCENFDSLPSLGMIPKFLTVTSLLGRAA<br>EELFEDLIPRPSCIVSDMALPWTIQIAHKFNVPRIVFYSLSYLYLLVMANLRALD<br>VGTMLASSGSEPIIMPNLPDRVEVTKSQLIFTLDPDFLEWGAEMAKADHASYGFI<br>VNSYDELEPKYLKAYKKAIGSDKVWCVGPVCLCNKETVDMAERGNKAAIDEY<br>ECTEWLDRQEPGSVVYAALGSICNAVAAQIIELGLGLEASNRPFIWVIRQANASE<br>EIENWISEYGFEEKTKGRGVVIRGWSPQVLILSHPAIGGFITHCGWNSTIEGISAG<br>VPMVAWPLFADQIFNEKLIVQVLKVGVSVGVEKSFIWGGEEEIGVQVKKEAIRD<br>AVERLMEGEGSEEMRRRVKDLAKRAKTAMEEGGSSHLNIKLLIEDVMHLTDSG<br>KAPSSSGFTFAPV*
TpUGT73GB2	MDAARPHFVLFPLMSPGHLIPMIDMAKLLAQHGVAVSIVTSPLNALRFESTVVYR<br>AAESGLCIDLLTIPFPWLEAGVPEGCENIDSLPSRSLIRNFFLASTLMQRPFEQLLE<br>VLHPPPSCIVSGKNLAWTAETASKFQIPRFFFDGMGCFASACSHKIEVSGVHKTV<br>ANSSDTFLVPGLPHKIELTKSKLPENLDGASDMKDVLNSSLVGDFGADGILVNT<br>FEELESEYIREYKKDKGDKVWCVGPVSACNTLHADKFERGKTASINETECLGW<br>LDPWERQSVVYACLGSICGLKSWQLIELGLGLEASNKPFIWVIGGGEKTEDLRK<br>WVDENGFTERTRERGLLVWGWAPQVSILSHPAVGAFLTHCGWNSALEGITAG<br>VPTLTCPLFSEQFLNEQLIEQVLGIGVRLGVESCVKWGMEEKAGTIIKREKVKN<br>AIEKILDHTDEAGTRRKKAREVGEMARRAIAKGGSSYDDMDKFIAYVVKRNRH<br>RL*
VvUGT73CK9	MASQLLQLHFVLIPFMAPGHLIPMVDMARLLAQHGVIVTVVTTPLNATRFKSMI<br>DRAVESGLQIHLLELQFPAVEAGLPEGCENVDLLPSRSLIRNFFVAASMLQQPLE<br>QLFQELQPRPSCIISGKNLAWTADTARKFQIPRLYFDAMSCFAFSCSHNLEASKV<br>HESISKLETFLVPGLPDQIELTKAQLPESLNPDSSDLTGILNQMRASESIADGIVV<br>NTYEELEPRYVKEYKRIKGDKVWCIGPVSACNKLNLDKAERGKKALVDENQC<br>LRWLDSWEPNSVVYACLGSISGLTALQLIELGLGLEASNRPFIWVIRGGEKSKEL<br>ERWILEEGFEERTEGRGLLIRGWAPQMLILSHPSIGVFLTHCGWNSTLEGVCTGV<br>PILTCPLFAEQFINEKLVVQILGIGVSVGVESAVTWGMEEKFGVVMKREDVMK<br>AIDEVMDKGEGGEKRRKRARELGEMAKKAIEEGGSSYLNMKRLIHYILQQTIG<br>NPSTQLS*
PsUGT73FA6	MALQEHNQLHFVLFPPMAQGHLIPTIDMARLFAERGITVTIITTPLNTFRFKTIID<br>RAVDSGFSIRLLQLRFPSVEAGLPEGCENVDSLPSREMVSNFFEATSVLQEPLEQ<br>LLGVLKPVPSCIISAIAFPWTAETARKLQIPRLVFHGTCCFSLLCCHNILEYKVAD<br>SITSESESFVIPGLPDRIEIIKAMLPGNLPQSSGDLKNFRDQINAAELTAHGVVVNS<br>FNELEPMYAEEYLKAKFGKVWCTGPVSLCNKETLDKAERGNKASVDESQCLK<br>WLDLKDPGSVLYVCLGSLCRLTTSEMIELGLGLEASKHPFIWVIRGRDKYLDLE<br>KWLLEEKFEERNKDRGLVIKGWAPQILILSHKAIGGFLTHCGWNSVLEGVCAG<br>VPMITWPMGEEQFFNEKLVVQVLGIGVKIRGDDIHASDATKPMELMEEHRKFG<br>SDENKREEVEKAVNRVMDGGGEGEERRRKVRELGERAKKAMEGGGSSQLNM<br>TLFIQDIQQQGPKQG*
BvUGT73BG6	MASTDQIHVIVIPLMSQSHLIPMIDLSKILAKRGLIITIITTPLNAARYQATVDRAIR<br>SGRLRIKLVPLPFPCNEAGLPEGCENLDSLPSPDLQTKFLVSSSLLQAPLESHLEK<br>VDPRPSCIISSLPWTKDVAYKFNIPRFAYQGISCFTLLCSHKMMLHKVHENVKY<br>DNEPFLVPDVPDKIEFTYAQLPKMMRTSSSHGDDGIDLKDIRHQFKQAEISAEGL<br>LVNTFEELELDYVKSYQKVAMGNVLCVGALSLCNRMVKDYEEMTSIDEHYCL<br>KWLDTMKPKSVIYVCFGSLCHLLPKQLIEIGKGLEASNSPFIWIIREVDYNEEIKK<br>WVKNENFEDRLKGKGLIIKGWAPQVLILSHPATGGFMTHCGWNSTLEGVSAGV<br>PMITWPMFAEQFYNEKLIVQVLKIGIRIGVEVTMQWGEEANDGIYVKRESVKD<br>VVDQLMDEGEKGQERRRKAQELGERANQAVEEGGSSYMNITSFIEHVKQWCN<br>LN*

UGT73s	Sequences
JrUGT73AB29	MASMITSQPHFVLIPLMAQGHMIPMIDMARLFAEHGVIVSLVTTPYNASRFETTIHRATGSGLPILLVQLEFPCKQVGLPAGYENLDILPSRDLLTKFYEGLSMLQQPLEKHLQNQRHPPTCIISDKCLSWTSNTAQKFNIPRLVFHGMGCFSLLSSHNIKFYNAHRSISSDSEPFVIPGLPQRILITRAQLPGSFVTLPGLDDVRDKMQEAESTAYGVVVNTFNELEQGIVEEYGKAIKKKVWCVGPVSLYNKESLDKFERGDKTLIDEQQCLEWLNSMEPSSVIYTCLGSLCRLVPSQIIELGLGLEASKQPFIWVIKTGERYLELEEWLENERFEERNKGRGLLIKGWAPQDFILSHPAIGGFITHCGWNSTIESVCHGVPMITWPLFSEQFFNEKLVVEILRIGIRVGVEIPVRWGEEEKVGVLVKKEKVEKAIALLMDGGDEGVRRRKKARELGEMARRAMEKGGSSQLNMSSLIEDITKLQSIRSRQGGVVTSNCI*
PpUGT73BT3	MASESHDDQLHFVLIPLMSPGHLIPMADNAKLLAERGVVVTIVTTPLNAIRIKPIIDRSIDSGLPIQLVQFSLPLQEFGLPEGCENMDSVPSRKLFWNFFAAVEKLQEPVEKFLETMKPNPSCIIADKYMAWTANIARKFRIPRLLFDGTSCFALLCSHNIQEHKVLESVSGSEPFLVPGLPDEIELTKFQLPGNMNPGSKDFSSLHDKVKESEEGAYGIVVNSFEELEFEYVKEFKKVNQGRVWSIGPVSLSNKTALDKAQRGNMASIDENKCLNWLGSWPQSSVVYACLGSLSQVTTLQLVELGLGLEASNRPFVWVISRNKIDEWEKWLLEDGFEERINGRGLLIHGWAPQVLILSHPAVGGFLTHCGWNSTLEGICAGIPMITWPLFAEQFYNEKFIVQVLKIGESVGAKVAIPLGEQEISKVLVKRVEFKEAIDKVMIKEGKEGEDRRKRARELAVLAKKATEGRGSSYLNMTLLIEDIRSCKANN*

Table S12. Summary of *S. grosvenorii* datasets used for transcriptome analysis

Sample tissues	Groups	Descriptions	BioProjects
fruit-1	a	3 days after anthesis	PRJEB23466
fruit-2	a	3 days after anthesis	PRJEB23466
fruit-3	a	5 days after anthesis	PRJNA773651
fruit-4	a	5 days after anthesis	PRJNA773651
fruit-5	a	5 days after anthesis	PRJNA773651
fruit-6	a	10 days after anthesis	PRJNA773651
fruit-7	a	10 days after anthesis	PRJNA773651
fruit-8	a	10 days after anthesis	PRJNA773651
fruit-9	a	15 days after anthesis	PRJNA773651
fruit-10	a	15 days after anthesis	PRJNA773651
fruit-11	a	15 days after anthesis	PRJNA773651
fruit-12	a	20 days after anthesis	PRJNA773651
fruit-13	a	20 days after anthesis	PRJNA773651
fruit-14	a	20 days after anthesis	PRJNA773651
fruit-15	a	20 days after anthesis	PRJEB23466
fruit-16	a	20 days after anthesis	PRJEB23466
fruit-17	a	25 days after anthesis	PRJNA773651
fruit-18	a	25 days after anthesis	PRJNA773651
fruit-19	a	25 days after anthesis	PRJNA773651
fruit-20	a	30 days after anthesis	PRJNA773651
fruit-21	a	30 days after anthesis	PRJNA773651
fruit-22	a	30 days after anthesis	PRJNA773651
fruit-23	a	35 days after anthesis	this study
fruit-24	a	35 days after anthesis	this study
root-1	b	roots	PRJEB23466
root-2	b	roots	PRJEB23466
root-3	b	roots	PRJEB23466
root-4	b	young roots	this study
root-5	b	young roots	this study
root-6	b	young roots	this study
stem-1	c	mature stems	this study
stem-2	c	mature stems	this study
stem-3	c	mature stems	this study
leaves-1	d	leaves beside fruits	PRJEB23466
leaves-2	d	leaves beside fruits	PRJEB23466
leaves-3	d	leaves of female plants	PRJEB23466
leaves-4	d	leaves of female plants	PRJEB23466
leaves-5	d	leaves of female plants	PRJEB23466
leaves-6	d	leaves of male plants	PRJEB23466
leaves-7	d	leaves of male plants	PRJEB23466
leaves-8	d	leaves of male plants	PRJEB23466
bud-1	e	bud	PRJNA1209606
bud-2	e	bud	PRJNA1209606
bud-3	e	bud	PRJNA1209606
bud-4	e	bud	PRJNA1209606
bud-5	e	bud	PRJNA1209606
bud-6	e	bud	PRJNA1209606

Table S13. Summary of species included in the comparative analysis, with notes on genome quality and manual curation of UGT73 annotations

Clade	Family	Species	Genome quality	Manual curation	UGT73 tandem array	UGT73 copy number
Core eudicots	Brassicaceae	*Arabidopsis thaliana*	chromosome	No	√	6
	Datiscaceae	*Datisca glomerata*	scaffold	Yes	×	0 (approx.)
	Begoniaceae	*Begonia loranthoides*	chromosome	Yes	×	0 (approx.)
	Cucurbitaceae	*Begonia darthvaderiana*	chromosome (contaminated)	No	√	4
		Begonia peltatifolia	chromosome	No	√	2 (approx.)
		Begonia masoniana	chromosome	No	√	2
		Citrullus lanatus	chromosome	No	√	7
		Cucumis sativus	chromosome	Yes	√	5
		Cucurbita pepo	chromosome	Yes	√	3 (approx.)
		Cucurbita moschata	chromosome	No	√	4
		Herpetospermum pedunculosum	chromosome	Yes	√	6
		Trichosanthes anguina	chromosome	No	√	7
		Sicyos edulis	chromosome	No	√	7
		Momordica charantia	chromosome	Yes	√	6
		Siraitia grosvenorii	chromosome	No	√	9
		Thladiantha pustulata	chromosome	No	√	4
		Gynostemma pentaphyllum	chromosome	No	√	6
	Coriariaceae	*Coriaria nepalensis*	chromosome	No	√	3
	Juglandaceae	*Cyclocarya paliurus*	chromosome	Yes	√	6
	Juglandaceae	*Juglans regia*	chromosome	Yes	√	4
	Betulaceae	*Corylus avellana*	chromosome	Yes	√	10
	Rosaceae	*Prunus persica*	chromosome	No	√	7
	Cannabaceae	*Cannabis sativa*	chromosome	No	√	11
	Fabaceae	*Glycine max*	chromosome	No	√	6
		Astragalus membranaceus	chromosome	No	√	9
	Vitaceae	*Vitis vinifera*	chromosome	No	√	4
	Rubiaceae	*Coffea arabica*	chromosome	No	√	4
	Lamiaceae	*Prunella vulgaris*	chromosome	Yes	√	3
	Asteraceae	*Stevia rebaudiana*	chromosome	Yes	√	3
	Araliaceae	*Aralia elata*	chromosome	No	√	6
	Campanulaceae	*Platycodon grandiflorus*	chromosome	Yes	√	11
	Apiaceae	*Centella asiatica*	chromosome	No	√	9
		Daucus carota	chromosome	No	√	3

	Caryophyllaceae	*Saponaria officinalis*	chromosome	No	×	1
	Chenopodiaceae	*Beta vulgaris*	chromosome	No	×	1
Basal eudicots	Ranunculaceae	*Coptis chinensis*	chromosome	No	×	0
		Aquilegia coerulea	scaffold	No	×	0
	Papaveraceae	*Papaver somniferum*	chromosome	No	×	1
Monocots	Poaceae	*Oryza sativa*	chromosome	No	×	0
Magnoliids	Aristolochiaceae	*Aristolochia fimbriata*	chromosome	No	×	0
	Lauraceae	*Persea americana*	chromosome	No	×	0
ANA-grade	Nymphaeaceae	*Nymphaea colorata*	chromosome	No	×	0

Note: In this table, copy numbers are marked as “approx.” for species with highly fragmented assemblies or incomplete gene models. To assess the robustness of our findings to genome data quality, we conducted a sensitivity analysis using a subset of nine species with chromosome-level assemblies and high BUSCO scores (>91%), as summarized in Figure 6B. Manual curation of the syntenic block “Oxred-PE-UGT73-TLP” confirmed that the key observations—retention of this region and consistency in tandem duplication patterns—remained stable across variations in genome data quality.

References

[1] a) J. M. Augustin, S. Drok, T. Shinoda, K. Sanmiya, J. K. Nielsen, B. Khakimov, C. E. Olsen, E. H. Hansen, V. Kuzina, C. T. Ekstrøm, T. Hauser, S. Bak, *Plant Physiol.* **2012**, *160* (4), 1881, https://doi.org/10.1104/pp.112.202747; b) B. Petit, A.-C. Mitaine-Offer, C. Delaude, T. Miyamoto, C. Tanaka, M.-A. Lacaille-Dubois, *Phytochemistry* **2019**, *162*, 260, https://doi.org/10.1016/j.phytochem.2019.03.020.

[2] Y. Duan, W. Du, Z. Song, R. Chen, K. Xie, J. Liu, D. Chen, J. Dai, *Acta Pharmaceutica Sinica B* **2023**, *13* (1), 271, https://doi.org/10.1016/j.apsb.2022.05.015.
